# Supplementary material for: Differentiable land model reveals global environmental controls on latent ecological functions
Source: Nat Commun. 2026 May 21;17:6670. doi: 10.1038/s41467-026-73395-4 (PMC13381539; doi:10.1038/s41467-026-73395-4)
Supplement: Supplementary file 1 — Supplementary information [file 41467_2026_73395_MOESM1_ESM.pdf]

Supplementary Information for "Differentiable Land Model Reveals Global Environmental Controls on Latent Ecological Functions"

Jianing Fang, Kevin Bowman, Wenli Zhao, Xu Lian, and Pierre Gentine

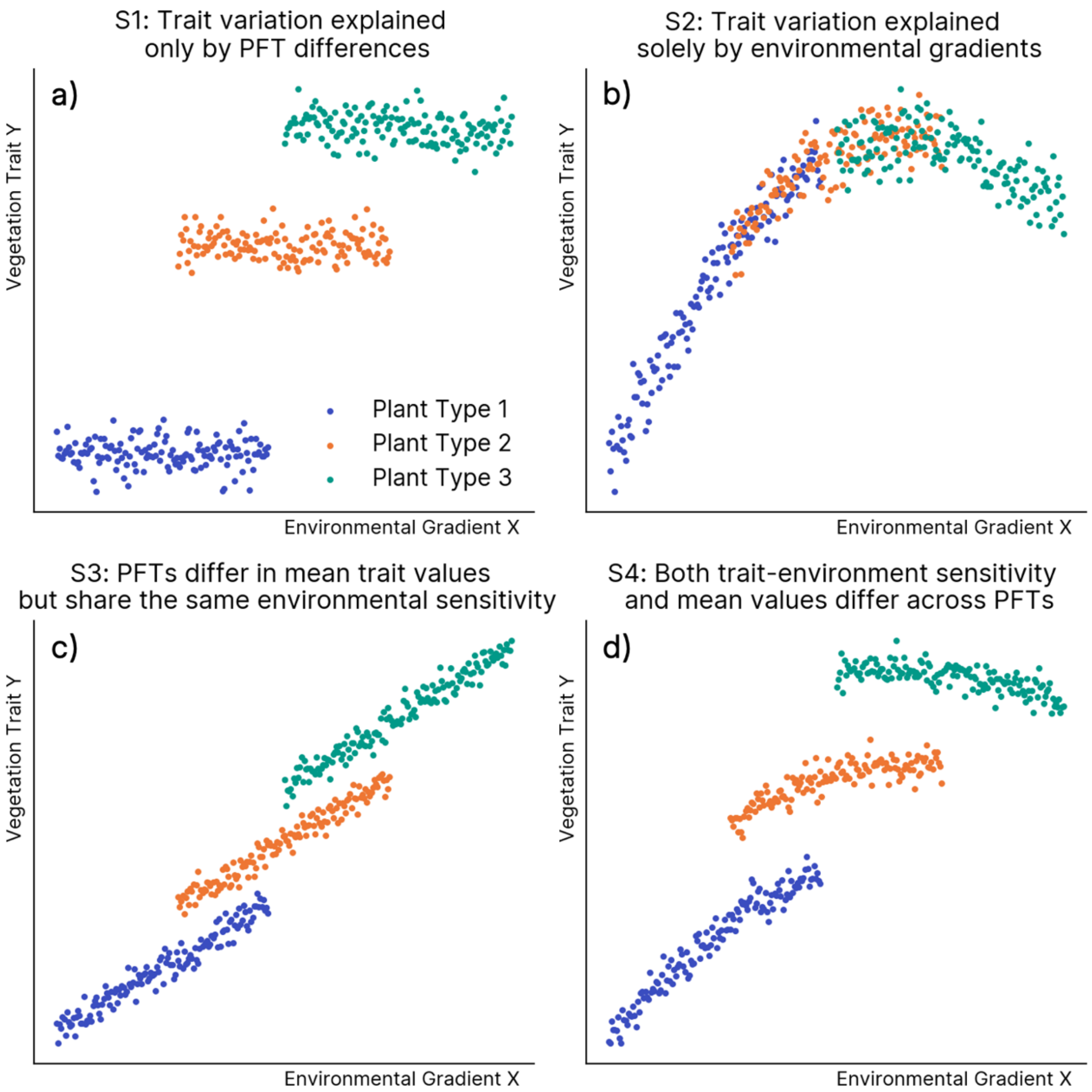

Fig. S1 | Conceptual illustration of four hypothetical scenarios regarding the roles of plant functional types (PFTs) and environmental gradients in shaping the spatial distribution of plant functional traits. a) S1 – PFT-only control: Trait values vary between PFTs but remain largely constant within each PFT, showing minimal response to environmental gradients. b) S2 – Environment-only control: Trait values respond continuously to environmental gradients in a universal way, independent of PFT classification. c) S3 – Consistent scaling across PFTs: Mean trait values differ among PFTs, but trait–environment relationships (i.e., the slopes) are similar across PFTs. d) S4 – PFT-specific trait–environment relationships: Both mean trait values and the sensitivity of traits to environmental gradients differ among PFTs, resulting in distinct trait–environment relationships for each PFT.

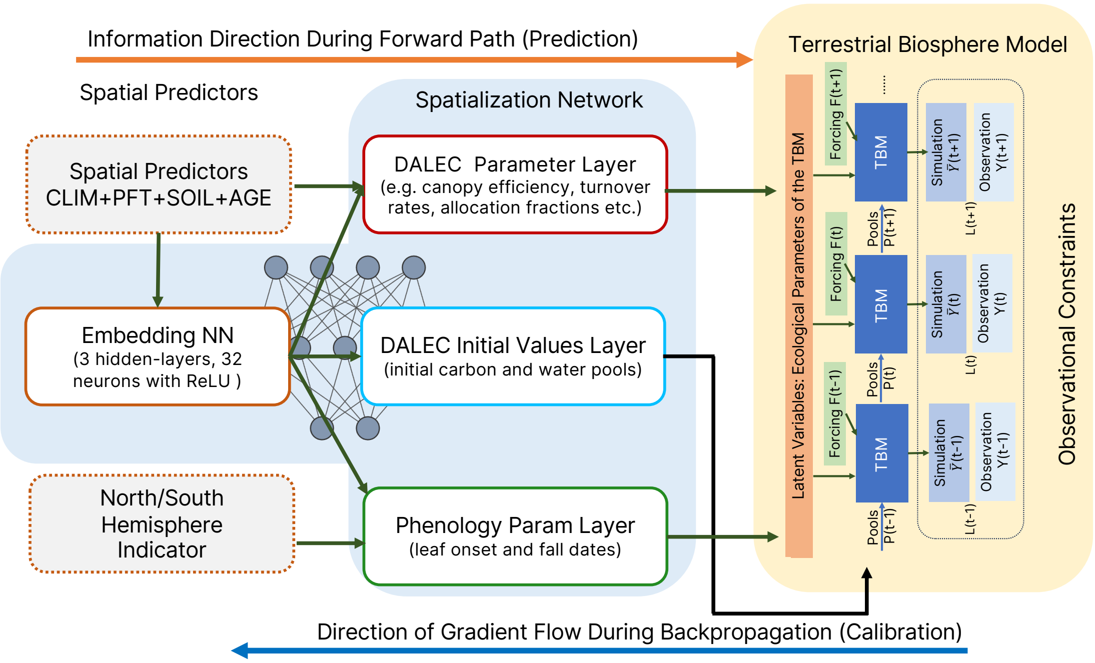

Fig. S2 | The detailed design of the spatialization network. The spatialization network (shown in blue) consists of an embedding NN that first uses spatial predictors to learn an embedding of environmental conditions at each 0.25° pixel. The embedding is then passed into three output layers to predict, respectively, the initial conditions (n=7, e.g., carbon and water stock in each pool at time zero), the ecological parameters (n=31, e.g., allocation fractions to different pools, canopy efficiency, Q10, and turnover rates), and the phenology parameters of the DALEC model (n=2, leaf onset & fall days). A North/South hemisphere indicator variable identifies the austral and boreal phenology cycles. The predicted initial conditions and model parameters are passed into the differentiable DALEC model to simulate ecosystem dynamics and compute an optimization target based on observational constraints. During the backward pass, the gradient of the neural parameters with respect to the loss function is used to update each layer in the spatialization NN.

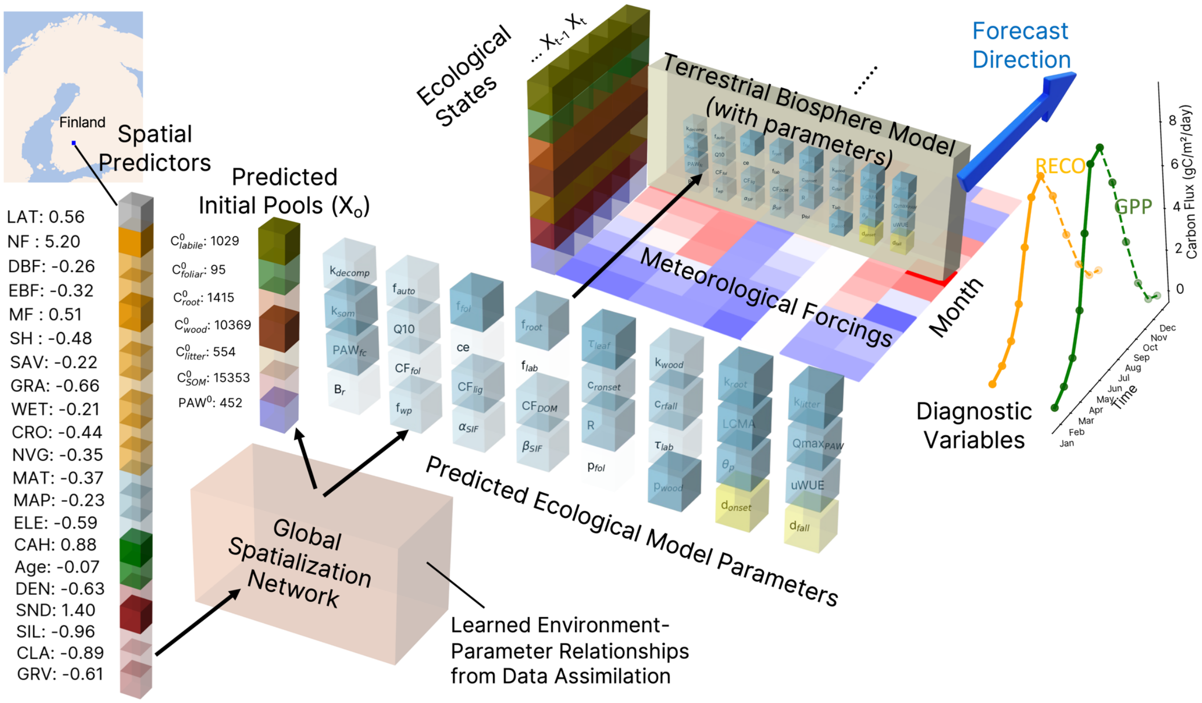

Fig. S3 | Example of the internal data flow through DifferLand for a sample grid cell in central Finland. A vector of standardized spatial predictors—including PFT fractions, climatological variables, forest age, maximum canopy height, and soil texture properties—is fed into the spatialization neural network to predict both the initial ecosystem states at the start of the simulation and the ecological parameters specific to the grid cell. These predicted ecological parameters are then used to parameterize the differentiable terrestrial biosphere model. The model's differential equations are integrated forward in time, driven by monthly meteorological forcings, to update ecosystem states and simulate monthly carbon and water exchanges within the grid cell. This process is repeated at each grid cell across the global land surface.

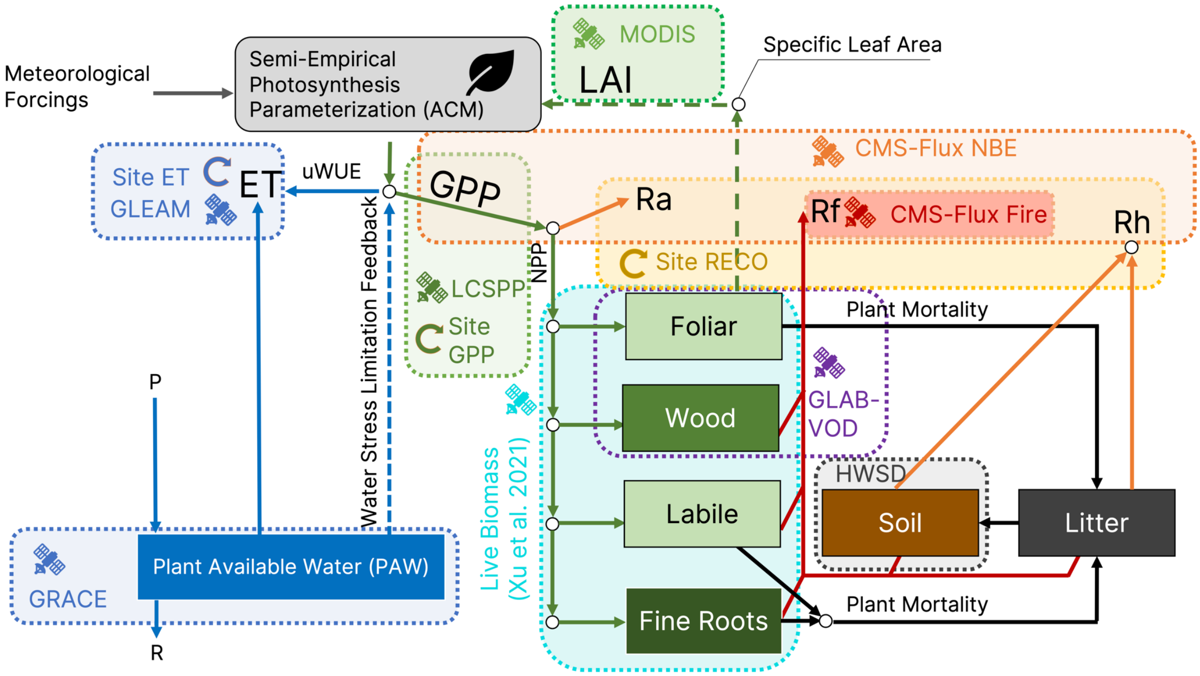

Fig. S4 | Structure of the DALEC model and assimilated observational constraints. DALEC is an intermediate-complexity terrestrial biosphere model that simulates key ecosystem processes including photosynthesis (via the semi-empirical Aggregate Canopy Model, ACM), carbon allocation, mortality, decomposition, and fire disturbance. It also incorporates water limitation effects on carbon cycling. The model tracks six carbon pools and one soil water reservoir (shown as solid rectangular boxes), which serve as the model's prognostic state variables. Global satellite-informed constraints are indicated by the satellite icon, and site-level eddy covariance constraints are represented by the cyclical arrow symbol. Shaded dashed boxes illustrate the relationships between simulated state variables and the observational data used to constrain corresponding model components. Vegetation Optical Depth (VOD) is not assimilated in the default configuration presented in the main text but can optionally be included as an additional constraint.

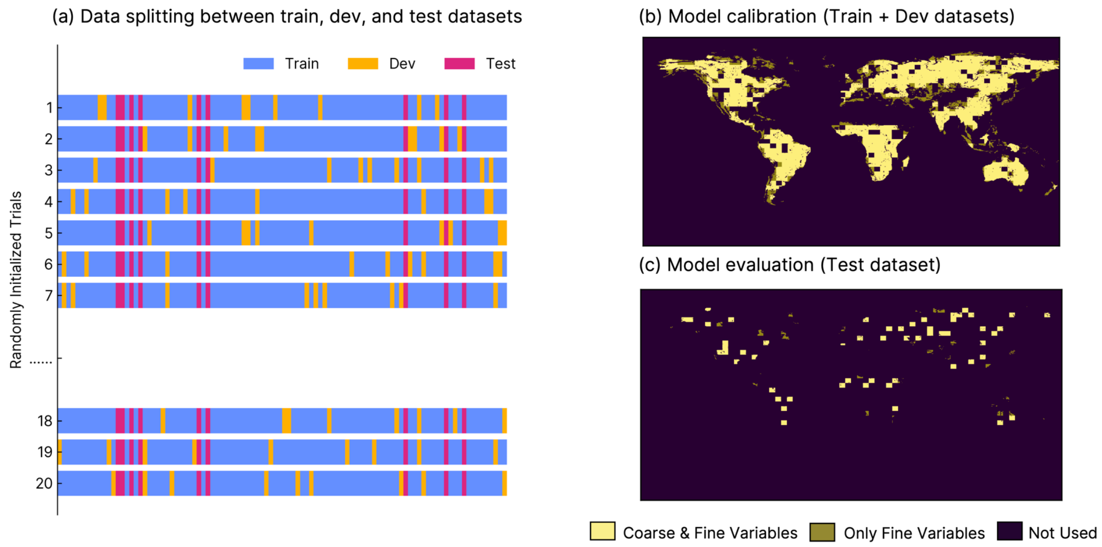

Fig. S5 | Data splitting and the multi-resolution data assimilation approach. We first reserved 10% of all the available 4×5° patches for independent model testing. We then randomly sampled 90% of the remaining 4×5° patches for model training (81% of the total data) for each randomly initialize trials and used the rest of the patches for model development and hyperparameter tuning (9% of total data). The training and development sets (but not the test set) are then combined to retrain the model to obtain latent ecological parameters and environment-parameter relationships for further analysis. (a) shows schematics of data splitting and bootstrapping for 20 randomly initialized trials. (b) and (c) show the locations of data used for calibration and evaluation. Pixels with more than 80% non-vegetated surface are removed from the experiment. A 4×5° patch is included during training as a minibatch (containing a maximum of 320 nested 0.25° samples) if at least 10% of the nested 0.25° cells are vegetated land surface, but we only assimilate coarse resolution variables (CMS-Flux NEE and GRACE EWT) as a batch level constraint if at least 80% of the 0.25-degree nested grids are vegetated land surface.

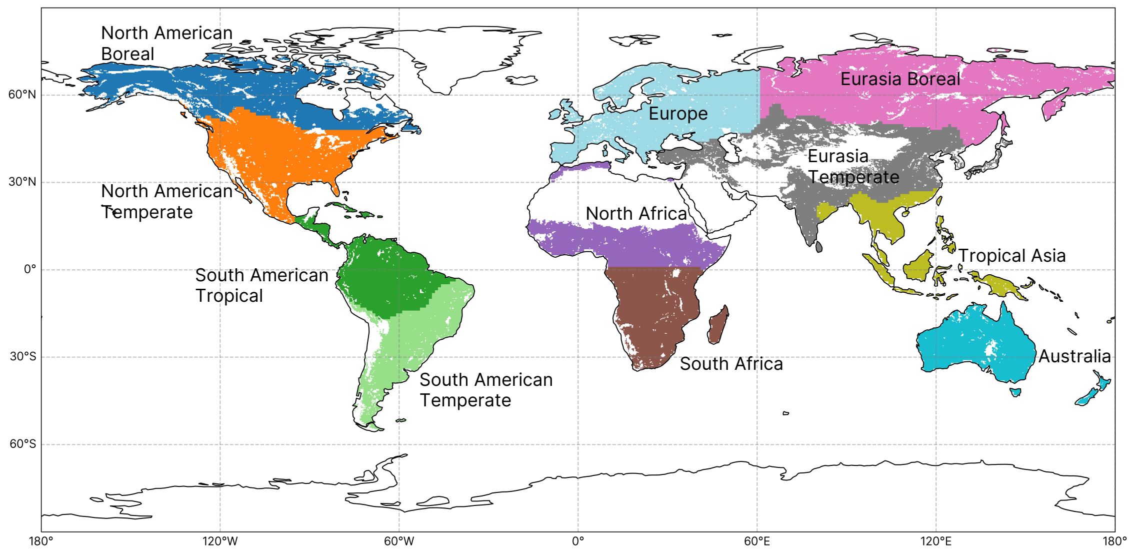

Fig. S6 | The spatial extent of the TRANSCOM regions.

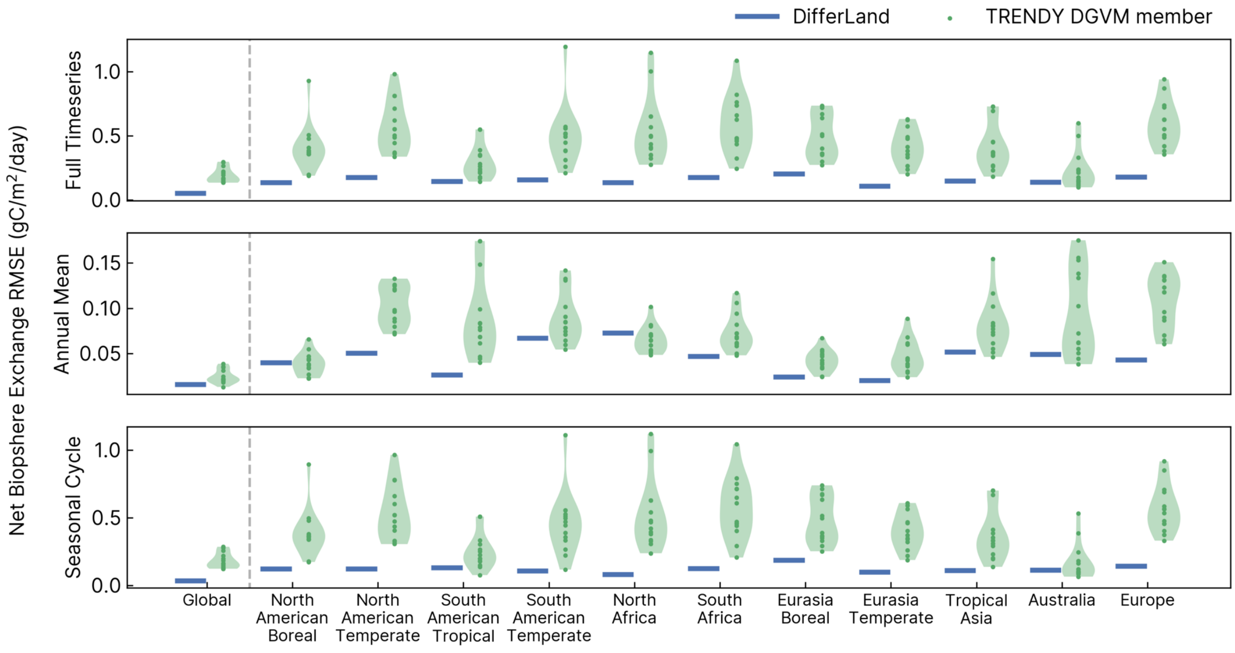

Fig. S7 | Comparison of net biosphere exchange (NBE) performance between DifferLand and TRENDY DGVMs across regions and temporal scales. Violin plots show the root mean square error (RMSE; gC m^{-2} day^{-1}) of NBE simulations compared to benchmark observations from the CMS-Flux atmospheric CO_2 inversion for the period 2010–2022. Errors are evaluated over: (top) the full time series, (middle) annual mean, and (bottom) seasonal cycle. Each dot represents an individual TRENDY v12 model member (n=14, see Table S4). Each green violin represents the distribution of RMSE from individual TRENDY dynamic global vegetation model (DGVM) members, while blue bars indicate the RMSE of DifferLand. Evaluations are conducted globally and across 11 TRANSCOM regions. Lower RMSE values indicate better agreement with the observational benchmark.

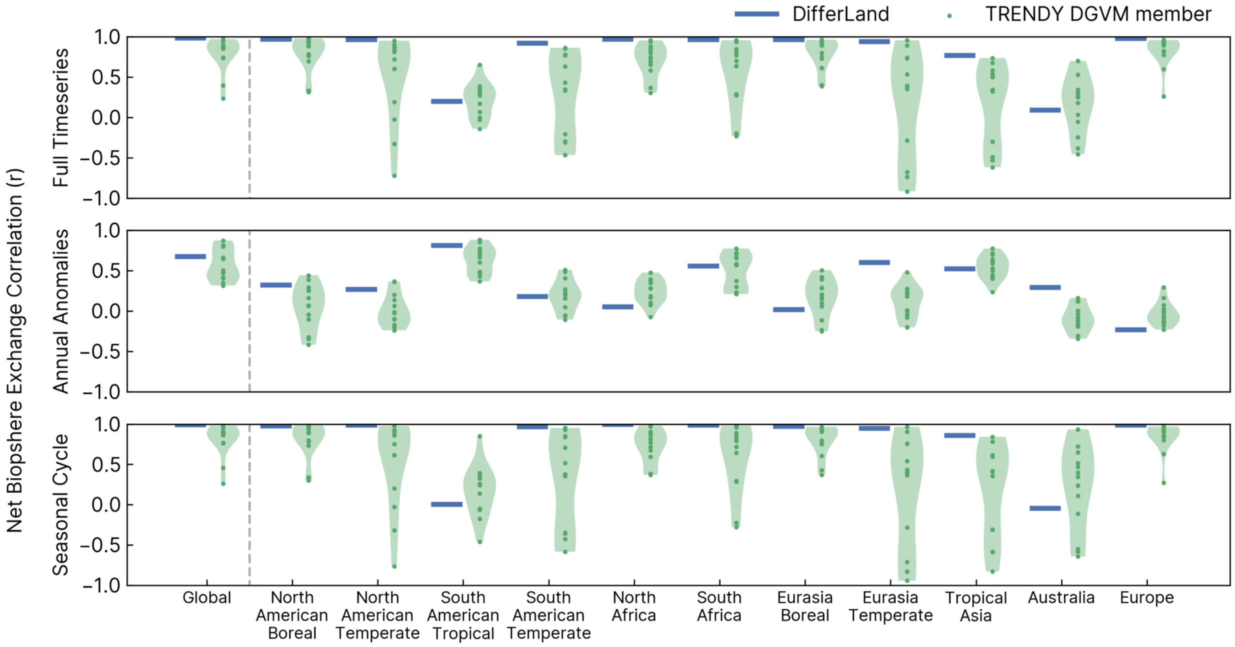

Fig. S8 | Comparison of net biosphere exchange (NBE) correlation between DifferLand and TRENDY DGVMs across regions and temporal scales. Violin plots show the Pearson correlation coefficient (r) between simulated and observed NBE from the CMS-Flux atmospheric CO_2 inversion over the period 2010–2022. Correlations are evaluated over: (top) the full time series, (middle) annual anomalies, and (bottom) seasonal cycles. Each dot represents an individual TRENDY v12 model member (n=14, see Table S4). Each green violin represents the distribution of correlations from individual TRENDY dynamic global vegetation model (DGVM) members, while blue bars indicate the correlation achieved by DifferLand. Evaluations are performed globally and across 11 TRANSCOM regions. Higher correlation values indicate better temporal agreement with the benchmark observations.

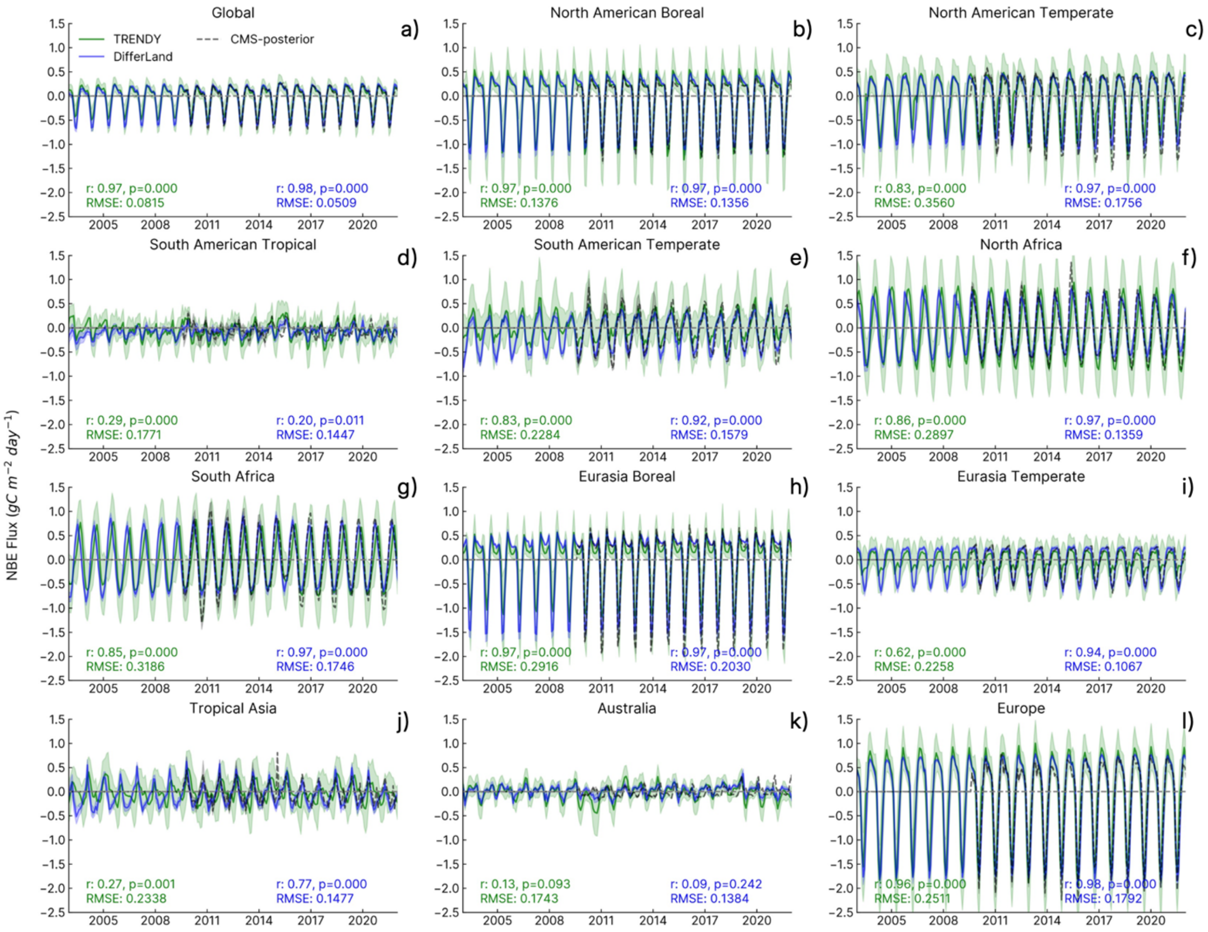

Fig. S9 | Time series of net biosphere exchange (NBE) from DifferLand and TRENDY models against CMS-Flux atmospheric CO_2 inversion. Time series of NBE (gC m^{-2} day^{-1}) from DifferLand (blue) and the multi-model mean of TRENDY DGVMs (green) from 2003 to 2022, as well as the benchmark CMS-Flux posterior from the available period of 2010-2022 (black). a) shows the global mean results; b-l) display the results for each of the TRANSCOM region. Solid lines represent the ensemble means, while the error bands indicate the range of 1 standard deviation for both the DifferLand and TRENDY ensembles. Pearson correlation coefficients (r), *p*-values, and RMSE values are reported for DifferLand and TRENDY ensemble mean relative to CMS-Flux over 156 months.

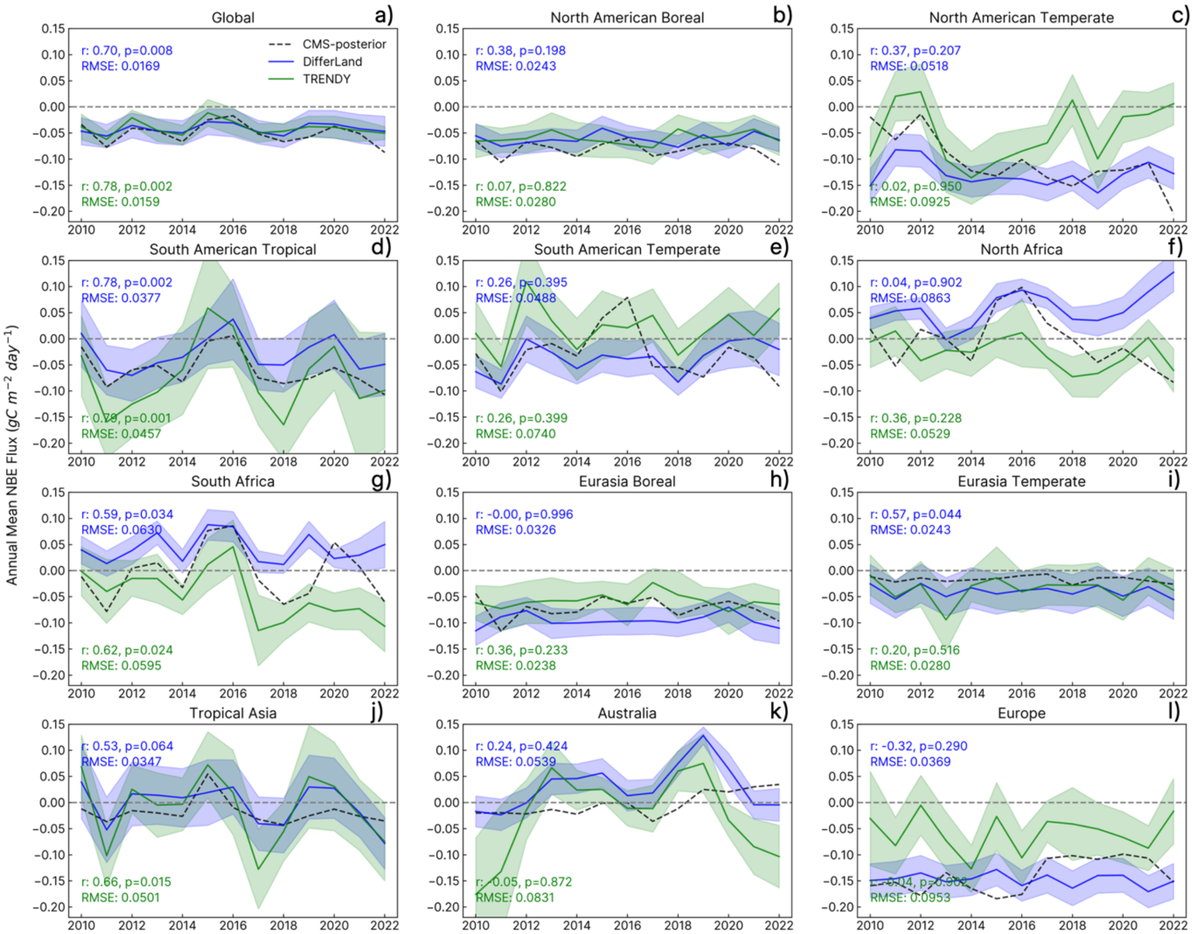

Fig. S10 | Annual mean net biosphere exchange (NBE) from DifferLand and TRENDY models against CMS-Flux atmospheric CO_2 inversion. a) shows the global mean results; b-l) display the results for each of the TRANSCOM region. Solid lines represent the ensemble means, while the error bands indicate the range of 1 standard deviation for both the DifferLand and TRENDY ensembles. Pearson correlation coefficients (r), *p*-values, and RMSE values are reported for DifferLand and TRENDY ensemble mean relative to CMS-Flux over 13 annual data points.

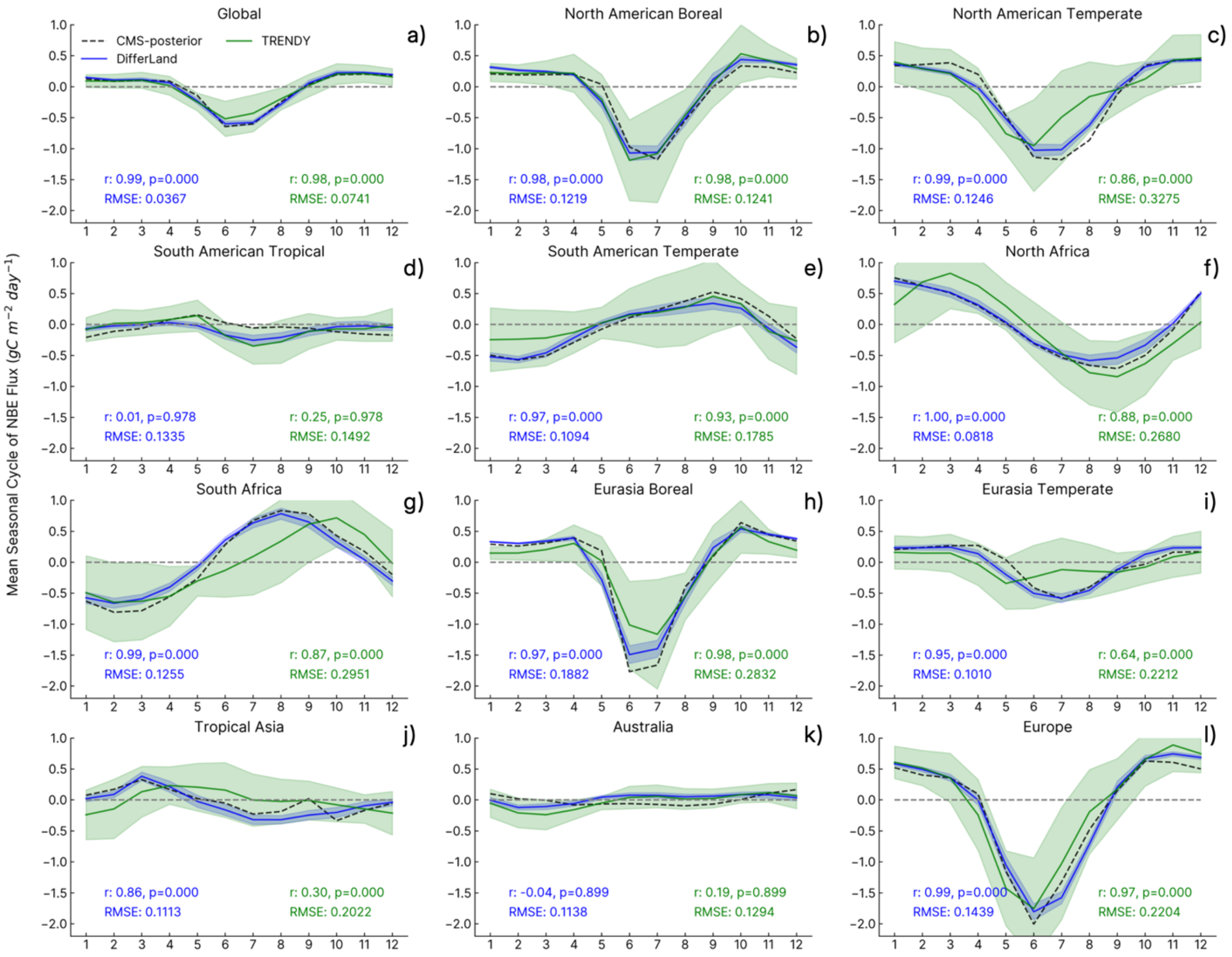

Fig. S11 | Mean seasonal cycle of net biosphere exchange (NBE) from DifferLand and TRENDY models against CMS-Flux atmospheric CO_2 inversion. a) shows the global mean results; b-l) display the results for each of the TRANSCOM region. Solid lines represent the ensemble means, while the error bands indicate the range of 1 standard deviation for both the DifferLand and TRENDY ensembles. Pearson correlation coefficients (r), *p*-values, and RMSE values are reported for DifferLand and TRENDY ensemble mean relative to CMS-Flux over 13 annual data points.

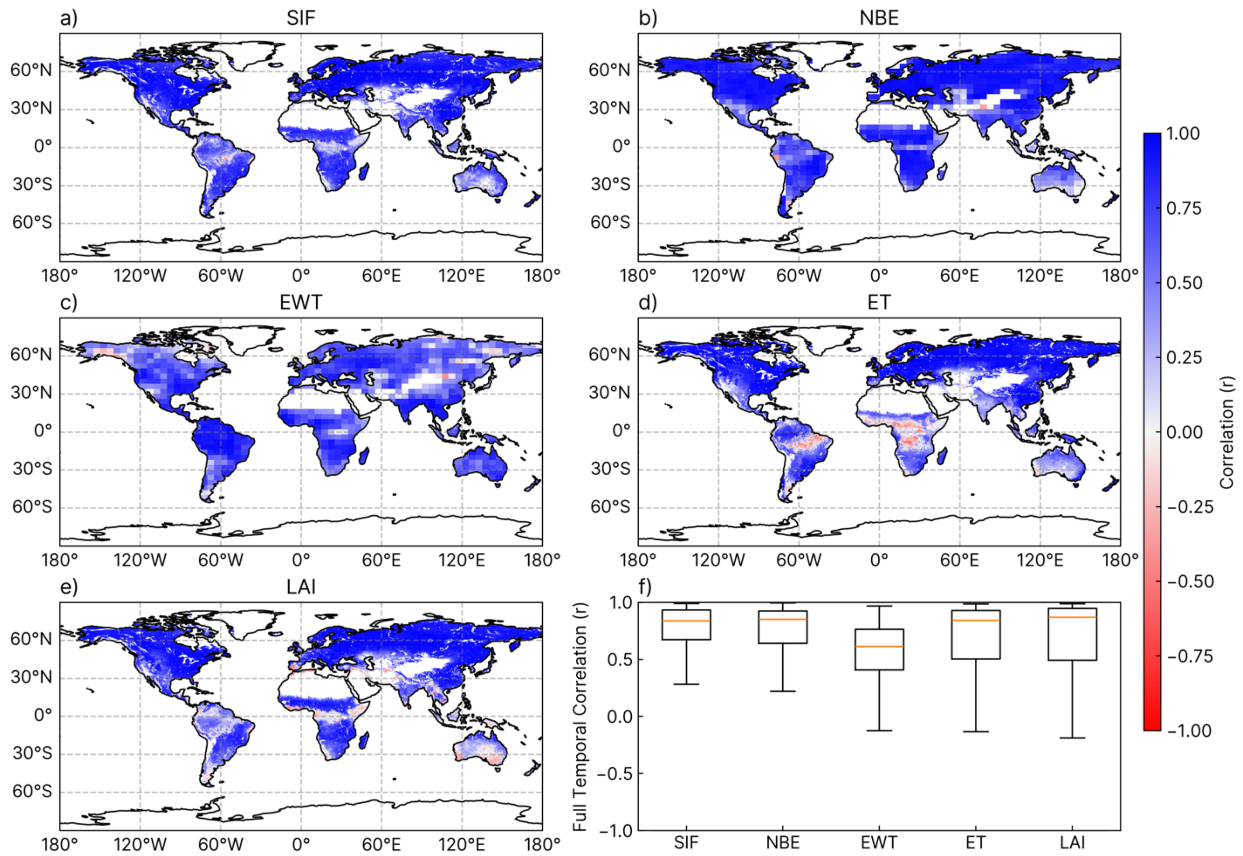

Fig. S12 | The pixelwise temporal correlation between simulated and observed variables. a) SIF, b) NBE, c) EWT, d) ET, e) LAI. The panels display the average correlation values across the n=10 out of the 20 ensemble members using the full set of predictors with the lowest training loss. f) Shows the distribution of pixelwise correlations across pixels. Each orange line represents the median, while each box encompasses the first and third quartiles of the distribution. The whiskers extend to points within 1.5 times the interquartile range from the box.

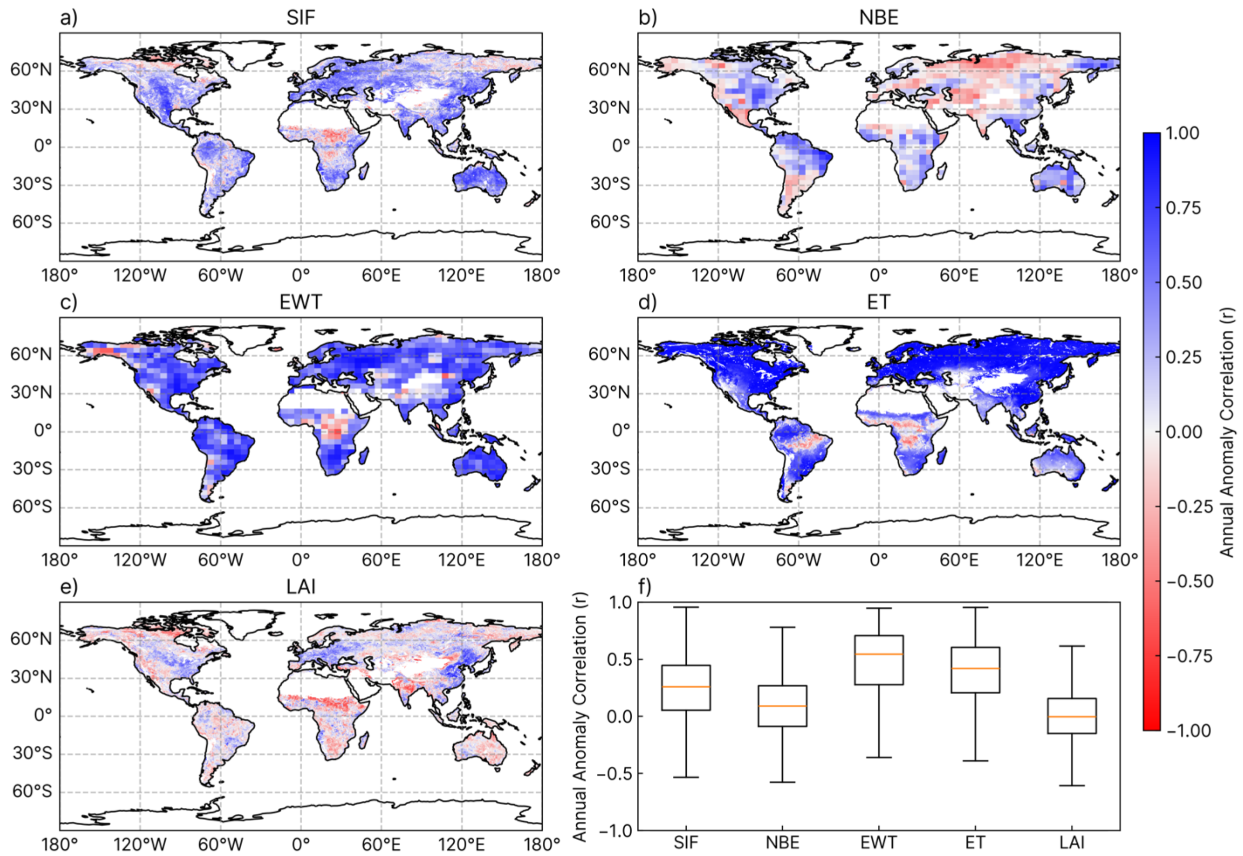

Fig. S13 | The pixelwise temporal correlations of the annual anomalies of simulated and observed variables. a) SIF, b) NBE, c) EWT, d) ET, e) LAI. The panels display the average correlation values across the n=10 out of the 20 ensemble members using the full set of predictors with the lowest training loss. f) Shows the distribution of pixelwise correlations across pixels. Note: The low correlation in interannual LAI anomalies arises from DALEC2's fixed leaf onset and senescence[1], which do not respond to interannual atmospheric variability. This limitation could be addressed by incorporating dynamic leaf carbon allocation within a more realistic phenology module (e.g., Norton et al., 2023[2]). Each orange line represents the median, while each box encompasses the first and third quartiles of the distribution. The whiskers extend to points within 1.5 times the interquartile range from the box.

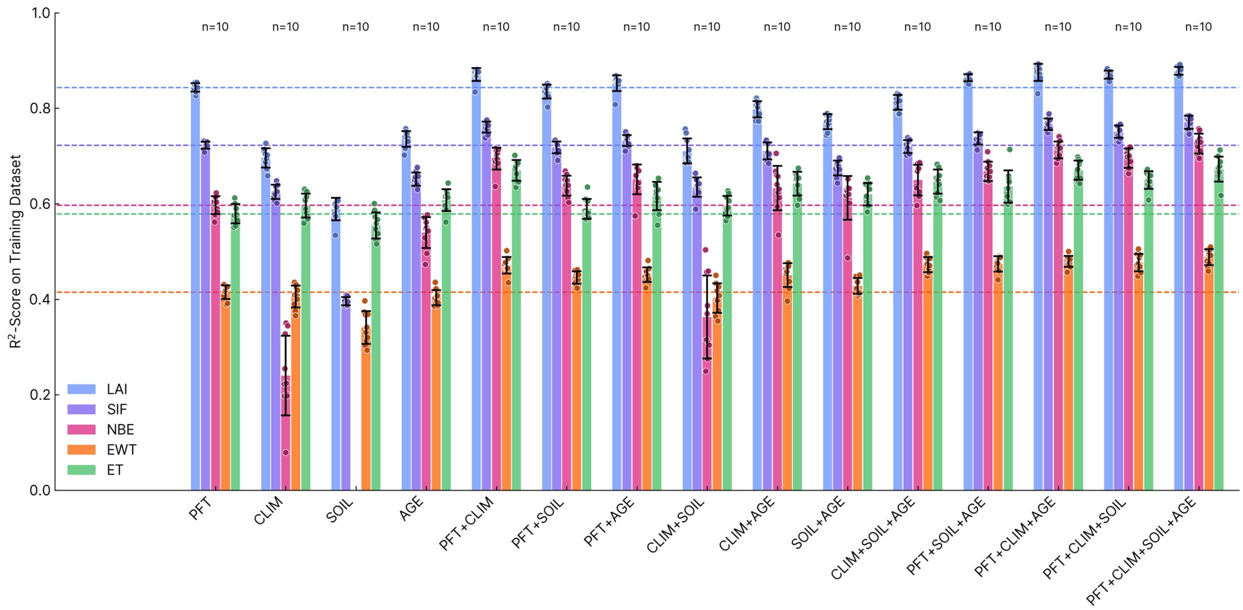

Fig. S14 | The R^2-score of DifferLand simulations for different predictor combinations on LAI, SIF, NBE, EWT, and ET constraints, evaluated on the training pixels. The columns show the ensemble mean of the n=10 out of the 20 runs for each configuration with the lowest training loss, whereas the error bars indicate the standard deviation. The dashed lines correspond to the performance of the model configuration using only PFT fractions to highlight the additional contributions of environmental predictors for parameter spatialization on top of PFTs. Variables are aggregated along both spatial and temporal dimensions before computing the metric. Individual model members are plotted as dots (n=10), and the error bars represent 1 standard deviation away from the means.

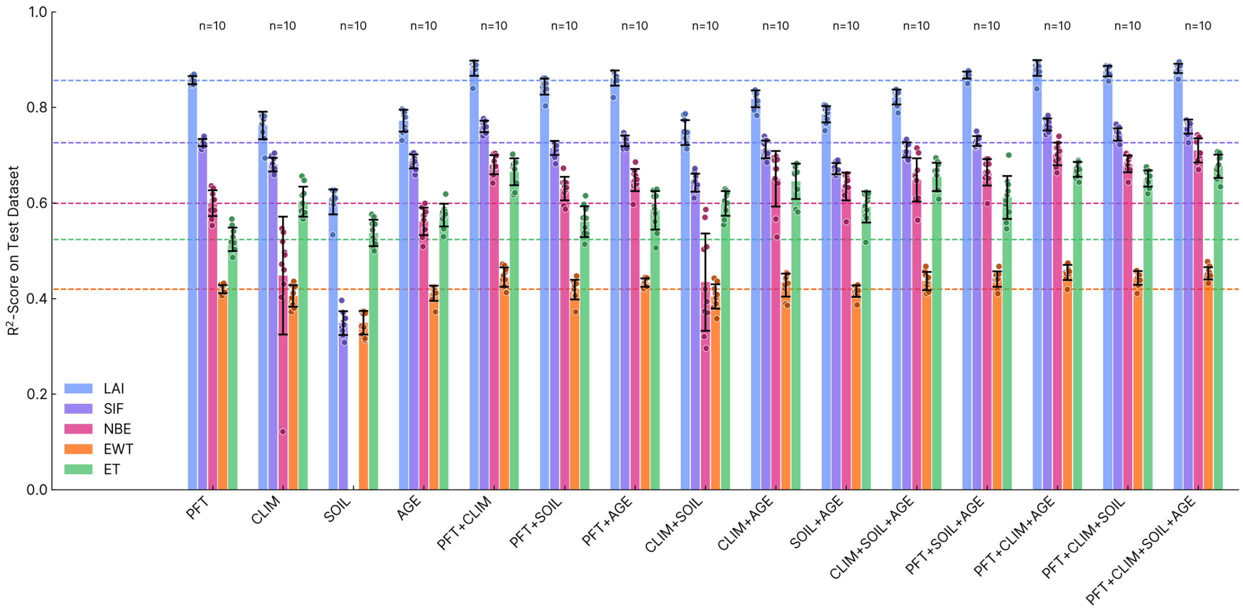

Fig. S15 | The R^2-score of DifferLand simulations for different predictor combinations on LAI, SIF, NBE, EWT, and ET constraints, evaluated on the test pixels. Individual model members are plotted as dots (n=10), and the error bars represent 1 standard deviation away from the means

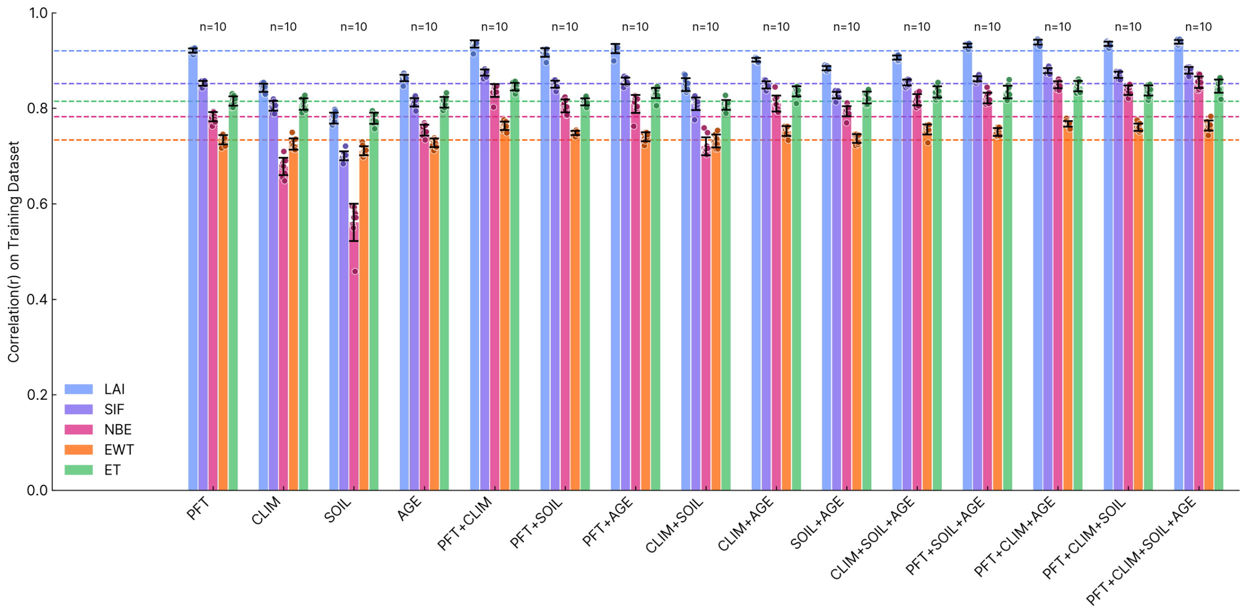

Fig. S16 | The Pearson Correlation of DifferLand simulations for different predictor combinations on LAI, SIF, NBE, EWT, and ET constraints, evaluated on the training pixels. Variables are aggregated along both spatial and temporal dimensions before computing the metric. Individual model members are plotted as dots (n=10), and the error bars represent 1 standard deviation away from the means.

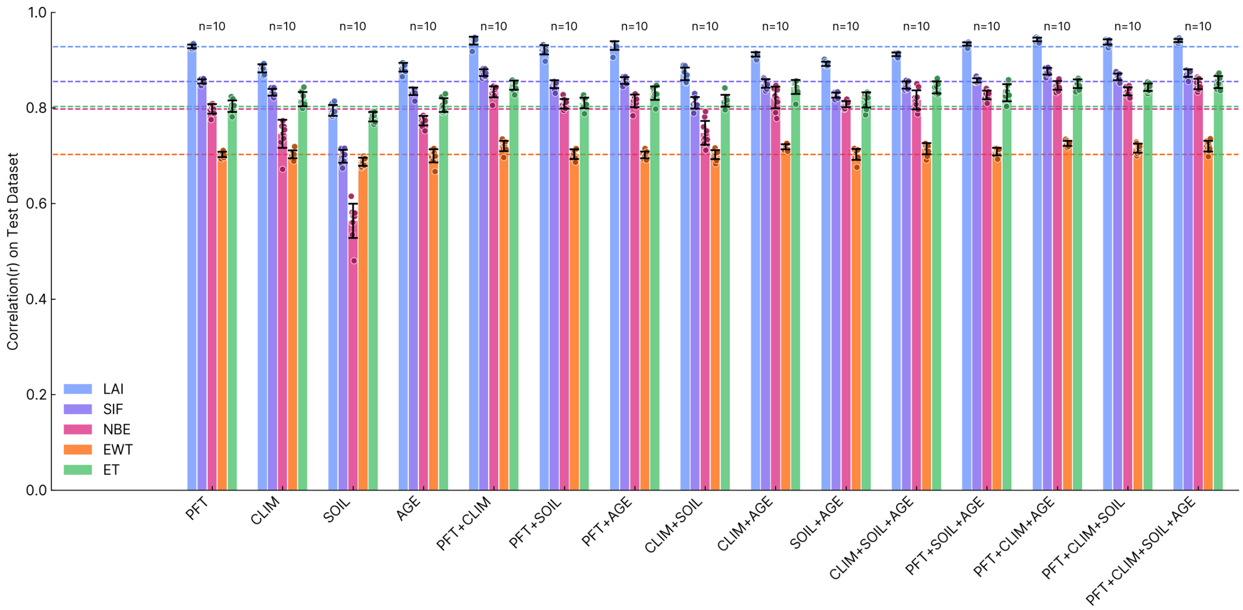

Fig. S17 | The Pearson Correlation of DifferLand simulations for different predictor combinations on LAI, SIF, NBE, EWT, and ET constraints, evaluated on the test pixels. Variables are aggregated along both spatial and temporal dimensions before computing the metric. Individual model members are plotted as dots (n=10), and the error bars represent 1 standard deviation away from the means.

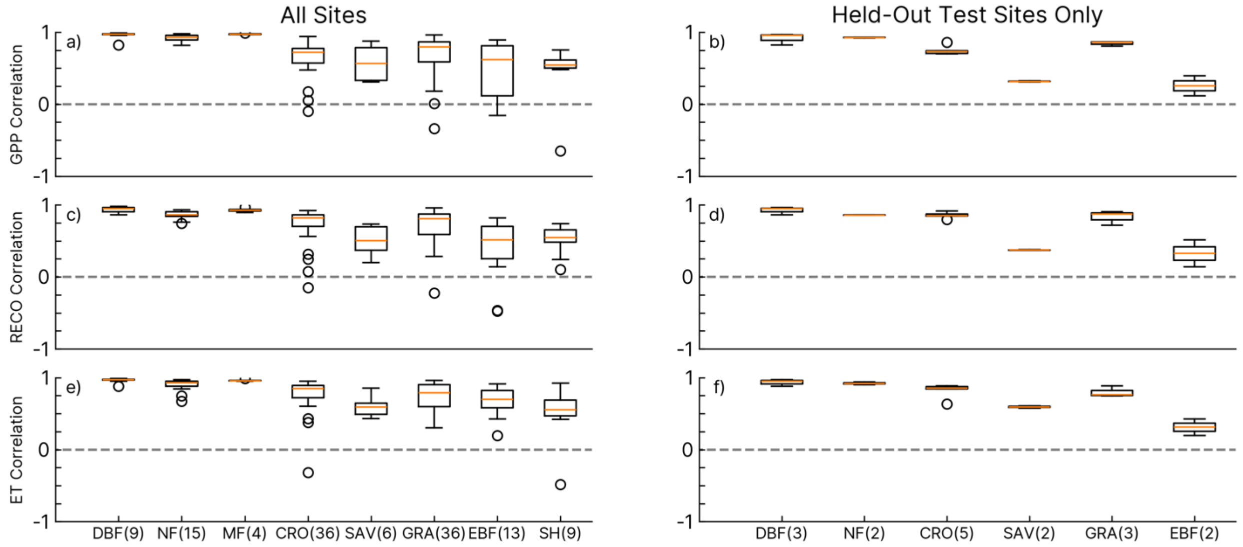

Fig. S18 | Correlation between DifferLand modeled GPP, RECO, and ET and eddy covariance estimates. The left column shows all eddy covariance sites, while the right column shows only sites that overlap with the test pixels withheld from training. Each orange line represents the median, while each box encompasses the first and third quartiles of the distribution. The whiskers extend to points within 1.5 times the interquartile range from the box. Points that lie past the whiskers are shown as fliers. The number of sites for each PFT is marked the parenthesis. a,c,e) show results for all sites, while b,d,f) display only sites withheld from training.

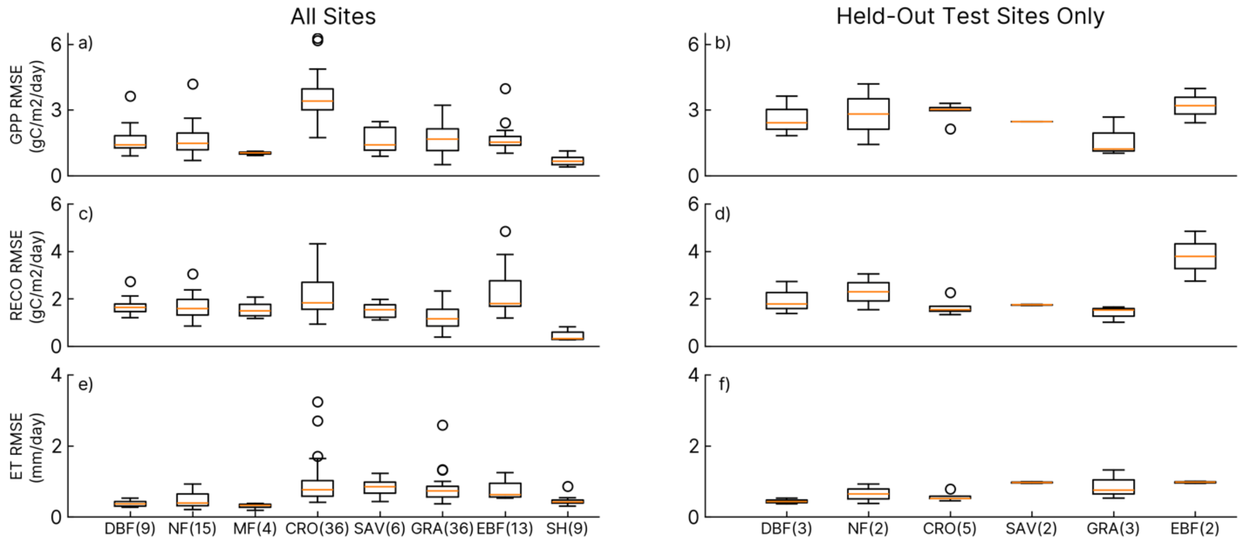

Fig. S19 | Root mean square error (RMSE) between DifferLand modeled GPP, RECO, and ET and eddy covariance estimates. The left column shows all eddy covariance sites, where the right column shows only sites that overlap with the test pixels withheld from training. Each orange line represents the median, while each box encompasses the first and third quartiles of the distribution. The whiskers extend to points within 1.5 times the interquartile range from the box. Points that lie past the whiskers are shown as fliers. The number of sites for each PFT is marked the parenthesis. a,c,e) show results for all sites, while b,d,f) display only sites withheld from training.

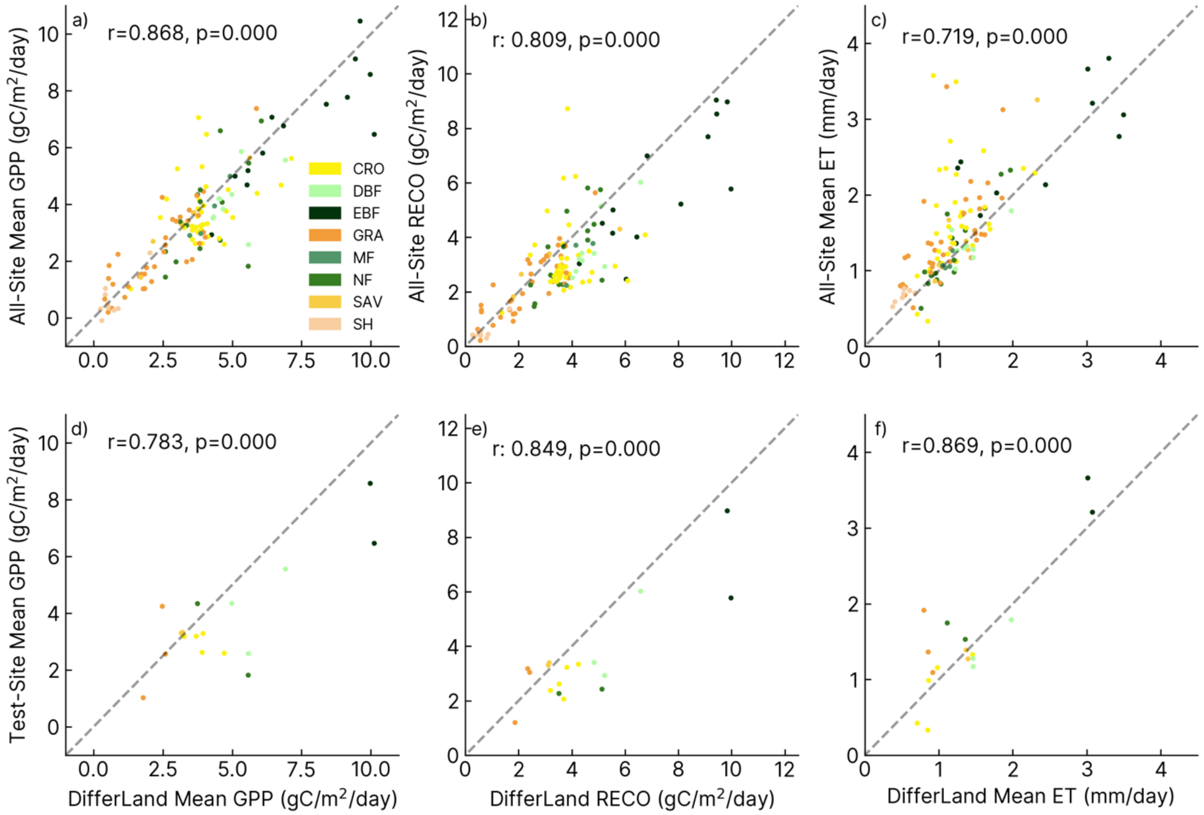

Fig. S20 | Comparing mean eddy-covariance (EC) estimates of GPP, RECO, and ET with DifferLand simulations at the closest 0.25° pixel. a-c) compares the results across all sites, while d-f) displays the results for only the sites that were withheld from model calibration. DifferLand simulations are computed as the mean from n=10 out of the 20 runs using the full set of predictors with lowest training loss. The dots are color coded by IGBP land cover type of each site. The dashed diagonal line is the 1:1 line. A total of n=128 sites with at least 12 months of measurements between 2003-2023, including n=17 sites withheld from training, are selected for this analysis (i.e., excluding sites with only shorter records).

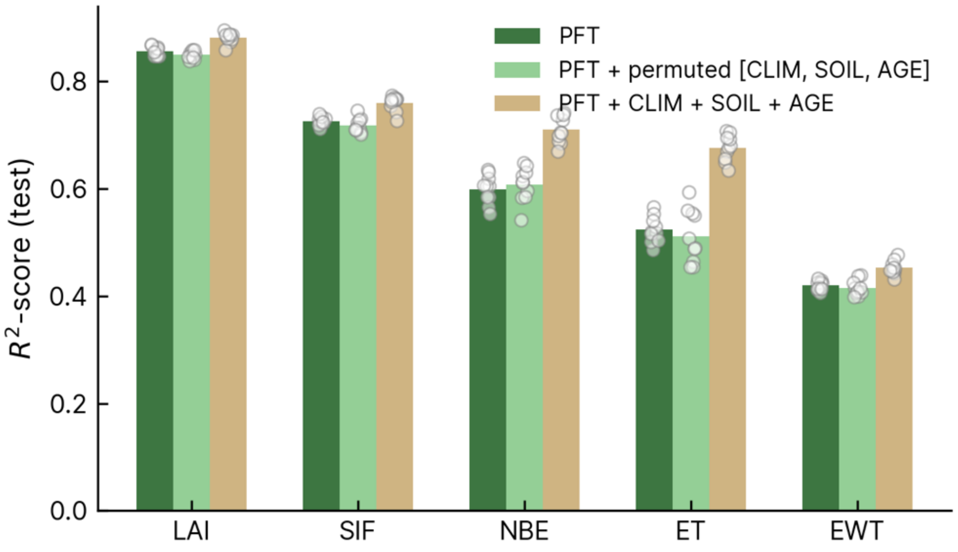

Fig. S21 | Evaluating against the null hypothesis that increasing degrees of freedom in the spatialization neural network *per se* explains the model performance gain compared with the PFT-online baseline. We randomly permuted the CLIM, SOIL, and AGE predictors to destroy the spatial pattern but kept the same input dimensionality and parameter count in the neural network as the PFT+CLIM+SOIL+AGE configuration. We found the PFT + permuted[CLIM, SOIL, AGE] setup offered no performance gain compared to the PFT-only baseline, rejecting the null hypothesis. Dots represent individual model members (n=10), bars represent ensemble means.

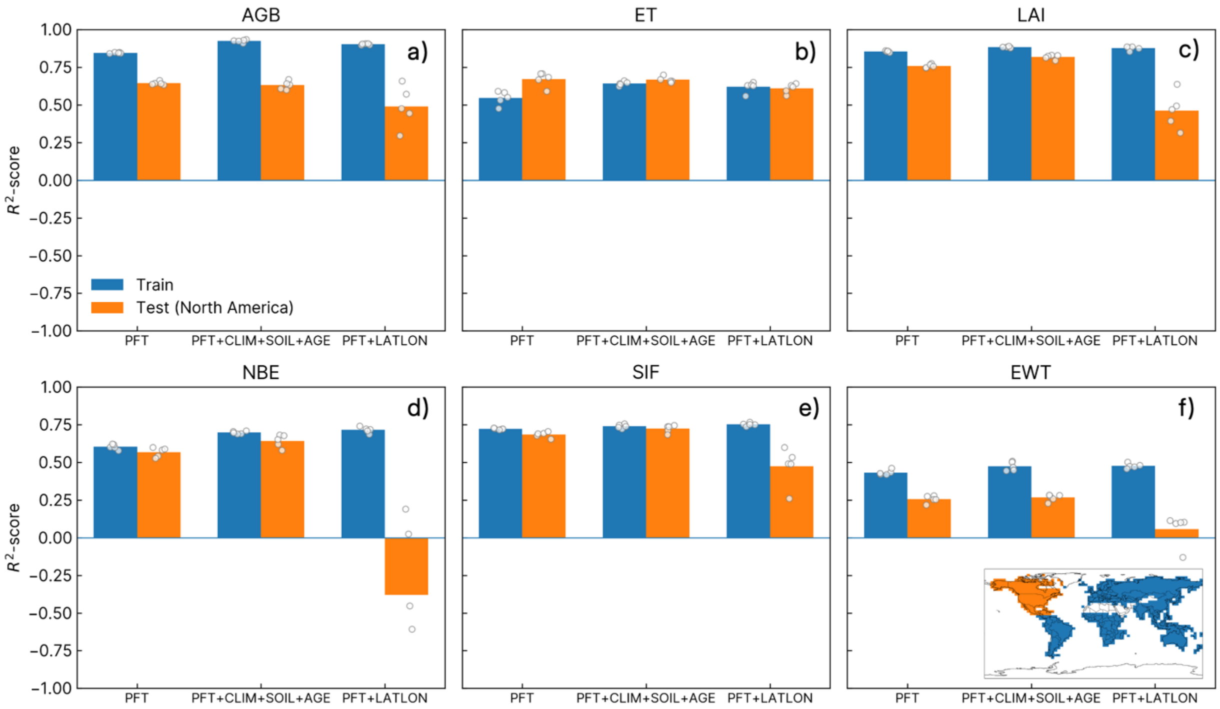

Fig. S22 | Evaluating DifferLand against the null hypothesis of artificial performance gain due to spatial autocorrelation and confounding variables. The PFT+CLIM+SOIL+AGE setup was compared with the PFT+LATLON setup, which uses the latitude and longitude coordinates in addition to the PFT variables. We trained both models with only pixels in Eurasia, Oceania, Africa, and South America. We then evaluated model performance on North America as an out-of-sample generalization test. We found that although the PFT+CLIM+SOIL+AGE and PFT+LATLON configurations achieved near equal performance on the training set, the PFT+LATLON configurations that simply memorized the within-PFT spatial variations in the training set generalized poorly on the out-of-sample test. In contrast, the PFT+CLIM+SOIL+AGE configuration generalized well in the unseen continent. Dots represent individual model members (n=5), bars represent ensemble means. a) AGB; b) ET; c) LAI; d) NBE; e) SIF; f) EWT.

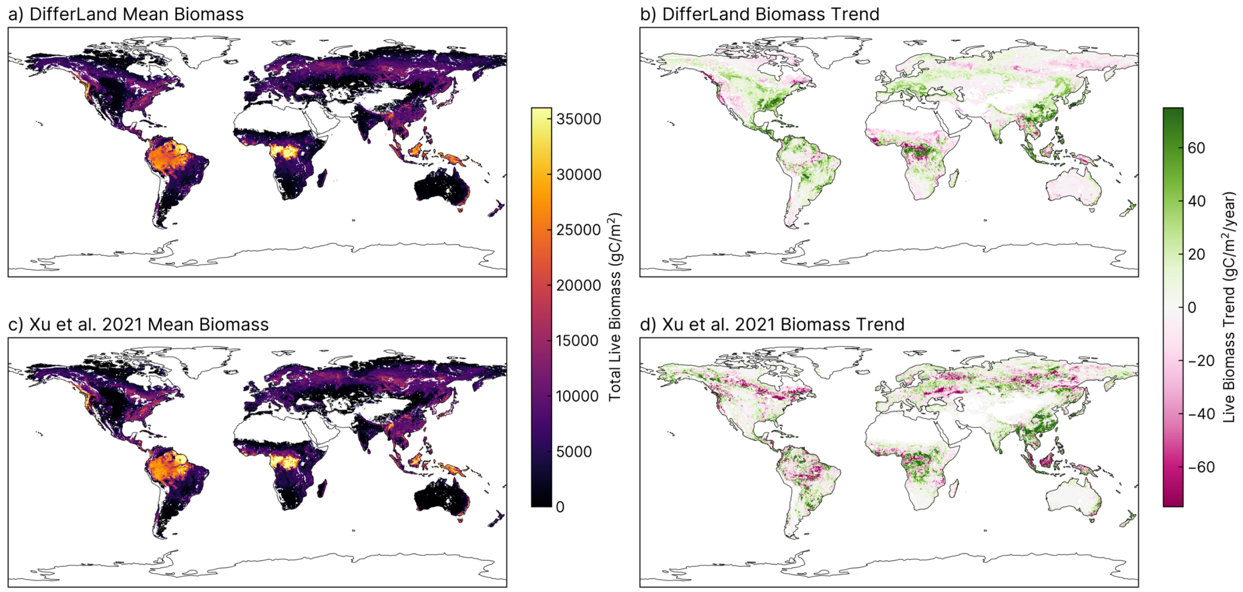

Fig. S23 | Evaluation of DifferLand simulated live biomass against the assimilated Xu et al. 2021[3] biomass. a) DifferLand mean biomass (2003-2023); b) DifferLand Biomass trend; c) Xu et al. 2021 mean biomass; d) Xu et al. 2021 biomass trend.

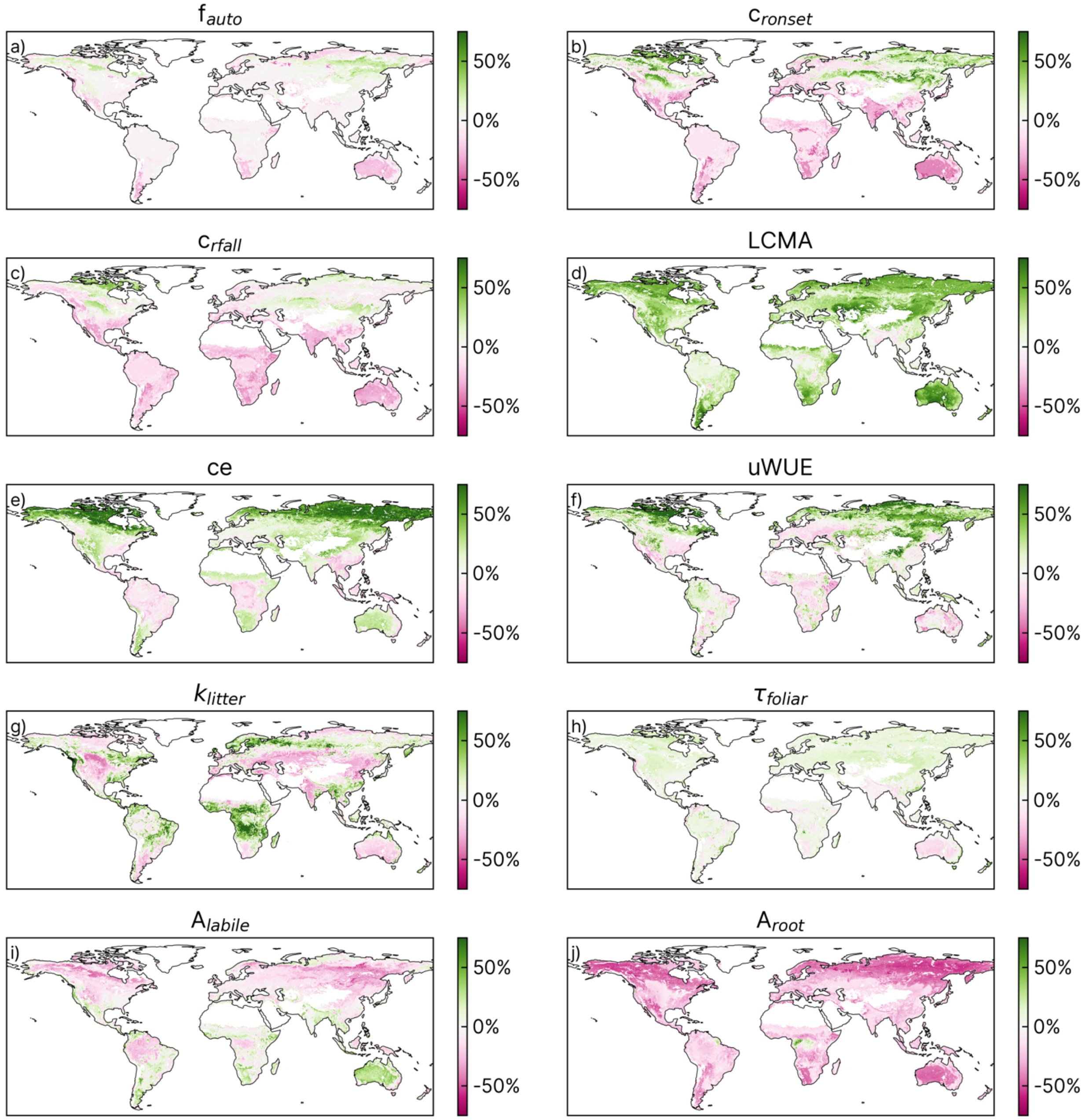

Fig. S24 | Relative parameter difference between the PFT-only and the PFT+CLIM+SOIL+AGE configurations. a) f_{auto}; b) c_{ronset}; c) c_{rfall}; d) LCMA; e) ce; f) uWUE; g) k_{litter}; h) τ_{foliar}; i) A_{labile}; j) A_{root}.

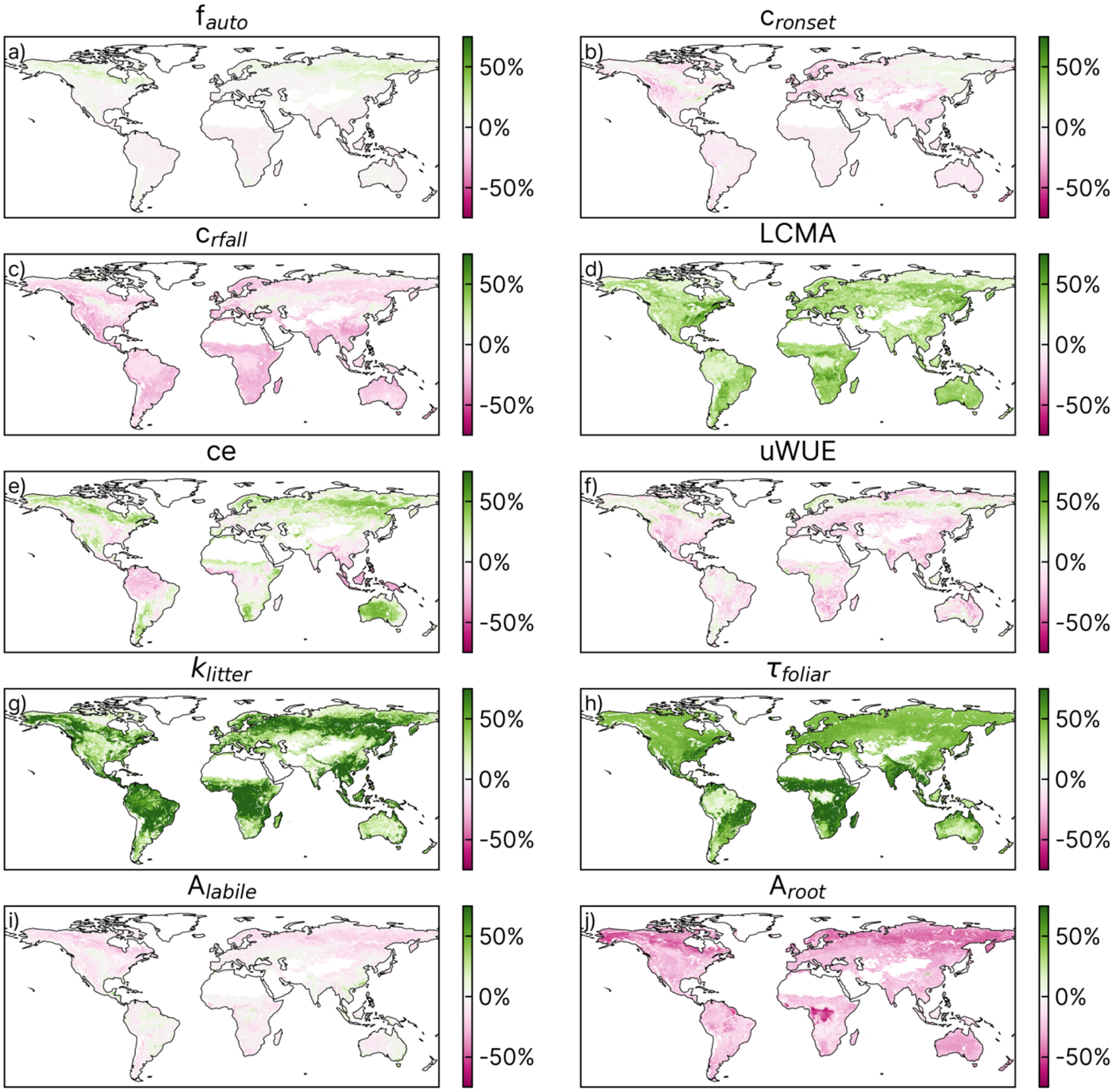

Fig. S25 | Relative parameter difference between the CLIM+SOIL+AGE and the PFT+CLIM+SOIL+AGE configurations. a) f_{auto}; b) c_{ronset}; c) c_{rfall}; d) LCMA; e) ce; f) uWUE; g) k_{litter}; h) τ_{foliar}; i) A_{labile}; j) A_{root}.

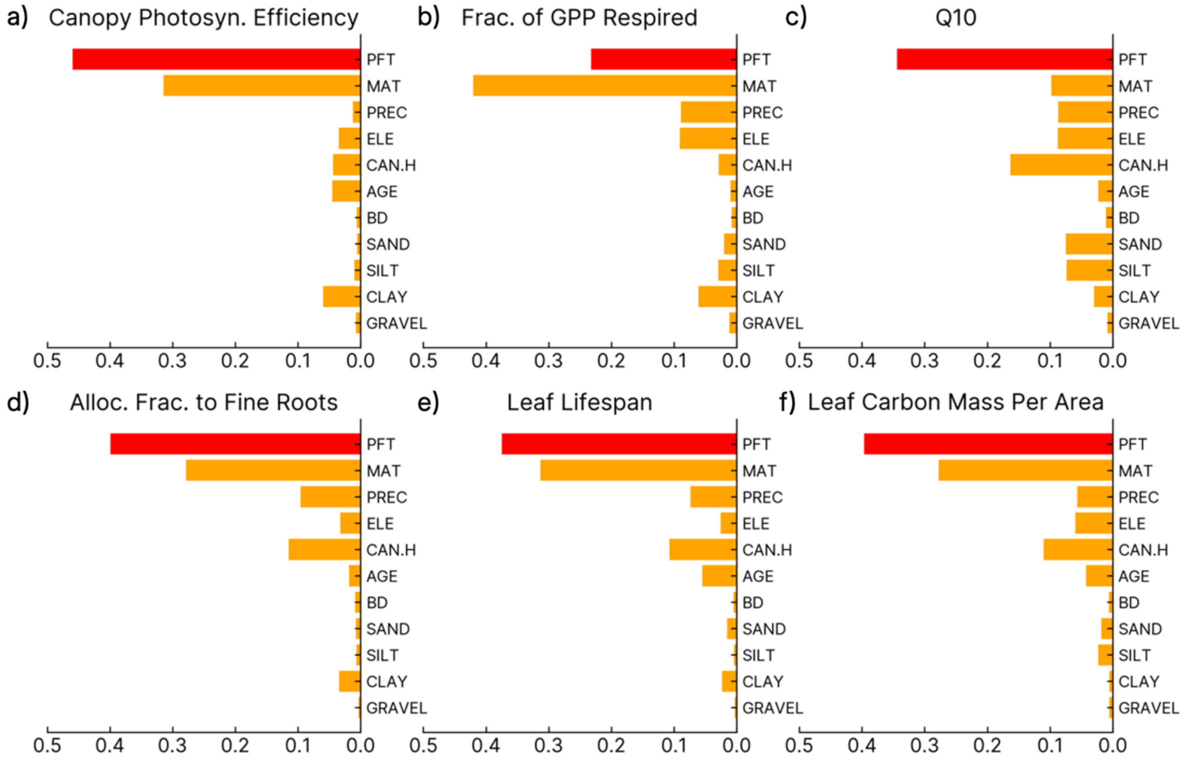

Fig. S26 | Feature contribution to the prediction of several key ecological parameters. Feature contribution is based on the normalized absolute ensemble-averaged SHAP values of 10 ensemble members trained with the full set of PFT+CLIM+SOIL+AGE predictors, with all PFT variables consolidated into a single bar. a) Canopy photosynthetic efficiency; b) fraction of GPP respired; c) Q10; d) allocation fraction to fine roots; e) leaf lifespan; f) leaf carbon mass per area.

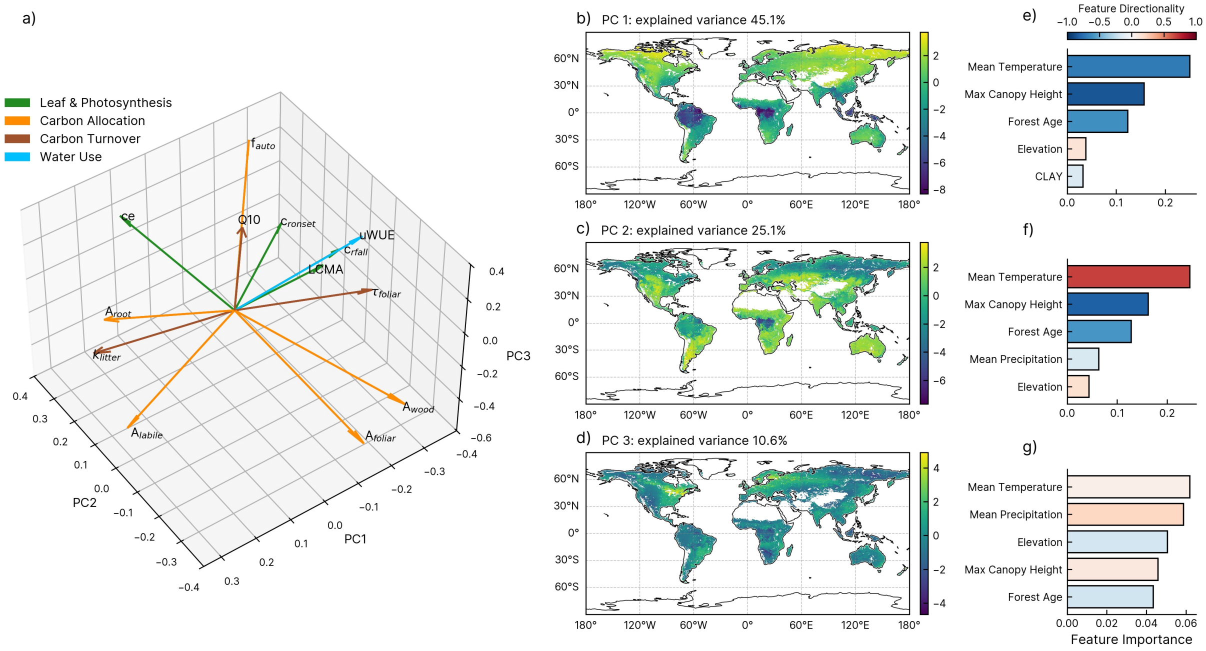

Fig. S27 | Similar to Fig. 3 in the main text, but for the CLIM+SOIL+AGE configuration. a) Principal component analysis (PCA) of 13 latent ecological parameters inferred from the ensemble mean of the CLIM+SOIL+AGE model configuration. b–d**)** Spatial distribution of PC1–PC3 scores projected from the spatialization neural network onto the PCA axes, with the proportion of variance explained by each component labeled. e–g) SHAP-based feature attribution for each principal component, showing the influence of spatial predictors. Note PC1 (b) and PC2 (c) are visually similar to that of the PFT+CLIM+SOIL+AGE configuration, but PC3 (d) does not match the spatial pattern of cropland distribution due the lack of corresponding spatial predictor.

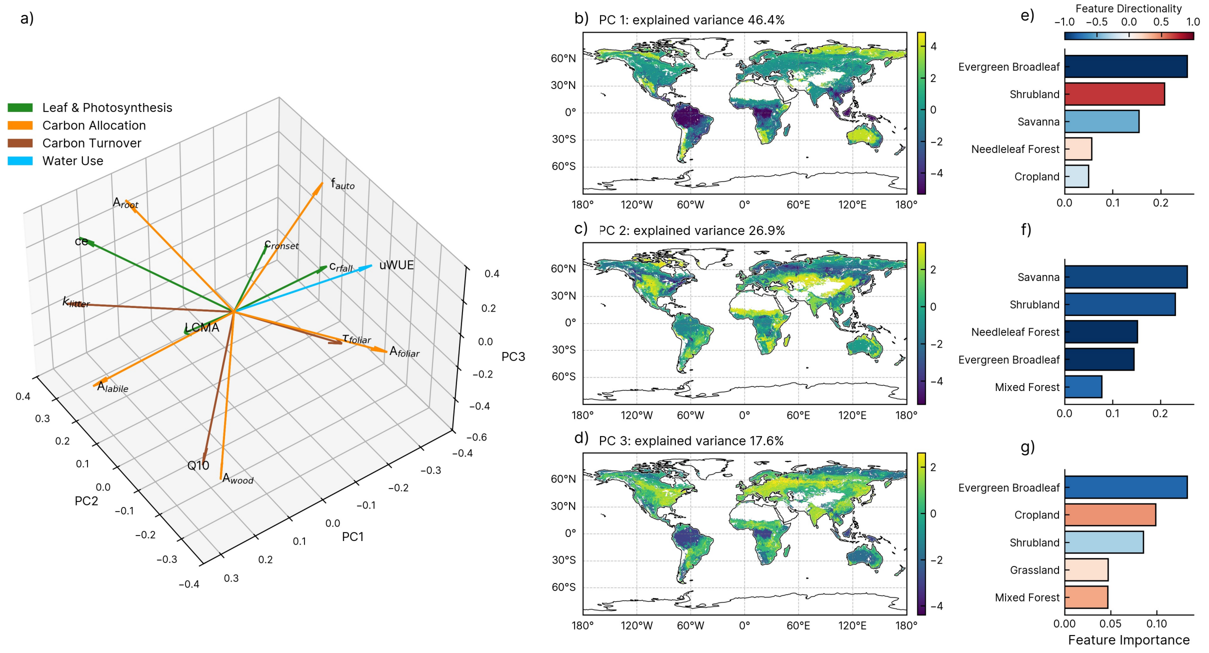

Fig. S28 | Similar to Fig. 3 in the main text, but for the PFT-only configuration. a) Principal component analysis (PCA) of 13 latent ecological parameters inferred from the ensemble mean of the PFT model configuration. b–d**)** Spatial distribution of PC1–PC3 scores projected from the spatialization neural network onto the PCA axes, with the proportion of variance explained by each component labeled. e–g) SHAP-based feature attribution for each principal component, showing the influence of spatial predictors.

Supplementary Notes 1 Equation for transforming the physical model parameters from the real space to their physical range (adapted from Fang & Gentine 2024[4])

$$p_{\mathbb{R}} = \tan\left(\left(\frac{\log\left(\frac{p_p}{p_{min}}\right)}{\log\left(\frac{p_p}{p_{max}}\right)} - 0.5 \right) \times \pi \right)$$

Where $p_{\mathbb{R}} \in \mathbb{R}$ is the parameter transformed into the real space, $p_p \in (p_{min}, p_{max})$ is the parameter in its physical unit, p_{min} is the minimum parameter value in its physical range, and p_{max} is the maximum parameter value in its physical range.

Supplementary Notes 2 A brief description of the DALEC model (adapted from Fang & Gentine 2024[4])

The DALEC model is a dynamical terrestrial biosphere model. The state vector of the model ($\vec{x}_t$) includes the size of the carbon and the water pools at each time step.

$$\vec{x}_t = \left[C_{labile,t}, C_{foliar,t}, C_{wood,t}, C_{root,t}, C_{litter,t}, C_{som,t}, PAW_t, PUW_t\right]$$

The LAI at time t can be computed using $LAI_t = \frac{C_{labile,t}}{LCMA}$, where LCMA is the leaf carbon mass per area.

At each time step, the model computes the non-water limited GPP using the Aggregate Canopy Model[5]

$$GPP_{ACM,\,t} = ACM(LAI_t,\ T_{min,t},\ T_{max,t},\ CO_2,\ DOY_t,\ SSRD_t,\ LAT)$$

A β factor representing soil water limitation is multiplied to compute the soil water limitation effect. $GPP_t = GPP_{ACM,\,t} \times \beta_t$.

Next, a fraction of the GPP_t is used for autotrophic respiration ($R_{a,t}$) based on a learned allocation constant (f_{auto}). The remaining Net Primary Production (NPP), $NPP_t = GPP_t - R_{a,t}$, is allocated between the labile, leaf, wood, and root pools with constant allocation fractions to be learned from data.

The seasonal cycle of leaf development is modelled by the CEDA model[1], which computes the periodic transfer fraction of carbon from the labile to the foliar pool (leaf unset factor Φ_{onset}), and the periodic transfer fraction of carbon from the foliar to the litter pool (Φ_{fall}).

$$\Phi_{onset} = \frac{\sqrt{2}}{\sqrt{\pi}} \times \left(\frac{6.9088}{c_{ronset}}\right) \times e^{-\left(sin\left(\frac{\frac{t - d_{onset} - 0.6245 c_{onset}}{365.25}}{\pi}\right) \cdot \frac{\sqrt{2}\times\pi}{c_{ronset}\times 365.25}\right)^2}$$

$$\Phi_{fall} = \frac{\sqrt{2}}{\sqrt{\pi}} \times \left(\frac{-log(1 - \frac{1}{\tau_{leaf}})}{c_{rfall}}\right) \times e^{-(sin(\frac{\frac{t - d_{fall} + \Psi_f}{365.25}}{\pi}) \cdot \frac{\sqrt{2}\times\pi}{c_{rfall}\times 365.25})^2}$$

The parameters c_{ronset} and c_{rfall} define the duration in which 68% of the leaf onset and leaf fall happen (the slope of the phenology cycle), whereas $d_{onset}\%365.25$ and $d_{fall}\%365.25$ define the DOY on which maximum leaf fall and leaf onset rate occur, and $\frac{1}{\tau_{leaf}}$ corresponds to the average leaf loss fraction within a year. Ψ_f is a numerical term used to ensure that the maximum leaf loss rate occurs on day $d_{fall}\%365.25$ of the year. See Bloom & Williams 2015 for further details.

Litter generations of the wood and root pool are modulated by their respective turnover rates (k_{wood} and k_{root}), and the released carbon is transferred to the litter pool.

For instance, wood $litter_t = C_{wood,t} \times (1 - (1 - k_{wood})^{dt}) / dt$, where dt is the time step of the model. For the daily timestep used in this study, dt=1, so we have wood $litter_t = C_{wood,t} \times k_{wood}$.

Similarly, litter respiration, soil organic matter (SOM) respiration, and litter decomposition into SOM is computed with litter turnover rate (k_{litter}), SOM turnover rate (k_{SOM}) and litter decomposition rate (k_{decomp}). These rates are modified by a multiplicative temperature and precipitation sensitivity term (Ω_t).

$$\Omega_t = e^{\gamma(T_t - \overline{T})} \times ((P_t/\bar{P} - 1) \times \theta_p + 1)$$

Where γ is the temperature sensitivity parameter, T_t is the (monthly) daily mean temperature of time t, $\overline{T}$ is the long-term mean temperature, P_t is the (monthly) precipitation at time t, $\bar{P}$ is the long-term mean precipitation rate, and θ_p is the precipitation sensitivity parameter.

For the PAW water pool, we update the PAW pool at each time step as $PAW_{t+1} = PAW_t + P_t - ET_t - R_t$, where R_t encompasses both surface runoff and the transfer of water into water pools unavailable to plants.

At the end of the time step, the produced and transferred carbon is added to each carbon pool, whereas the released and respired carbon is subtracted from each pool.

Supplementary Notes 3 Dynamical constraints of model trajectory

To ensure the DALEC model remains stable over multi-decadal simulations, we incorporate differentiable dynamical constraints into the loss function. These constraints enforce soft steady-state approximations and discourage unrealistic exponential growth or decay in state variables. Specifically, we assume that the trajectory's mean input-to-output ratio is reasonably close to the theoretical equilibrium ratio at the simulation start, and we add a loss term in the objective function to penalize substantial deviation from this equilibrium. Incorporating such constraints is particularly important for avoiding spurious drifts in simulated NBE early in the run, when NBE observations are unavailable and initial litter and fine root pool sizes are uncertain. Detailed descriptions of dynamical constraints in DALEC models can be found in Bloom & Williams (2015)[1] and its code is adapted from the CARDAMOM_v2.3 release[6].

Supplementary Notes 4 Sensitivity of model results to alternative assimilated datasets

We conducted sensitivity experiments to assess whether incorporating environmental variables consistently improves the simulation of vegetation dynamics compared with the PFT-only baseline, using alternative assimilated datasets. Table S1 lists the datasets used in each experiment. In each setup, the model was trained with an alternative data stream for one target variable—NBE (ΔNBE), LAI (ΔLAI), live biomass (ΔBiomass), VOD (ΔVOD), or fire emissions (ΔFire)—while keeping the datasets for all other target variables identical to the default configuration described in the main text. This design isolates the effect of using alternative data sources for individual target variables.

For NBE, we evaluated an alternative atmospheric inversion from the monthly Copernicus Atmosphere Monitoring Service (CAMS) spanning 2001–2023[7]. In contrast to CMS-Flux, the primary CAMS inversion is constrained by ground-based CO_2 measurements rather than satellite-derived column-integrated CO_2, and it provides continuous coverage over the entire simulation period. The dataset was first conservatively reprojected from its native 1.4° × 0.7° resolution to 0.25° to match the model grid, and then assimilated at the 4° × 5° patch level following the same procedure used for CMS-Flux.

For LAI, we used the Copernicus LAI product, which merges SPOT-VGT[8], PROBA-V, and Sentinel-3/OLCI[9] observations into a continuous monthly record from 2001 to 2023. These datasets have been homogenized by the Copernicus team to ensure temporal consistency. The Copernicus LAI series provides an alternative, MODIS-independent observational stream of canopy structure.

For biomass, we tested the IB-AGC[10], an SMOS L-VOD informed annual live biomass product from 2010-2020 at 25 km resolution, as an alternative to the Xu et al. 2021 biomass product used in the main method. The VOD-based biomass product has potential to provide better temporal patterns and reduce signal saturation in densely forested regions, although its shorter duration and wide gaps due to radio frequency interference (RFI-filtering) also pose additional challenges[11].

In addition, we also optionally assimilated GLAB-VOD[12], a global 18-day L-band equivalent AI-based vegetation optical depth (VOD) dataset at 25 km resolution from 2003-2020 as an additional constraint for aboveground biomass. As the seasonal variations of L-VOD includes information from both canopy structure and water content, we assimilate VOD only at the annual level to minimize the signal of water content[11]. We used a linear observation operator assuming that annual L-VOD can be approximated as a linear combination of the contributions from annual mean foliar biomass and annual mean woody biomass

$$\overline{VOD_{annual}} = p_{wood} \times \overline{C_{wood}} + p_{fol} \times \overline{C_{fol}}$$

Where p_{wood} and p_{fol} are treated as model parameters to be predicted by the spatialization network. This linear observation operator was chosen as opposed to an exponential function form for its parsimony and empirical efficacy in a previous model-data fusion study[13]. Lastly, we also tested assimilating fire carbon emission using the CMS Carbon Flux for Fire[14] inversion between 2010 and 2016, informed by satellite column carbon monoxide concentration measurements from MOPITT, at the 4° × 5° patch level[15].

Table S1 Experiment Setups for Testing Model Sensitivity to Alternative Assimilated Datasets

	NBE	Leaf Area Index	Biomass	VOD	Fire C Emission
**Default**	CMS-Flux GCB 2023 (2010-2023)	BNU Reprocessed LAI (2001-2023)	Xu et al. 2021 (2001-2021 annual)	Not Used	GFED5 Fire Emission (2001-2022, patch level assimilation)
**ΔNBE**	**CAMS NBE (2001-2023)**	--	--	--	--
**ΔLAI**	--	**COPERNICUS LAI (2001-2023)**	--	--	--
**ΔBiomass**	--	--	**IB-AGC (2010-2020, annual)**	--	--
**ΔVOD**	--	--	--	**GLAB-VOD (2003-2020, annual mean)**	--
**ΔFire**	--	--	--	--	**CMS-Flux Fire (2001-2018 monthly 4x5°)**

"--": same as the default configuration

Each experiment was trained under both the PFT-only and PFT+CLIM+SOIL+AGE configurations, with ten independent runs for each setup. We then selected the five runs with the lowest training loss to reduce random uncertainties from model initialization. All variables are assimilated monthly at the 0.25° model grid unless otherwise specified. Model performance on the test dataset was evaluated using the R^2-score on LAI, SIF, NBE, Live Biomass (LBM), ET, and EWT. For a fair comparison, both the default and the alternative models are evaluated on the same set of models used to train the model, thereby avoiding discrepancies in performance metrics arising from differences in the underlying dataset values.

We found that including environmental predictors consistently improves simulated vegetated states and ecological dynamics, independent of the choices of individual alternative datasets (Fig. S29). The robustness likely stems from (1) the overall consistency in the spatial patterns of target variables across different datasets, and (2) the resilience of the multi-stream data assimilation framework to variations in individual target variables (Fig. S4), as the model's structural assumptions and complementary constraints from other variables buffer the impact of any single target variable on overall vegetation dynamics.

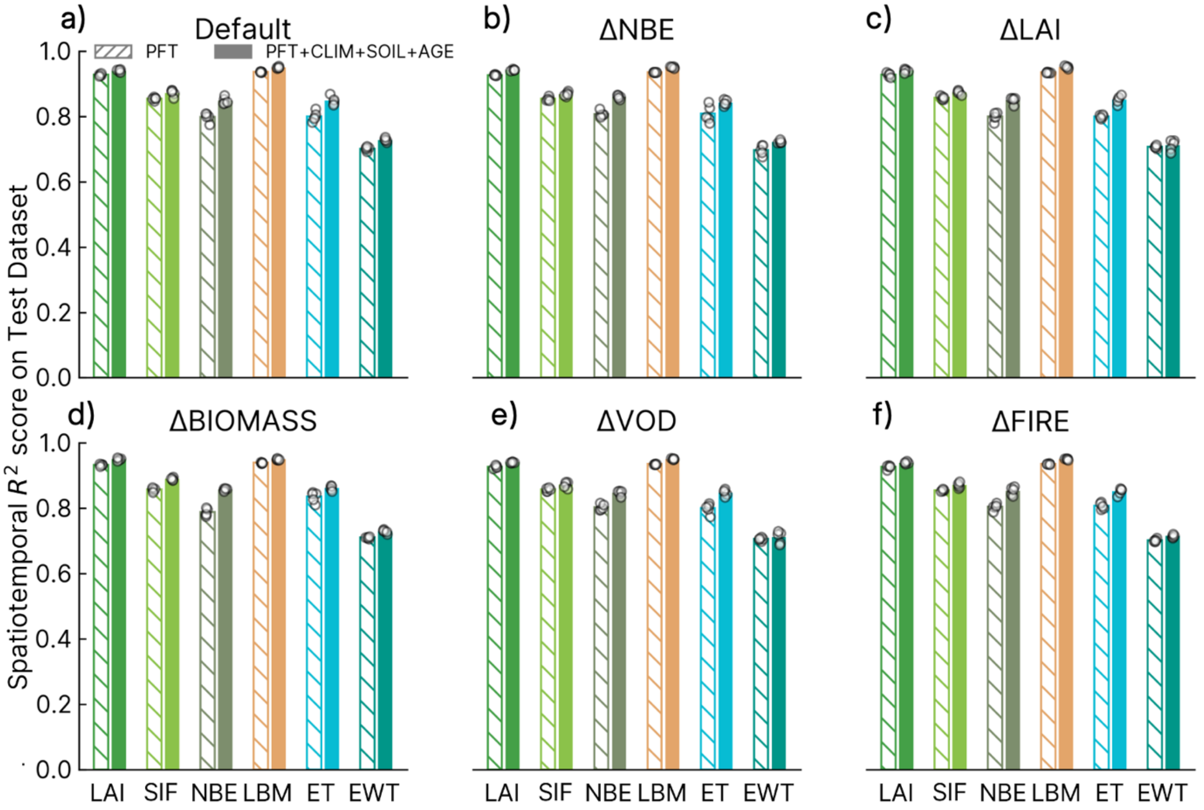

Fig. S29 | Sensitivity analysis of model performance for alternative assimilated datasets. For a) the default dataset usage, and b-f) each sensitivity experiment, we compared the aggregated spatiotemporal R^2-score on held-out test pixels between the PFT-only baseline and the PFT+CLIM+SOIL+AGE configuration. The title on above each panel is indicating the label of each sensitivity experiment setup, as described in Table S1. Each dot represents an individual model member (n=5), and the bars represent the ensemble mean.

Because NBE from atmospheric inversion serves as a critical top-down constraint on the terrestrial carbon cycle—integrating ecosystem carbon fluxes—yet carries larger spatial uncertainties due to limitations in atmospheric transport modeling[16], we further examined how simulated carbon fluxes differ globally and regionally when using CMS-Flux (in the default runs) versus the CAMS inversion. We found that this experiment assimilating the alternative CAMS NBE inversion (corresponding to the "ΔNBE" experiment in Table S1) shows consistent spatial and temporal patterns as the default runs (Fig. S30), although the overall net carbon sink is stronger at -2.79±0.94 PgC/year compared with the -2.09±0.62 PgC/year in the default runs, consistent with the more sinky pattern in CAMS (-2.77 PgC/year) versus CMS (-2.08±0.09 PgC/year). The global annual NBE anomaly correlation between DifferLand-CAMS and CAMS is slightly lower than the correlation between default runs and CMS-Flux (Fig. S31a). Region comparisons suggest CAMS exhibits large interannual excursions in annual NBE trajectories in the South American Tropical (Fig. S31d), South American Temperate (Fig. S31d), North Africa (Fig. S31f), and Tropical Asia (Fig. S31j), potentially due to the paucity of surface CO_2 measurements in those TRANSCOM regions to constrain the carbon dynamics. In contrast, seasonal cycle correlations with the assimilated datasets are consistently strong regardless of the choice of atmospheric inversion, confirming DifferLand's ability to capture seasonal variations in land carbon flux at global and regional levels (Fig. S32).

As CAMS is available prior to 2010, the ΔNBE runs may also serve as a baseline to examine whether the default runs that assimilate NBE only after 2010 are able to reproduce the overall carbon dynamics. Despite differences in mean NBE arising from biases in the respective atmospheric inversions, the default and ΔNBE runs exhibit broadly consistent annual anomalies in global and regional carbon fluxes before 2010 (Fig. S32). This consistency suggests that the multi-stream data assimilation framework can capture carbon cycle dynamics even in historical periods without direct NBE constraints.

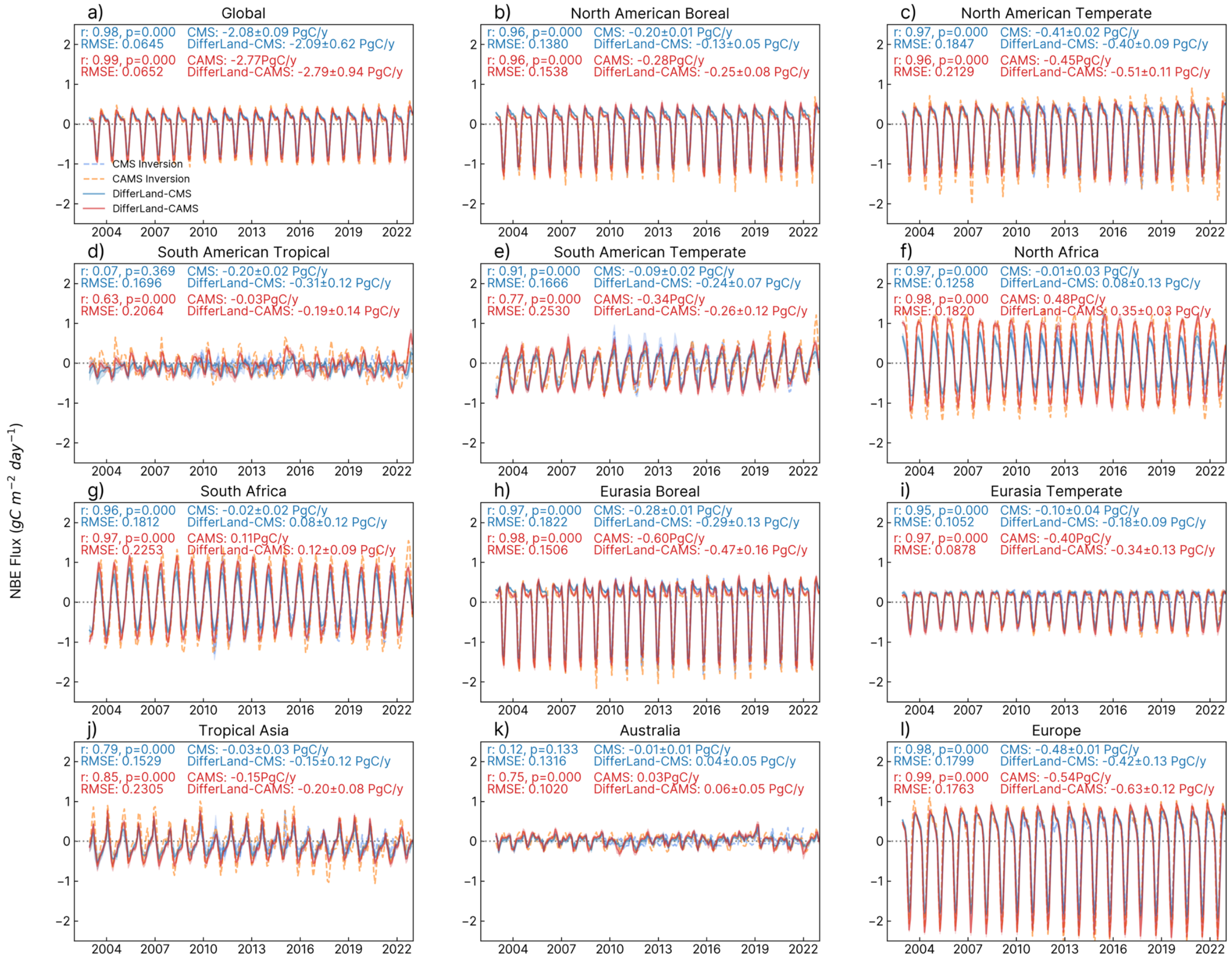

Fig. S30 | DifferLand-simulated net biosphere exchange obtained by assimilating either CMS-Flux NBE or CAMS NBE, compared against the corresponding CMS-Flux and CAMS atmospheric inversion products. We show global and regional NBE time series to evaluate the consistency of DifferLand simulations when constrained by satellite column CO_2 based CMS-Flux versus in situ CO_2 constrained CAMS. CAMS provides a longer temporal coverage from 2001 to 2023, whereas CMS-Flux was only available from 2010 to 2022 at the time of this experiment. Solid lines and the surrounding error bands represent the ensemble mean ± 1 standard deviation of CMS-Flux and CAMS calibrated DifferLand runs (n=5 runs in each case). a) shows the global mean results; b-l) display the results for each of the TRANSCOM region.

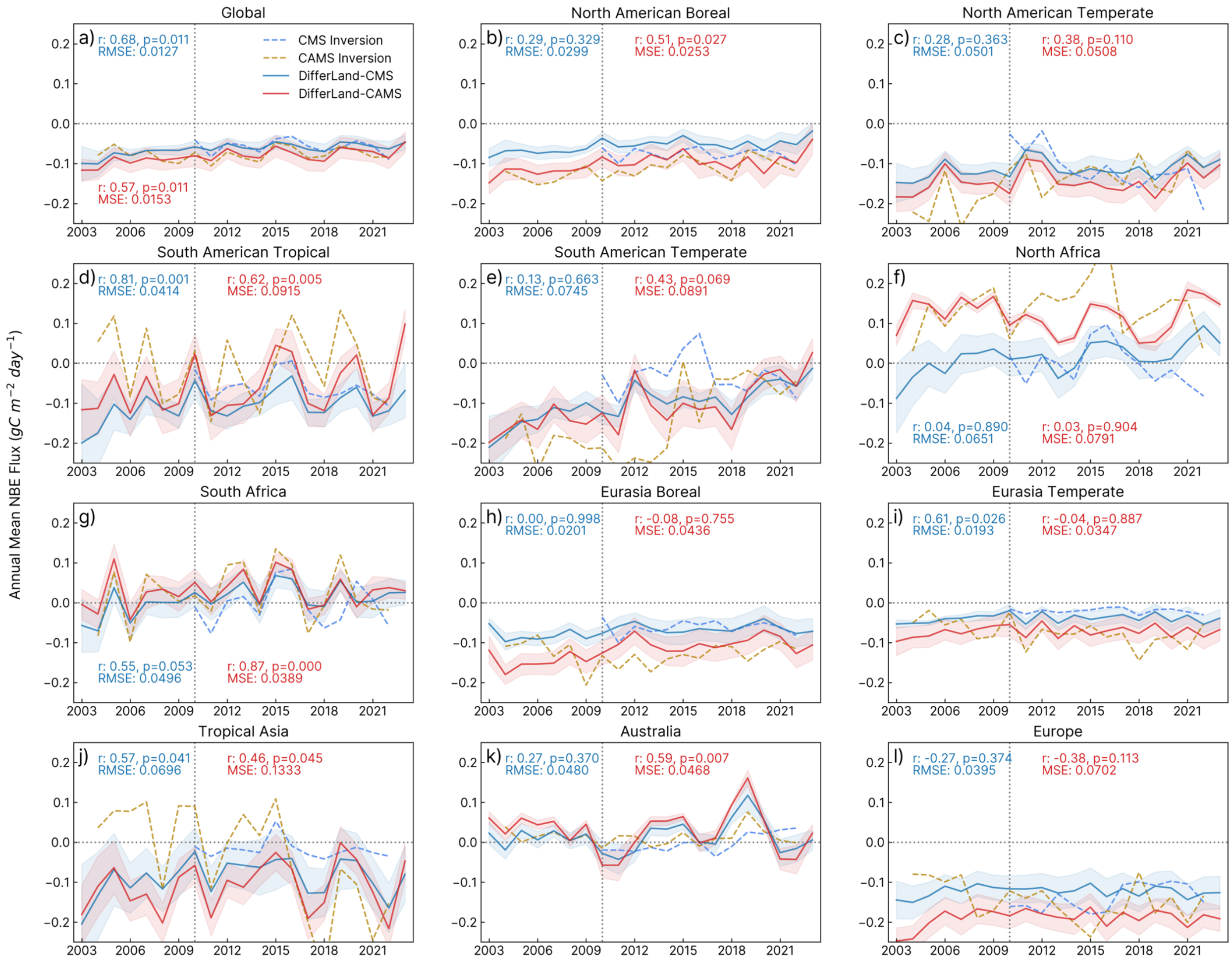

Fig. S31 | Similar to Fig. S30, but comparing only the annual mean NBE. The vertical dotted line indicates period before 2010 when only CAMS NBE inversion was available. Solid lines and the surrounding error bands represent the ensemble mean ± 1 standard deviation of CMS-Flux and CAMS calibrated DifferLand runs (n=5 runs in each case). a) shows the global mean results; b-l) display the results for each of the TRANSCOM region.

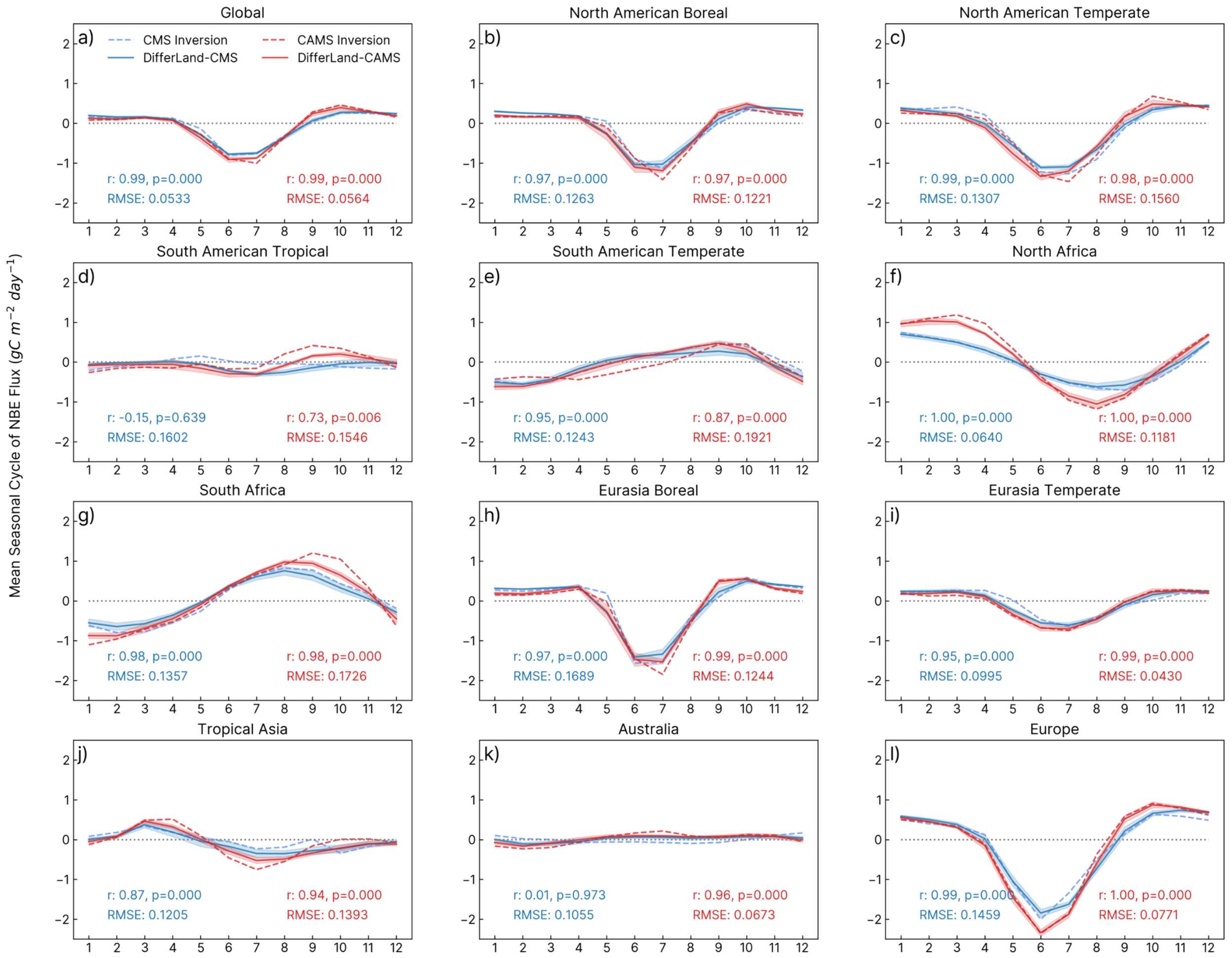

Fig. S32 | Similar to Fig. S30, but comparing the annual mean seasonal cycle. Solid lines and the surrounding error bands represent the ensemble mean ± 1 standard deviation of CMS-Flux and CAMS calibrated DifferLand runs (n=5 runs in each case). a) shows the global mean results; b-l) display the results for each of the TRANSCOM region.

Using the latent parameters inferred from the sensitivity experiments that assimilated alternative observational constraints, including the ΔNBE configuration using CAMS-NBE instead of CMS-Flux (Fig. S33), the ΔLAI configuration using Copernicus LAI instead of MODIS LAI (Fig. S34), and the ΔEC (Fig. S35) configuration excluding eddy-covariance flux data, we evaluated how these choices affect the emergent structure of the parameter space. For each sensitivity configuration, we applied the same principal component analysis as in Fig. 3 to the selected latent parameters, using identical preprocessing, standardization, and spatial masking. While the relative ordination of individual parameters in the principal component space exhibits minor variations across configurations, the large-scale spatial patterns associated with each principal component and the fraction of variance they explain are highly consistent with the baseline case. This indicates that the dominant modes of covariation among latent parameters are robust to the choice of assimilated data streams and are not driven by any single observational constraint.

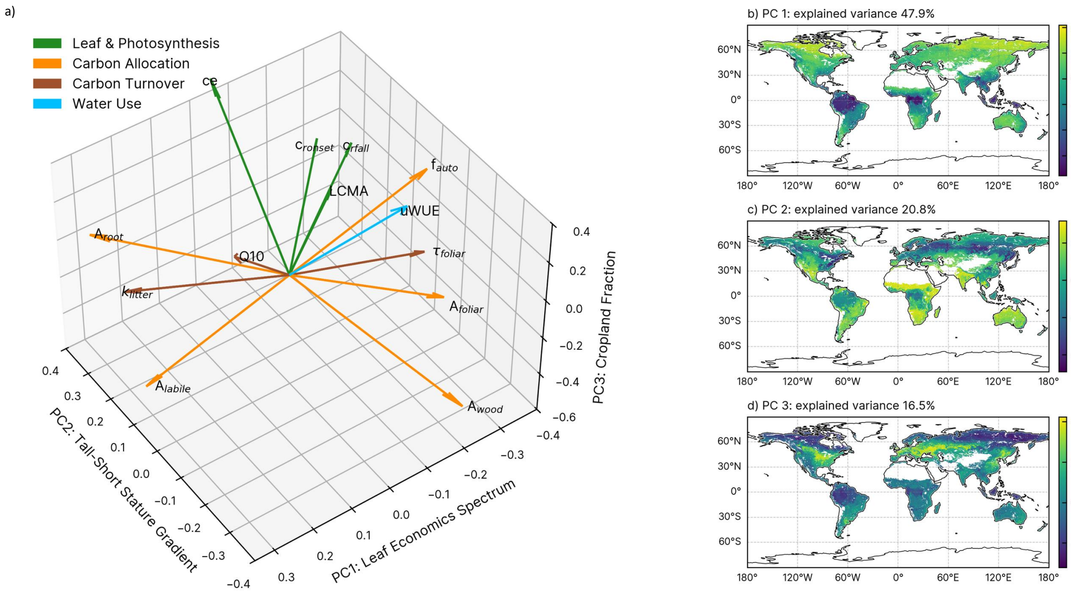

Fig. S33 | Principal Component Analysis of the latent parameters under the ΔNBE experiment, with the same notations as Fig. 3 in the main text. a) shows the loadings that project each parameter onto the first three PCs are plotted in the 3D plot. b-d) shows the spatial distribution of PC1–PC3 scores projected from the spatialization neural network onto the PCA axes, with the proportion of variance explained by each component labeled.

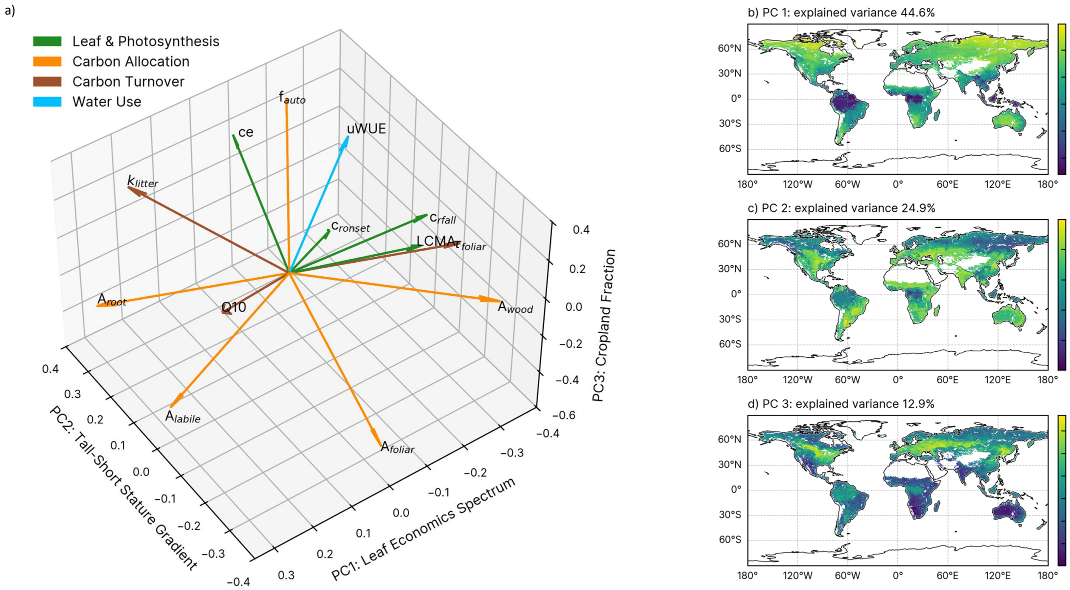

Fig. S34 | Same as Fig. S33 but for the ΔLAI configuration. a) shows the loadings that project each parameter onto the first three PCs are plotted in the 3D plot. b-d) shows the spatial distribution of PC1–

PC3 scores projected from the spatialization neural network onto the PCA axes, with the proportion of variance explained by each component labeled.

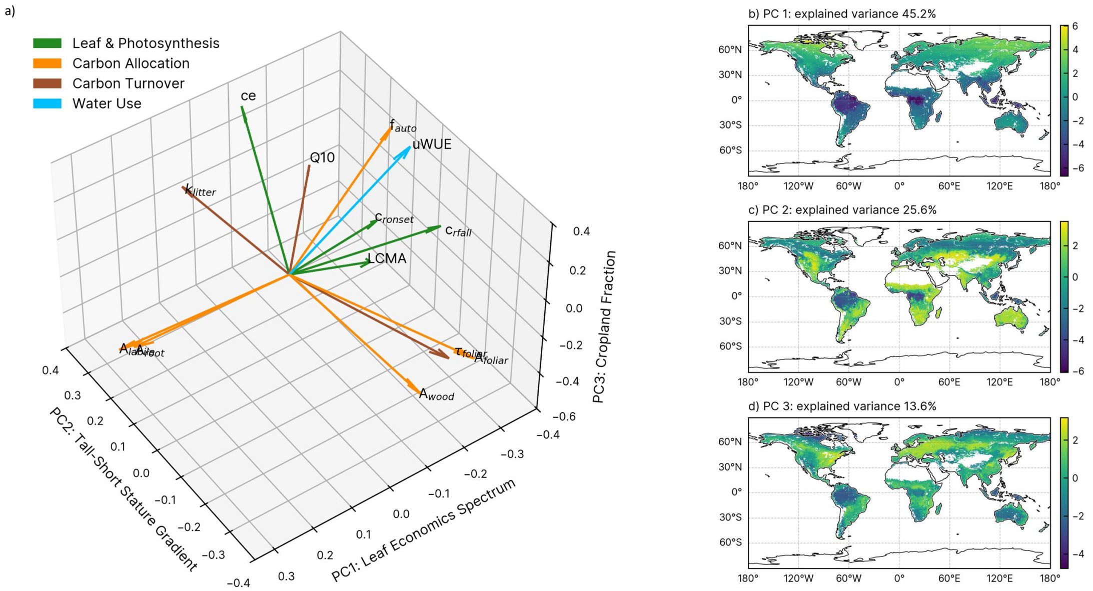

Fig. S35 | Same as Fig. S33 but for the ΔEC configuration. a) shows the loadings that project each parameter onto the first three PCs are plotted in the 3D plot. b-d) shows the spatial distribution of PC1–PC3 scores projected from the spatialization neural network onto the PCA axes, with the proportion of variance explained by each component labeled.

Supplementary Notes 5 Sensitivity analysis of different filtering criteria for spatial mismatches between assimilated eddy covariance datasets and model grid cells.

A source of uncertainty in this study arises from the scale mismatch between the eddy covariance (EC) tower footprint[17]—where turbulent fluxes of land–atmosphere exchange are measured, typically spanning ~100 m to several kilometers depending on wind direction and tower height—and the much larger 0.25° model grid cell (~25 km). To reduce this mismatch, we compute grid-cell mean fluxes by averaging across multiple EC sites within a cell whenever possible and exclude towers that represent atypical managed habitats (e.g., AU-Lox, an almond orchard in South Australia's Riverland). However, many grid cells contain only a single operating site, if any. To ensure minimal consistency between site vegetation and the containing grid cell, we require a threshold fraction of plant functional type (PFT) coverage for a site to be included. Higher thresholds improve representativeness but reduce site availability, whereas lower thresholds increase the risk of spatial mismatch. As a compromise, we tested four filtering schemes: a minimal coverage of 10% (ΔEC10, n=218 sites), 25% (default, n=180), and 50% (ΔEC50, n=123), along with a control case excluding all EC data (ΔEC). The spatial distribution of the included sites and the number of sites per plant functional type under each threshold are shown in Fig. S36.

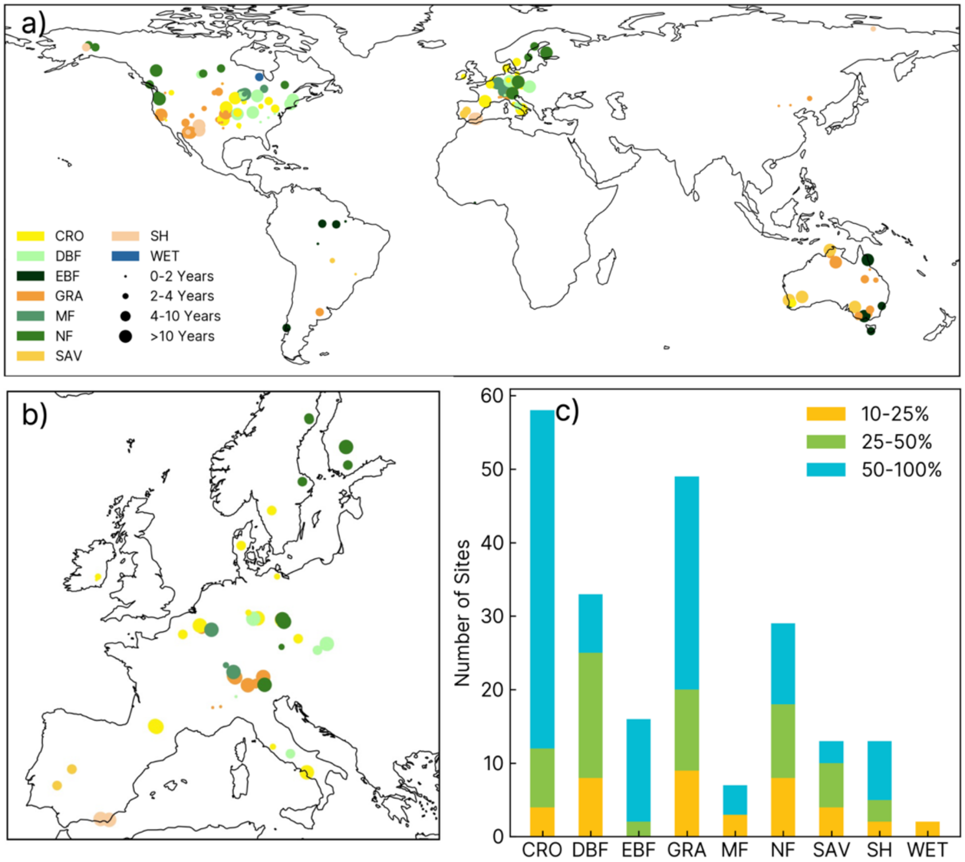

Fig. S36 | a) Spatial distribution of eddy covariance sites used in this study. Marker size indicates the length of available GPP observations at each site. b) Zoomed-in view of sites located in Europe. c) Distribution of sites by vegetation type. Colors indicate spatial homogeneity, defined as the fraction of the 0.25° grid cell that shares the same vegetation type as reported at the site level, categorized into three classes.

We found that changing the filtering thresholds or even leaving out eddy covariance data does not alter our main conclusion: adding spatial predictors improves the model's ability to predict ecological variables (Fig. S37). These results suggest the general spatial gradient of the environmental variables is mostly constrained by remote sensing and atmospheric inversion variables, rather than the sparse eddy covariance data.

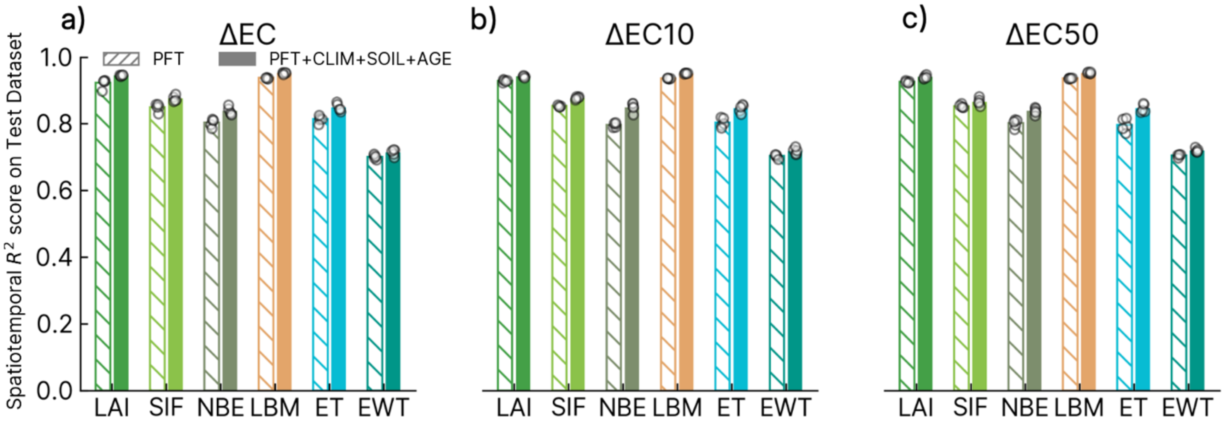

Fig. S37 | Sensitivity of the PFT and PFT+CLIM+SOIL+AGE configurations to different filtering thresholds for the spatial representativeness of eddy covariance data. a) ΔEC; b) ΔEC10; c) ΔEC50. Each dot represents an individual model member (n=5), and the bars represent the ensemble mean.

Eddy covariance data play a critical role in constraining the absolute magnitudes of ecosystem gross carbon uptake (GPP) and respiration (RECO) (Fig. S38). Without assimilation of eddy covariance observations (ΔEC), the DifferLand ensemble simulates a global GPP of 91.9 ± 27.8 PgC yr^{-1} and a global RECO of 88.2 ± 27.8 PgC yr^{-1} during 2003–2023. By contrast, under the default configuration with eddy covariance data assimilated, the model produces a global GPP of 136.7 ± 13.8 PgC yr^{-1} and RECO of 133.0 ± 13.6 PgC yr^{-1}. These values are consistent with independent benchmarks, including machine-learning–based upscaling of site-level GPP (e.g., FLUXCOM X-BASE, 125 ± 2.1 PgC yr^{-1}) and SIF-based global GPP estimates (135.5 ± 8.8 PgC yr^{-1})[18].

The largest discrepancies between ΔEC and the default runs occur in equatorial regions: with assimilation, GPP and RECO reach ~7 gC m^{-2} day^{-1}, compared to only ~4 gC m^{-2} day^{-1} without assimilation. Site-level evaluations in tropical forests confirm that omitting eddy covariance constraints leads to systematic underestimation of both fluxes. This outcome is expected because tropical forests typically exhibit near-zero net carbon balance and weak seasonal cycle (e.g., Fig. S30d) despite high rates of photosynthesis and respiration; without point-based flux measurements, the model has little information to constrain the true magnitudes of these component processes. We note that differences among eddy covariance assimilation setups are relatively small for ET (Fig. S38), as its latitudinal gradient is already partially constrained by the assimilated GLEAM ET. Nonetheless, model runs assimilating site-level ET generally simulate lower values than those relying solely on gridded products.

Varying the filtering thresholds for site spatial representativeness had only a minor effect on simulated GPP and RECO, with global GPP estimates of 135.7 ± 8.2 PgC yr^{-1} under strict filtering (ΔEC50) and 139.6 ± 6.7 PgC yr^{-1} under more relaxed criteria (ΔEC10). This suggests that spatial mismatches between site footprints and model grid cells exert only a limited influence on global estimates. Nonetheless, future work should pursue more effective multiscale data-assimilation strategies and evaluate whether the inferred environment–trait relationships are consistent across canopy, ecosystem, and biome scales.

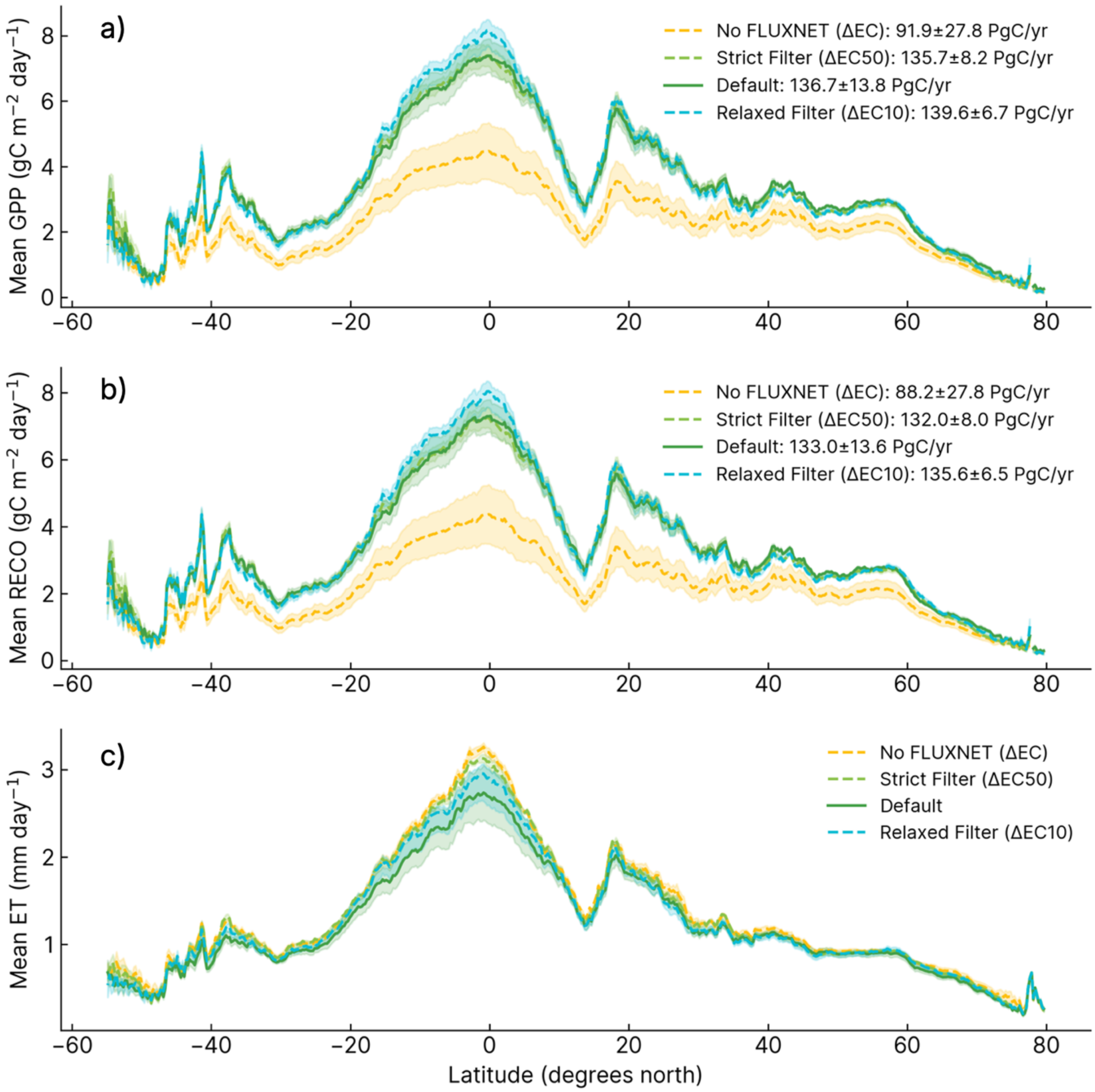

Fig. S38 | Latitudinal transects of mean GPP (top panel), RECO (middle panel), and ET (bottom panel) from model runs under four eddy covariance (EC) data assimilation configurations: no assimilation (ΔEC), a strict filtering criterion in which only sites whose reported PFT covers >50% of the overlapping grid cell are assimilated (ΔEC50, n=123 sites), the default setup in which sites with >25% coverage are assimilated (n=180 sites), and a relaxed criterion in which all sites with >10% coverage are assimilated (ΔEC10, n=218 sites). The lines and error bands represent the ensemble mean ± 1 standard error (n=5 runs) of zonally averaged fluxes at each latitude. Mean annual totals of GPP and RECO for each configuration are computed and labeled in the plots.

Supplementary Notes 6 Model parameter identifiability analysis results and comparison with independent studies

We examined the spatial patterns of the model-estimated ecological parameters. Given that our model includes 40 parameters with varying sensitivities to the assimilated datasets, we conducted a parameter identifiability analysis to select parameters exhibiting robust patterns across independently initialized ensemble members (Fig. S39, Fig. S40, Fig. S41). We find that parameters controlling ecological processes on seasonal or shorter time scales, such as phenology (the timing of leaf onset and senescence), leaf carbon mass per area (LCMA), canopy photosynthetic efficiency, and evapotranspiration (uWUE and r_b) can be robustly constrained by the assimilated datasets. Additionally, the model ensembles learn consistent patterns of carbon use efficiency (CUE), the allocation fractions to labile, root, and wood pools, the sensitivity of heterotrophic respiration to temperature (Q10) and precipitation (θ_p), and the residence time (the inverse of turnover rate, Fig. S43) of the labile (τ_{labile}), foliar (τ_{foliar}), and litter pools (τ_{litter}).

In contrast to the good performance of short-term processes, parameters related to processes on multiannual to multi-decadal timescales, such as wood turnover (τ_{wood}), litter to soil organic matter decomposition rate (k_{decomp}) and SOM turnover (τ_{SOM}), are subject to larger uncertainties. The ensemble means and standard deviations of residence time (Fig. S43) and allocation fractions (Fig. S44) are broadly consistent with previous pixel-level model-data fusion studies at coarser resolution[19], but exhibit smoother patterns with reduced local noise, due to the use of a global spatialization network and spatially continuous predictors.

We evaluated the retrieved ecological parameters against independent observational datasets and synthesis products wherever such constraints are available, focusing on both absolute magnitudes and large-scale spatial patterns. For carbon use efficiency, DifferLand retrieves a global mean value of 0.36 ± 0.03 with spatial variation ranging from 0.26 to 0.69. This is broadly consistent with site-based syntheses reporting a mean of 0.43 ± 0.12 across 2,737 eddy-covariance site-years[20] and a global synthesis of more than 200 site-level studies reporting a mean of 0.46 and a range of 0.22 to 0.79[21]. The retrieved CUE also exhibits a systematic decrease from boreal and temperate ecosystems toward the tropics, consistent with both site measurements[21] and satellite-derived MODIS NPP/GPP products[22], which show lower carbon use efficiency in warm and wet environments.

For leaf carbon mass per area (LCMA), DifferLand retrieves high values in wet tropical and semi-arid regions and lower values in temperate forests, broadly consistent with global LCMA maps derived from statistical upscaling of trait databases[19,23] (Fig. S45). The main deviation in absolute values occurs in tropical forests, where DifferLand retrieves higher LCMA values than suggested by existing upscaled products. However, observational constraints on LCMA in tropical forests remain sparse, and tropical ecosystems exhibit exceptionally large functional diversity in leaf traits, which limits the robustness of current benchmarks and complicates direct comparison. As a result, this discrepancy likely reflects a combination of weak observational constraint and genuine ecological heterogeneity. Additional trait measurements and improved representation of tropical functional diversity will be needed to more robustly evaluate and constrain LCMA in these regions.

We further evaluated additional parameters introduced in the revised manuscript. The retrieved temperature sensitivity of heterotrophic respiration (Q10) was compared with Q10 values inferred from soil organic carbon stocks using the Carnegie–Ames–Stanford Approach under equilibrium assumptions (Fig. S46). DifferLand retrieves biome-scale Q10 values that are consistently lower than those inferred by Zhou et al. (2009)[24], but show a similar ordering across biomes, with higher Q10 in colder ecosystems

and lower Q10 in warmer ones. We note that substantial differences in model structure, assimilated datasets, and modeling assumptions may introduce biases that limit comparability.

For belowground allocation, we compared the retrieved fine-root-to-total biomass fraction with the one of the few globally available benchmarks, which reports total root (fine plus coarse) to total biomass[25]. Despite the structural mismatch between these quantities, both the retrieved and benchmark datasets show a systematic decline in root allocation with increasing precipitation. When stratified by precipitation quantiles in log space, both datasets exhibit decreasing median root allocation from arid to humid regimes, although the retrieved fine-root fraction is consistently lower in magnitude, as expected given that coarse roots are treated as part of woody biomass in DifferLand (Fig. S47). This agreement in gradient, despite differences in definition and scale, indicates that DifferLand captures the large-scale climatic control on belowground investment.

Taken together, these comparisons demonstrate that while direct pointwise or absolute validation is constrained by differences in variable definitions, spatial and temporal scales, and observational availability, the inferred parameters are quantitatively plausible and consistently reproduce large-scale gradients across independent datasets. Although the comparison underscores the limitations of data in certain biomes like tropical forests, we acknowledge that DifferLand has the capacity to readily incorporate additional observations or incorporate observation-informed priors of ecological parameters. This potential allows DifferLand to further enhance the robustness of the retrieved ecological parameters and process dynamics as new observations become available.

We also compared the trait–environment relationships retrieved by DifferLand (Fig. 4) with relationships inferred from independent globally upscaled datasets (Fig. S48), including V_{cmax25} from Luo et al. (2021)[26], carbon use efficiency from Luo et al. (2025)[20], and the belowground-to-total live biomass fraction from Huang et al. (2021)[25]. Across plant functional types and variables, the dominant spatial gradients are broadly similar between the retrieved relationships and those inferred from these independent products. Photosynthetic capacity proxies and carbon use efficiency both tend to decline with increasing precipitation, with grasslands and shrublands exhibiting higher values than forests and tropical forests exhibiting lower carbon use efficiency than cooler or drier biomes. Carbon use efficiency also shows a clear decline with increasing mean annual temperature, consistent with the patterned captured by the spatialization network. The belowground-to-total biomass fraction likewise decreases in wetter environments, indicating a shift toward aboveground investment under reduced water limitation. These shared patterns indicate that the spatialization network in DifferLand retrieves large-scale gradients that are consistent with those emerging from independent observational syntheses.

At the same time, the strength and shape of the relationships differ in some cases. In particular, the observational products exhibit weaker sensitivities to temperature and canopy height than the retrieved DifferLand parameters, especially for V_{cmax25}. This likely reflects differences in how the quantities are defined and estimated, as the DifferLand parameters represent effective canopy-scale quantities within an aggregated model framework, whereas the observational datasets are constructed by extrapolating site-level measurements using a limited set of predictors that often do not include canopy height. In addition, the observational root allocation product integrates over both fine and coarse roots, whereas DifferLand explicitly represents fine-root allocation. These differences suggest that the comparisons are most informative at the level of large-scale gradients rather than absolute magnitudes or local sensitivities.

Supplementary Notes 7 Structural limitations in the current DALEC framework

Despite strong overall performance, the hybrid DALEC framework shows reduced skill for water cycle variables (spatiotemporal R^2 = 0.68 for ET and 0.45 for EWT on test pixels). This likely reflects the simplified formulation of evapotranspiration based on fixed water-use efficiency, which cannot capture dynamic stomatal regulation under variable meteorological conditions. Additional mismatches arise because GRACE-derived equivalent water thickness integrates deep groundwater and large-scale hydrological processes that are absent from the DALEC soil-bucket scheme, and because vertical soil moisture transport and groundwater dynamics are not explicitly modeled. Potential improvements include more mechanistic stomatal and transpiration formulations (potentially supported by hybrid-ML approaches), multi-layer soil hydrology distinguishing plant-available versus unavailable water pools, and watershed-scale constraints such as streamflow to better anchor modeled water balance.

The phenology module is even more restrictive, as DALEC prescribes fixed dates and durations for leaf onset and senescence. This simple structure prevents the model from capturing interannual variability in leaf dynamics driven by atmospheric forcings such as temperature, soil moisture, vapor pressure deficit, or photoperiod. As a result, while the model well reproduced the mean seasonal cycle of LAI, mismatches in the interannual variations of simulated and target LAI are evident, particularly in ecosystems where phenology is strongly climate-sensitive. Future improvements should incorporate more flexible phenology formulations that couple mechanistic environmental cues with data-driven parameterizations and explicitly represent leaf turnover and age structure.

In addition to these discussed errors, misspecification and process simplification in other components of the mechanistic model, including photosynthesis, respiration, carbon turnover, and belowground carbon, can also bias the inferred parameter values and their apparent environmental dependencies. These structural errors can be partially absorbed by the inferred parameters and the result in biased environment-parameter relationships. Together, these limitations underscore priority areas for future hybrid land model development, highlighting the need for more mechanistic yet flexible formulations to improve predictions of ecosystem responses under a changing climate.

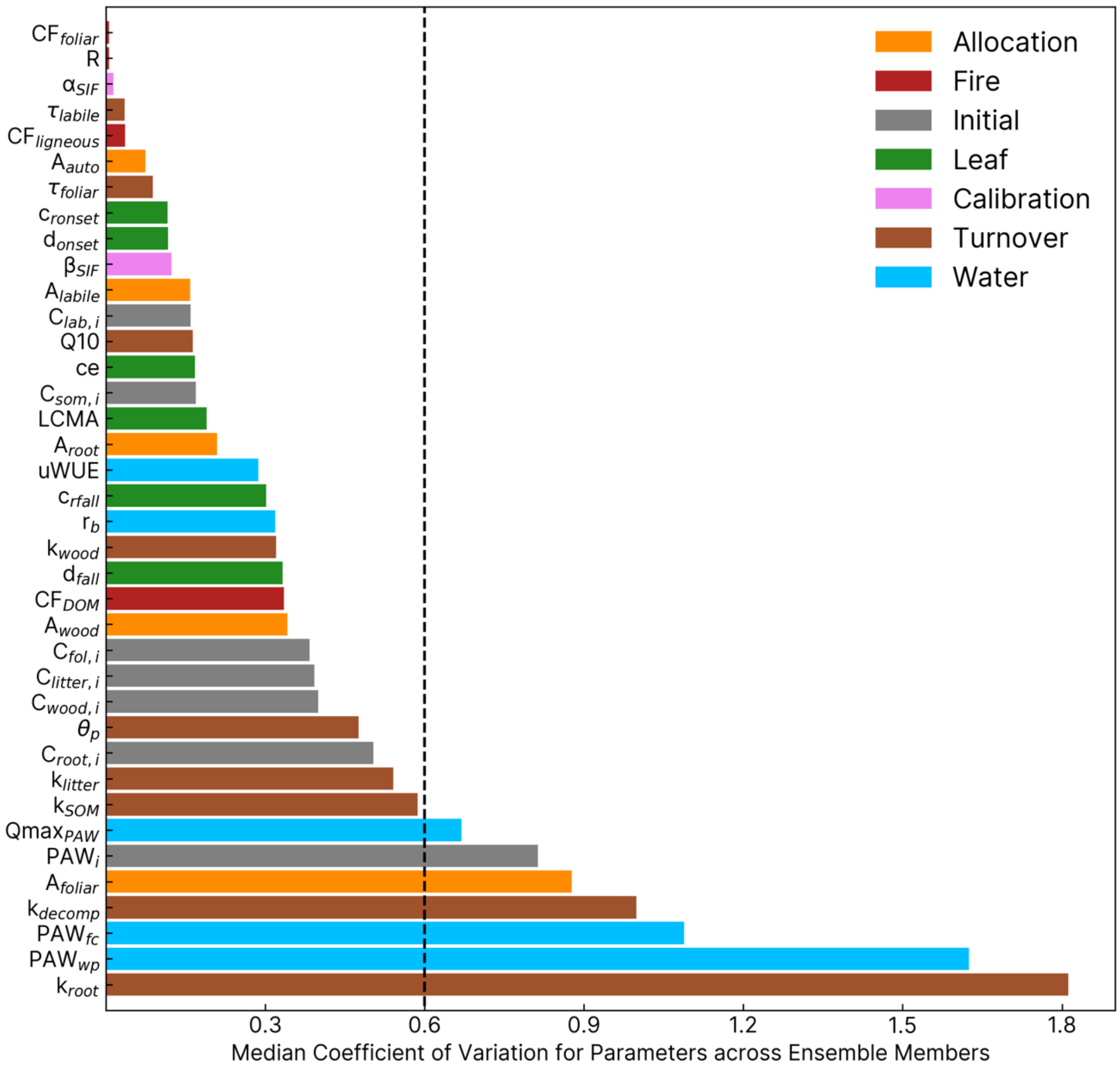

Fig. S39 | The spatial median coefficient of variation of parameters across n=10 of 20 ensembles of the PFT+CLIM+SOIL+AGE configuration that achieved the best training loss. The parameters are ranked with ascending median coefficient of variation. We used a median coefficient of variation of 0.6 as a threshold for identifiable latent model parameters. The parameters are color coded to represent their role in the DALEC model (see legend).

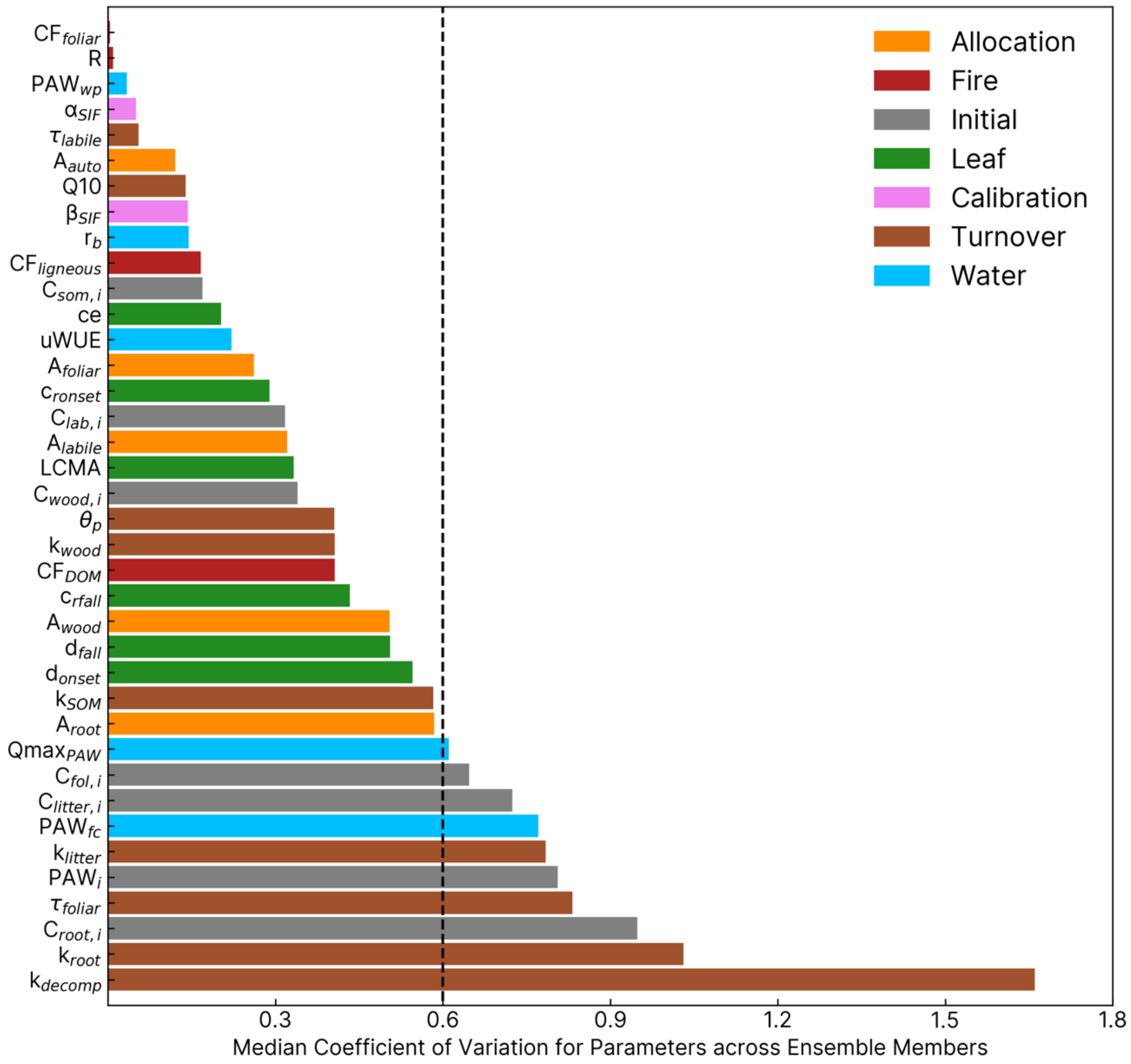

Fig. S40 | The spatial median coefficient of variation of parameters across n=10 out of 20 ensembles of the CLIM+SOIL+AGE configuration that achieved the best training loss.

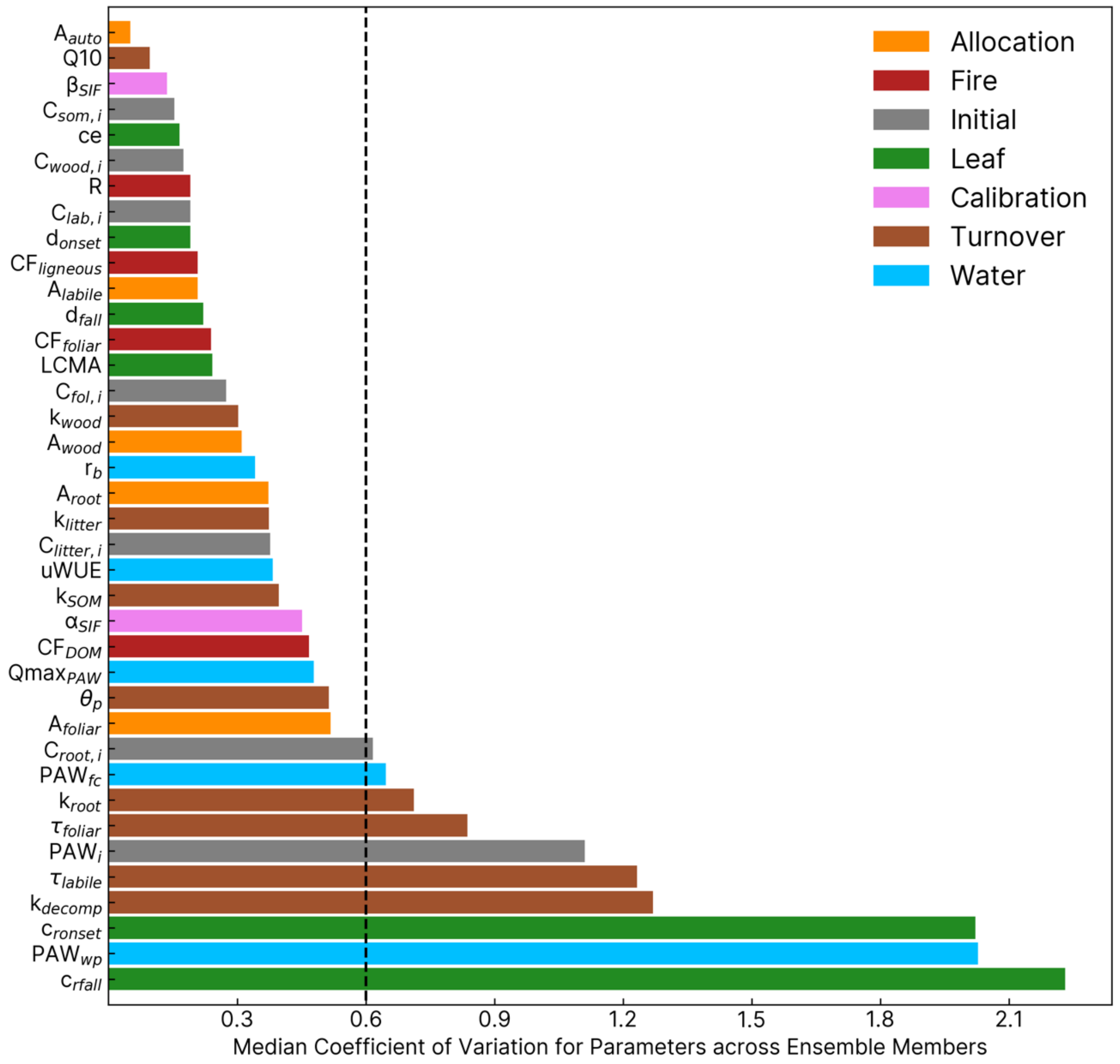

Fig. S41 | The spatial median coefficient of variation of parameters across n=10 out of 20 ensembles of the PFT configuration that achieved the best training loss.

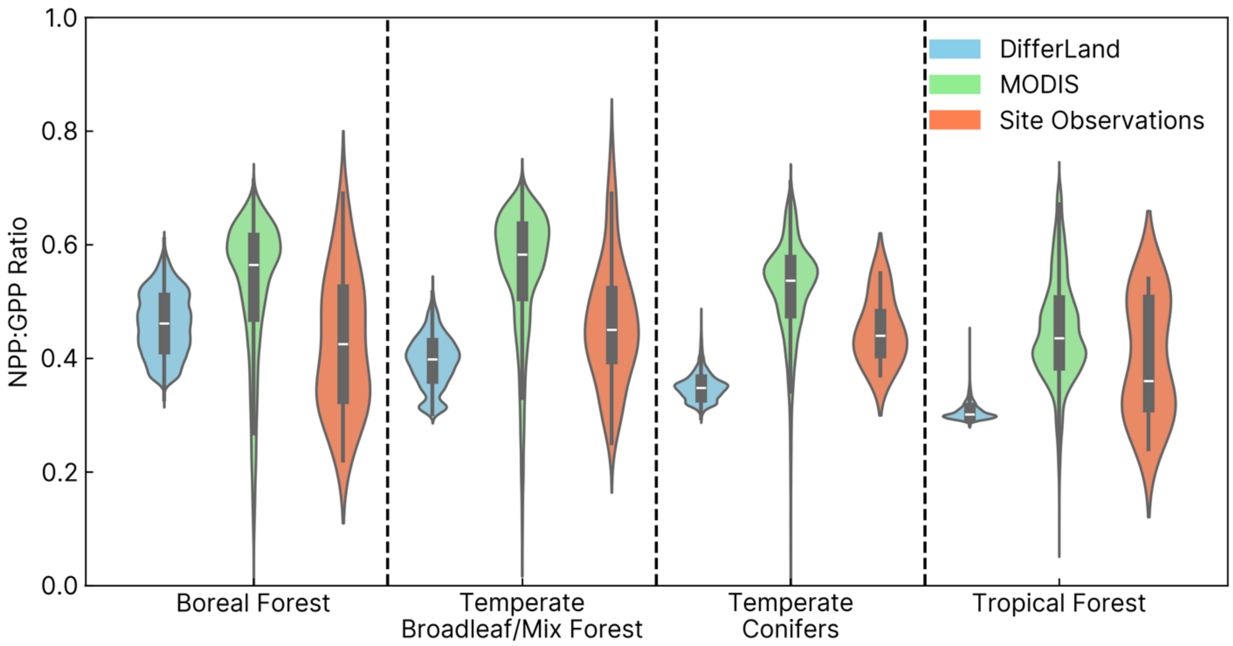

Fig. S42 | Comparing NPP:GPP ratio retrieved by DifferLand against in situ measurements and MODIS-based NPP and GPP products. For DifferLand, we first computed the ensemble mean NPP:GPP ratio across n=10 out of the 20 ensembles of the PFT+CLIM+SOIL+AGE configuration that achieved the lowest training loss, and then derived the per-biome spatial distribution using WWF biome maps[27]. For MODIS, we derived the mean NPP and GPP values from MOD17A3HGF.061 between 2010 and 2023, and computed the per-biome distribution. For site observations, the results are obtained from Collati & Prentice 2019 by aggregating site-level studies based on the biome classification presented in the paper[21]

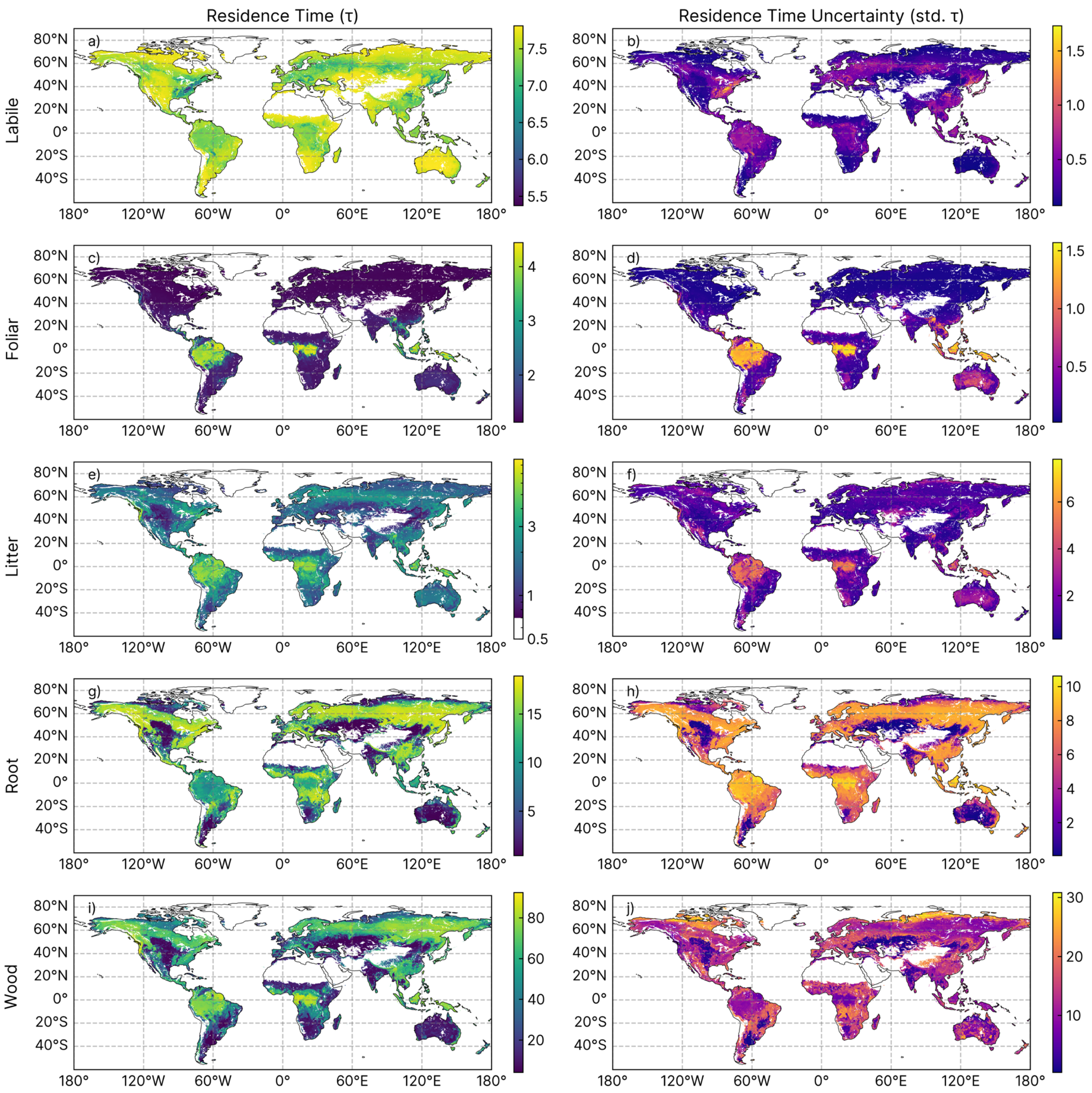

Fig. S43 | The mean residence time (τ) of different carbon pools across n=10 of the 20 ensembles of the PFT+CLIM+SOIL+AGE configuration that achieved the lowest training loss. The ensemble mean values are plotted in the first column (a,c,e,g,i), and the ensemble standard deviation are plotted in the second column (b,d,f,h,j).

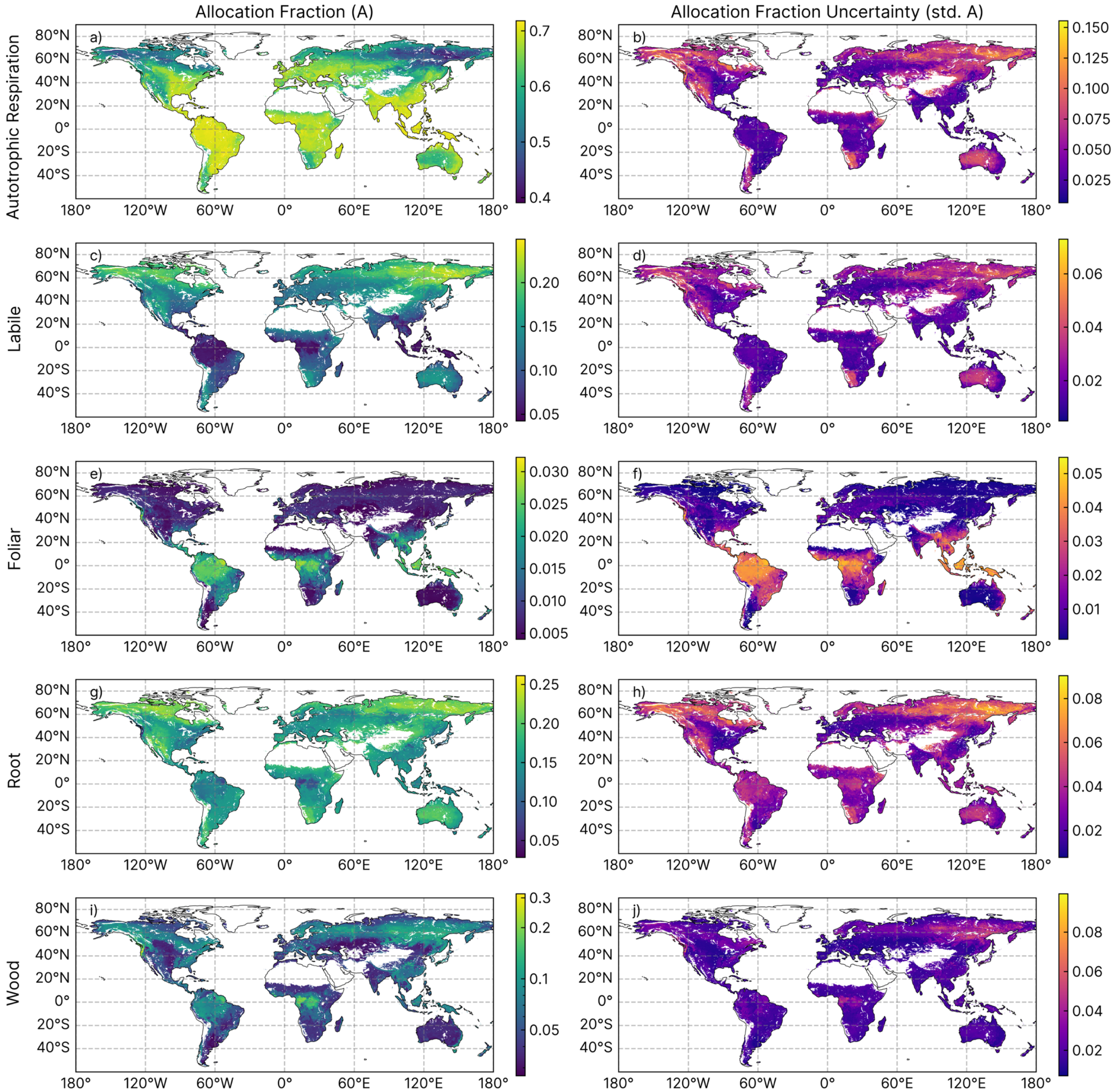

Fig. S44 | The allocation fraction (A) of different carbon pools across n=10 of the 20 ensembles of the PFT+CLIM+SOIL+AGE configuration that achieved the lowest training loss. The ensemble mean values are plotted in the first column (a,c,e,g,i), and the ensemble standard deviation are plotted in the second column (b,d,f,h,j).

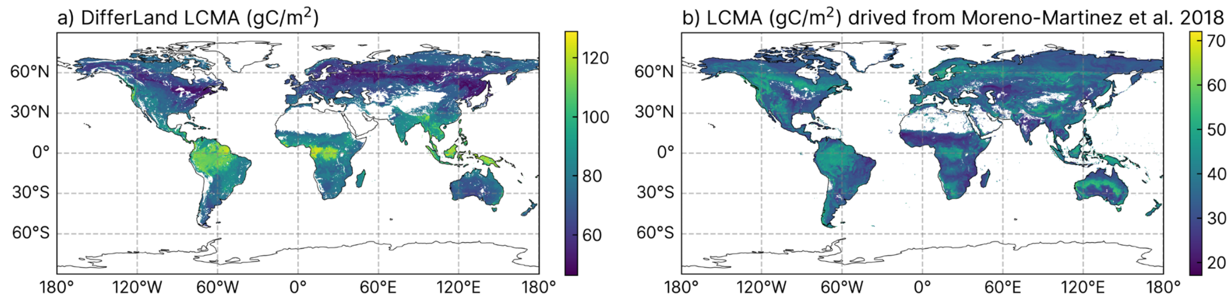

Fig. S45| a) Comparing a) DifferLand retrieved LCMA with b) an upscaled LCMA map from the TRY database and the MODIS LAI values[23].

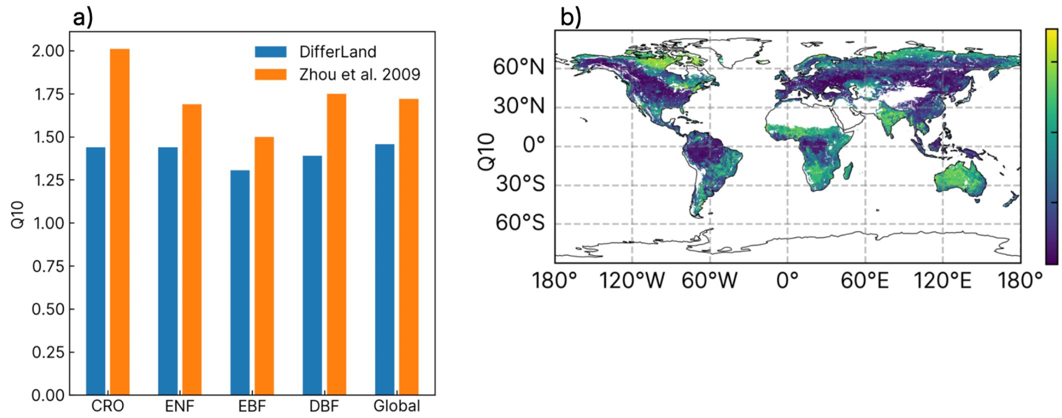

Fig. S46 | a) Comparison of DifferLand-retrieved Q_{10} with Q_{10} inverted from soil organic carbon data using Carnegie-Ames-Stanford Approach under equilibrium conditions (CASA)[24]; b) Q10 map retrieved from DifferLand.

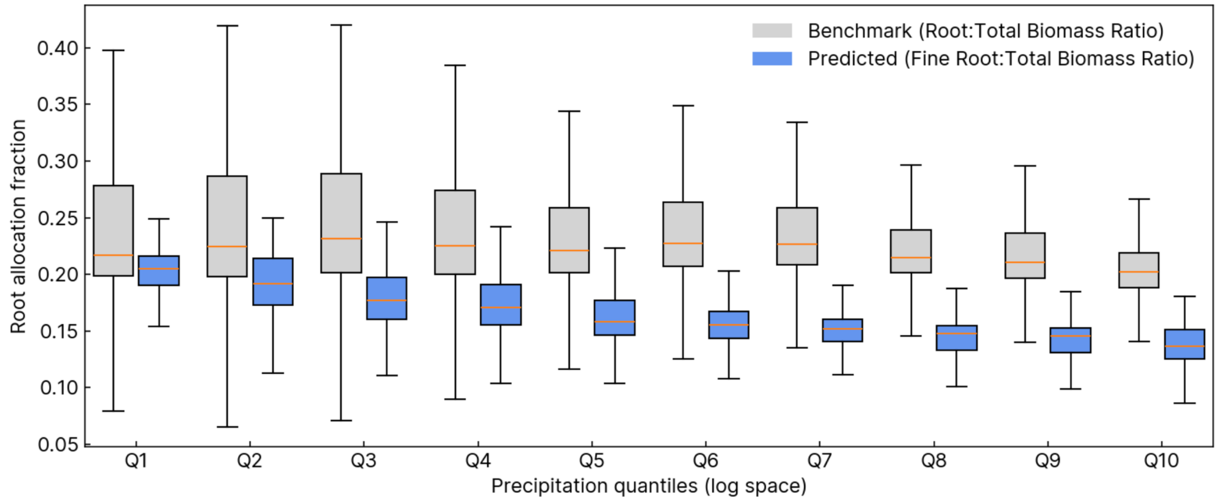

Fig. S47 | Comparison of DifferLand-simulated fine-root allocation fraction with independent observational benchmarks of root (fine + coarse) to total biomass fraction[25]. The benchmark was resampled to 0.25° resolution to match DifferLand. Each quintile of precipitation in the log space contains ~15395 pixels, with quintile median ratios denoted as yellow bars.

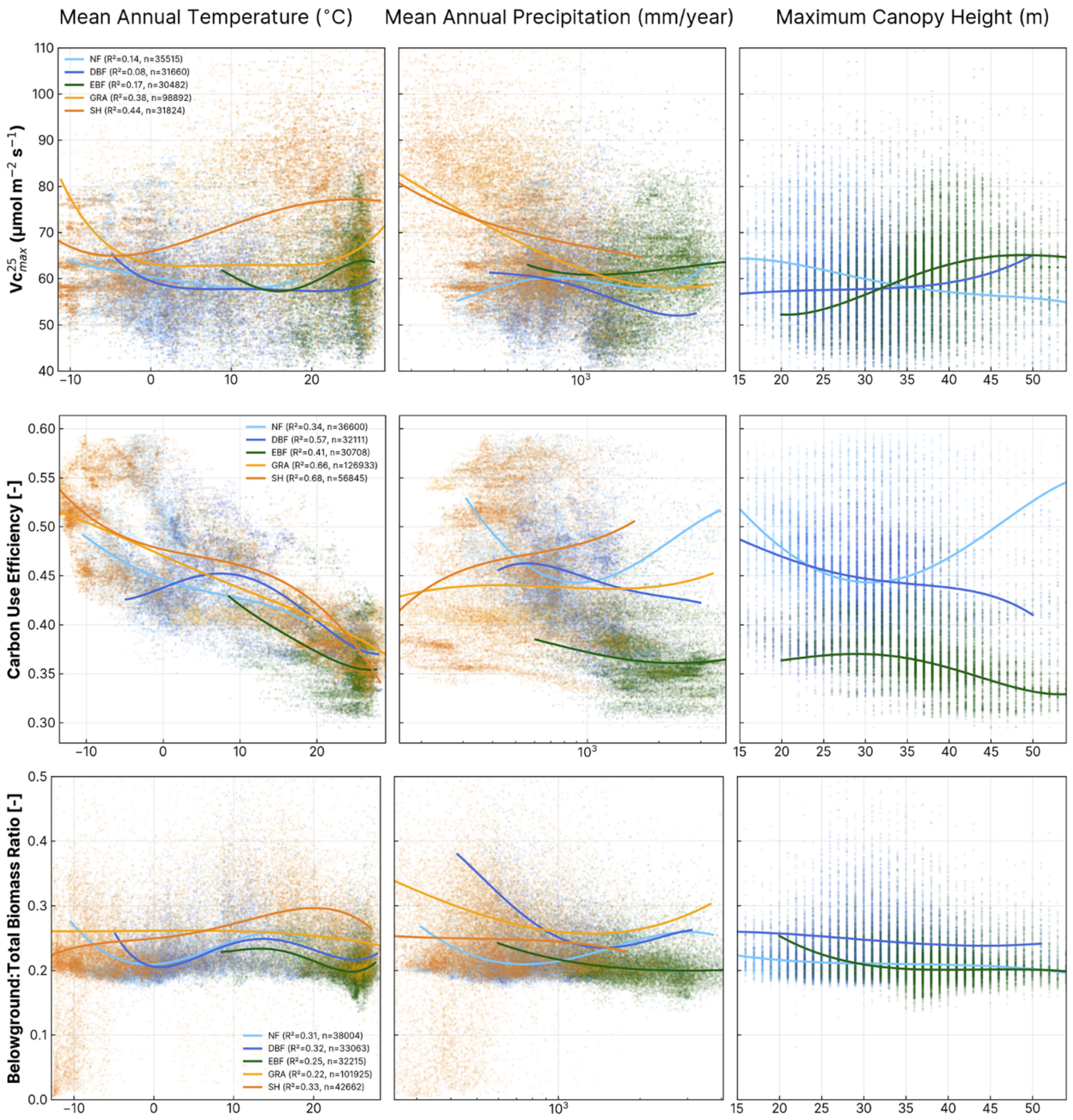

Fig. S48 | Spatial relationships between independently upscaled Vcmax25[26], carbon use efficiency[20], and belowground-to-total live biomass fraction[25] and mean annual temperature (MAT), mean annual precipitation (MAP), and maximum canopy height (MCH), shown by plant functional type. Rows correspond to different traits, and columns correspond to the same predictors used in DifferLand. For each trait and PFT, we fitted a fourth-degree multivariate polynomial using MAT, log-transformed MAP, and MCH as predictors, restricting the analysis to 0.25° grid cells in which the PFT occupies at least 90 percent of the area. The number of grid cells and the coefficient of determination are shown in the legend. These fitted relationships are compared with the conditional SHAP relationships in Fig. 4.

Table S2. The List of Model Parameters in DALEC and Their Physical Ranges

Parameter	Description	Unit	Min	Max
f_{auto}	Fraction of GPP allocation to autotrophic respiration	unitless	0.2	0.8
f_{foliar}	Fraction of NPP allocation to foliar pool	unitless	0.01	0.5
F_{labile}	Fraction of (NPP - foliar production) allocated to foliar pool	unitless	0.01	0.5
f_{root}	Fraction of (NPP - foliar production - labile production) allocated to root production	unitless	0.01	1
τ_{lab}	Residence time of carbon in the labile pool	year	1.001	8
τ_{foliar}	Residence time of carbon in the leaf pool	year	1.001	8
k_{wood}	Carbon turnover rate of the wood pool	day^{-1}	$2.5\times e^{-7}$	0.001
k_{root}	Carbon turnover rate of the fine root pool	day^{-1}	0.0001	0.01
k_{litter}	Carbon turnover rate of the litter pool	day^{-1}	0.0001	0.01
k_{som}	Carbon turnover rate of soil organic matter	day^{-1}	$1\times e^{-7}$	0.001
γ	Temperature sensitivity of litter decomposition and heterotrophic respiration	unitless	0.018	0.08
ce	Nitrogen-limited canopy efficiency of the Aggregate Canopy Model	unitless	5	50
d_{onset}	Leaf onset day, on DOY=Bday%365.25 labile C release rate reaches the maximum	unitless	365.25	1461
c_{ronset}	Labile C release period	day	30.4375	100
d_{fall}	Leaf fall day, on DOY=Fday%365.25 leaf fall rate reaches the maximum	unitless	365.25	1461

c_{rfall}	Leaf fall period	day	30.4375	150
LCMA	Leaf carbon mass per area	gC m^{-2} leaf area	5	200
uWUE	Underlying water use efficiency	gC kg $H2O^{-1}$ $hPa^{-0.5}$	0.5	30
Boese_r	Shortwave solar radiation downward adjustment factor to ET	kg H2O MJ^{-1}	0.01	0.3
$Qmax_{PAW}$	Runoff focal point of the plant available water pool	kgH_2O m^{-2}	1	100000
PAW_{fc}	Field capacity for the PAW pool	kgH_2O m^{-2}	1	10000
f_{wp}	Wilting point fraction as of field capacity	unitless	0.01	0.5
θ_p	Precipitation sensitivity of litter decomposition and heterotrophic respiration	unitless	0.01	1
k_{decomp}	Litter decomposition rate	day^{-1}	$1\times e^{-5}$	0.01
$C_{lab, i}$	Initial C stock in the labile pool	gC m^{-2}	1	2000
$C_{fol, i}$	Initial C stock in the foliar pool	gC m^{-2}	1	2000
$C_{wood, i}$	Initial C stock in the wood pool	gC m^{-2}	1	2000
$C_{root, i}$	Initial C stock in the fine root pool	gC m^{-2}	1	100000
$C_{litter, i}$	Initial C storage in the litter pool	gC m^{-2}	1	2000
$C_{som, i}$	Initial C storage in the soil organic matter pool	gC m^{-2}	1	200000
PAW_i	Initial water storage in the PAW pool	kgH_2O m^{-2}	1	10000
$CF_{ligneous}$	Combustion factor of ligneous biomass	unitless	0.01	1

CF_{DOM}	Combustion factor of dead organic matter	unitless	0.01	1
CF_{foliar}	Combustion factor of foliar biomass	unitless	0.01	1
R	Fire resilience factor	unitless	0.01	1
p_{wood}	Wood biomass coefficient in the relationship with VOD	m^2/gC	0.000001	0.00008
p_{fol}	Foliar biomass coefficient in the relationship with VOD	m^2/gC	0.0001	0.01
α_{SIF}	Slope factor GPP-SIF relationship	$(mW\ m^{-2}\ nm^{-1}\ sr^{-1})$ / $(gC\ m^{-2})$	1/35	1/3
β_{SIF}	Intercept of GPP-SIF relationship	$mW\ m^{-2}\ nm^{-1}\ sr^{-1}$	-0.5	1/6

Note: for the results in the main text, we reparametrized the allocations to carbon pools as ratios of total GPP for better interpretability, such that CUE= 1- f_{auto}, $A_{foliar} = (1 - f_{auto}) \times f_{foliar}$, $A_{labile} = (1 - f_{auto} - A_{foliar}) \times f_{labile}$, $A_{root} = (1 - f_{auto} - A_{foliar} - A_{labile}) \times f_{root}$, and $A_{wood} = 1 - f_{auto} - A_{foliar} - A_{labile} - A_{root}$. We further calculated $Q10 = e^{10\times\gamma}$.

Table S3. Hyperparameters in the DifferLand Model

Description	Value
Learning rate of the optimizer	0.0005
Number of layers in the embedding NN	3
Number of neurons in the embedding NN	32
Dimensionality of the spatial embedding	32
Total number of training epochs	199
SIF loss term weight	400
LAI loss term weight	10
Live biomass loss weight	5e-5
Soil organic matter loss weight	3e-6
GLEAM ET loss weight	1
NBE loss weight (at patch-level)	3000
NBE annual mean loss weight (at patch-level)	3000 * 3.46
Fire C emission loss weight (at patch-level)	1000
Poor water pool initialization penalty term weight*	400
EWT loss weight (at patch-level)	0.2
VOD loss weight	100
GPP eddy-covariance loss weight	500
RECO eddy-covariance loss weight	500
ET eddy-covariance loss weight	500
EDC penalty weight	10

* This term is used to prevent excessive drying of the PAW pool associated with poor initialization. See Supplementary Notes 5 of Fang & Gentine (2024)[4] for details.

Table S4. A list of TRENDY v12 LSMs/DGVMs[28] used in the study

Model Name	Model References
CABLE-POP	Wang, Y.p., et al., 2009. A global model of carbon, nitrogen and phosphorus cycles for the terrestrial biosphere. Biogeosciences 7, 2261–2282.
CLASSIC	Melton, J. R., Arora, V. K., Wisernig-Cojoc, E., Seiler, C., Fortier, M., Chan, E., & Teckentrup, L. (2020). CLASSIC v1. 0: the open-source community successor to the Canadian Land Surface Scheme (CLASS) and the Canadian Terrestrial Ecosystem Model (CTEM)–Part 1: Model framework and site-level performance. Geoscientific Model Development, 13(6), 2825-2850. Seiler, C., Melton, J. R., Arora, V. K., & Wang, L. (2021). CLASSIC v1. 0: the open-source community successor to the Canadian Land Surface Scheme (CLASS) and the Canadian Terrestrial Ecosystem Model (CTEM)–Part 2: Global benchmarking. Geoscientific Model Development, 14(5), 2371-2417.
CLM5.0	Lawrence, D.M., et al., 2019. The community land model version 5: description of new features, benchmarking, and impact of forcing uncertainty. J. Adv. Model. Earth Syst. 11 (12), 4245–4287.
E3SM	Yang, X., Thornton, P., Ricciuto, D., Wang, Y. & Hoffman, F. Global evaluation of terrestrial biogeochemistry in the Energy Exascale Earth System Model (E3SM) and the role of the phosphorus cycle in the historical terrestrial carbon balance. *Biogeosciences* **20**, 2813–2836 (2023).
EDv3	Ma, L., Hurtt, G., Ott, L., Sahajpal, R., Fisk, J., Lamb, R., ... & Sullivan, J. (2021). Global evaluation of the Ecosystem Demography model (ED v3. 0). Geoscientific Model Development Discussions, 2021, 1-41.
ISAM	Arora, V.K., Boer, G.J., 2005. A parameterization of leaf phenology for the terrestrial ecosystem component of climate models. Glob. Chang. Biol. 11 (1), 39–59. El-Masri, B., et al., 2013. Carbon dynamics in the Amazonian Basin: integration of eddy covariance and ecophysiological data with a land surface model. Agric. For. Meteorol. 182-183, 156–167.
ISBA-CTRIP	Delire, C., Séférian, R., Decharme, B., Alkama, R., Calvet, J. C., Carrer, D., ... & Tzanos, D. (2020). The global land carbon cycle simulated with ISBA-CTRIP: Improvements over the last decade. Journal of Advances in Modeling Earth Systems, 12(9), e2019MS001886.
JSBACH	Reick, C. H., Gayler, V., Goll, D., Hagemann, S., Heidkamp, M., Nabel, J. E., ... & Wilkenskjeld, S. (2021). JSBACH 3-The land component of the MPI Earth System Model: documentation of version 3.2.
JULES	Best, M. J., Pryor, M., Clark, D. B., Rooney, G. G., Essery, R., Ménard, C. B., ... & Harding, R. J. (2011). The Joint UK Land Environment Simulator (JULES), model description–Part 1: energy and water fluxes. Geoscientific Model Development, 4(3), 677-699. Clark, D. B., Mercado, L. M., Sitch, S., Jones, C. D., Gedney, N., Best, M. J., ... & Cox, P. M. (2011). The Joint UK Land Environment Simulator (JULES), model description–Part 2: carbon fluxes and vegetation dynamics. Geoscientific Model Development, 4(3), 701-722.
LPJwsl	Calle, L., & Poulter, B. (2020). Ecosystem age-class dynamics and distribution in the LPJ-wsl v2. 0 global ecosystem model. Geoscientific Model Development Discussions, 2020, 1-44.
LPX_Bern	Sitch, S., et al., 2003. Evaluation of ecosystem dynamics, plant geography and terrestrial carbon cycling in the LPJ dynamic global vegetation model. Glob. Chang. Biol. 9 (2), 161–185.
OCN	Thornton, P. E., Doney, S. C., Lindsay, K., Moore, J. K., Mahowald, N., Randerson, J. T., ... & Lee, Y. H. (2009). Carbon-nitrogen interactions regulate climate-carbon cycle feedbacks: results from an atmosphere-ocean general circulation model. Biogeosciences, 6(10), 2099-2120.
ORCHIDEE	Krinner, G., Viovy, N., de Noblet-Ducoudré, N., Ogée, J., Polcher, J., Friedlingstein, P., ... & Prentice, I. C. (2005). A dynamic global vegetation model for studies of the coupled atmosphere-biosphere system. Global biogeochemical cycles, 19(1).
VISIT	Ito, A., & Inatomi, M. (2012). Use of a process-based model for assessing the methane budgets of global terrestrial ecosystems and evaluation of uncertainty. Biogeosciences, 9(2), 759-773.

References

1. Bloom, A. A. & Williams, M. Constraining ecosystem carbon dynamics in a data-limited world: Integrating ecological ‘common sense’ in a model-data fusion framework. *Biogeosciences* **12**, 1299–1315 (2015).
2. Norton, A. J. *et al.* Improved process representation of leaf phenology significantly shifts climate sensitivity of ecosystem carbon balance. *Biogeosciences* **20**, 2455–2484 (2023).
3. Xu, L. *et al.* Changes in global terrestrial live biomass over the 21st century. *Sci. Adv.* **7**, eabe9829 (2021).
4. Fang, J. & Gentine, P. Exploring Optimal Complexity for Water Stress Representation in Terrestrial Carbon Models: A Hybrid-Machine Learning Model Approach. *J Adv Model Earth Syst* **16**, e2024MS004308 (2024).
5. Williams, M. *et al.* Predicting gross primary productivity in terrestrial ecosystems. *Ecological Applications* **7**, 882–894 (1997).
6. Norton, A. CARDAMOM-framework/CARDAMOM_v2.3. https://doi.org/10.5281/zenodo.8063861 (2023).
7. Copernicus Atmosphere Monitoring Service. CAMS global inversion-optimised greenhouse gas fluxes and concentrations. https://doi.org/10.24381/ed2851d2 (2020).
8. European Union’s Copernicus Land Monitoring Service information. Leaf Area Index 1999-2020 (raster 1 km), global, 10-daily – version 2. https://doi.org/10.2909/d5fdc595-2e03-4cbe-a39e-5f006f9cef07 (2017).
9. European Union’s Copernicus Land Monitoring Service information. Leaf Area Index 2014-present (raster 300 m), global, 10-daily – version 1. https://doi.org/10.2909/219fdc9f-616b-444b-a495-198f527b4722 (2017).
10. Li, X. *et al.* IB-AGC: Annual 25 km global live biomass carbon product from SMOS L-band passive microwave vegetation optical depth. *Sci Data* **12**, 1156 (2025).
11. Wigneron, J.-P. *et al.* Global carbon balance of the forest: satellite-based L-VOD results over the last decade. *Front. Remote Sens.* **5**, 1338618 (2024).
12. Skulovich, O., Gentine, P., Wigneron, J. P. & Xiaojun, L. GLAB-VOD: Global L-band AI-Based Vegetation Optical Depth Dataset Based on Machine Learning and Remote Sensing. Zenodo https://doi.org/10.5281/zenodo.10306094 (2024).
13. Skulovich, O., Famiglietti, C., Konings, A. & Gentine, P. *Constraining Respiration Flux and Carbon Pools in a Simple Ecosystem Carbon Model*. (2024) doi:10.22541/au.170708964.48441645/v1.
14. Bowman, K. Carbon Monitoring System Carbon Flux for Fire L4 V2 (CMSFluxFire). https://doi.org/10.5067/HO07ZJEQBMHE (2020).
15. Bowman, K. *et al.* Global and Brazilian Carbon Response to El Niño Modoki 2011–2010. *Earth and Space Science* **4**, 637–660 (2017).
16. Liu, J. *et al.* Carbon Monitoring System Flux Net Biosphere Exchange 2020 (CMS-Flux NBE 2020). *Earth System Science Data* **13**, 299–330 (2021).
17. Chu, H. *et al.* Representativeness of Eddy-Covariance flux footprints for areas surrounding AmeriFlux sites. *Agricultural and Forest Meteorology* **301–302**, 108350 (2021).
18. Li, X. & Xiao, J. Mapping Photosynthesis Solely from Solar-Induced Chlorophyll Fluorescence: A Global, Fine-Resolution Dataset of Gross Primary Production Derived from OCO-2. *Remote Sensing* **11**, 2563 (2019).
19. Bloom, A. A., Exbrayat, J.-F., Van Der Velde, I. R., Feng, L. & Williams, M. The decadal state of the terrestrial carbon cycle: Global retrievals of terrestrial carbon allocation, pools, and residence times. *Proceedings of the National Academy of Sciences* **113**, 1285–1290 (2016).
20. Luo, X. *et al.* Global variation in vegetation carbon use efficiency inferred from eddy covariance observations. *Nat Ecol Evol* https://doi.org/10.1038/s41559-025-02753-0 (2025) doi:10.1038/s41559-025-02753-0.
21. Collalti, A. & Prentice, I. C. Is NPP proportional to GPP? Waring’s hypothesis 20 years on. *Tree Physiology* **39**, 1473–1483 (2019).

22. Hongyan, Z. Global pattern of NPP to GPP ratio derived from MODIS data: effects of ecosystem type, geographical location and climate. *Global Ecology and Biogeography* (2008).
23. Moreno-Martínez, Á. *et al.* A methodology to derive global maps of leaf traits using remote sensing and climate data. *Remote Sensing of Environment* **218**, 69–88 (2018).
24. Zhou, T., Shi, P., Hui, D. & Luo, Y. Global pattern of temperature sensitivity of soil heterotrophic respiration (Q10) and its implications for carbon-climate feedback. *Journal of Geophysical Research: Biogeosciences* **114**, (2009).
25. Huang, Y. *et al.* A global map of root biomass across the world's forests. *Earth Syst. Sci. Data* **13**, 4263–4274 (2021).
26. Luo, X. *et al.* Global variation in the fraction of leaf nitrogen allocated to photosynthesis. *Nat Commun* **12**, 4866 (2021).
27. Olson, D. M. *et al.* Terrestrial Ecoregions of the World: A New Map of Life on Earth: A new global map of terrestrial ecoregions provides an innovative tool for conserving biodiversity. *BioScience* **51**, 933–938 (2001).
28. Friedlingstein, P. *et al.* Global carbon budget 2023. *Earth System Science Data* **15**, 5301–5369 (2023).
