# Supplementary material for: Differentiable land model reveals global environmental controls on latent ecological functions
Source: Nat Commun. 2026 May 21;17:6670. doi: 10.1038/s41467-026-73395-4 (PMC13381539; doi:10.1038/s41467-026-73395-4)
Supplement: Supplementary file 3 — Supplementary information [file 41467_2026_73395_MOESM3_ESM.pdf]

Description of Additional Supplementary Files

File Name: Supplementary Data 1
Description: A list of eddy covariance sites used in the study
