# Supplementary material for: Differentiable land model reveals global environmental controls on latent ecological functions
Source: Nat Commun. 2026 May 21;17:6670. doi: 10.1038/s41467-026-73395-4 (PMC13381539; doi:10.1038/s41467-026-73395-4)
Supplement: Supplementary file 5 — Supplementary information [file 41467_2026_73395_MOESM5_ESM.pdf]

nature portfolio

Corresponding author(s): Jianing Fang, Pierre Gentine

Last updated by author(s): Apr 15, 2026

Reporting Summary

Nature Portfolio wishes to improve the reproducibility of the work that we publish. This form provides structure for consistency and transparency in reporting. For further information on Nature Portfolio policies, see our Editorial Policies and the Editorial Policy Checklist.

Statistics

For all statistical analyses, confirm that the following items are present in the figure legend, table legend, main text, or Methods section.

n/a	Confirmed	
☐	☒	The exact sample size (*n*) for each experimental group/condition, given as a discrete number and unit of measurement
☐	☒	A statement on whether measurements were taken from distinct samples or whether the same sample was measured repeatedly
☒	☐	The statistical test(s) used AND whether they are one- or two-sided<br>*Only common tests should be described solely by name; describe more complex techniques in the Methods section.*
☐	☒	A description of all covariates tested
☒	☐	A description of any assumptions or corrections, such as tests of normality and adjustment for multiple comparisons
☐	☒	A full description of the statistical parameters including central tendency (e.g. means) or other basic estimates (e.g. regression coefficient) AND variation (e.g. standard deviation) or associated estimates of uncertainty (e.g. confidence intervals)
☒	☐	For null hypothesis testing, the test statistic (e.g. *F*, *t*, *r*) with confidence intervals, effect sizes, degrees of freedom and *P* value noted<br>*Give P values as exact values whenever suitable.*
☒	☐	For Bayesian analysis, information on the choice of priors and Markov chain Monte Carlo settings
☒	☐	For hierarchical and complex designs, identification of the appropriate level for tests and full reporting of outcomes
☐	☒	Estimates of effect sizes (e.g. Cohen's *d*, Pearson's *r*), indicating how they were calculated

Our web collection on statistics for biologists contains articles on many of the points above.

Software and code

Policy information about availability of computer code

Data collection	https://github.com/JianingFang/DifferLand_Global
Data analysis	python 3.12 jax numpy xarray pandas shap netCDF4 matplotlib cartopy scikit-learn optax tqdm

For manuscripts utilizing custom algorithms or software that are central to the research but not yet described in published literature, software must be made available to editors and reviewers. We strongly encourage code deposition in a community repository (e.g. GitHub). See the Nature Portfolio guidelines for submitting code & software for further information.

Data

Policy information about availability of data

All manuscripts must include a data availability statement. This statement should provide the following information, where applicable:

- Accession codes, unique identifiers, or web links for publicly available datasets
- A description of any restrictions on data availability
- For clinical datasets or third party data, please ensure that the statement adheres to our policy

The DifferLand driver files, including processed spatial predictors, forcing data, and model configuration files, are publicly accessible on Zenodo at https://doi.org/10.5281/zenodo.13984225. Additionally, processed model output used to generate the figures is also deposited in the same Zenodo repository. The DifferLand model code is available at Zenodo (https://doi.org/10.5281/zenodo.19410424).
All datasets used in this study are publicly available from their respective repositories. Key datasets and their DOIs include: MODIS land cover product MCD12C1 (https://doi.org/10.5067/MODIS/MCD12C1.061), ERA5 reanalysis (https://doi.org/10.24381/cds.adbb2d47), Global 30 Arc-Second Elevation dataset GTOPO30

(https://doi.org/10.5066/F7DF6PQS), global forest age 1km dataset (https://doi.org/10.17871/ForestAgeBGI.2021), Harmonized World Soil Database v1.2 (https://doi.org/10.3334/ORNLDAAC/1247), NOAA global CO2 record (https://doi.org/10.15138/9N0H-ZH07), GRACE/GRACE-FO terrestrial water storage (https://doi.org/10.5067/TEMSC-3JC63), the LCSPP-MODIS photosynthesis proxy (https://doi.org/10.5281/zenodo.11658088), the reprocessed MODIS Version 6.1 Leaf Area Index dataset (https://doi.org/10.4121/21858717.v2), CMS-Flux Net Biome Exchange (NBE) (https://cmsflux.jpl.nasa.gov/get-data/gcp-2023/), and GLEAM v4.2a evapotranspiration data (https://www.gleam.eu/#downloads). GFED5 burned area data are available at https://zenodo.org/records/7668424, while GFED5 fire emissions data are available from the Global Fire Data portal (https://www.globalfiredata.org/). MODIS burned area fraction data are available via NASA EarthData Search (https://search.earthdata.nasa.gov/). Global live biomass dataset from Xu et al. (2021) is available at can be accessed at https://zenodo.org/records/4161694 or by contacting the authors of the dataset.

Additional datasets include: Copernicus Atmosphere Monitoring Service (CAMS) global inversion-optimized greenhouse gas fluxes and concentrations (https://doi.org/10.24381/ed2851d2); Copernicus Leaf Area Index 1999–2020, 1 km, 10-daily (https://doi.org/10.2909/d5fdc595-2e03-4cbe-a39e-5f006f9cef07); Copernicus Leaf Area Index 2014–present, 300 m, 10-daily (https://doi.org/10.2909/219fdc9f-616b-444b-a495-198f527b4722); IB-AGC global live biomass carbon product (https://doi.org/10.5281/zenodo.15676176); GLAB-VOD vegetation optical depth dataset (https://doi.org/10.5281/zenodo.10306094); and CMS-Flux Fire L4 V2 carbon fluxes (https://doi.org/10.5067/HO07ZJEQBMHE).

Eddy covariance observations used in this study were compiled from the FLUXNET2015 dataset, AmeriFlux FLUXNET, ICOS, and OzFlux networks. These datasets are publicly available under the Creative Commons Attribution 4.0 International (CC BY 4.0) license. Detailed site-level citations, dataset access links, and metadata are provided in Supplementary Data 1.

Research involving human participants, their data, or biological material

Policy information about studies with human participants or human data. See also policy information about sex, gender (identity/presentation), and sexual orientation and race, ethnicity and racism.

Reporting on sex and gender	Not applicable. Human data not used for this study.
Reporting on race, ethnicity, or other socially relevant groupings	Not applicable. Human data not used for this study.
Population characteristics	Not applicable. Human data not used for this study.
Recruitment	Not applicable. Human data not used for this study.
Ethics oversight	Not applicable. Human data not used for this study.

Note that full information on the approval of the study protocol must also be provided in the manuscript.

Field-specific reporting

Please select the one below that is the best fit for your research. If you are not sure, read the appropriate sections before making your selection.

☐ Life sciences ☐ Behavioural & social sciences ☒ Ecological, evolutionary & environmental sciences

For a reference copy of the document with all sections, see nature.com/documents/nr-reporting-summary-flat.pdf

Ecological, evolutionary & environmental sciences study design

All studies must disclose on these points even when the disclosure is negative.

Study description	Training and evaluating a differentiable terrestrial biosphere model by assimilating satellite and in-situ observations of ecological and carbon cycle processes.
Research sample	Datasets used in this study are publicly available from their respective repositories. Key datasets and their DOIs include: MODIS land cover product MCD12C1 (https://doi.org/10.5067/MODIS/MCD12C1.061), ERA5 reanalysis (https://doi.org/10.24381/cds.adbb2d47), Global 30 Arc-Second Elevation dataset GTOPO30 (https://doi.org/10.5066/F7DF6PQS), global forest age 1km dataset (https://doi.org/10.17871/ForestAgeBGI.2021), Harmonized World Soil Database v1.2 (https://doi.org/10.3334/ORNLDAAC/1247), NOAA global CO2 record (https://doi.org/10.15138/9N0H-ZH07), GRACE/GRACE-FO terrestrial water storage (https://doi.org/10.5067/TEMSC-3JC63), the LCSPP-MODIS photosynthesis proxy (https://doi.org/10.5281/zenodo.11658088), the reprocessed MODIS Version 6.1 Leaf Area Index dataset (https://doi.org/10.4121/21858717.v2), CMS-Flux Net Biome Exchange (NBE) (https://cmsflux.jpl.nasa.gov/get-data/gcp-2023/), and GLEAM v4.2a evapotranspiration data (https://www.gleam.eu/#downloads). GFED5 burned area data are available at https://zenodo.org/records/7668424, while GFED5 fire emissions data are available from the Global Fire Data portal (https://www.globalfiredata.org/). MODIS burned area fraction data are available via NASA EarthData Search (https://search.earthdata.nasa.gov/). Global live biomass dataset from Xu et al. (2021) is available at can be accessed at https://zenodo.org/records/4161694 or by contacting the authors of the dataset.<br><br>Additional datasets include: Copernicus Atmosphere Monitoring Service (CAMS) global inversion-optimized greenhouse gas fluxes and concentrations (https://doi.org/10.24381/ed2851d2); Copernicus Leaf Area Index 1999–2020, 1 km, 10-daily (https://doi.org/10.2909/d5fdc595-2e03-4cbe-a39e-5f006f9cef07); Copernicus Leaf Area Index 2014–present, 300 m, 10-daily (https://doi.org/10.2909/219fdc9f-616b-444b-a495-198f527b4722); IB-AGC global live biomass carbon product (https://doi.org/10.5281/zenodo.15676176); GLAB-VOD vegetation optical depth dataset (https://doi.org/10.5281/zenodo.10306094); and CMS-Flux Fire L4

	V2 carbon fluxes (https://doi.org/10.5067/HO07ZJEQBMHE).<br><br>Eddy covariance observations used in this study were compiled from the FLUXNET2015 dataset, AmeriFlux FLUXNET, ICOS, and OzFlux networks. These datasets are publicly available under the Creative Commons Attribution 4.0 International (CC BY 4.0) license. Detailed site-level citations, dataset access links, and metadata are provided in Supplementary Data 1.
Sampling strategy	To assimilate multi-resolution observational constraints at both fine (0.25deg) and coarse (4 deg x 5 deg) resolutions, we first divided the globe into 3240 4 deg x 5 deg patches, each corresponding to the grid of coarse-resolution datasets. Each patch contains 320 nested fine-resolution grid cells (Fig. S5). We filtered these patches to retain only those with at least 32 valid vegetated fine-resolution grid cells. Out of the 944 patches meeting this criterion, 10% of the patches (N = 95) were randomly selected and reserved for model testing (Fig. S5c). Prior to training each ensemble member, we randomly sampled 90% of the remaining 849 patches for training, while the unselected patches formed a development set used for hyperparameter tuning and monitoring training progress (Fig. S5a).
Data collection	Data were obtained from existing datasets as described in the data availability section.
Timing and spatial scale	Global studies at 0.25 degree spatial resolution. Simulation period from 2001-2023.
Data exclusions	The first two years of the simulation were treated as spin-up and excluded from the analysis. Non-vegetated or very sparsely vegetated pixels were excluded.
Reproducibility	The results can be fully reproduced using the code and data provided. All model code is open sourced. Detailed instructions for how to set up the software environment and perform the simulation were provided in the documentation within the code repository.
Randomization	Not applicable.
Blinding	Not applicable.

Did the study involve field work? ☐ Yes ☒ No

Reporting for specific materials, systems and methods

We require information from authors about some types of materials, experimental systems and methods used in many studies. Here, indicate whether each material, system or method listed is relevant to your study. If you are not sure if a list item applies to your research, read the appropriate section before selecting a response.

Materials & experimental systems

n/a	Involved in the study
☒	☐ Antibodies
☒	☐ Eukaryotic cell lines
☒	☐ Palaeontology and archaeology
☒	☐ Animals and other organisms
☒	☐ Clinical data
☒	☐ Dual use research of concern
☒	☐ Plants

Methods

n/a	Involved in the study
☒	☐ ChIP-seq
☒	☐ Flow cytometry
☒	☐ MRI-based neuroimaging

Plants

Seed stocks	Not applicable
Novel plant genotypes	Not applicable
Authentication	Not applicable
