# Supplementary material for: Differentiable land model reveals global environmental controls on latent ecological functions
Source: Nat Commun. 2026 May 21;17:6670. doi: 10.1038/s41467-026-73395-4 (PMC13381539; doi:10.1038/s41467-026-73395-4)
Supplement: Supplementary file 2 — Supplementary information [file 41467_2026_73395_MOESM2_ESM.pdf]

nature portfolio

Peer Review File

Differentiable Land Model Reveals Global Environmental Controls on Latent Ecological Functions

Corresponding Author: Mr Jianing Fang

This file contains all reviewer reports in order by version, followed by all author rebuttals in order by version.

Version 0:

Reviewer comments:

Reviewer #1

(Remarks to the Author)
This manuscript introduces an ambitious and well-executed hybrid modeling framework, DifferLand, to advance the spatial representation of ecological parameters in terrestrial biosphere models. The differentiable coupling of a neural network with a process-based model is both novel and timely, enabling the learning of latent ecological traits across space while preserving physical consistency. The authors provide a good analysis, from predictive skill against multiple observational datasets to interpretable gradients in ecological trait distributions. Their results offer crucial insight into the limitations of traditional PFT-based parameterizations and propose a path forward that could improve Earth system modeling across scales.
The study is methodologically sound, data-rich, and well-validated. It addresses a central challenge in Earth system science —how to balance model flexibility with physical interpretability and data constraints. The approach is not only of interest to climate and ecological modelers but is a significant contribution to the broader field of physics-informed machine learning. However, several points should be addressed before further considering publication.

General comment:
1. Provide more discussion on how DifferLand could be integrated into operational TBMs or ESMs.
2. Clarify how uncertainty propagates in data-sparse regions.

Specific comment:
1. In the abstract, the role of explainable AI (SHAP) could be explicitly introduced since it's a central tool in interpreting learned relationships.
2. In the introduction, please introduce the concept of latent ecological functions earlier (before page 3). Also, could cite more ML-for-climate studies from NeurIPS/ICLR/ICML to bridge communities.
3. In methodology, the transition from spatial predictors → latent parameters → TBM outputs is well-structured, but could benefit from a schematic of data flow across scales (train/test/ensemble dynamics). In addition, is the DALEC model run at each time step with full forcing or only climatology?
4. In model performance and validation, please add discuss spatial mismatch between model resolution and flux tower footprints more deeply. Also, acknowledge the model's limits in the tropics and Australia—good honesty here, could explore how to overcome.
5. About parameter spatialization and Vvariance partitioning, please consider showing SHAP partitioning here to preface explainable AI section. Also, a comparison to traditional PFT-based TBM output would further quantify improvement.
6. About SHAP analysis in environment–parameter relationships, please add interaction effects or pairwise SHAP interaction plots (e.g., temperature × age). Also, please consider regional SHAP breakdowns (e.g., different biome regimes). Further clarify how SHAP uncertainty is handled (e.g., through ensemble SDs).
7. About trait coordination via PCA, please link PCA results more explicitly to SHAP-derived patterns. Also, explore whether PCA axes could guide future trait-based model clustering.
8. In discussion, add more on SHAP as a tool for process discovery (e.g., revealing unknown thresholds, nonlinearities), and could elaborate on how DifferLand could help forecast climate-driven shifts in ecological function.
9. In Figure S26 (SHAP contributions) could be expanded with group-wise or biome-level SHAP summaries. Please consider including a worked example pixel to show how environment → parameter → prediction → observation alignment works.

(Remarks on code availability)
After briefly reviewing in the core code (DALEC993.py), the provided code for the DifferLand framework demonstrates a well-structured and technically sound implementation of a differentiable land surface model using JAX. It integrates neural networks with process-based ecological modeling in a novel and flexible way, allowing for parameter inference and global trait estimation with physical consistency. The core simulation logic is robust, modular, and clearly aligned with the scientific goals of the manuscript. However, while the underlying code quality is strong, the overall reproducibility and usability of the code are currently limited by the absence of essential supporting materials. Specifically, the code lacks a README file, installation or usage instructions, defined dependencies, and example workflows or pretrained models. As such, the results of the paper are only partially reproducible at this stage, and external users would face significant challenges in deploying the model without additional guidance. Nonetheless, with modest improvements to documentation and packaging, this code has high potential to become a valuable resource for the Earth system modeling and machine learning communities.

Reviewer #2

(Remarks to the Author)
This study presents DifferLand, an AI-enabled TBM designed to evaluate the impact of spatially-varying parameters on global carbon and water flux simulations. The main findings are: 1) spatially discrete and categorical PFTs provide limited explanation for spatial parameter variability, 2) local climate and forest demography offer greater explanatory power, and 3) the key drivers of spatial parameter variability are growing season length, leaf economics, and agricultural intensity. DifferLand represents an innovative integration of physical processes and AI. While the study's motivation is clearly articulated and logically structured, several major concerns the authors should thoroughly address:

1.The baseline: The current baseline definition is misleading, as it does not accurately quantify the impact of omitting spatially varying parameters. In existing TBMs, parameters are constant within a PFT but vary across PFTs. Therefore, this configuration should serve as the baseline, corresponding to the PFT only setup in this paper. Then the spatially-varying parameter (even within each PFT) setup should come only from CLIM, SOIL, and AGE, which are not explicitly considered in current TBMs. An incorrect baseline could result in misleading conclusions.

A pertinent question is: At 0.25-degree resolution, what is the impact of excluding spatial variability within each PFT? It would be beneficial to present maps comparing results between PFT-only and PFT+CLIM+SOIL+AGE setups to underscore the importance of spatially varying parameters.

2.Long-term prediction: DifferLand generates spatially varying parameters (as many alternative approaches/studies have already done), but do these parameters hold in time to enable more reliable long-term prediction? As a modeler, our perspective is to improve the predictability into the future, not only seasonally, but also over decades and centuries. Can DifferLand significantly improve TBMs' predictability, as seen in Fig. 4b of Gordon and Doney (2018) DOI: 10.1126/science.aam8328? Otherwise, its practical utility in enhancing TBM predictability is limited.

A related question: can DifferLand improve prediction of the response of C cycle to CO_2 fertilization and warming? As this study assumes space-for-time substitution (which is a big assumption), even with a relatively short-term training period with sizable interannual variability should still offer some improvement in model parameterization of CO_2 and warming response. Also, how about predictability to extreme events in DifferLand?

3.Impact of uncertainty in remote sensing data: This study heavily relies on remote sensing products, which carry substantial uncertainties, and their respective uncertainties are asymmetrical. For example, it's well-documented that MODIS LAI has significant bias (in both magnitude and seasonality) in the tropics; VOD data has huge data gaps in the tropics; LTSIF has never been validated in the tropics; CMS data has coarse resolutions that may not necessarily help constrain the spatial variability of model parameters within each PFT, etc. How do these uncertainties affect parameter retrieval? Would alternative data products alter the conclusions? A thorough assessment of these uncertainties is essential to ensure DifferLand's robustness.

4.Agriculture intensity: The claim that agricultural intensity is a major driver of spatially varying parameters requires caution. Agriculture intensity would indeed impact carbon fluxes, however, not through parameter spatialization, but through genetic cultivar differences and management practices, as well as their relatively rapid temporal changes and technology adoptions (as opposed to natural systems). This comes to the question of how agriculture systems are treated in DALEC and DifferLand? As this is an intermediate complexity model, the agriculture system must be overly simplified. Therefore, it is puzzling that how agriculture intensity is impacting spatial variability of model parameters. Also, agriculture intensity can mean different things in different communities, what exactly the definition of agriculture intensity is in this study?

5.Parameter sensitivity and resolved/unresolved processes: Identifying which of the 40 parameters is most sensitive to spatialization is crucial. The most sensitive parameters indicate unresolved processes needing data-driven models, while the least sensitive highlight robust processes where spatial variation isn't a priority.

6.Performance of C storage: This study focuses on carbon fluxes only, of course due to more accessible and mature satellite products. But when quantifying carbon sequestration, the above- and below-ground biomass are more critical. Can the spatial-varying parameters in DifferLand improve carbon storage simulations?

7.Model assessment: The current model assessment combines spatial and temporal variability, likely resulting in high R^2 values. Would these results hold if decomposed into seasonal, interannual, and trend analyses? The current high R^2 is primarily driven by seasonal and spatial variations.

8.Technical questions:
a.How top-down and bottom-up data products complement each other? What if only top-down? And What if only bottom-up?
b.It must be noted SIF is NOT GPP; their decoupling (if using linear scaling) is most evident in the pan-tropics and high-latitude conifers. Details are lacking on how SIF was used to constrain parameters related to GPP.
c.Details are lacking on how NEE was determined from CMS NBE products.
d.Fig 3: I guess the impact of MAT has already been considered in current TBMs. For example, Vcmax25 is a parameter, but Vcmax depends on temperature variations. This means impact of local climate on parameters have already been incorporated in TBMs. So DifferLand identified additional MAT impact?
e.Fig S21 and Fig. S22: It'd be great to enlarge the spatial maps of parameters. Just eyeballing of the small maps, there are not too much differences of these parameters from PFT-specific values.

(Remarks on code availability)

Reviewer #3

(Remarks to the Author)
I co-reviewed this manuscript with one of the reviewers who provided the listed reports. This is part of the Nature Communications initiative to facilitate training in peer review and to provide appropriate recognition for Early Career Researchers who co-review manuscripts.

(Remarks on code availability)

Reviewer #4

(Remarks to the Author)
Comments:
The manuscript "Differentiable Land Model Reveals Global Environmental Controls on Latent Ecological Functions" presents a hybrid modeling framework that combines process-based and machine-learning approaches to infer ecological parameters and their environmental controls. The study aims to improve upon traditional plant functional type (PFT)-based parameterizations by integrating diverse Earth observations and leveraging a differentiable land model. While the methodology is novel and technically sophisticated, the manuscript places a strong emphasis on modeling techniques rather than providing new ecological or climate science insights. Given this focus, the study may be better suited for a specialized modeling journal rather than Nature Communications, which prioritizes broader conceptual advancements with broad interdisciplinary appeal.
The study effectively demonstrates that PFTs alone explain less than half of the spatial variability in ecological parameters controlling carbon fluxes and vegetation states. However, a more direct comparison with real-world observations is needed to support the validity of the model's inferred relationships. The brief discussion of R^2 values (Lines 138–146) lacks clarity on whether these statistics represent spatial or temporal variability, or whether they compare an ensemble mean across time with multiple field datasets. Including direct field comparisons, such as those presented in Figures S10 and S11, in the main text would enhance the credibility of the model's performance.
Furthermore, the manuscript would benefit from clearer distinctions between different sources of variability in the presented results. For example, the discussion in Line 228 mentions retrieved carbon use efficiency (CUE) values and independent site-level measurements, but it is unclear how these datasets were aggregated or how model performance is being evaluated. The interpretation of hybrid model outputs would be strengthened by explicitly defining whether specific results reflect training data variance, full predictor hybrid model performance, or validation against independent field observations.
The discussion of parameter importance is interesting but requires further elaboration. Figures S14 and S16 suggest that adding climate (CLIM) parameters improves model performance beyond PFT-based parameterizations, while additional variables (SOIL, AGE) provide diminishing returns. However, Figures S18 and 2f indicate that AGE and SOIL still contribute significantly in certain contexts. A more detailed analysis of these spatial variations—such as region-specific differences in parameter importance—would be valuable in clarifying these results. Additionally, the study notes that the hybrid model has lower predictive power for water cycle variables (ET, EWT), but the manuscript does not fully explore potential reasons for this limitation. Given the importance of water availability in future climate projections, discussing how the model handles moisture-related processes and whether additional constraints could improve performance would be worthwhile.
The study's methodological framework relies on four spatiotemporal parameter groups (PFT, CLIM, SOIL, AGE), which appear to have relatively low temporal variability. This could partially explain why the "Forcing and Structure" group (which includes daily temperature and precipitation) accounts for a large fraction of spatiotemporal variance (Line 190). Clarifying whether the hybrid model operates at a daily timestep or whether forcing variables are temporally aggregated to match the resolution of parameter groups would help resolve this issue.
Additional technical refinements would also improve clarity. For example, Figure 2 should explicitly state whether it represents an aggregate over the entire time period (2010–2023), and the omission of ET from panel 2f should be confirmed

as intentional or corrected. The manuscript also contains minor grammatical and typographical errors (e.g., Line 62: "requires" should be "require"; Line 241: "level-level" should likely be "leaf-level"), which should be addressed in revision.

Recommendation:
While the manuscript presents a technically rigorous modeling framework, its primary contributions lie in methodological advancements rather than broad conceptual or interdisciplinary insights. The study focuses heavily on the implementation and validation of a novel modeling approach rather than on generating new ecological or climate science findings with broad implications. Given this emphasis, I recommend rejection from Nature Communications but suggest resubmission to a more specialized journal focused on land surface modeling, machine learning applications in Earth system science, or geophysical research.

(Remarks on code availability)

Reviewer #5

(Remarks to the Author)
I co-reviewed this manuscript with one of the reviewers who provided the listed reports. This is part of the Nature Communications initiative to facilitate training in peer review and to provide appropriate recognition for Early Career Researchers who co-review manuscripts.

(Remarks on code availability)

Version 1:

Reviewer comments:

Reviewer #1

(Remarks to the Author)
Dear authos,

I appreciate the substantially revised manuscript. By the current version, it has been reasonable to accept for publication.For the following reason:

The authors addressed the reviewers' comments by tightening the framing, expanding analyses, and clarifying methods. They reframed the narrative to emphasize ecological mechanisms and the added value of their approach beyond technical novelty, and they standardized evaluation by adopting a clear PFT-only baseline for comparison.
Methodologically, they distinguished static spatial predictors from time-varying forces, documented the full data flow with a schematic and a worked pixel example, and broadened observational evaluation by incorporating additional eddy-covariance networks and multiple alternative remote-sensing products in a structured sensitivity suite. Concerns about spatial scale mismatch were tackled with representativeness tests, while interpretability was strengthened through biome-level SHAP summaries and clearer variance partitioning of predictor contributions. They acknowledged and analyzed weaker skills in moisture-related processes, outlined concrete upgrades for stomatal conductance and multi-layer hydrology, and compared performance against established DGVM/TRENDY benchmarks across relevant timescales. Editorially, definitions (e.g., NBE) and terminology (e.g., agricultural intensity) were clarified, figures and metrics were cleaned up for consistency, and code, documentation, and tutorials were released to support transparency and reproducibility. Remaining scope limits—particularly true multi-decadal predictability—were explicitly discussed with reasoned justification and a roadmap for future work.

(Remarks on code availability)
The code (based on core code DALEC993.py) demonstrates several notable strengths that indicate high-quality scientific software development.

The implementation provides a comprehensive carbon cycle model with seven carbon and water pools, multiple flux pathways, and sophisticated process representations, including phenology, fire disturbance, and water stress. Particularly impressive is the flexible framework offering six different water stress formulations, which allows for scientific experimentation and comparison of different modeling approaches. The code carefully implements minimum and maximum bounds on all pools with detailed mass balance corrections, ensuring physical consistency throughout the simulation.

Despite these strengths, to me only a technical issues that should be addressed to improve code robustness. The code uses several magic numbers such as WATER_POOL_SCALE = 1500 and GPP_SCALE = 8 without documentation of their scientific justification or rationale.

Reviewer #2

(Remarks to the Author)
I appreciate the substantial revisions and the authors' efforts to address my earlier concerns—for example, establishing an appropriate baseline configuration and analyzing parameter importance. That said, the extensive rewrite has made the manuscript more challenging to follow than the previous version. The study's objectives are not stated with sufficient clarity, partly due to ambiguous terminology and missing definitions of key concepts. I also noted apparent inconsistencies—for instance, in the discussion of acclimation effects and the suitability of the approach for extrapolation/long-term prediction. Clarifying these issues is essential for readers to understand the scientific contribution and how this work advances the broader goal of improving predictability of ecosystem dynamics under climate change.

1. Ambiguity/Inconsistency of terminology. There are many important concepts that were not clearly defined or misused. Here are some examples:
• Both "Plant-Type" and "PFT" were used throughout the manuscript and even in the same sentence. What are their differences?
• What is the definition of "environment gradient" – one of the core concepts in this manuscript? What is its difference from environmental forcing? Gradient can mean many different things in different dimensions under different timescales.
• "Ecological parameters/traits": In Fig. 1 caption, "photosynthesis" is one of the 40 ecological parameters, however, photosynthesis is a process, a variable, a flux, not a parameter. Similarly, In the response letter: LAI and SIF are latent ecological traits, which are incorrect. Moreover, photosynthetic efficiency, and carbon use efficiency are all variables not parameters, so they are supposed to vary in time and space
• "Ecological latent space": How to validate the latent space? How do that compare in the context of theoretical/field ecology research?
Clear concept and consistent terminology is fundamental to ensure readers understands the manuscript.

2. Long-term prediction. I appreciate some of the efforts that the authors made to address the suitability of Differland for improving long-term prediction, i.e., over decadal to century timescales. Considering that "Improving the long-term predictability of terrestrial biosphere model is a key motivation for developing our hybrid differentiable framework" (quote in the response letter) and "embedding such relationships into terrestrial models establishes a pathway toward adaptive models that better predict ecosystem resilience under climate change", it is important to thoroughly address this issue. However, I found that the argument and evidence that authors provided are insufficient. The two pieces of "supporting evidences" provided are: 1) lower RMSE in simulated NBE relative to TRENDY simulations (Fig. S8), and 2) evaluation of DifferLand with CAMS NBE for a different period from the training period. These two tests do not necessarily mean "generalization into the future" in the time dimension, although it can imply that it can generalize in space during the contemporary period. It is also inconsistent that, on the one hand, the authors said "full decadal-to-centennial testing is beyond the scope of this study", but on the other hand, the authors made a dedicated section "Implications under changing climate" using extrapolation approach based on the assumption of space for time substitution. The key problem is that all observational products, used for training DifferLand, represent the environmental regime and variations only for a limited period, while future prediction is for a totally new/novel regime that was never seen by contemporary observational product. Yes, the training period of 2010-2022 differs from the validation period 2003-2009, but the difference between these two period is NOT at the same level of magnitude for their difference with the period of 2080-2099 (as is shown in Fig. S2, the second test). Even though DifferLand is doing a superb job in optimizing parameter, and especially the combination of parameters in the latent space, it does not necessarily mean these parameters/traits and their relationship with ambient environmental conditions will hold into the future. But in line 774-775, the authors "assumed that the relationships between parameters and the environment remained stable throughout the study period", and apply such relationship for extrapolation. It is risky to assume the generalizability of DifferLand to the future, and the extrapolation results can be misleading.
In addition, one of the fundamental assumptions here: Space-for-time substitution, can cause misleading results: Evans et al., 2025: https://www.nature.com/articles/s41558-025-02392-0 . So the authors have to be really cautious in extrapolating their findings into the long-term future predictability – the ultimate goal.

3.Data uncertainty. I appreciate the authors' massive efforts to evaluate the uncertainty of satellite product on the final results. But I'm a little confused about Text S4 and Fig. S30-31. Do these figures show the absolute magnitude of NBE or deltaNBE? The figures say the former while the text says the latter. It is also confusing that what does R2 means for deltaNBE, DeltaLAI, etc, in Fig. S29. With lack of clarity of these figures, it's hard to evaluate the robustness of the results to the data choice.
Moreover, when looking at Fig. S31d, there sign of NBE in south American Tropics (the Amazon region) is different between CAMS inversion and CMS inversion: the former shows an overall weak carbon source while the latter shows strong carbon sink. Because DifferLand was trained on CMS product (over 2010-2023), its predicted NBE during 2003 to 2009 is also a carbon sink, which is in great contrast to the strong source from CAMS inversion.

4. Agriculture intensity. Even though the authors replaced "agricultural intensity" with "cropland fraction", it does not address my previous concern on why cropland is a major driver of spatially varying ecological parameters? This is not intuitive. Neither cropland distribution nor agriculture activity can impact the ecological traits for natural vegetation. They do not have any causal connections. Cropland fraction appears as the 3rd axes is primarily an artifact that satellite products of carbon fluxes, used for DifferLand training, is greatly impacted by agriculture activities. But "agriculture activity" or "cropland fraction" itself is not the cause for the ecological parameters or the coordination across them.
BTW, this phrase pf "agricultural intensity" still exists in the revised manuscript – line 820.

5. Acclimation: Their authors' notion and discussion on "Acclimation" is self-contradictory. Specifically, in Line 771-786 "We

note that the conclusions drawn from this research are dependent on the timeframe considered for the observations. In this 23-year study, spatial predictors were treated as temporally invariant, under the assumption that their temporal variations are negligible compared to their mean spatial gradients. Furthermore, we assumed that the relationships between parameters and the environment remained stable throughout the study period, implying that plant acclimation and adaptation to environmental changes occur on much longer timescales".
When addressing my questions on Vcmax, the authors responded that "The temperature dependence we identify (now Fig. 4a) does not represent the short-term enzymatic limitation of Rubisco, but rather the long-term acclimation of photosynthetic efficiency to climatological mean annual temperature. By capturing this acclimation, DifferLand highlights an additional source of parameter variation along environmental gradients that is typically missing in TBMs assuming fixed Vcmax25."
So it's very confusing whether DifferLand included acclimation or not, from authors' texts.

6. Abstract. I recommend a thorough revision of the abstract to enhance clarity. Suggestions below.
- Line 9: Do ecosystems primarily reflect evolutionary history or current environment? – which aspects of ecosystems – morphology, composition, functional traits, fluxes, storage? As the first sentence setting the tone of the manuscript, it is important to have precise and clear description.
- Line 10: "unresolved questions" – The answer to the question is known, because its from both evolutionary history and current environment. What is unknown is how do traits combine and coordinate, and whether represent such coordination can make a difference in improving predictability of TBMs for future environments.
Line 10-13: there is no logical connection between the two clauses.
Line 13: "govern" means "determine"; traits itself do not determine the carbon fluxes or vegetation dynamics, it only "impact".
Line 14: "yet such multivariate relationships cannot be directly observed" – this is already being observed by many studies, which also cited by the authors.
Line 17: "DifferLand" is an approach, it cannot "explain" the variance; only certain processes/mechanisms can "explain" variances of observed variables.
Line 19-20: The "nonlinear interactions" is already known, not uniquely revealed by "interpretable machine learning"; but ML can capture such nonlinear interactions.
Line 21: "such relationships" – be explicit.

7. Fig. 4. Fig. 4 and Fig. S1 seem to contradict to each other. The similar shapes of traits-temperature relationship (e.g., slopes) across PFTs in Fig. 4 indicate that the S3 scenario is sufficient – "Consistent scaling across PFTs – mean trait values differ among PFTs, but trait-environment relationship are similar across PFTs".

8. SIF and GPP relationship. Although the authors justify the linear relationship between SIF and GPP at the monthly scale and beyond, their slope/intercept values in Table S2 are wild. Theoretically, there should be no intercept between them. SIF is directly related to the actual electron transport rate, which is balanced with carboxylation in the dark reaction. Their mechanistic equations are all multipliable, no additive.

Other comments:
1. Validation and comparison with existing datasets/studies: As this work aims not only to demonstrate a technical framework (Figs. 1-2) but also to test ecological hypotheses and facilitate knowledge discovery (e.g., Figs. 3-4), assessing the accuracy of the inferred parameters is equally important.
(a) While I appreciate the authors' efforts in comparing two inferred parameters (CUE and LCMA, Figs. S39 and S42) with independent datasets and site-level measurements, the validation appears limited and largely qualitative. More quantitative analyses would add credibility.
(b) The manuscript would also benefit from supporting the findings in Fig. 4 with observational evidence or laboratory experiments.
(c) Fig. S36 shows low variability across ensembles initialized with different starting values, suggesting robustness of the inferred parameters. However, these values may still be affected by structural errors in the physics-based model, not only in the hydrology components (as discussed in Text S7) but also potentially in any components, which should be discussed. The inferred values and their linkage with environmental variables may also be sensitive to the selection of data constraints. While the authors tested how the selection of data constraints affects relative performance of hybrid models (Fig. S29), its impact on knowledge discovery (Figs. 3-4) remains to be evaluated.

2. Improved model performance vs increased number of predictors and degrees of freedom: Although the full set (PFT+CLIM+SOIL+AGE) performs best compared to the PFT-only setting, in many cases the improvement is incremental (Figs. S15-18). Have the authors accounted for the increased degrees of freedom associated with using substantially more predictors? How might this affect the comparison and interpretation of the relative importance of PFT versus other spatialization variables?
More broadly, the rationale for choosing these spatialization variables should be clarified. Is there any variable selection procedure? Additionally, could model performance be artificially improved simply by including more predictors that introduce spatial variability, even if some of them are only weakly or incidentally related to the underlying processes?
I appreciate that the authors tested different datasets for calibration. However, the spatialization predictors may also have uncertainties, especially forest age. It may lead to incorrect interpretation for predictor attribution. In addition to the MPI-BGC data, the authors may consider testing with the global forest age dataset (GFAD) (https://doi.pangaea.de/10.1594/PANGAEA.897392).
The comparison between simulated NBE from DifferLand and TRENDY DGVMs is interesting (Figs. S8-S12). However, since DifferLand is calibrated using CMS NBE, using CMS NBE as the reference may not represent an entirely independent comparison. The description in Lines 236-239 may therefore be inaccurate.

L986-987: MCD12C1 is provided at 0.05 degree, not 500m.

(Remarks on code availability)

Reviewer #3

(Remarks to the Author)
I co-reviewed this manuscript with one of the reviewers who provided the listed reports. This is part of the Nature Communications initiative to facilitate training in peer review and to provide appropriate recognition for Early Career Researchers who co-review manuscripts.

(Remarks on code availability)

Reviewer #4

(Remarks to the Author)
The authors have addressed all of my concerns adequately. I look forward to seeing how the scientific community reacts to this work.

(Remarks on code availability)

Reviewer #5

(Remarks to the Author)
I co-reviewed this manuscript with one of the reviewers who provided the listed reports. This is part of the Nature Communications initiative to facilitate training in peer review and to provide appropriate recognition for Early Career Researchers who co-review manuscripts.

(Remarks on code availability)

Version 2:

Reviewer comments:

Reviewer #2

(Remarks to the Author)
The authors have provided detailed and thoughtful responses to our previous comments. In particular, they have conducted additional validation of the inferred parameters with independent datasets, evaluating the magnitudes, spatial patterns, and retrieved trait–environment relationships, substantially strengthens the robustness of this study. The authors also performed additional tests to assess the potential impact of increased degrees of freedom associated with inclusion of additional predictors. Both the experiments of (1) PFT + perm[CLIM, SOIL, AGE] and (2) PFT+LATLON suggest that the improved performance of DifferLand with the selected predictors truly reflects its capability to capture meaningful environmental controls and to generalize spatially.

Overall, the authors have adequately addressed the concerns raised during the review process. I have no further comments and believe the manuscript is suitable for publication.

(Remarks on code availability)

Reviewer #3

(Remarks to the Author)
I co-reviewed this manuscript with one of the reviewers who provided the listed reports. This is part of the Nature Communications initiative to facilitate training in peer review and to provide appropriate recognition for Early Career Researchers who co-review manuscripts.

(Remarks on code availability)

Open Access This Peer Review File is licensed under a Creative Commons Attribution 4.0 International License, which permits use, sharing, adaptation, distribution and reproduction in any medium or format, as long as you give appropriate credit to the original author(s) and the source, provide a link to the Creative Commons license, and indicate if changes were made.
In cases where reviewers are anonymous, credit should be given to 'Anonymous Referee' and the source.
The images or other third party material in this Peer Review File are included in the article's Creative Commons license, unless indicated otherwise in a credit line to the material. If material is not included in the article's Creative Commons license and your intended use is not permitted by statutory regulation or exceeds the permitted use, you will need to obtain permission directly from the copyright holder.
To view a copy of this license, visit https://creativecommons.org/licenses/by/4.0/

Point-by-Point Responses to the Reviewers

Note: Reviewers' original comments are shown in black, our responses in blue, and the corresponding revisions to the manuscript in brown (with line number in the tracked change version of the manuscript).

Reviewer #1 (Remarks to the Author):

This manuscript introduces an ambitious and well-executed hybrid modeling framework, DifferLand, to advance the spatial representation of ecological parameters in terrestrial biosphere models. The differentiable coupling of a neural network with a process-based model is both novel and timely, enabling the learning of latent ecological traits across space while preserving physical consistency. The authors provide a good analysis, from predictive skill against multiple observational datasets to interpretable gradients in ecological trait distributions. Their results offer crucial insight into the limitations of traditional PFT-based parameterizations and propose a path forward that could improve Earth system modeling across scales.

The study is methodologically sound, data-rich, and well-validated. It addresses a central challenge in Earth system science—how to balance model flexibility with physical interpretability and data constraints. The approach is not only of interest to climate and ecological modelers but is a significant contribution to the broader field of physics-informed machine learning. However, several points should be addressed before further considering publication.

We sincerely thank the reviewer for their constructive evaluation and insightful feedback, which have greatly contributed to improving the quality and clarity of our manuscript. In response, we have carefully revised the manuscript to incorporate the reviewer's suggestions. Specifically, we have extended the SHAP analysis to examine plant type-specific differences in trait–environment relationships and interaction effects, enhanced our calibration strategy to improve model performance in data-sparse regions, evaluated uncertainties arising from scale mismatches between remote sensing and eddy covariance observations, and added comparisons with traditional PFT-based TBMs/LSMs. Additionally, we have improved the structure of our codebase and provided detailed documentation to facilitate its use and adaptation for diverse research applications. We hope these revisions will not only strengthen the current study but also broaden the accessibility and impact of DifferLand as a tool for exploring new ecological hypotheses. Please find below our detailed responses to each of the reviewer's comments.

General comment:

1. Provide more discussion on how DifferLand could be integrated into operational TBMs or ESMs.

We thank the reviewer for this insightful suggestion. In the revised manuscript, we have expanded the discussion to clarify how the insights gained from DifferLand could be integrated into operational terrestrial biosphere models (TBMs) and Earth system models (ESMs), and the potential broader implications of this integration.

We propose that hybrid-physics modeling offers a promising pathway for parameter calibration in operational land surface models (LSMs), a process that is currently constrained by the high dimensionality of the parameter space and the substantial computational costs involved (Raoult et al., 2024). Many existing parameter estimation studies in LSMs have focused on only one or a few parameters at a time, often assigning uninformative and independent priors—represented by diagonal prior covariance matrices—which misrepresent potential parameter covariation and confounding effects (Raoult et al., 2024). By first identifying parameters that are both observationally constrained and climatically sensitive within an intermediate-complexity framework, researchers can prioritize a tractable subset of candidate parameters for subsequent analysis in full-complexity LSMs. Moreover, the robust spatial coordination of ecological traits revealed in our study provides a meaningful source of predictability to help address the "curse of dimensionality" in complex models (Fisher & Koven, 2020; Xu & Trugman, 2021). By encoding these correlation relationships between parameters into an informative prior covariance matrix, we can robustly constrain the parameter search space for more complex models and reduce equifinality.

In the long term, the differentiable programming paradigm explored in DifferLand offers a promising avenue for addressing the structural and parametric biases that currently limit confidence in long-term projections of the terrestrial carbon sink (Bonan & Doney, 2018). Differentiable programming enables end-to-end learning of ecological functional relationships within an ecologically consistent modeling framework, creating new opportunities to uncover emergent patterns in ecosystem processes (Shen et al., 2023). By integrating multi-modal observations of today's climate and ecological states, these emergent patterns can provide tighter constraints on uncertainties in future climate projections than traditional emergent-constraint studies, which largely depend on simple univariate linear relationships between current and projected climate (Bowman et al., 2018). Furthermore, coupling differentiable land models

with differentiable general circulation models, such as NeuralGCM (Kochkov et al., 2024), opens a powerful future pathway for jointly learning biophysical and biogeochemical feedbacks between land and atmosphere across sub-seasonal to decadal timescales. While implementing a differentiable land model may require significant initial technical and numerical investment—particularly in translating legacy components written in C or Fortran into modern differentiable frameworks—the potential long-term benefits are considerable. Such integration holds strong potential to improve the fidelity of future projections for both the terrestrial carbon cycle and the broader climate system in next-generation hybrid Earth system models (Schneider et al., 2017).

We have incorporated these discussions in Line 831-850 of the revised text.

2. Clarify how uncertainty propagates in data-sparse regions.

We thank the reviewer for raising this crucial question. In DifferLand, most of the global observational constraints (e.g., gridded remote sensing datasets and atmospheric inversion of net biosphere exchange) have in theory near-continuous spatial coverage over land. However, the quality of the optical-sensor-based datasets are often lower in tropical forests (due to frequent cloud cover and sensor saturation) and high-latitude regions (due to low solar zenith angle and snow cover), whereas passive L-band radiometer-based products (i.e., VOD and VOD-derived biomass estimates) are subject to data gaps after radio-frequency interference (RFI) filtering in China, India, North America, and parts of Europe (Wigneron et al., 2024; H. Yang et al., 2023). In contrast, data imbalance issue is more prominent in the distribution of eddy covariance data, as there are more assimilated observations in North America and Europe compared with other continents (Fig. R1).

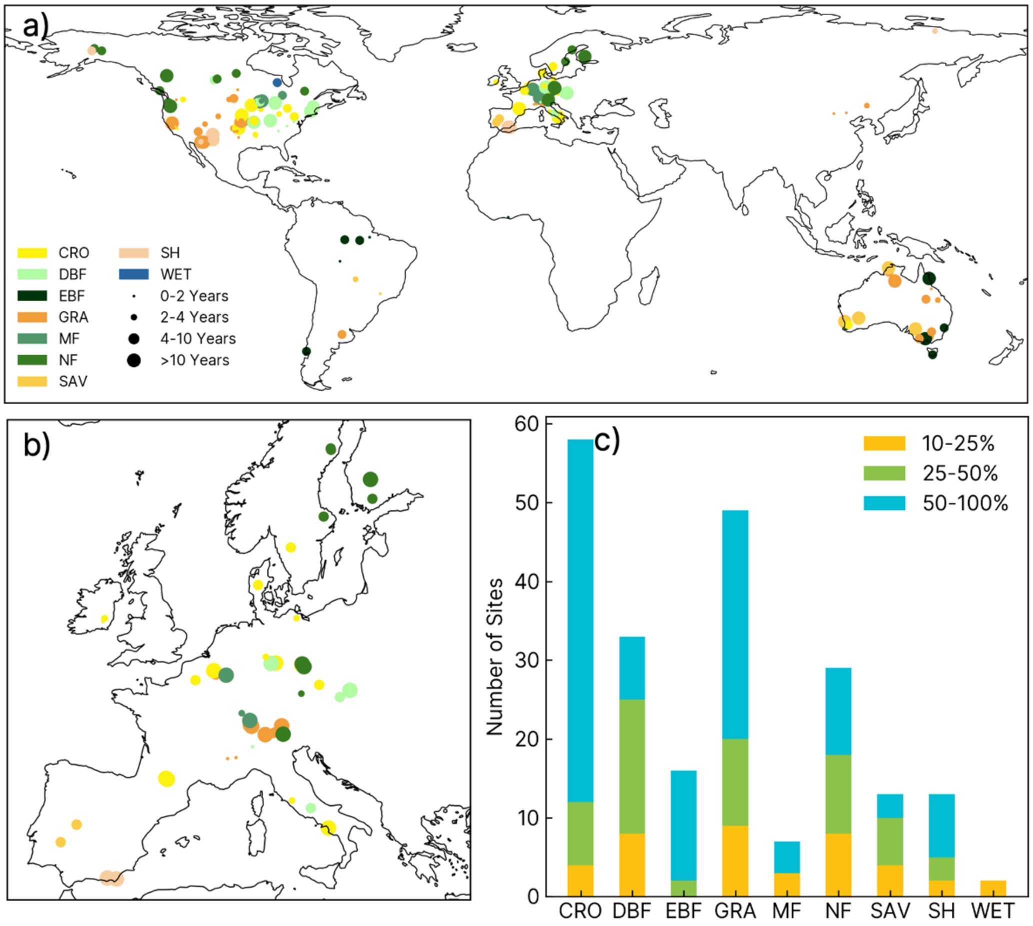

Fig. R1 (Fig. S33 in SI) | Spatial distribution of eddy covariance sites used in this study. Marker size indicates the length of available GPP observations at each site. b) Zoomed-in view of sites located in Europe. c) Distribution of sites by vegetation type. Colors indicate spatial homogeneity, defined as the fraction of the 0.25º grid cell that shares the same vegetation type as reported at the site level, categorized into three classes at 10%, 25%, and 50% thresholds.

In this study, we adopt a hierarchical modeling approach (Gelman et al., 1995) to reconcile imperfect observational data streams within a *unified latent ecological space*, structured by a global spatialization network. The framework assumes that parameter–environment relationships constrained by observations in data-rich regions can be generalized to reduce uncertainties in data-sparse areas. The predictive skill of these inferred relationships ultimately depends on the validity of the model structure, the generality of the learned dependencies, and the strength of observational constraints. To assess how uncertainty propagates into data-sparse regions, we examined the spatial patterns of model performance. We found that performance in SIF, ET, and LAI tends to be lower in tropical forests along the equator—likely due to signal saturation in optical remote sensing—and in the Australian hinterland, where sparse vegetation and

low signal-to-noise ratios limit canopy measurements. Nevertheless, DifferLand is still able to capture the first-order spatial gradients of ecosystem fluxes and the seasonal cycles of key parameters in these regions (Fig. R2).

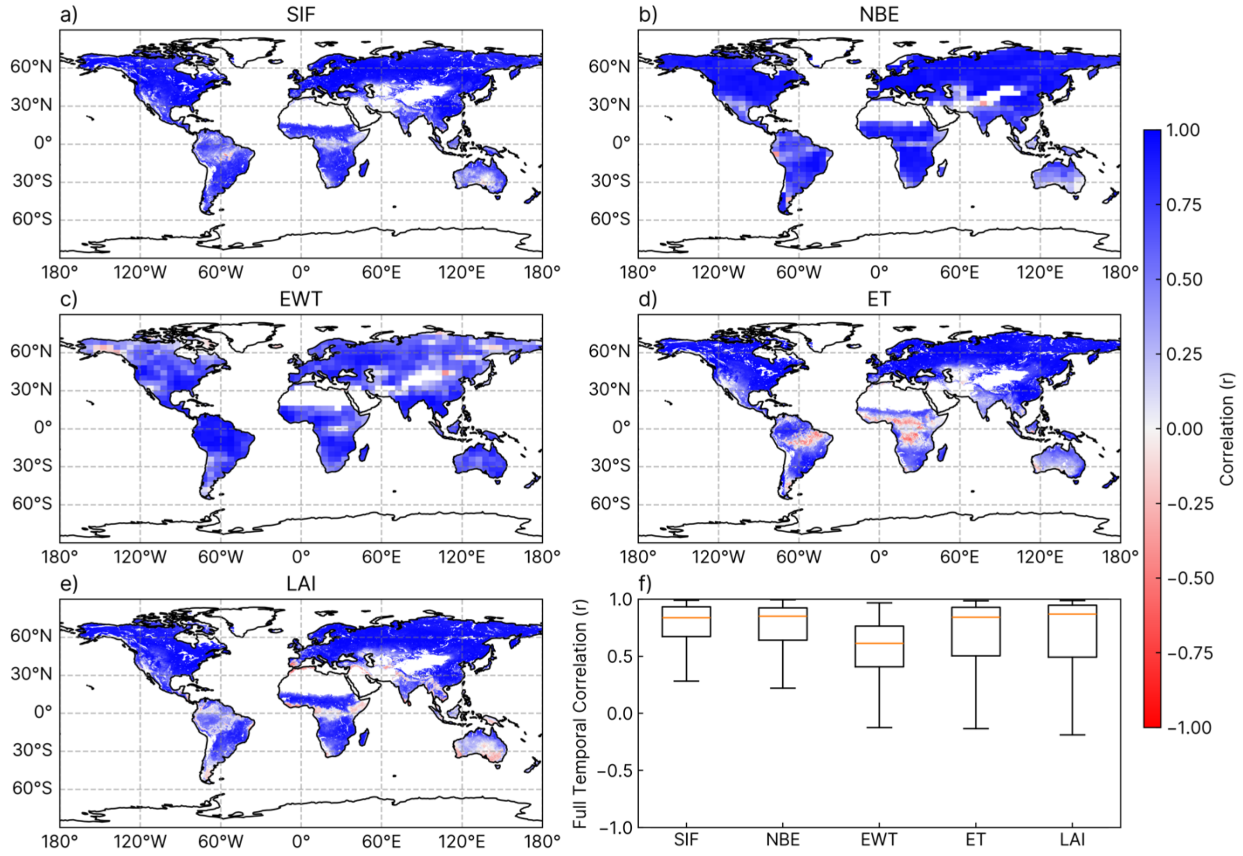

Fig. R2 (Fig. S13 in SI) | The pixelwise temporal correlation between simulated and observed variables. a) SIF, b) NBE, c) EWT, d) ET, e) LAI. The panels display the average correlation values across the 10 out of the 20 ensemble members using the full set of predictors with the lowest training loss. f) Shows the distribution of pixelwise correlations across pixels.

The effect of data imbalance is also evident in the eddy covariance datasets. To assess potential limitations, we evaluated the root mean square error and correlation coefficients of towers across different biomes (Fig. R3 and Fig. R4). Model–observation agreement was strongest in deciduous broadleaf, needleleaf, mixed forests, and grasslands ($r > 0.8$), where eddy covariance coverage is relatively dense. In contrast, correlations were weaker ($r = 0.3$–0.5) at savanna and evergreen broadleaf forest sites, where tower observations are sparse. Croplands present a special case: despite being well represented in the training (36 sites) and validation (5 sites) sets, they exhibited comparatively large model–data mismatches. This likely reflects the high spatial heterogeneity of managed agricultural systems, where

grid-cell averages poorly represent tower footprints, as well as the absence of explicit agricultural processes in the model. These findings highlight that data imbalance drives higher uncertainties in certain biomes and can guide future satellite missions and in-situ campaigns to improve coverage in data-sparse regions.

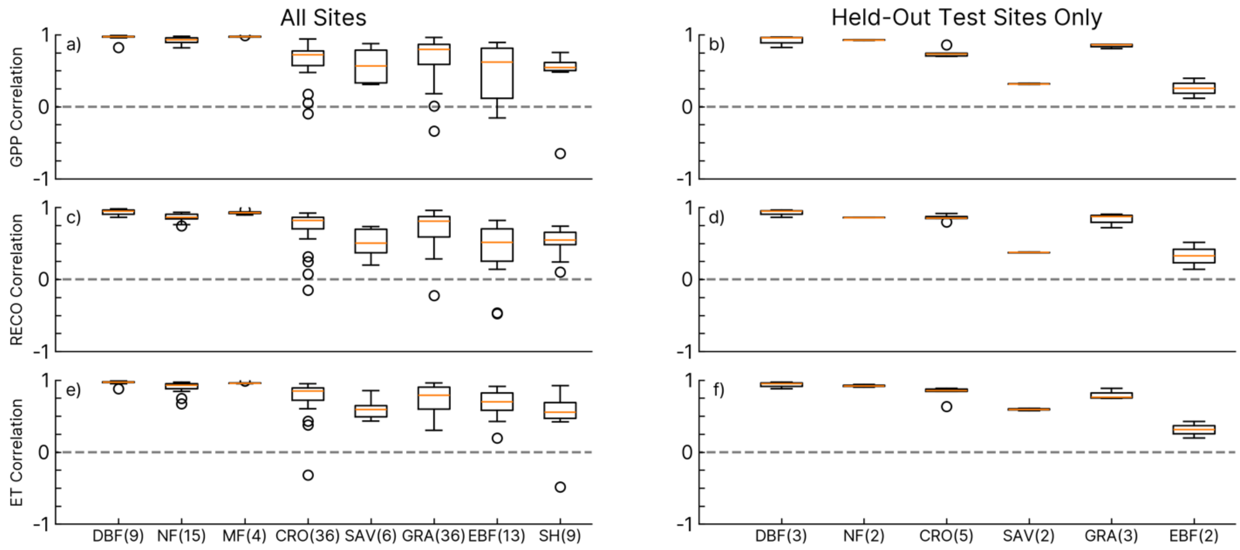

Fig. R3 (Fig. S19) | Correlation between DifferLand modeled GPP, RECO, and ET and eddy covariance estimates. The left column shows all eddy covariance sites, where the right column shows only sites that overlap with the test pixels withheld from training.

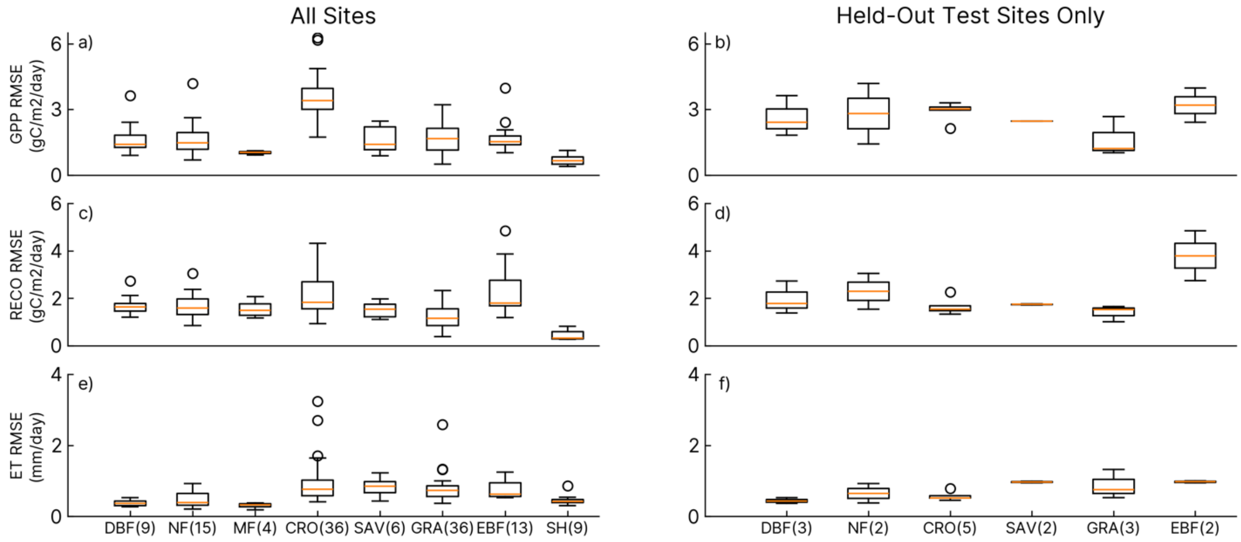

Fig. R4 (Fig. S20) | Root mean square error (RMSE) between DifferLand modeled GPP, RECO, and ET and eddy covariance estimates. The left column shows all eddy covariance sites, where the right column shows only sites that overlap with the test pixels withheld from training.

Specific comment:

1. In the abstract, the role of explainable AI (SHAP) could be explicitly introduced since it's a central tool in interpreting learned relationships.

Thanks a lot for this suggestion. We have included the role of explainable AI in the revised Abstract.

Line 19-21: “Interpretable machine learning shows that these coordinated axes emerge from nonlinear interactions between plant-type attributes and local environment.”

2. In the introduction, please introduce the concept of latent ecological functions earlier (before page 3). Also, could cite more ML-for-climate studies from NeurIPS/ICLR/ICML to bridge communities.

Thank you for the suggestion. We have revised the abstract and introduction to present the concept of latent ecological functions earlier in the manuscript.

In the abstract, we have (Line 9-17): “Do ecosystems primarily reflect evolutionary history or current environment? Predicting land–atmosphere exchange hinges on this unresolved question. Plant traits adapt to particular environments over evolutionary timescales, yet their individual relationships with current climate and soils are often obscured by limited sampling, plant-type effects, and multiple adaptive strategies that can yield similar outcomes. Crucially, it is the coordination of traits—rather than any single trait—that governs vegetation dynamics and ecosystem fluxes, yet such multivariate relationships cannot be directly observed. We present DifferLand, a differentiable hybrid model that integrates process understanding with machine learning to uncover latent trait–environment relationships from global satellite and in-situ observations (2001–2023).”

In the introduction, we have (Line 91-101): “Nonetheless, despite these limitations, mechanistic TBMs still encode key physiological processes—photosynthesis, respiration, allocation, turnover, and responses to environmental stress—and thus provide our best process-based approximation of how traits, represented as model parameters, together mediate carbon and water exchanges with the atmosphere. This perspective motivates our central hypothesis: that multivariate trait–environment relationships may be learned by inverting a TBMs using observed global vegetation dynamics and spatial environmental predictors of plant traits. The tradeoffs and covariation among high-dimensional ecological parameters, together with the nonlinear interactions of environmental gradients, can be effectively represented in an ‘ecological latent space’ (Fan et al., 2025)—a physics-informed machine learning–derived low-dimensional embedding (Brown et al., 2025) of global ecological functions—that enhances predictions of land–atmosphere carbon and water exchange beyond models relying on PFT-based parameterizations.”

We agree with the reviewer that citing additional ML-for-climate and AI-for-science studies helps to better bridge communities. Specifically, our notion of learning latent trait–environment relationships parallels the classical machine learning concept of *representation learning* (Bengio et al., 2013), in which retrieved ecological axes serve as ecologically consistent, low-dimensional representations of multivariate trait–environment interactions. Our approach also resonates with recent advances in latent-space data assimilation for atmospheric forecasting using autoencoders (Fan et al., 2025). In addition, it shares synergies with the growing interest in foundational models for geospatial data that integrate multiple remote sensing and reanalysis products to learn effective embeddings of Earth system processes (Brown et al., 2025). We highlight these works in our paper to underscore such connections and encourage stronger collaboration between ecological modeling and AI research communities.

3. In methodology, the transition from spatial predictors → latent parameters → TBM outputs is well-structured, but could benefit from a schematic of data flow across scales (train/test/ensemble dynamics). In addition, is the DALEC model run at each time step with full forcing or only climatology?

We would like to thank the reviewer for this great suggestion. In our Supplementary Information, we include a figure showing the split for train/test workflow with detailed descriptions in the caption (Fig. S6).

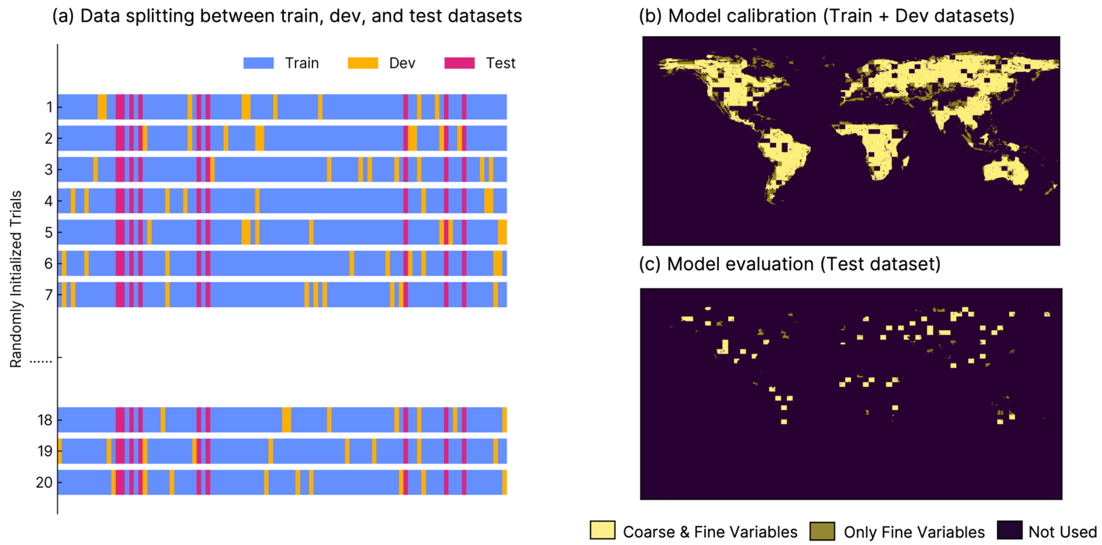

Fig. S6 | Data splitting and the multi-resolution data assimilation approach. We first reserved 10% of all the available 4×5° patches for independent model testing. We then randomly sampled 90% of the remaining 4×5° patches for model training (81% of the total data) for each randomly initialize trials and used the rest of the patches for model development and hyperparameter tuning (9% of total data). The training and development sets (but not the test set) are then combined to retrain the model to obtain latent ecological parameters and environment-parameter relationships for further analysis. (a) shows schematics

of data splitting and bootstrapping for 20 randomly initialized trials. (b) and (c) show the locations of data used for calibration and evaluation. Pixels with more than 80% non-vegetated surface are removed from the experiment. A 4×5° patch is included during training as a minibatch (containing a maximum of 320 nested 0.25° samples) if at least 10% of the nested 0.25° cells are vegetated land surface, but we only assimilate coarse resolution variables (CMS-Flux NEE and GRACE EWT) as a batch level constraint if at least 80% of the 0.25-degree nested grids are vegetated land surface.

4. In model performance and validation, please add discuss spatial mismatch between model resolution and flux tower footprints more deeply. Also, acknowledge the model's limits in the tropics and Australia—good honesty here, could explore how to overcome.

Thank you for this valuable suggestion. We fully agree that the scale mismatch between the model resolution (0.25°) and flux tower footprints (O[100–1000 m]) is an important source of uncertainty, as substantial spatial heterogeneity can exist within a single grid cell. In the revised manuscript, we performed a detailed sensitivity analysis on the spatial representativeness of assimilated eddy covariance sites. Yet, we found that varying the filtering thresholds for site representativeness had little effect on the spatial gradients of GPP and RECO, whereas the inclusion or exclusion of eddy covariance data strongly influenced these gradients. We have included these detailed results and discussions in Text S5 of the Supplementary Information.

Text S5. Sensitivity analysis of different filtering criteria for spatial mismatches between assimilated eddy covariance datasets and model grid cells.

A major source of uncertainty in this study arises from the scale mismatch between the eddy covariance (EC) tower footprint (Chu et al., 2021)—where turbulent fluxes of land–atmosphere exchange are measured, typically spanning ~100 m to several kilometers depending on wind direction and tower height—and the much larger 0.25° model grid cell (~25 km). To reduce this mismatch, we compute grid-cell mean fluxes by averaging across multiple EC sites within a cell whenever possible and exclude towers that represent atypical managed habitats (e.g., AU-Lox, an almond orchard in South Australia's Riverland). However, many grid cells contain only a single operating site, if any. To ensure minimal consistency between site vegetation and the containing grid cell, we require a threshold fraction of plant functional type (PFT) coverage for a site to be included. Higher thresholds improve representativeness but reduce site availability, whereas lower thresholds increase the risk of spatial mismatch. As a compromise, we tested four filtering schemes: a minimal coverage of 10% (ΔEC10, N = 213 sites), 25% (default, N =

180), and 50% (ΔEC50, N = 123), along with a control case excluding all EC data (ΔEC). Note that the thresholds here represent a minimal coverage cutoff, but the typical plant type representativeness across sites are substantially higher. The spatial distribution of the included sites and the number of sites per plant functional type under each threshold are shown in Fig. S33 (Fig. R1).

We found that changing the filtering thresholds or even leaving out eddy covariance data does not alter our main conclusion: adding spatial predictors improves the model's ability to predict ecological variables, as shown in Fig. R5 (Fig. S34). These results suggest the general spatial gradient of the environmental variables is mostly constrained by remote sensing and atmospheric inversion variables, rather than the sparse eddy covariance data.

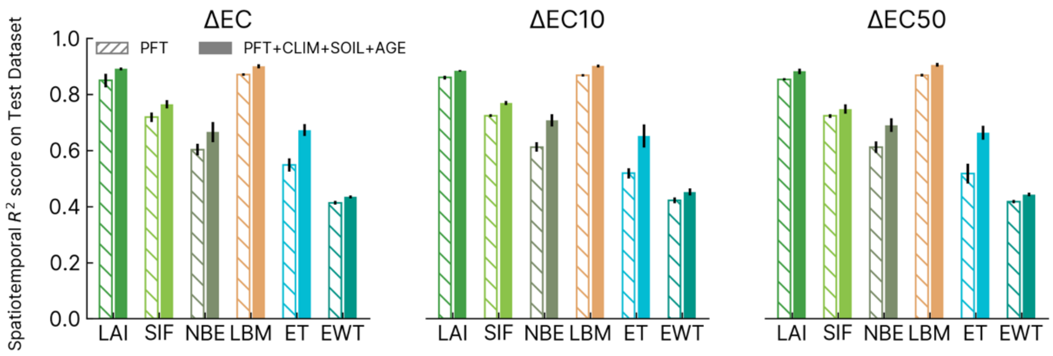

Fig. R5 (Fig. S34) | Sensitivity of the PFT and PFT+CLIM+SOIL+AGE configurations to different filtering thresholds for the spatial representativeness of eddy covariance data.

Eddy covariance data play a critical role in constraining the absolute magnitudes of ecosystem gross carbon uptake (GPP) and respiration (RECO) (Fig. S35). Without assimilation of eddy covariance observations (ΔEC), the DifferLand ensemble simulates a global GPP of 91.9 ± 27.8 PgC yr^{-1} and a global RECO of 88.2 ± 27.8 PgC yr^{-1} during 2003–2023. By contrast, under the default configuration with eddy covariance data assimilated, the model produces a global GPP of 136.7 ± 13.8 PgC yr^{-1} and RECO of 133.0 ± 13.6 PgC yr^{-1}. These values are consistent with independent benchmarks, including machine-learning–based upscaling of site-level GPP (e.g., FLUXCOM X-BASE, 125 ± 2.1 PgC yr^{-1}) and SIF-based global GPP estimates (135.5 ± 8.8 PgC yr^{-1}) (Xing Li & Xiao, 2019).

The largest discrepancies between ΔEC and the default runs occur in equatorial regions: with assimilation, GPP and RECO reach ~7 gC m^{-2} day^{-1}, compared to only ~4 gC m^{-2} day^{-1} without assimilation. Site-level evaluations in tropical forests confirm that omitting eddy covariance constraints leads to systematic underestimation of both fluxes. This outcome is expected because tropical forests

typically exhibit near-zero net carbon balance and weak seasonal cycle (e.g., Fig. S30d) despite high rates of photosynthesis and respiration; without point-based flux measurements, the model has little information to constrain the true magnitudes of these component processes. We note that differences among eddy covariance assimilation setups are relatively small for ET (Fig. S35), as its latitudinal gradient is already partially constrained by the assimilated GLEAM ET. Nonetheless, model runs assimilating site-level ET generally simulate lower values than those relying solely on gridded products.

Varying the filtering thresholds for site spatial representativeness had only a minor effect on simulated GPP and RECO, with global GPP estimates of 135.7 ± 8.2 PgC yr^{-1} under strict filtering (ΔEC50) and 139.6 ± 6.7 PgC yr^{-1} under more relaxed criteria (ΔEC10). This suggests that spatial mismatches between site footprints and model grid cells exert only a limited influence on global estimates. Nonetheless, future work should pursue more effective multiscale data-assimilation strategies and evaluate whether the inferred environment–trait relationships are consistent across canopy, ecosystem, and biome scales.

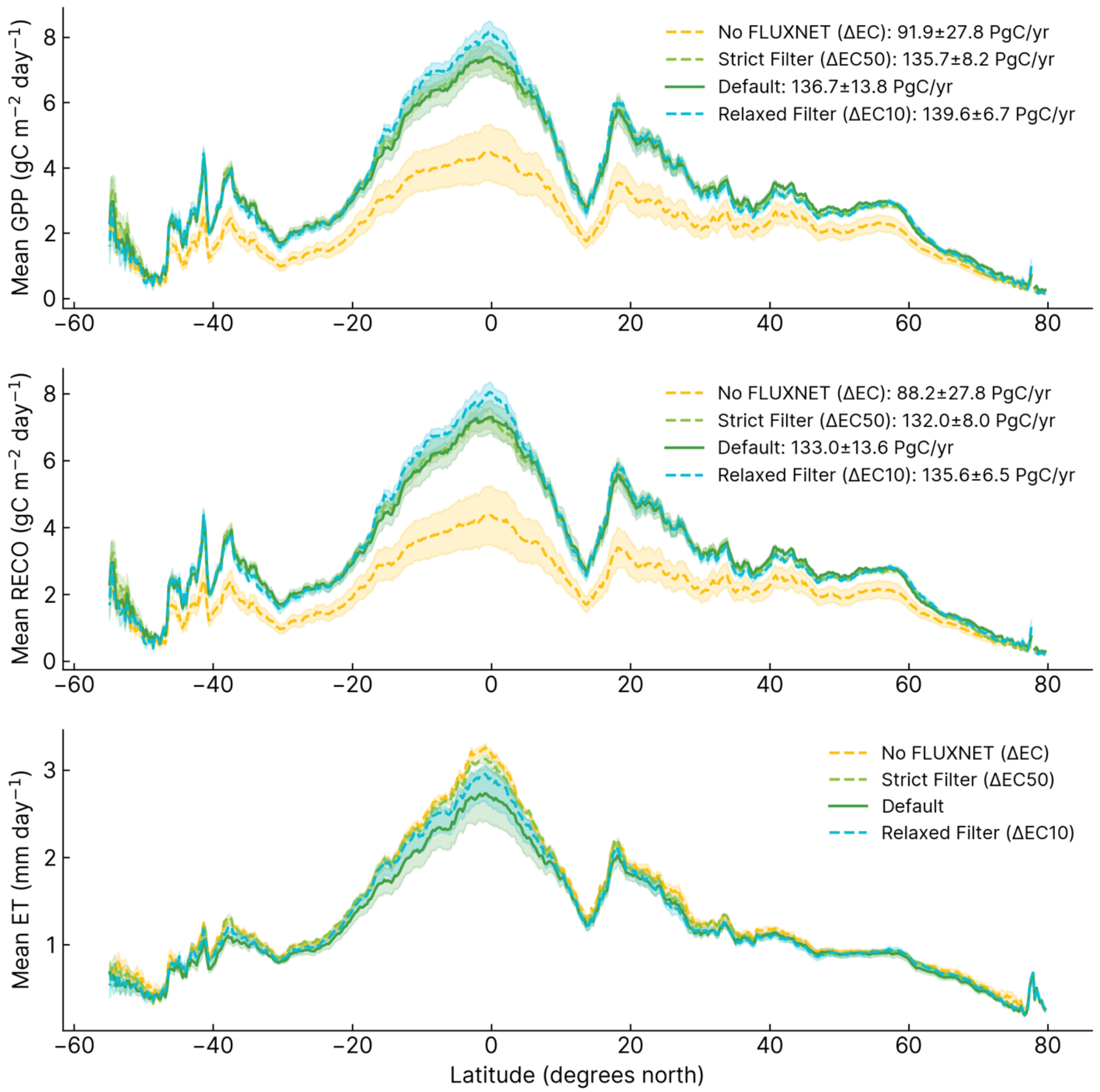

Fig. R6 (Fig. S35) | Latitudinal transects of mean GPP, RECO, and ET from model runs under four eddy covariance (EC) data assimilation configurations: no assimilation (ΔEC), a strict filtering criterion in which only sites whose reported plant type covers >50% of the overlapping grid cell are assimilated (ΔEC50, *N* = 123 sites), the default setup in which sites with >25% coverage are assimilated (*N* = 180 sites), and a relaxed criterion in which all sites with >10% coverage are assimilated (ΔEC10, *N* = 218 sites). Mean annual totals of GPP and RECO for each configuration are computed and labeled in the plots.

We have also made effort to improve the model performance in the tropics and Australia. In general, the lower performance is due to 1) a sign issue in fire carbon flux in our original implementation that affects

fire-prone ecosystems, which has been corrected in the revision; 2) significant data imbalance in the FLUXNET2015 dataset, which contains very few towers in the tropics and Australia after 2010. To counter this data imbalance, we first extended the simulation period from 2010-2023 used in the original manuscript to 2001-2023 to maximize overlap with as many eddy covariance towers as possible. Next, we gathered all publicly available eddy covariance observations (as of April 2025) from the FLUXNET2015, ICOS, Amerifux FLUXNET, LBA-ECO (Amazonian), OzFlux (Australian) datasets that substantially expand the number of observations available in the Southern Hemisphere. As the number of flux observations are limited in tropical ecosystems, we applied a rescaling in the loss term to weight factors to observations from each site that is inversely proportional to the number of site-months available for that specific plant type. This reweighting mitigates the data imbalance in eddy covariance observations across the tropical biomes to better constrain DifferLand's simulated carbon and water fluxes. For instance, we note a ~25% reduction in the RMSE for GPP and RECO in evergreen broadleaf forests after these modifications.

5. About parameter spatialization and Vvariance partitioning, please consider showing SHAP partitioning here to preface explainable AI section. Also, a comparison to traditional PFT-based TBM output would further quantify improvement.

Thank you for this helpful suggestion. We have revised the sequence of figures in the main text to establish a more natural flow of logic and to better preface the explainable AI section. Specifically:

- **Figure 2** now highlights the overall contributions of environmental predictors to simulated ecological fluxes and states, beyond the information captured by PFT-based predictors. The variance partitioning analysis further separates the independent contributions of plant functional type and environmental gradients, revealing that their interaction is most effective in explaining global variations in vegetation dynamics.
- To understand how multiple traits collectively respond to spatial predictors, we then present a **PCA-based analysis** that provides a holistic view of trait coordination. Here, SHAP analysis is used to quantify how the main axes of ecological organization are shaped jointly by environmental gradients and plant-type-specific attributes.
- Finally, we use **SHAP analysis in a more fine-grained manner** to examine the relationships between key spatial predictors (temperature, precipitation, and forest age) and several important ecological parameters (e.g., canopy photosynthetic efficiency, leaf carbon mass per area). We compare the trait–environment relationships obtained from the global CLIM+SOIL+AGE model with those derived from the PFT+CLIM+SOIL+AGE model conditioned on each plant type,

thereby showing how plant-type-specific effects modulate trait–environment relationships within vegetation classes.

This reorganization ensures that the results progress from overall model performance, to trait coordination, and then to detailed mechanistic interpretations. SHAP analysis is thus used consistently across multiple stages: first to highlight the coordinated responses of traits to environmental gradients, and then to dissect fine-scale trait–environment relationships both within and across plant types.

We further compared DifferLand simulated net biosphere exchange (NBE) with a set of DGVMs in the TRENDYv12 S3 ensemble. We found that DifferLand's simulated NBE dynamics after data assimilation better matches the CMS-Flux atmospheric inversion at both global and regional levels with lower RMSE compared with the majority of the DGVMs, and its ability to capture the decadal, interannual, and seasonal cycles of NBE is also competitive compared with most of the existing DGVMs. It should be noted that by making this comparison we are not necessarily claiming that DifferLand is structurally superior to the state-of-the-art DGVMs, as notably the model has a simplified structure and does not explicitly represent many key processes such as land use land cover change and nutrient limitations. Rather, such comparison indicates that on decadal timescale parameter uncertainty dominates the seasonal and interannual, and leveraging spatial trait-environment relationships embedded in observed carbon fluxes provides an effective approach to constrain such uncertainties. We included this comparison in the main text.

Despite its comparatively simplified process representation, DifferLand achieved significantly lower root mean squared errors and comparable correlation with the atmospheric inversion dataset than the much more structurally sophisticated dynamic global vegetation models (DGVMs) in the TRENDYv12 S3 ensemble (Fig. S8-12), both globally and regionally. These results suggest that uncertainties in model parameters are a major source of error in simulating land–atmosphere carbon fluxes at decadal timescales, and that hybrid modelling of trait–environment relationships offer a promising avenue to reducing these uncertainties.

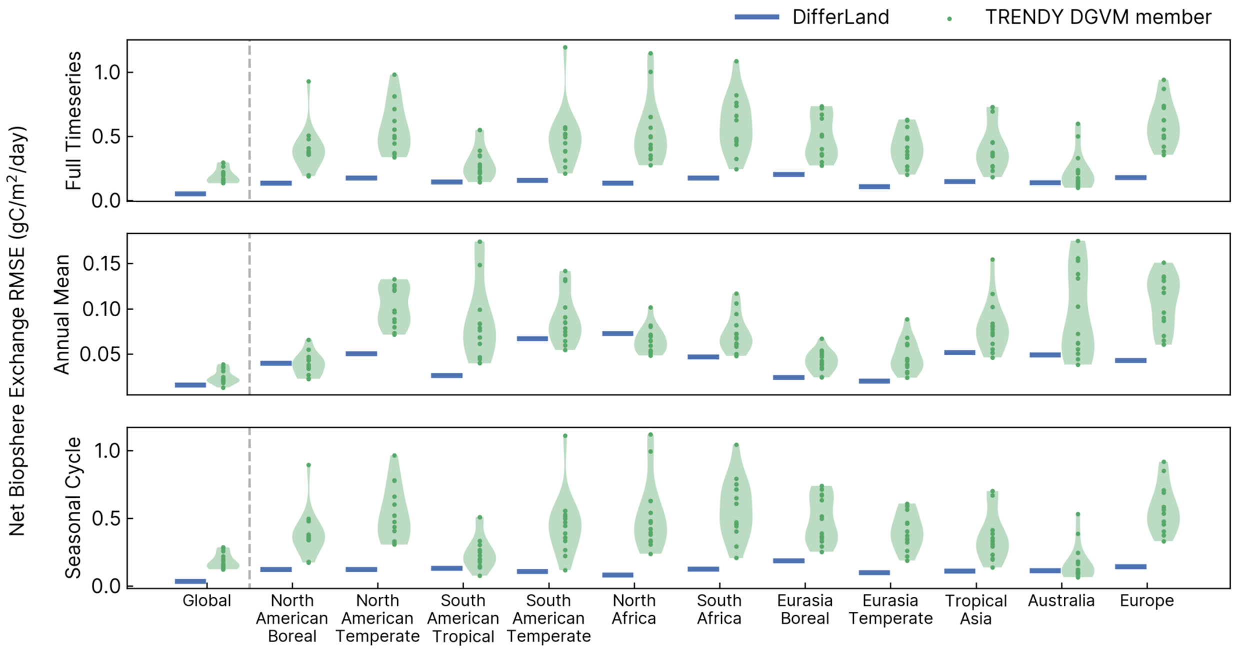

Fig. R7 (Fig. S8) | Comparison of net biosphere exchange (NBE) performance between DifferLand and TRENDY DGVMs across regions and temporal scales. Violin plots show the root mean square error (RMSE; gC m^{-2} day^{-1}) of NBE simulations compared to benchmark observations from the CMS-Flux atmospheric CO_2 inversion for the period 2010–2022. Errors are evaluated over: (top) the full time series, (middle) annual mean, and (bottom) seasonal cycle. Each green violin represents the distribution of RMSE from individual TRENDY dynamic global vegetation model (DGVM) members, while blue bars indicate the RMSE of DifferLand. Evaluations are conducted globally and across 11 TRANSCOM regions. Lower RMSE values indicate better agreement with the observational benchmark.

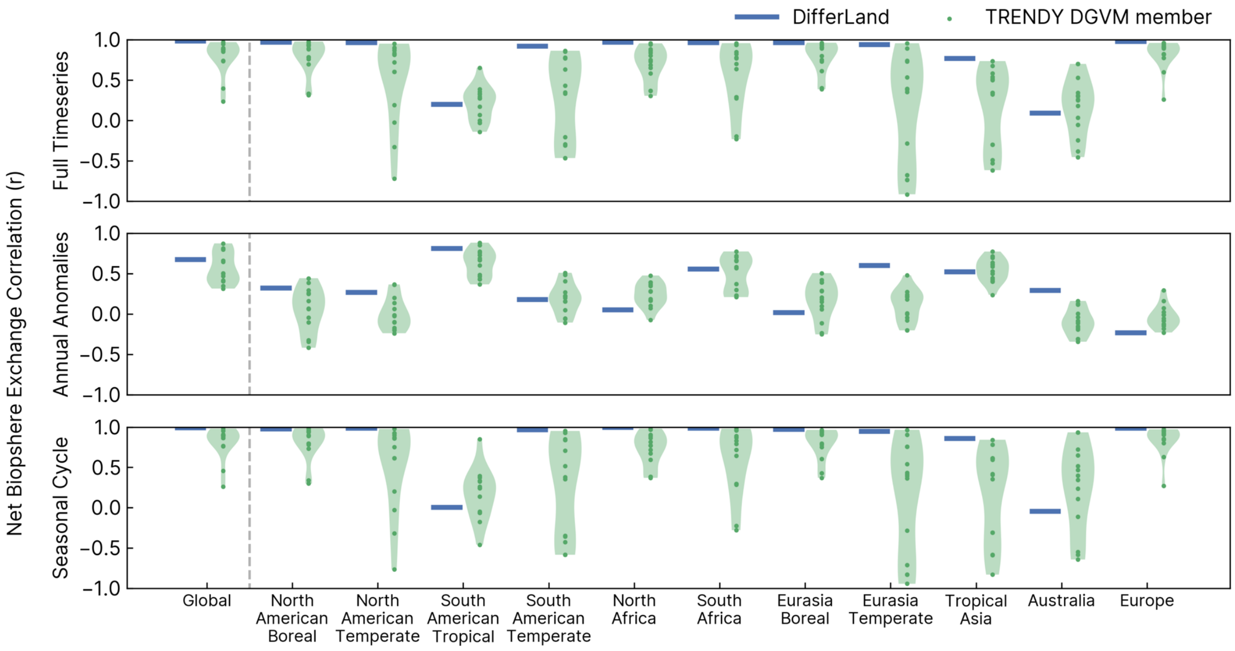

Fig. R8 (Fig. S9) | Comparison of net biosphere exchange (NBE) correlation between DifferLand and TRENDY DGVMs across regions and temporal scales. Violin plots show the Pearson correlation coefficient (r) between simulated and observed NBE from the CMS-Flux atmospheric CO_2 inversion over the period 2010–2022. Correlations are evaluated over: (top) the full time series, (middle) annual anomalies, and (bottom) seasonal cycles. Each green violin represents the distribution of correlations from individual TRENDY dynamic global vegetation model (DGVM) members, while blue bars indicate the correlation achieved by DifferLand. Evaluations are performed globally and across 11 TRANSCOM regions. Higher correlation values indicate better temporal agreement with the benchmark observations.

6. About SHAP analysis in environment–parameter relationships, please add interaction effects or pairwise SHAP interaction plots (e.g., temperature × age). Also, please consider regional SHAP breakdowns (e.g., different biome regimes). Further clarify how SHAP uncertainty is handled (e.g., through ensemble SDs).

We agree with the reviewer that SHAP interaction plots could in principle provide valuable insights into the interactions between environmental gradients (e.g., mean annual temperature) and biological information (e.g., forest demography). Indeed, our analysis suggests the interaction between biome specific effects and abiotic environmental gradients are crucial to explaining the variations in the observed vegetation dynamics (Fig. 2a). In practice, however, estimating such interaction effects directly

with SHAP for neural networks is both computationally prohibitive and prone to noise. This is because computing pairwise interactions require evaluating a quadratic number of combinations on top of the already exponential complexity of SHAP evaluations, and unlike TreeSHAP methods commonly used with ensemble models such as random forests or gradient boosting (e.g., XGBoost), SHAP values for neural networks must be approximated through sampling. These sampling-based approaches introduce substantial uncertainty, especially when attempting to isolate higher-order interactions. For this reason, rather than directly quantifying predictor interactions with SHAP, we rely on a variance partitioning framework to assess the contributions of different predictor groups to model performance on observable ecological fluxes and states, as shown in Fig. 2.

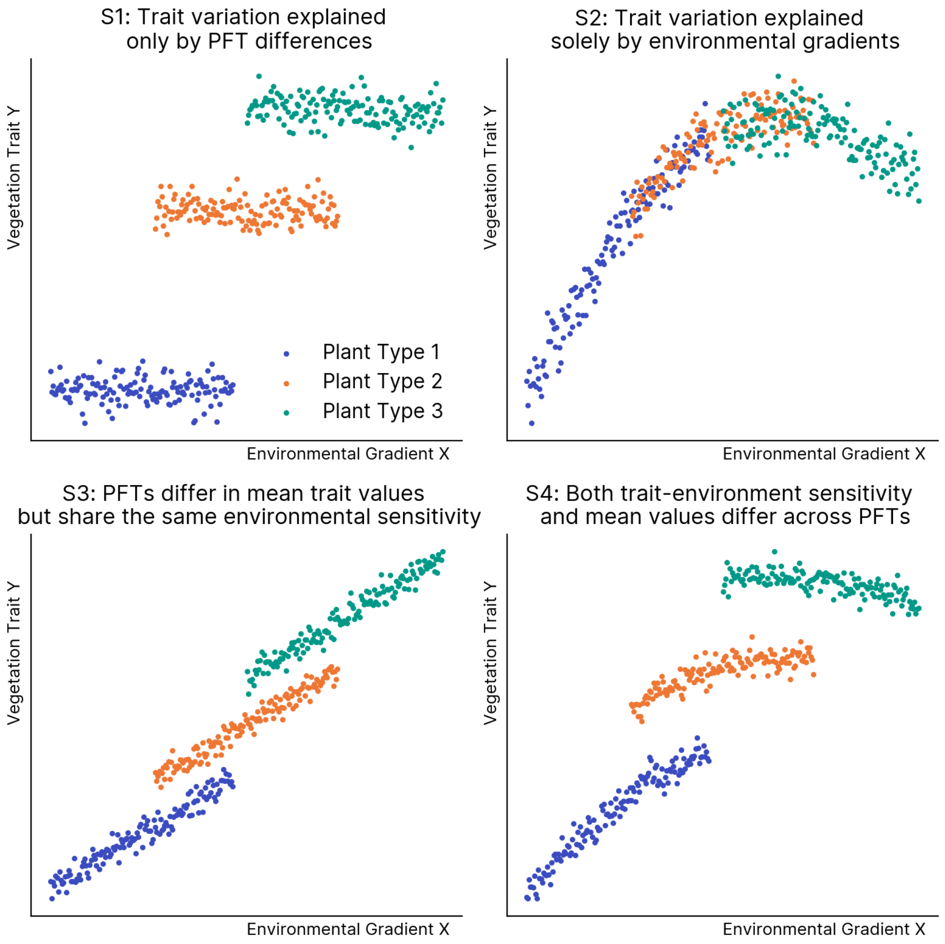

Fig. R9 (Fig. S1) | Conceptual illustration of four scenarios regarding the roles of plant functional types (PFTs) and environmental gradients in shaping the spatial distribution of plant functional traits. a) S1 – PFT-only control: Trait values vary between PFTs but remain largely constant within each PFT, showing minimal response to environmental gradients. b) S2 – Environment-only control: Trait values respond continuously to environmental gradients in a universal way, independent of PFT classification. c) S3 –

Consistent scaling across PFTs: Mean trait values differ among PFTs, but trait–environment relationships (i.e., the slopes) are similar across plant types. d) S4 – PFT-specific trait–environment relationships: Both mean trait values and the sensitivity of traits to environmental gradients differ among PFTs, resulting in distinct trait–environment relationships for each plant type.

However, we believe quantifying biome-specific SHAP effects address a key question in ecology regarding whether the trait-environment relationships are universal across all biomes, or whether differences across plant types dominate (see Fig. R9 for a conceptual illustration). We now included this comparison in the main text.

Line 530-593: To investigate the interaction between PFTs and environmental predictors, we compared functional relationships derived from model configurations with and without PFT predictors. The PFT-agnostic configuration (CLIM+SOIL+AGE) attempts to identify apparent global relationships between latent ecological parameters and spatial predictors, representing a hypothetical scenario where environment-trait relationships are uniform across plant types. Conversely, the PFT-aware model captures plant-type-specific dependencies between ecological parameters and environmental variables.

For a clearer extraction of environment-parameter relationships within each vegetation type, we restricted the SHAP analysis to pixels where a single plant type occupies at least 80% of the grid cell. Our analysis focused on four key ecological parameters that are robustly identifiable from global vegetation dynamics and exhibit distinct spatial patterns: photosynthetic efficiency, leaf carbon mass per area, carbon use efficiency, and root carbon allocation ratio. Among the environmental predictors, mean annual temperature, maximum canopy height, and—to a lesser extent—mean annual precipitation emerged as the most influential. We therefore examined their specific relationships with the selected ecological parameters (Fig. 4).

Mean annual temperature (MAP) exerts a dominant and often nonlinear influence on all four ecological parameters, indicating strong temperature regulation of ecosystem function consistent with the PC-level analysis. Photosynthetic efficiency rises with MAP from –15 °C to 15 °C before plateauing (Fig. 4a), while carbon use efficiency (NPP/GPP) declines from 0.45 to 0.35 and then stabilizes (Fig. 4g). Although global patterns are broadly mirrored within plant functional types (PFTs), notable differences exist in both mean values and temperature sensitivity. Grasslands and shrublands, spanning wide thermal ranges, generally exhibit higher photosynthetic efficiency (Fig. 4a), carbon use efficiency, (Fig. 4d) and root allocation ratios (Fig. 4j) than forests—traits reflecting acquisitive growth strategies in open habitats. In contrast, evergreen broadleaf forests show conservative strategies with low photosynthetic efficiency (Fig.

4a), high leaf carbon mass per area (Fig. 4d), and low carbon use efficiency (Fig. 4g), adapted to humid tropical conditions. Deciduous broadleaf and evergreen needleleaf forests exhibit sharp internal temperature sensitivity despite lower mean efficiencies. These PFT-specific patterns highlight that plant-type-specific responses can diverge from global trends, underscoring the need to account for ecological strategies in modeling trait–temperature relationships.

In contrast, the apparent global sensitivity of the four ecological parameters to mean annual precipitation largely reflects differences among plant functional types (PFTs) distributed along the precipitation gradient, rather than variations within PFTs. For example, grasslands and shrublands—typically characterized by higher photosynthetic efficiency—are more common in drier regions than forests, resulting in an apparent global decline in photosynthetic efficiency with increasing precipitation (Fig. 4b). However, within-PFT variation in precipitation is generally small. For carbon use efficiency (Fig. 4h) and root carbon allocation ratio, we still observe negative relationships with precipitation within most PFTs, consistent with global trends, though the within-PFT sensitivities are substantially smaller than the differences across PFTs. The divergence between global and within-PFT patterns is most pronounced in the relationship between maximum canopy height and photosynthetic capacity: while the global negative relationship is driven by differences in photosynthetic efficiency across PFTs (Fig. 4c), the within-PFT relationship is positive, suggesting that regions with greater resource availability support both higher canopy stature and photosynthetic potential. These contrasting patterns underscore the complementary roles of plant type and environmental gradients in shaping ecological parameter variation, reflecting distinct ecological processes operating at different organizational scales.

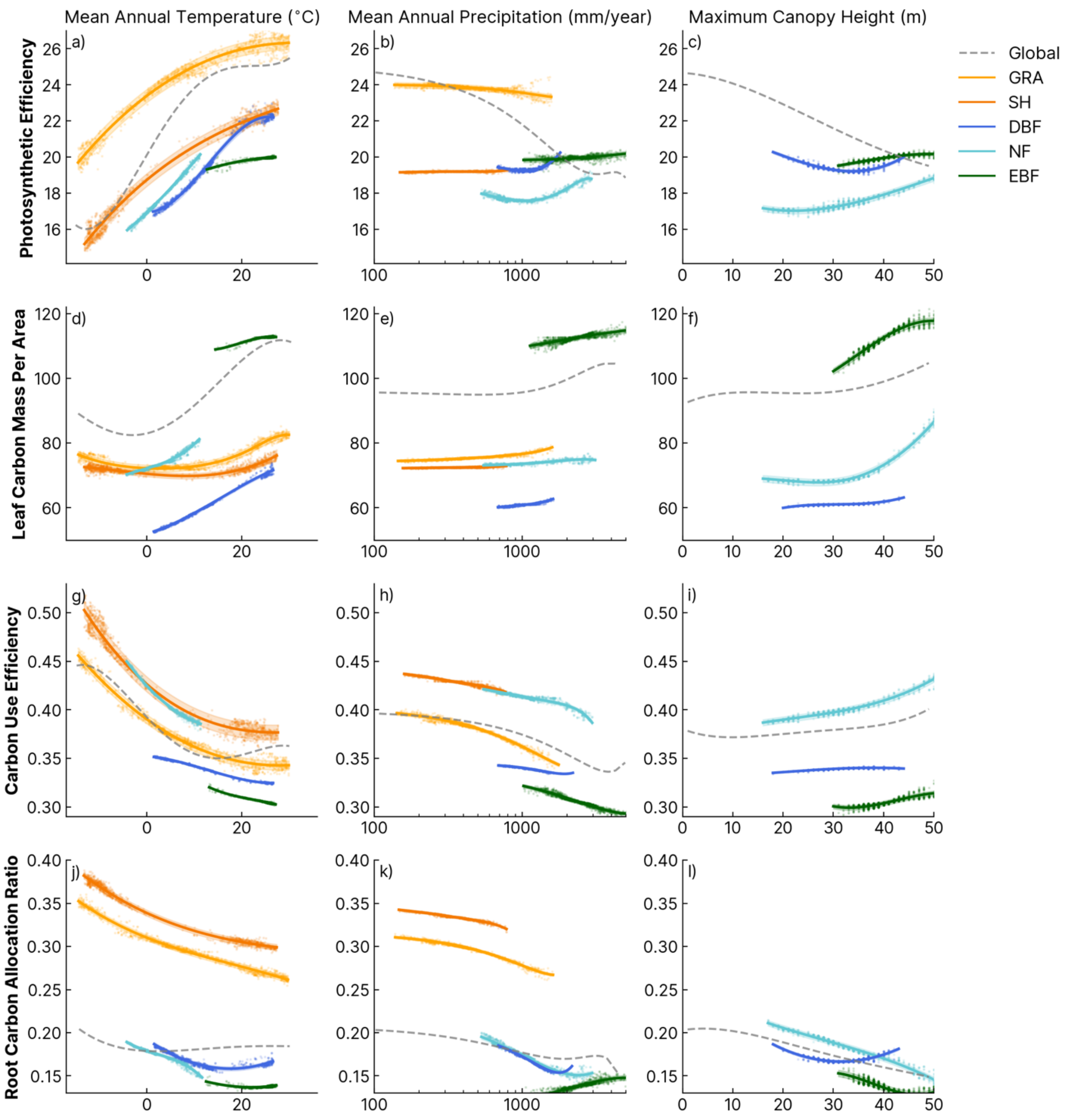

Fig. 4| Comparison of global and PFT-specific trait–environment relationships. Panels show how nitrogen-limited photosynthetic efficiency (gC m^{-2} leaf day^{-2}), leaf carbon mass per area (gC m^{-2} leaf), carbon use efficiency (unitless, 0–1), and fine root carbon allocation ratio (unitless, 0–1) vary with mean annual temperature, mean annual precipitation, and maximum canopy height. The global relationships (dashed lines) represent ensemble-mean SHAP-derived dependencies from the CLIM+SOIL+AGE model configuration. PFT-specific relationships are derived from the PFT+CLIM+SOIL+AGE model configuration, using samples where the specified PFT comprises at least 80% of the pixel. PFT

abbreviations: GRA – grassland; SH – shrubland; DBF – deciduous broadleaf forest; NF – needleleaf forest; EBF – evergreen broadleaf forest.

We further enriched the methods section to detail how ensemble-averaged SHAP values are calculated to reduce random uncertainties in individual model members.

Line 1223-1250:

SHAP Analysis for Feature Importance & Environment-Parameter Relationships

We applied kernel SHapley Additive exPlanations (SHAP) to extract the learned environment-parameter relationship from the spatialization neural network for a selection of latent ecological parameters(Lundberg & Lee, 2017). Kernel SHAP estimates Shapley values, informed by cooperative game theory, to quantify each feature's marginal and additive contribution to the model's output. To ensure robustness of SHAP results and reduce the random uncertainties associated with individual model members, we calculated ensemble-based SHAP by first defining an ensemble-averaged model M_{ens} from 10 out of 20 model members for each configuration that best converges on the training dataset.

$$M_{ens} = \frac{1}{10}\sum_{i=1}^{10} M(x, \theta_i)$$

Where M is the spatialization network, x denotes the spatial predictions, and θ_i are the neural network weights and biases of member i within the ensemble. From M_{ens} we sampled 100 grid cells to compute the background distribution, and then computed the SHAP values from 1,000 randomly selected grid cells within the training dataset. Feature importance of different predictors was determined by ranking the mean absolute SHAP values from the 1,000 samples. This procedure is repeated for each selected parameter to obtain global trait-environment relationships. To derive plant-type-specific SHAP results, we further computed conditional SHAP values by sampling from grid cells where a specified plant functional type constitutes at least 80% of the area. For SHAP analysis on the principal components, we linearly projected M_{ens} into the principal component space using the PCA loadings and computed the SHAP analysis within this projected space.

7. About trait coordination via PCA, please link PCA results more explicitly to SHAP-derived patterns. Also, explore whether PCA axes could guide future trait-based model clustering.

Thanks a lot for this helpful advice. In the revised manuscript, we conducted SHAP analysis on the principal components to understand how environmental gradients and plant type information together shape the coordination of plant traits underlying the observed vegetation dynamics.

Line 393-439:

DifferLand's ability to robustly predict vegetation dynamics across space by leveraging environment–parameter relationships suggests that global vegetation patterns may give rise to convergent plant functional traits, which in turn enable spatial predictability of the terrestrial carbon cycle. To investigate this hypothesis, we conducted a principal component analysis (PCA) on 13 ecologically meaningful and spatially identifiable parameters (see "Parameter Identifiability Analysis" in Methods and Text S6) retrieved from the model using the full set of spatial predictors to identify covarying traits and the primary axes of spatial variability (Fig. 3a). To further examine how environmental drivers influence the covariation among ecological parameters, we applied Shapley Additive exPlanations (SHAP) to the ecological axes obtained by projecting the predicted parameters onto the principal components (PCs) using the loadings from the previous analysis (Fig. 3e-g). We assessed the overall importance of each spatial predictor by calculating its mean absolute SHAP value, and quantified the strength and directionality of its influence using the difference in SHAP values between the upper (Q3) and lower (Q1) quantiles. This approach retains the non-linear trait–environment relationships captured by the spatialization network while simplifying the analysis of parameter covariation through a linear transformation of the ecological space.

The first principal component (PC1), explaining 53.2% of the variance, reflects the well-established leaf economics spectrum, distinguishing between plants that invest in long-lived leaves with high leaf carbon mass per area (LCMA) and those with shorter-lived leaves characterized by lower carbon investment and greater allocation to labile carbohydrates supporting seasonal leaf onset (Fig. 3b). The second component (PC2), explaining 22.1% of the variance (Fig. 3a,c), captures the tall–short vegetation gradient (Fig. 3g): short, often herbaceous vegetation exhibits high photosynthetic capacity and rapid litter turnover, whereas taller vegetation tends to have lower photosynthetic efficiency and allocates more carbon to woody biomass. These two ecological axes are well documented in trait databases at the species and community levels, but here we show they also emerge from satellite-observed vegetation dynamics without imposing explicit trait–environment relationships. Interestingly, a third component (PC3), explaining 10.5% of the variance (Fig. 3b), highlights regions with high photosynthetic efficiency and intensive cropland use (e.g., the U.S. Midwest, Southern Europe, and Northeastern China), potentially reflecting the artificial selection of high-yield cultivars or the prevalence of photosynthetically efficient C4 crops such as maize.

Mean annual temperature emerged as the primary predictor for all three principal components (PCs; Fig. 3e–g), reflecting a first-order energy control on plant functional traits. In warmer climates, plants tend to exhibit traits that support longer growing seasons, higher photosynthetic efficiency, greater allocation to

woody biomass, and taller canopy height—strategies advantageous for light competition and perennial growth. In contrast, plants in colder environments adopt traits such as shorter stature, reduced leaf lifespan, and increased carbon allocation to belowground biomass. Maximum canopy height, a proxy for growth potential, is most strongly associated with PC2. Despite the dominant role of climate, plant-type predictors also play a key role in shaping the ecological axes. Although a model configuration using only environmental predictors (CLIM+SOIL+AGE) can broadly reproduce the spatial patterns of PC1 and PC2, the inclusion of plant functional type (PFT) information—particularly cropland extent—is essential to capture the spatial variability related to managed crop productivity represented in PC3. These findings suggest that while the covariation of ecological parameters primarily reflects macroecological environmental gradients, it also bears the imprint of present-day plant type distributions and land-use legacies.

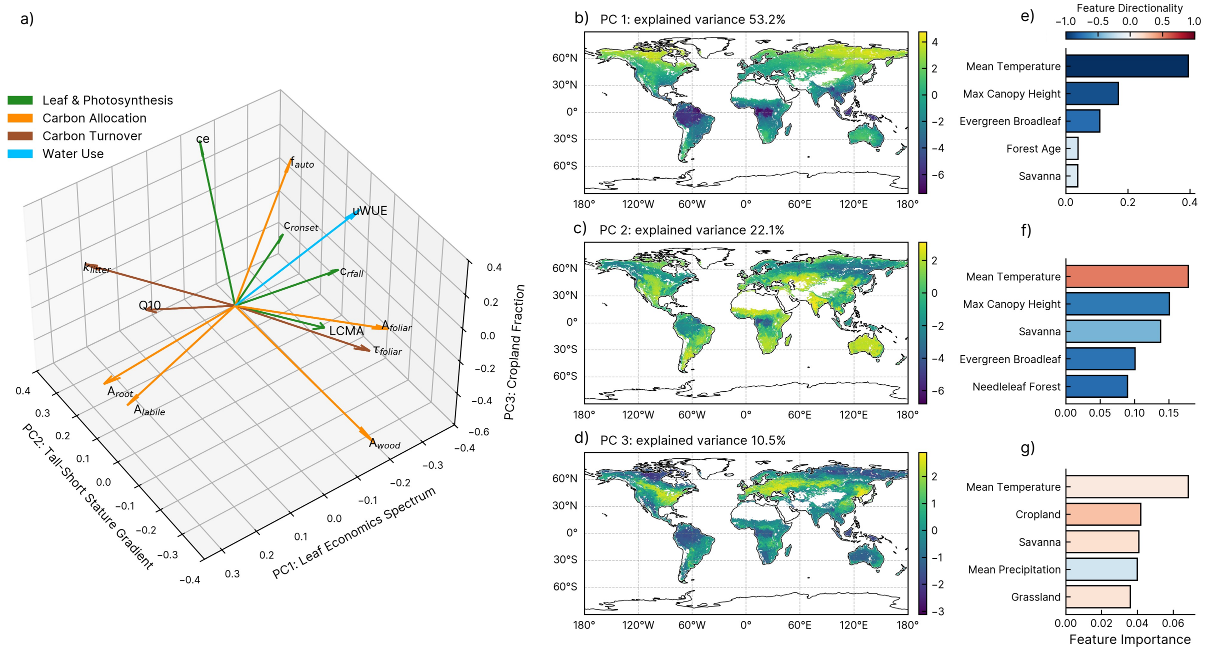

Fig. 3 | Coordination of latent ecological functional traits along three principal axes. a) Principal component analysis (PCA) of 13 latent ecological parameters inferred from the ensemble mean of the PFT+CLIM+SOIL+AGE model configuration. The loadings that project each parameter onto the first three PCs are plotted in the 3D plot. The 13 parameters include canopy photosynthetic efficiency (ce), underlying water use efficiency (uWUE), temperature sensitivity of heterotrophic respiration (Q10), autotrophic respiration fraction (f_{auto}), litter turnover (k_{litter}), leaf carbon mass per area (LCMA), leaf onset (c_{ronset}) and fall duration (c_{rfall}), leaf lifespan (τ_{foliar}), and allocation fraction of GPP to labile (f_{labile}), foliar (A_{foliar}), wood (f_{wood}), and fine roots (f_{root}) pools; b-d) plot the spatial maps of the top three PC scores. Parameters are grouped into four categories: leaf & photosynthesis (green), carbon allocation (orange),

carbon turnover (brown), and water use (blue). The three principal axes are interpreted as: PC1 – *Leaf Economics Spectrum*, PC2 – *Tall–Short Stature Gradient*, and PC3 – *Cropland Fraction*. b–d) Spatial distribution of PC1–PC3 scores projected from the spatialization neural network onto the PCA axes, with the proportion of variance explained by each component labeled. e–g) SHAP-based feature attribution for each principal component, showing the influence of spatial predictors. Bars represent mean absolute SHAP values (feature importance), while colors indicate the direction and strength of association, calculated as the median SHAP difference between the upper (Q3) and lower (Q1) quartiles of each predictor.

8. In discussion, add more on SHAP as a tool for process discovery (e.g., revealing unknown thresholds, nonlinearities), and could elaborate on how DifferLand could help forecast climate-driven shifts in ecological function.

Thanks a lot for this great suggestion. In the revised manuscript, we added a new section on how DifferLand could help infer potential climate-driven shifts in ecological functions. In particular, we highlight the importance of plant-type specific environment-trait relationships in modulating future changes in ecological functions, as plant-type specific effects can either act as a form of ecological legacy that buffer potential changes in ecological parameters or amplify ecological shifts if there is a strong local sensitivity to environmental gradient in the context of climate change.

Line 628-759:

Implications for Ecological Shifts Under Changing Climate

The strong sensitivity of key ecological parameters to climatological gradients—particularly temperature—raises the question of whether spatial dependencies can inform potential shifts in ecological functioning under ongoing changes in mean climate. Assuming, as a theoretical extrapolation, that spatial-environmental relationships approximate temporal responses (see limitations of this assumption in the Discussions), whether ecological traits vary continuously along environmental gradients or reflect categorical differences among plant types has major implications for predicting terrestrial carbon and water dynamics in a changing climate. If ecological parameters within a given PFT show stronger sensitivity to temperature or precipitation than the global average, then ecosystems dominated by that PFT may exhibit amplified functional responses to similar climate shifts. Conversely, if ecological function is largely governed by PFT identity, with limited variation within types, then substantial functional changes would require shifts in vegetation composition—transitions that are often constrained by dispersal limits, recruitment bottlenecks, and potential ecological tipping points (e.g., a shift from tropical forest to savanna in the Amazon). While current PFT distributions reflect adaptation to past

climate regimes over evolutionary timescales, they may act as ecological legacies that buffer against rapid climate-driven functional change on multi-decadal timescales relevant to contemporary climate change.

As an extrapolation experiment, we estimated theoretical changes in key ecological functional parameters using projected mean temperature and precipitation for 2081–2099 under the "Middle of the Road" SSP2-4.5 scenario (O'Neill et al., 2016) averaged across CMIP6 ensemble members (Table S5). These projections were evaluated using latent environment–parameter relationships derived from either a plant-type-agnostic model (CLIM+SOIL+AGE) or a plant-type-aware model (PFT+CLIM+SOIL+AGE) (Fig. S2). Under the plant-type-aware configuration, photosynthetic efficiency is projected to increase globally, with the largest gains occurring in boreal shrublands and temperate forests of the Northern Hemisphere due to both higher inferred sensitivity of photosynthetic efficiency to climatological temperature gradients in cooler regions and greater projected warming (Fig. S2a). However, incorporating plant-type-specific responses dampens the magnitude of this increase: the projected gain in photosynthetic efficiency is reduced by 0.5–1.75 gC m^{-2} leaf day^{-1} (about half of the magnitude) compared to estimates assuming a uniform global temperature dependency (Fig. S2b). Similarly, leaf carbon mass per area (LCMA) is projected to increase by 3-4 gC m^{-2} in Northern Hemisphere temperate regions under the plant-type-agnostic model (Fig. S2b), but this increase is reduced by approximately 1-2 gC m^{-2} when accounting for plant-type-specific effects (Fig. S2f). These results suggest that plant-type-specific constraints may reduce the magnitude of future increases in plant carbon uptake.

In contrast, both carbon use efficiency (Fig. S2c) and the root carbon allocation fraction (Fig. S2d) are projected to decline under the SSP2-4.5 scenario, leading to a lower NPP:GPP ratio and a potential shift in internal carbon allocation within plants across the Northern Hemisphere. While the projected decline in carbon use efficiency is similar under both the globally uniform and plant-type-aware relationships (Fig. S2g), plant-type-specific effects may amplify the reduction in root allocation (Fig. S2h)—resulting in a more pronounced decrease in the fraction of carbon allocated to fine roots, particularly in boreal shrublands and tundra ecosystems. These differences underscore the importance of accounting for functional diversity when projecting ecosystem responses to climate change. Nevertheless, we emphasize that these theoretical projections rely on a space-for-time substitution, assuming that current trait–environment relationships—reflecting equilibrium adaptations to climatological gradients—remain valid under future climate conditions. Whether such relationships will hold under anthropogenic climate change remains an open question, warranting further investigation through longitudinal studies and experimental evidence.

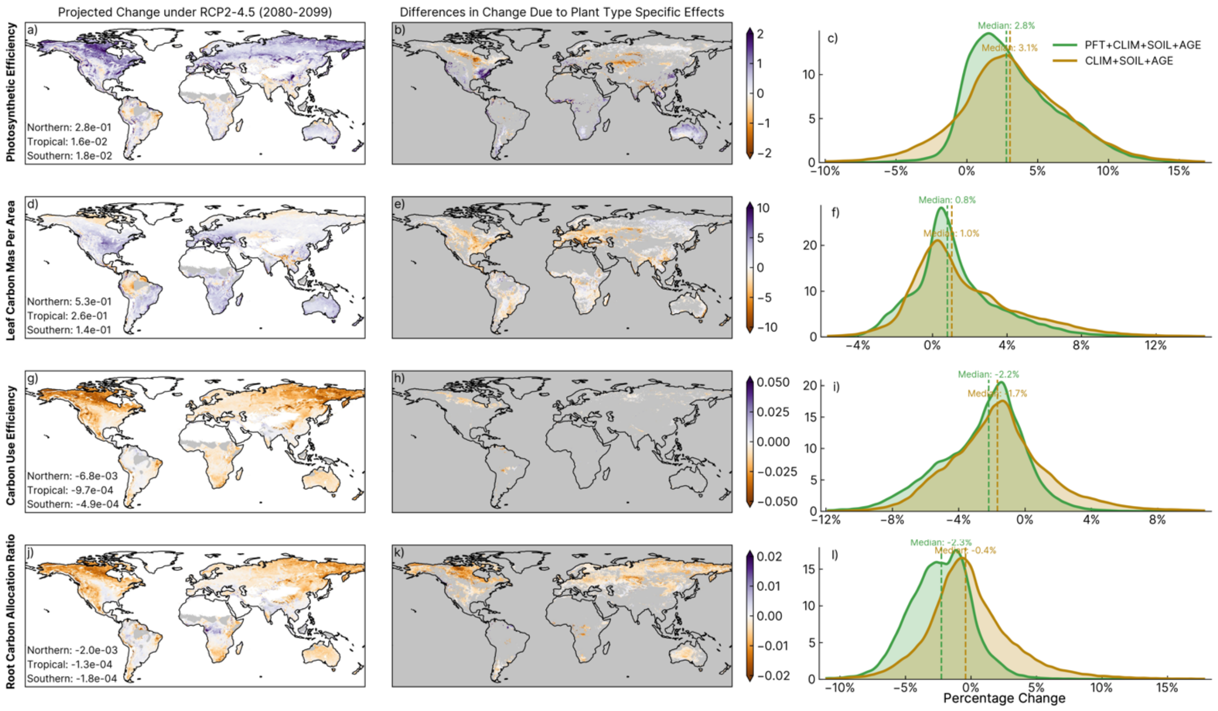

Fig. S2 | Projected changes in key ecological parameters under future climate scenarios. a–d) Projected changes in nitrogen-limited photosynthetic efficiency, leaf carbon mass per area, carbon use efficiency, and root carbon allocation ratio for the period 2080–2099 under the CMIP6 RCP2.4–5 scenario. Projections are made by applying learned trait–environment relationships—derived from the PFT+CLIM+SOIL+AGE model configuration—to projected changes in mean annual temperature and precipitation, assuming a space-for-time substitution. Regions where future climate conditions exceed the 99th percentile of the current climatological range are masked out. Mean changes across northern, tropical, and southern latitudinal bands are summarized in each panel. e–h) Differences in projected changes between the PFT+CLIM+SOIL+AGE and CLIM+SOIL+AGE model configurations, highlighting the effect of plant functional type–specific trait responses. Each configuration uses an ensemble of 10 model members. Only statistically significant differences ($p < 0.05$), determined via permutation tests, are shown; nonsignificant regions are masked out.

9. In Figure S26 (SHAP contributions) could be expanded with group-wise or biome-level SHAP summaries. Please consider including a worked example pixel to show how environment → parameter → prediction → observation alignment works.

We examined the biome-level SHAP relationships in Fig. 3. To illustrate the environment → parameter → prediction → observation alignment, we included a worked pixel in Finland to showcase the inner mechanism of the DifferLand framework.

Line 944-948: "We present a worked example from a site in Finland illustrating the inner mechanism of the DifferLand framework: the spatialization network maps environmental predictors into ecological parameters, which then parameterize the DALEC model to simulate trajectories of ecological states and fluxes under monthly meteorological forcing (Fig. S4)."

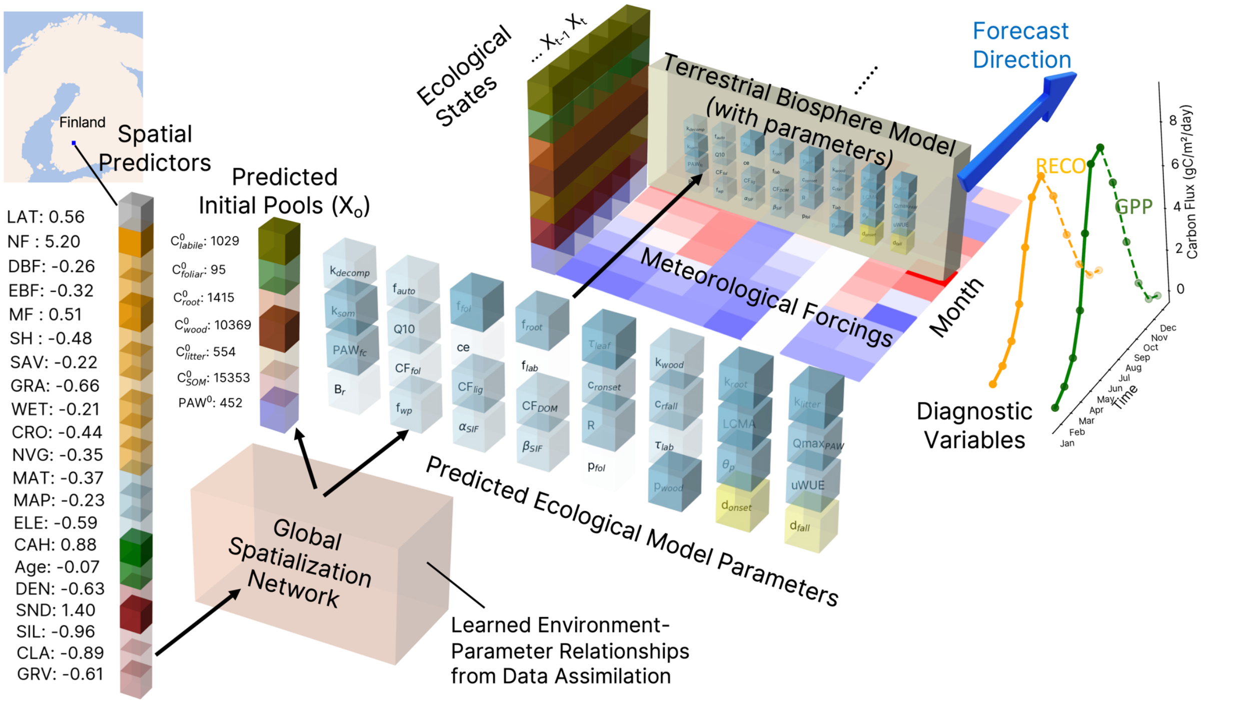

Fig. S4 | Example of the internal data flow through DifferLand for a sample grid cell in central Finland. A vector of standardized spatial predictors—including PFT fractions, climatological variables, forest age, maximum canopy height, and soil texture properties—is fed into the spatialization neural network to predict both the initial ecosystem states at the start of the simulation and the ecological parameters specific to the grid cell. These predicted ecological parameters are then used to parameterize the differentiable terrestrial biosphere model. The model's differential equations are integrated forward in time, driven by monthly meteorological forcings, to update ecosystem states and simulate monthly carbon and water exchanges within the grid cell. This process is repeated at each grid cell across the global land surface.

Reviewer #1 (Remarks on code availability):

After briefly reviewing in the core code (DALEC993.py), the provided code for the DifferLand framework demonstrates a well-structured and technically sound implementation of a differentiable land surface model using JAX. It integrates neural networks with process-based ecological modeling in a novel and flexible way, allowing for parameter inference and global trait estimation with physical consistency.

The core simulation logic is robust, modular, and clearly aligned with the scientific goals of the manuscript. However, while the underlying code quality is strong, the overall reproducibility and usability of the code are currently limited by the absence of essential supporting materials. Specifically, the code lacks a README file, installation or usage instructions, defined dependencies, and example workflows or pretrained models. As such, the results of the paper are only partially reproducible at this stage, and external users would face significant challenges in deploying the model without additional guidance. Nonetheless, with modest improvements to documentation and packaging, this code has high potential to become a valuable resource for the Earth system modeling and machine learning communities.
We thank the reviewer for the constructive feedback on our code base. In response, we have updated our repository to include all necessary data, installation instructions, and configurations to reproduce the results presented in the manuscript. We have also added comprehensive documentation (to be hosted on GitHub Pages upon release, with a PDF version provided as a supporting document) that describes the package API and offers notebook-based tutorials demonstrating example workflows, including how to extend the model with custom ecological modules. In addition, we have refactored the main package to adopt a more modular and reusable design, and we have incorporated comments and docstrings throughout the code where appropriate. We hope these improvements will enhance the usability of the package and support its adoption within the Earth system modeling and machine learning communities.

Reviewer #2 (Remarks to the Author):

This study presents DifferLand, an AI-enabled TBM designed to evaluate the impact of spatially-varying parameters on global carbon and water flux simulations. The main findings are: 1) spatially discrete and categorical PFTs provide limited explanation for spatial parameter variability, 2) local climate and forest demography offer greater explanatory power, and 3) the key drivers of spatial parameter variability are growing season length, leaf economics, and agricultural intensity. DifferLand represents an innovative integration of physical processes and AI. While the study's motivation is clearly articulated and logically structured, several major concerns the authors should thoroughly address:
We sincerely appreciate the reviewer's thoughtful and constructive comments, which have greatly helped us improve the quality and clarity of our work. We have thoroughly revised the manuscript to address the concerns raised and have provided detailed clarifications where necessary. Attached please find our point-to-point response to each comment raised by the reviewer.

1.The baseline: The current baseline definition is misleading, as it does not accurately quantify the impact of omitting spatially varying parameters. In existing TBMs, parameters are constant within a PFT but vary across PFTs. Therefore, this configuration should serve as the baseline, corresponding to the PFT only setup in this paper. Then the spatially-varying parameter (even within each PFT) setup should come only from CLIM, SOIL, and AGE, which are not explicitly considered in current TBMs. An incorrect baseline could result in misleading conclusions.

We thank the reviewer for this valuable comment. We agree that, in most existing TBMs, parameters are constant within a PFT but vary across PFTs. Therefore, the PFT-only configuration is the most appropriate baseline when evaluating the additional spatial explanatory power provided by CLIM, SOIL, and AGE variables. In the revised manuscript, we have explicitly adopted the PFT-only configuration as the baseline for all relevant comparisons and have clarified this definition throughout the text.

For example, in the revised Figure 2a, we normalize the mean absolute error (MAE) of the CLIM+SOIL+AGE and PFT+CLIM+SOIL+AGE configurations relative to the PFT-only configuration (normalized to 1). This representation more clearly illustrates the added value of incorporating environmental predictors. The results confirm that PFTs alone cannot fully capture the spatial variability in ecological parameters needed to explain the observed global vegetation dynamics, particularly for evapotranspiration. At the same time, environmental variables alone are insufficient to capture spatial variations in latent ecological traits—especially for LAI and SIF. Instead, the comparison highlights that interactions between plant types and environmental variables are essential for explaining variations in vegetation growth and carbon cycling, while water cycle dynamics are more strongly governed by environmental gradients and meteorological forcing. Subsequent hierarchical partition (Fig. 2 b-f) further allows us to isolate the *independent* contributions of PFT information and CLIM, AGE, and SOIL variables. Here, the contributions of the environmental variables quantify regions where these variables provide additional information of within-PFT parameter variations.

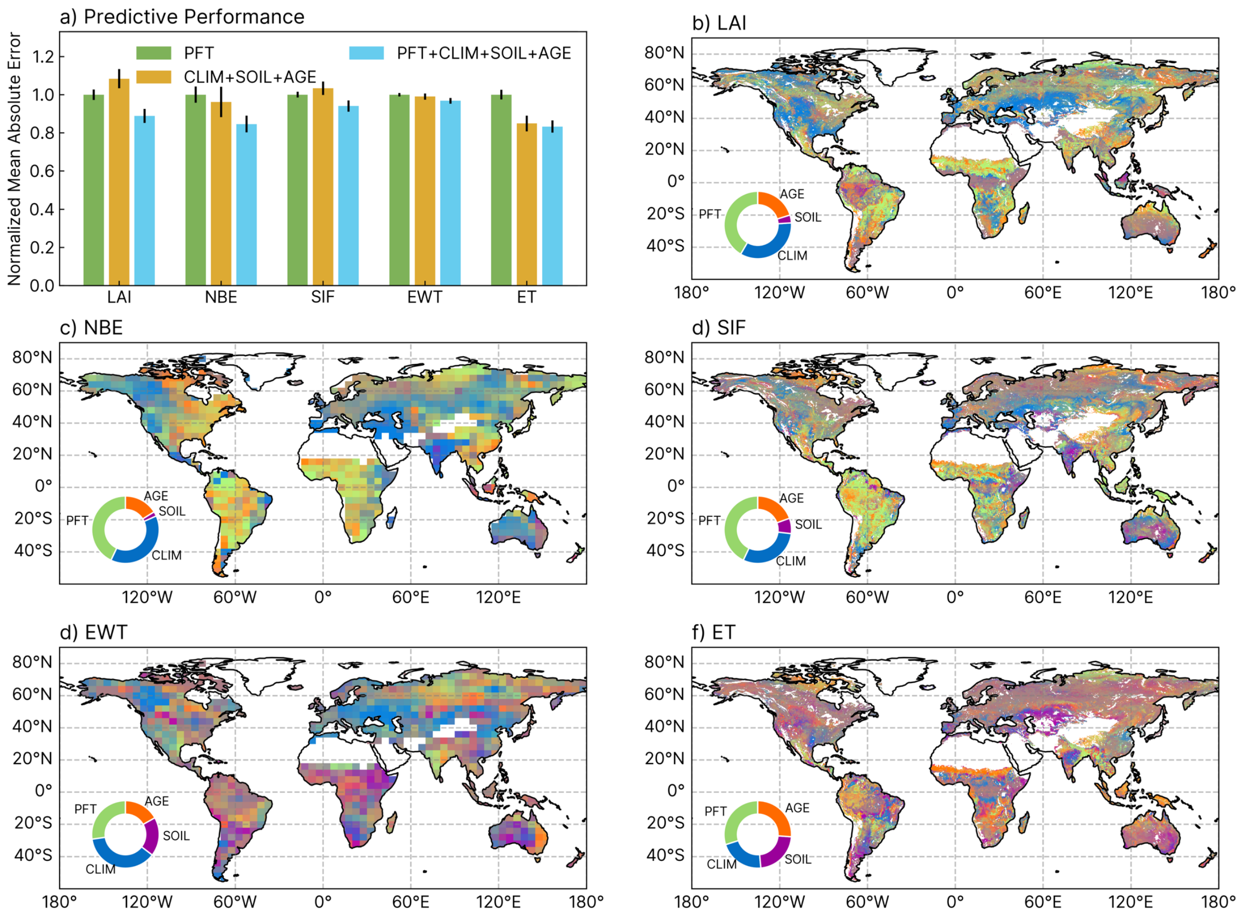

Fig. 2 | Hierarchical partitioning of explained variance by four predictors groups. Panel a) compares the mean absolute error on held-out pixels for LAI, NBE, SIF, EWT, and ET across three model configurations: PFT-only, environmental variables only (CLIM+SOIL+AGE), and combined PFT and environmental variables (PFT+CLIM+SOIL+AGE). Errors are normalized relative to the PFT-only configuration. Panels b-f) show the proportion of temporal variance explained in LAI, NBE, SIF, EWT, and ET within each grid cell, attributed to the model using ecological parameters predicted from spatial information in PFT, CLIM, AGE, and SOIL variables, respectively. The inset shows the proportion of pixels where each predictor group (PFT or environmental variables) has the dominant effect.

We thoroughly revised our manuscript to ensure a consistent comparison with the PFT-only baseline.

Line 147-155: “To evaluate whether trait–environment relationships provide independent information for predicting global ecological dynamics beyond what is captured by PFT-based categories, we trained models using either only PFT fractions as spatial predictors or a combination of PFT fractions and environmental predictors reflecting climatology, forest age and growth potential, and soil properties. Note

that in both cases, all tunable ecological parameters in the process-based model, as well as the initial values of the carbon and water pools, are optimized against observations using the same meteorological forcings via the spatialization network. Thus, the performance differences reflect the information content of the spatial predictors rather than differences in default parameter choices, model initialization, or structural assumptions."

Line 165-194: "Experiment results demonstrate that environmental gradients provide essential spatial information beyond plant functional types (PFTs) for predicting vegetation trait distributions. When incorporating all environmental predictors (PFTs, climate variables, forest age, and soil properties), DifferLand effectively captures the spatial and temporal patterns of both *in-situ* and remotely sensed observations of vegetation dynamics from 2001 to 2023 (Fig. S15 & Fig. S16). It generalizes well to held-out pixels, achieving a total spatiotemporal R^2 values of 0.88± 0.01 for LAI, 0.76± 0.01 for SIF, 0.71± 0.03 for NBE, 0.68± 0.02 for ET, and 0.45± 0.01 for EWT. The model also accurately captures the mean global biomass (Fig. S22a) and reasonably reproduces the assimilated trends in biomass over the past two decades (Fig. S22b). Notably, the model demonstrates minor differences in predictive performance between the training and test pixels, suggesting the model generalized well at unseen pixels (Fig. S15 & Fig. S16). In contrast, the baseline model relying solely on PFT fractions yields substantially lower total R^2 scores of 0.86 ±0.01, 0.73± 0.01, 0.60±0.03, 0.52±0.02, and 0.42±0.01 for LAI, SIF, NBE, and ET, and EWT respectively, when evaluated over held-out pixels, and exhibits a total spatiotemporal mean absolute error (MAE) that are 3–20% higher than those of the full environmental predictor configuration (Fig. 2a). These findings indicate PFTs alone do not fully capture the spatial variability in ecological parameters required to explain the observed global vegetation dynamics recorded by satellite and atmospheric inversion data.

However, the results also indicate that environmental variables alone are insufficient to capture spatial variations in latent ecological traits represented by the model parameters. While using only climatology, soil, and age predictors reduces MAE for ET predictions by 15% compared to the PFT-only baseline, plant-type-specific information remains crucial for explaining variability in canopy structure, as reflected in LAI, and in photosynthetic activity, as measured by SIF (Fig. 2a). When both PFT fractions and environmental variables are used as predictors, we observe larger reduction in errors compared to using either PFTs or environmental variables alone (Fig. 2a). For EWT, changes are relatively minor regardless of the choice of spatial predictors, likely because total water storage anomalies at the coarse spatial scale (4° × 5°) are primarily driven by meteorological anomalies rather than spatial variations in ecological parameters. Overall, these findings suggest that interactions between PFTs and environmental variables

are key to explaining variations in ecological functions related to vegetation growth and the carbon cycle, while water cycle dynamics are more strongly governed by environmental conditions or meteorological forcings."

A pertinent question is: At 0.25-degree resolution, what is the impact of excluding spatial variability within each PFT? It would be beneficial to present maps comparing results between PFT-only and PFT+CLIM+SOIL+AGE setups to underscore the importance of spatially varying parameters.
We thank the reviewer for this valuable suggestion. In response, we added maps that explicitly compare parameters retrieved from the PFT-only versus PFT+CLIM+SOIL+AGE setups. These maps highlight that the latter captures within-PFT variations that the PFT-only configuration fails to resolve (Fig. R10, Fig. S24 in the SI). For example, the PFT-only approach overestimates canopy photosynthetic efficiency (ce) in Arctic shrublands, since it cannot distinguish them from tropical shrublands of the same PFT. By incorporating climate information, however, the model accounts for mean temperature controls and produces more realistic parameter estimates.

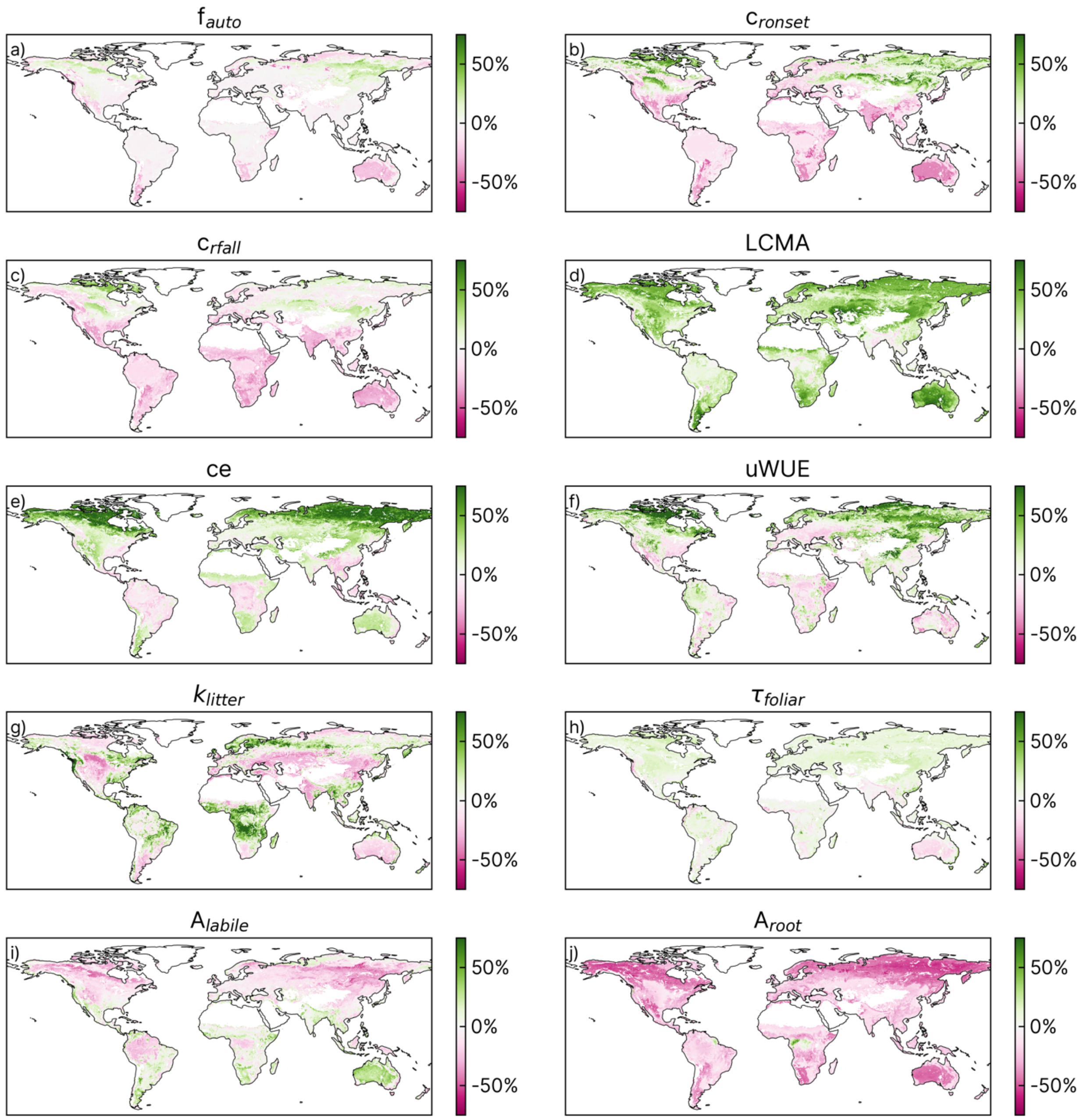

Fig. R10 (Fig. S24) | Relative parameter difference between the PFT-only and the PFT+CLIM+SOIL+AGE configurations.

To further assess the role of environmental gradients in shaping within-PFT variability of ecological parameters, we analyzed conditional SHAP values to quantify parameter dependencies on environmental gradients among grid cells dominated by the same plant type (Fig. 4). If parameters were uniform within each plant class, we would expect to see distinct values for each PFT only, with no additional variation across environments (as in Scenario 1 of Fig. R9). Conversely, if parameters were determined solely by abiotic environmental gradients and insensitive to plant type, we would expect a continuous scaling along

environmental gradients, with minimal differences among PFTs occupying similar environments (Scenario 2). Our experiments, however, reveal a more complex picture: ecological parameters not only vary substantially within PFTs (Scenario 3), but sometimes also exhibit different sensitivities to climatic gradients across PFTs (Scenario 4). We have clarified these findings and their implications in the revised text.

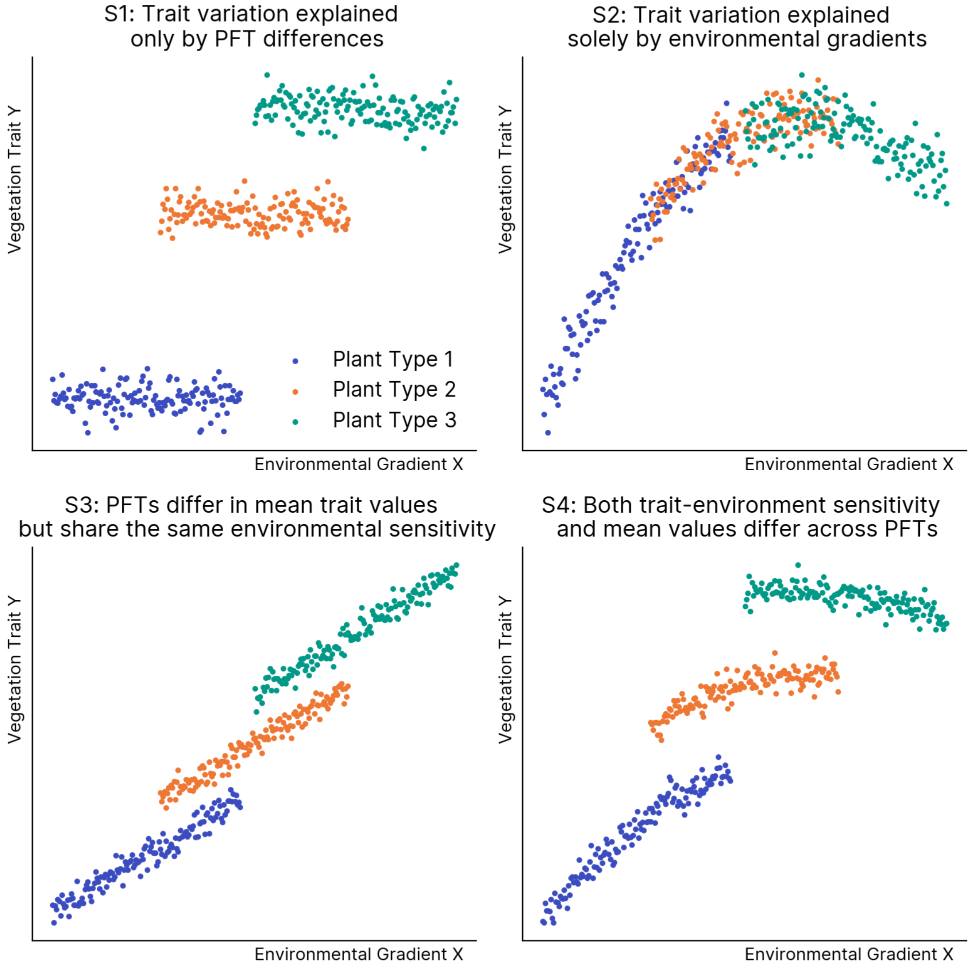

Fig. R9 (Fig. S1) | Conceptual illustration of four scenarios regarding the roles of plant functional types (PFTs) and environmental gradients in shaping the spatial distribution of plant functional traits. a) S1 – PFT-only control: Trait values vary between PFTs but remain largely constant within each PFT, showing minimal response to environmental gradients. b) S2 – Environment-only control: Trait values respond continuously to environmental gradients in a universal way, independent of PFT classification. c) S3 – Consistent scaling across PFTs: Mean trait values differ among PFTs, but trait–environment relationships (i.e., the slopes) are similar across plant types. d) S4 – PFT-specific trait–environment relationships: Both mean trait values and the sensitivity of traits to environmental gradients differ among PFTs, resulting in distinct trait–environment relationships for each plant type.

Line 530-593: To investigate the interaction between PFTs and environmental predictors, we compared functional relationships derived from model configurations with and without PFT predictors. The PFT-agnostic configuration (CLIM+SOIL+AGE) attempts to identify apparent global relationships between latent ecological parameters and spatial predictors, representing a hypothetical scenario where environment-trait relationships are uniform across plant types. Conversely, the PFT-aware model captures plant-type-specific dependencies between ecological parameters and environmental variables.

For a clearer extraction of environment-parameter relationships within each vegetation type, we restricted the SHAP analysis to pixels where a single plant type occupies at least 80% of the grid cell. Our analysis focused on four key ecological parameters that are robustly identifiable from global vegetation dynamics and exhibit distinct spatial patterns: photosynthetic efficiency, leaf carbon mass per area, carbon use efficiency, and root carbon allocation ratio. Among the environmental predictors, mean annual temperature, maximum canopy height, and—to a lesser extent—mean annual precipitation emerged as the most influential. We therefore examined their specific relationships with the selected ecological parameters (Fig. 4).

Mean annual temperature (MAP) exerts a dominant and often nonlinear influence on all four ecological parameters, indicating strong temperature regulation of ecosystem function consistent with the PC-level analysis. Photosynthetic efficiency rises with MAP from –15 °C to 15 °C before plateauing (Fig. 4a), while carbon use efficiency (NPP/GPP) declines from 0.45 to 0.35 and then stabilizes (Fig. 4g). Although global patterns are broadly mirrored within plant functional types (PFTs), notable differences exist in both mean values and temperature sensitivity. Grasslands and shrublands, spanning wide thermal ranges, generally exhibit higher photosynthetic efficiency (Fig. 4a), carbon use efficiency, (Fig. 4d) and root allocation ratios (Fig. 4j) than forests—traits reflecting acquisitive growth strategies in open habitats. In contrast, evergreen broadleaf forests show conservative strategies with low photosynthetic efficiency (Fig. 4a), high leaf carbon mass per area (Fig. 4d), and low carbon use efficiency (Fig. 4g), adapted to humid tropical conditions. Deciduous broadleaf and evergreen needleleaf forests exhibit sharp internal temperature sensitivity despite lower mean efficiencies. These PFT-specific patterns highlight that plant-type-specific responses can diverge from global trends, underscoring the need to account for ecological strategies in modeling trait–temperature relationships.

In contrast, the apparent global sensitivity of the four ecological parameters to mean annual precipitation largely reflects differences among plant functional types (PFTs) distributed along the precipitation

gradient, rather than variations within PFTs. For example, grasslands and shrublands—typically characterized by higher photosynthetic efficiency—are more common in drier regions than forests, resulting in an apparent global decline in photosynthetic efficiency with increasing precipitation (Fig. 4b). However, within-PFT variation in precipitation is generally small. For carbon use efficiency (Fig. 4h) and root carbon allocation ratio, we still observe negative relationships with precipitation within most PFTs, consistent with global trends, though the within-PFT sensitivities are substantially smaller than the differences across PFTs. The divergence between global and within-PFT patterns is most pronounced in the relationship between maximum canopy height and photosynthetic capacity: while the global negative relationship is driven by differences in photosynthetic efficiency across PFTs (Fig. 4c), the within-PFT relationship is positive, suggesting that regions with greater resource availability support both higher canopy stature and photosynthetic potential. These contrasting patterns underscore the complementary roles of plant type and environmental gradients in shaping ecological parameter variation, reflecting distinct ecological processes operating at different organizational scales.

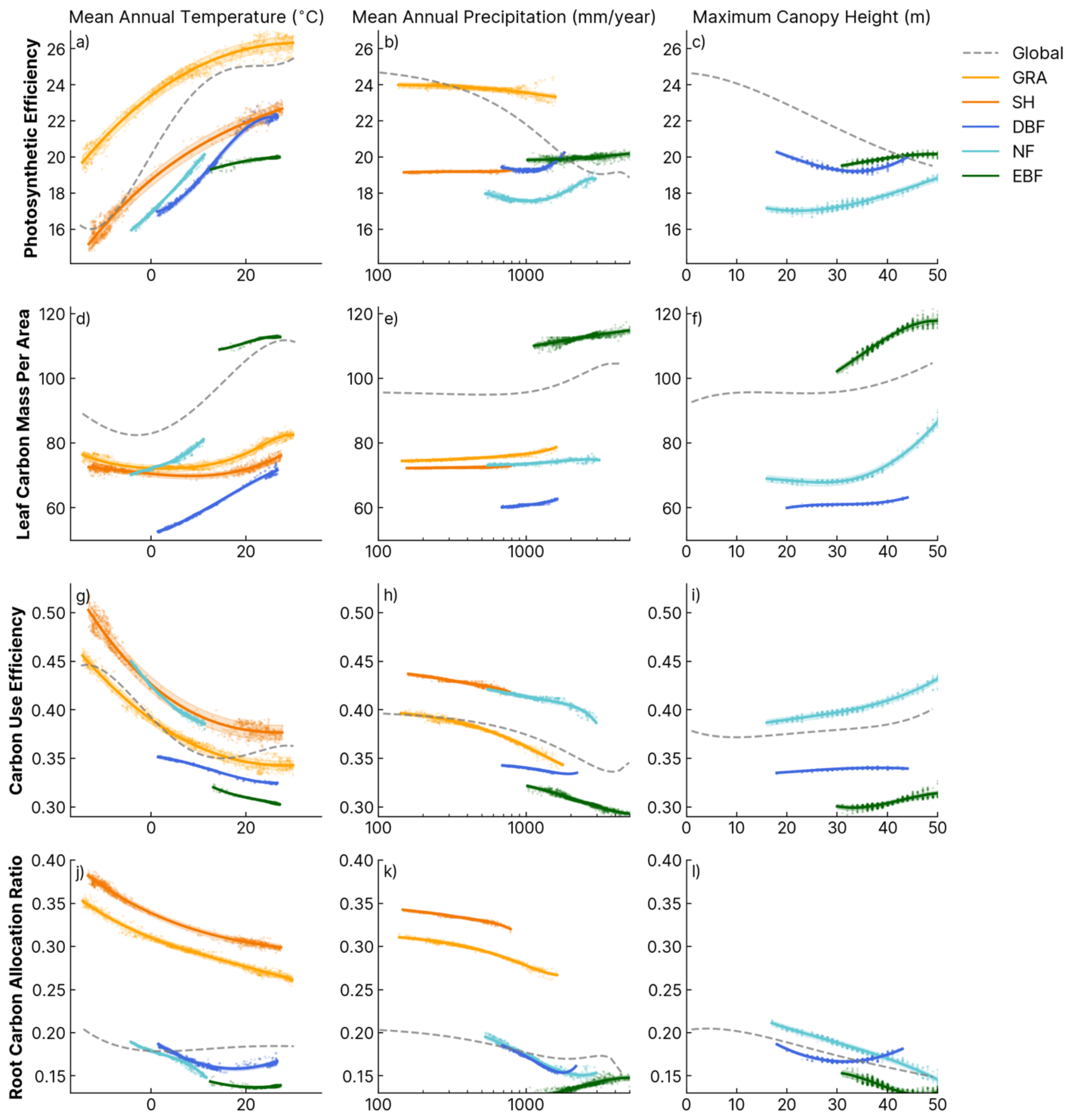

Fig. 4 | Comparison of global and PFT-specific trait–environment relationships. Panels show how nitrogen-limited photosynthetic efficiency (gC m^{-2} leaf day^{-2}), leaf carbon mass per area (gC m^{-2} leaf), carbon use efficiency (unitless, 0–1), and fine root carbon allocation ratio (unitless, 0–1) vary with mean annual temperature, mean annual precipitation, and maximum canopy height. The global relationships (dashed lines) represent ensemble-mean SHAP-derived dependencies from the CLIM+SOIL+AGE model configuration. PFT-specific relationships are derived from the PFT+CLIM+SOIL+AGE model configuration, using samples where the specified PFT comprises at least 80% of the pixel. PFT

abbreviations: GRA – grassland; SH – shrubland; DBF – deciduous broadleaf forest; NF – needleleaf forest; EBF – evergreen broadleaf forest.

2.Long-term prediction: DifferLand generates spatially varying parameters (as many alternative approaches/studies have already done), but do these parameters hold in time to enable more reliable long-term prediction? As a modeler, our perspective is to improve the predictability into the future, not only seasonally, but also over decades and centuries. Can DifferLand significantly improve TBMs' predictability, as seen in Fig. 4b of Gordon and Doney (2018) DOI: 10.1126/science.aam8328? Otherwise, its practical utility in enhancing TBM predictability is limited.

Thank you for raising this crucial point. We agree that the dominant role of structural errors in long-term projections of terrestrial biosphere models (TBMs) is a key motivation for developing our hybrid differentiable framework. While several studies have explored parameter spatialization, the distinctive contribution of DifferLand lies in its end-to-end differentiability, which integrates spatial correlations, trait covariation, and the physical consistency of process-based models. This design links long-term adaptation to prevailing climate with short-term responses to forcing anomalies, providing a more robust foundation for prediction.

As highlighted in the manuscript, single trait–environment relationships often exhibit limited predictability due to equifinality and confounding effects. In contrast, DifferLand retrieves coordinated axes of trait covariation in a latent parameter space, offering a more stable representation of vegetation dynamics across gradients. This multivariate, process-informed structure provides a pathway to reduce structural uncertainty and improve projections.

Although our evaluation in this study primarily focused on spatial generalizability—given the lack of direct benchmarks for decadal-to-centennial ecological forecasts—we argue there are strong reasons to expect improved long-term predictability. One way to frame this is through the concept of *emergent constraints* (Bowman et al., 2018). Traditional emergent constraint studies often rely on simple linear regressions between aggregated current and future states in an ensemble of climate models, but their success depends on two conditions: (1) robust observational constraints on current ecological states in model ensembles, and (2) a reliable link between current and future model states (Bowman et al., 2018). DifferLand contributes to both: its multi-stream data assimilation yields spatially explicit, multivariable constraints on the current climate–ecosystem state, and its differentiable, process-informed structure has

the potential to better capture the link between present and future states across an ensemble. These properties have the potential to make DifferLand ensemble projections more effective than traditional approaches in constraining uncertainties in future climate states.

We provide two pieces of supporting evidence. First, despite its simplified process representation, DifferLand achieves substantially lower RMSE in simulated NBE compared with state-of-the-art DGVMs when evaluated against atmospheric inversion datasets over decadal time scale (Fig. S8) consistent with previous analyses that parameter uncertainty is a major contributor to structural error and can be reduced through data assimilation. Second, as an indirect out-of-sample test, we evaluated DifferLand against CAMS NBE for 2003–2009, a period withheld from assimilation (which only used CMS-Flux NBE for 2010–2022). DifferLand successfully reproduced interannual anomalies at both global and regional scales (Fig. S31), suggesting that it can learn robust biosphere sensitivities to climate drivers and generalize to periods without direct observational constraints.

Together, these results indicate that while full decadal-to-centennial testing is beyond the scope of this study, DifferLand provides a structurally more reliable basis for improving long-term TBM predictability, directly addressing the concern raised by the reviewer.

A related question: can DifferLand improve prediction of the response of C cycle to CO2 fertilization and warming? As this study assumes space-for-time substitution (which is a big assumption), even with a relatively short-term training period with sizable interannual variability should still offer some improvement in model parameterization of CO2 and warming response. Also, how about predictability to extreme events in DifferLand?

We agree with the reviewer that DifferLand has strong potential to improve predictions of the carbon cycle under CO_2 fertilization, climate change, and extreme events. Indeed, we are already pursuing two follow-up studies in this direction. The first aims to quantify the effect of CO_2 fertilization on photosynthesis and water-use efficiency by combining long-term satellite observations with global CO_2 inversions, where the differentiable framework enables us to disentangle regional differences in plant responses to rising CO_2 (e.g., nutrient limitation, water availability, and biome-specific strategies). A second study applies DifferLand to investigate the ecological mechanisms linking carbon cycle anomalies to hydroclimatic extremes driven by El Niño–Southern Oscillation events in the Amazon basin. Together, these efforts further illustrate the capacity of DifferLand to address key uncertainties in interannual variability and long-term projections of the terrestrial carbon sink (as supported by comparison with TRENDY models shown in Fig. S8 and S9 of this study). However, a full treatment of these results is

beyond the scope of the present manuscript, and we plan to report them in more dedicated follow-up publications.

We note that DifferLand leverages both spatial and temporal variability in the dataset to retrieve parameters for dynamic processes such as carbon allocation, water stress, and the sensitivity of heterotrophic respiration to climate drivers. However, for more slowly varying processes, the informative signal arises primarily from spatial gradients at decadal timescales. In the revised discussion, we explicitly acknowledge its potential limitations while clarifying why this approach remains necessary at present, given the lack of long-term observational records needed to constrain ecological processes across multi-decadal timescales.

Line 771-786: We note that the conclusions drawn from this research are dependent on the timeframe considered for the observations. In this 23-year study, spatial predictors were treated as temporally invariant, under the assumption that their temporal variations are negligible compared to their mean spatial gradients. Furthermore, we assumed that the relationships between parameters and the environment remained stable throughout the study period, implying that plant acclimation and adaptation to environmental changes occur on much longer timescales. However, these assumptions may not always hold for long-term predictions, in which case environmental conditions, land use patterns, population distributions (Kelly & Goulden, 2008), community compositions(H. Liu et al., 2018), and plant functional responses (Franks et al., 2014) could all undergo significant shifts. At multidecadal to centennial timescale, slow processes such CO_2 fertilization (Quetin et al., 2023), nutrient limitation (Terrer et al., 2019), evolving forest demography (Robinson et al., 2024; H. Yang et al., 2023), and belowground carbon dynamics(Huang et al., 2024; Tao et al., 2023) can dominate the trajectories of land-atmosphere carbon exchange, yet most of these slow processes can be constrained only with long-term observations or by making equilibrium assumptions. In the absence of long-term global observational records on plant adaptation to climate change, the environment-parameter relationships learned from spatial gradients in this study offer insights into potential future shifts in ecosystem functions (Packett 1989), assuming the validity of space-for-time substitution (Damgaard, 2019).

3.Impact of uncertainty in remote sensing data: This study heavily relies on remote sensing products, which carry substantial uncertainties, and their respective uncertainties are asymmetrical. For example, it’s well-documented that MODIS LAI has significant bias (in both magnitude and seasonality) in the tropics; VOD data has huge data gaps in the tropics; LTSIF has never been validated in the tropics; CMS data has coarse resolutions that may not necessarily help constrain the spatial variability of model

parameters within each PFT, etc. How do these uncertainties affect parameter retrieval? Would alternative data products alter the conclusions? A thorough assessment of these uncertainties is essential to ensure DifferLand's robustness.

We appreciate the reviewer's comment regarding the need to conduct a thorough sensitivity analysis to evaluate whether DifferLand is robust to various uncertainties in the assimilated datasets. In the revised manuscript, we performed a detailed sensitivity analysis and found that our main conclusion that incorporating latent trait-environment relationships improves model's ability to simulate vegetation dynamics and ecological carbon and water fluxes is robust to the choice of the datasets. We reported our sensitivity analysis and the results in the SI Text S4.

Text S4 Sensitivity of model results to alternative assimilated datasets

We conducted sensitivity experiments to assess whether incorporating environmental variables consistently improves the simulation of vegetation dynamics compared with the PFT-only baseline, using alternative assimilated datasets. Table S1 lists the datasets used in each experiment. In each setup, the model was trained with an alternative data stream for one target variable—NBE (ΔNBE), LAI (ΔLAI), live biomass (ΔBiomass), VOD (ΔVOD), or fire emissions (ΔFire)—while keeping the datasets for all other target variables identical to the default configuration described in the main text. This design isolates the effect of using alternative data sources for individual target variables.

For NBE, we evaluated an alternative atmospheric inversion from the monthly Copernicus Atmosphere Monitoring Service (CAMS) spanning 2001–2023 (Copernicus Atmosphere Monitoring Service, 2020). In contrast to CMS-Flux, the primary CAMS inversion is constrained by ground-based CO_2 measurements rather than satellite-derived column-integrated CO_2, and it provides continuous coverage over the entire simulation period. The dataset was first conservatively reprojected from its native 1.4° × 0.7° resolution to 0.25° to match the model grid, and then assimilated at the 4° × 5° patch level following the same procedure used for CMS-Flux.

For LAI, we used the Copernicus LAI product, which merges SPOT-VGT (European Union's Copernicus Land Monitoring Service information, 2017a), PROBA-V, and Sentinel-3/OLCI (European Union's Copernicus Land Monitoring Service information, 2017b) observations into a continuous monthly record from 2001 to 2023. These datasets have been homogenized by the Copernicus team to ensure temporal

consistency. The Copernicus LAI series provides an alternative, MODIS-independent observational stream of canopy structure.

For biomass, we tested the IB-AGC (Xiaojun Li et al., 2025), a SMOS L-VOD informed annual live biomass product from 2010-2020 at 25km resolution, as an alternative to the Xu et al. 2020 biomass product used in the main method. The VOD-based biomass product has potential to provide better temporal patterns and reduce signal saturation in densely forested regions, although its shorter duration and wide gaps due to radio frequency interference (RFI-filtering) also pose additional challenges (Wigneron et al., 2024).

In addition, we also optionally assimilated GLAB-VOD (Skulovich, Gentine, et al., 2024), a global 18-day L-band equivalent AI-based vegetation optical depth (VOD) dataset at 25km resolution from 2003-2020 as an additional constraint for aboveground biomass. As the seasonal variations of L-VOD includes information from both canopy structure and water content, we assimilate VOD only at the annual level to minimize the signal of water content (Wigneron et al., 2024). We used a linear observation operator assuming that annual L-VOD can be approximated as a linear combination of the contributions from annual mean foliar biomass and annual mean woody biomass

$$\overline{VOD_{annual}} = p_{wood} \times \overline{C_{wood}} + p_{fol} \times \overline{C_{fol}}$$

Where p_{wood} and p_{fol} are treated as model parameters to be predicted by the spatialization network. This linear observation operator was chosen as opposed to an exponential function form for its parsimony and empirical efficacy in a previous model-data fusion study (Skulovich, Famiglietti, et al., 2024). Lastly, we also tested assimilating fire carbon emission using the CMS Carbon Flux for Fire inversion between 2010 and 2016 (Bowman, 2020), informed by satellite column carbon monoxide concentration measurements from MOPITT, at the 4° × 5° patch level.

Table S1 Experiment Setups for Testing Model Sensitivity to Alternative Assimilated Datasets

	NBE	Leaf Area Index	Biomass	VOD	Fire C Emission
**Default**	CMS-Flux GCB 2023 (2010-2023)	BNU Reprocessed LAI (2001-2023)	Xu et al. 2020 (2001-2019 annual)	Not Used	GFED5 Fire Emission (2001-2022, patch level assimilation)
**ΔNBE**	**CAMS NBE (2001-2023)**	--	--	--	--
**ΔLAI**	--	**COPERNICUS LAI (2001-2023)**	--	--	--

**ΔBiomass**	--	--	**IB-AGC (2010-2020, annual)**	--	--
**ΔVOD**	--	--	--	**GLAB-VOD (2003-2020, annual mean)**	--
**ΔFire**	--	--	--	--	**CMS-Flux Fire (2001-2018 monthly 4x5°)**

"--": same as the default configuration

Each experiment was trained under both the PFT-only and PFT+CLIM+SOIL+AGE configurations, with ten independent runs for each setup. We then selected the five runs with the lowest training loss to reduce random uncertainties from model initialization. All variables are assimilated monthly at the 0.25° model grid unless otherwise specified. Model performance on the test dataset was evaluated using the R^2-score on LAI, SIF, NBE, Live Biomass (LBM), ET, and EWT. For a fair comparison, both the default and the alternative models are evaluated on the same set of models used to train the model, thereby avoiding discrepancies in performance metrics arising from differences in the underlying dataset values.

We found that including environmental predictors consistently improves simulated vegetated states and ecological dynamics, independent of the choices of individual alternative datasets (Fig. S29). The robustness likely stems from (1) the overall consistency in the spatial patterns of target variables across different datasets, and (2) the resilience of the multi-stream data assimilation framework to variations in individual target variables (Fig. S5), as the model's structural assumptions and complementary constraints from other variables buffer the impact of any single target variable on overall vegetation dynamics.

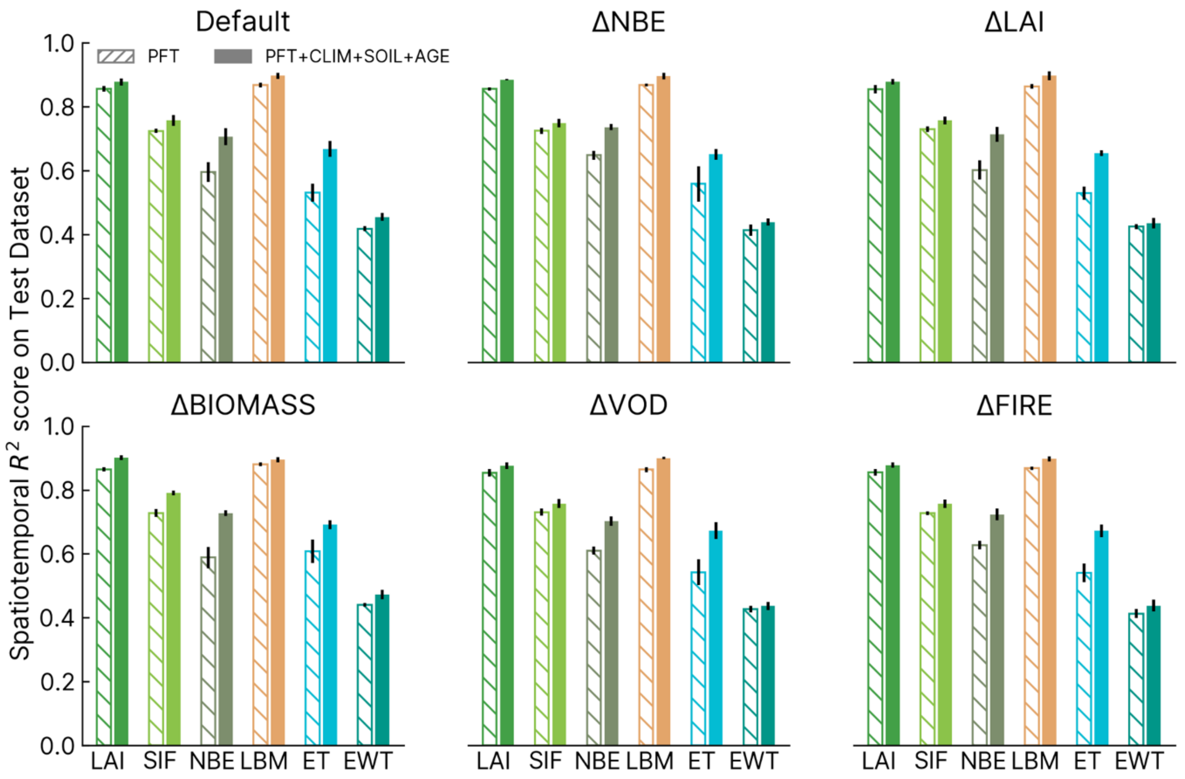

Fig. S29 | Sensitivity analysis of model performance for alternative assimilated datasets. For each sensitivity experiment, we compared the aggregated spatiotemporal R^2-score on held-out text pixels between the PFT-only baseline and the PFT+CLIM+SOIL+AGE configuration.

Because NBE from atmospheric inversion serves as a critical top-down constraint on the terrestrial carbon cycle—integrating ecosystem carbon fluxes—yet carries larger spatial uncertainties due to limitations in atmospheric transport modeling (J. Liu et al., 2021), we further examined how simulated carbon fluxes differ globally and regionally when using CMS-Flux (in the default runs) versus the CAMS inversion (in ΔNBE). We found that the ΔNBE runs show consistent spatial and temporal patterns as the default runs (Fig. S30), although the overall net carbon sink is stronger at -2.79±0.94 PgC/year compared with the -2.09±0.62 PgC/year in the default runs, consistent with the more sinky pattern in CAMS (-2.77 PgC/year) versus CMS (-2.08±0.09 PgC/year). The global annual NBE anomaly correlation between ΔNBE and CAMS is slightly lower than the correlation between default runs and CMS-Flux (Fig. S31a). Region comparisons suggest CAMS exhibits large interannual excursions in annual NBE trajectories in the South American Tropical (Fig. S31d), South American Temperate (Fig. S31d), North Africa (Fig. S31f), and Tropical Asia (Fig. S31j), potentially due to the paucity of surface CO_2 measurements in those TRANSCOM regions to constrain the carbon dynamics. In contrast, seasonal cycle correlations with the

assimilated datasets are consistently strong regardless of the choice of atmospheric inversion, confirming DifferLand's ability to capture seasonal variations in land carbon flux at global and regional levels (Fig. S32).

As CAMS is available prior to 2010, the ΔNBE runs may also serve as a baseline to examine whether the default runs that assimilate NBE only after 2010 are able to reproduce the overall carbon dynamics. Despite differences in mean NBE arising from biases in the respective atmospheric inversions, the default and ΔNBE runs exhibit broadly consistent annual anomalies in global and regional carbon fluxes before 2010 (Fig. S32). This consistency suggests that the multi-stream data assimilation framework can capture carbon cycle dynamics even in historical periods without direct NBE constraints.

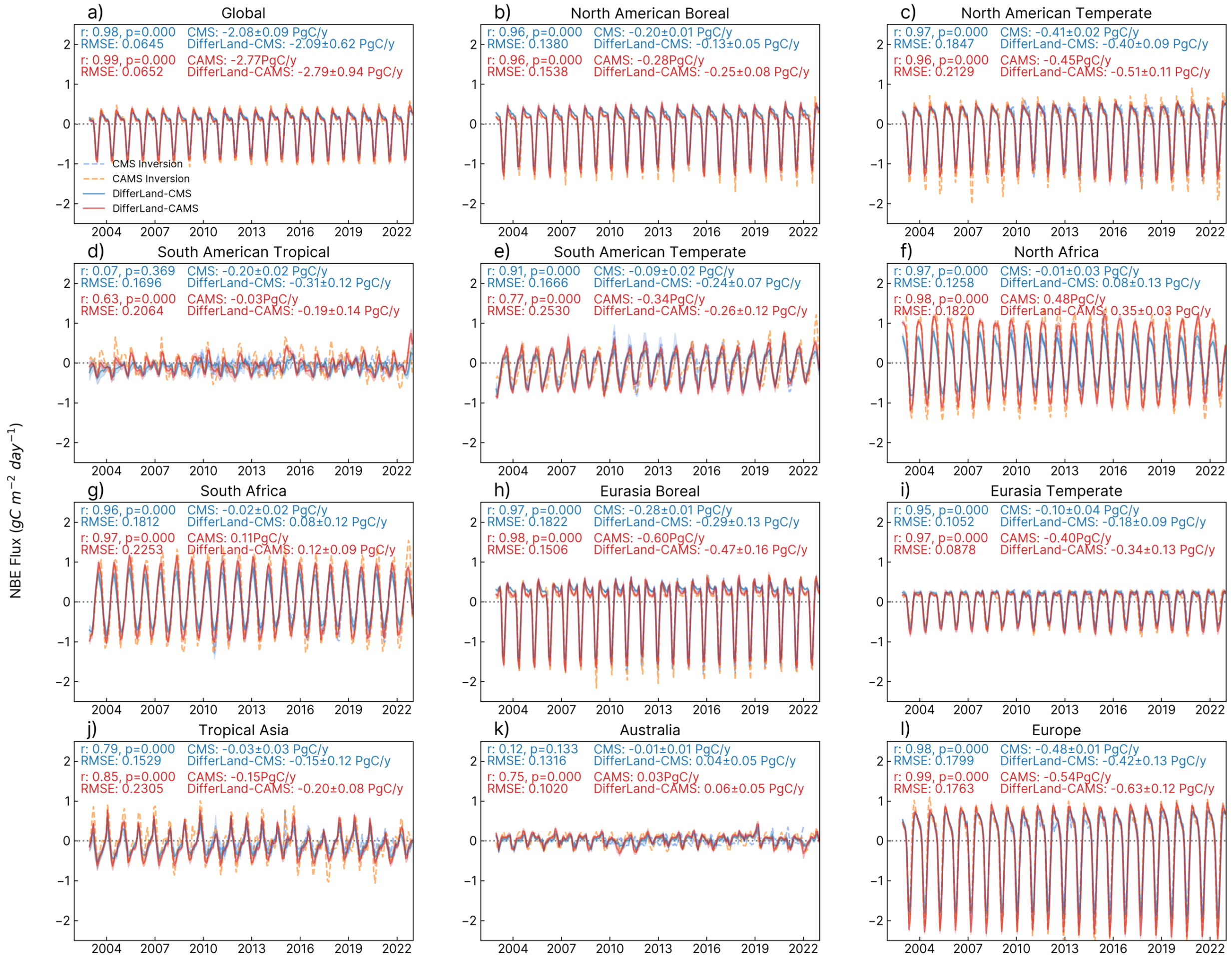

Fig. S30 | Sensitivity of decadal carbon cycle dynamics to an alternative choice of assimilated atmospheric inversion. Here we compare DifferLand's simulated net biosphere exchange at the global level and regional levels using either satellite column-integrated CO_2 constrained CMS-Flux or the

ground CO_2 measurement constrained CAMS atmospheric inversion. CAMS provides longer coverage that allows a longer data record to be assimilated between 2001-2023, whereas CMS-Flux was available only between 2010-2022 when we conducted the experiment.

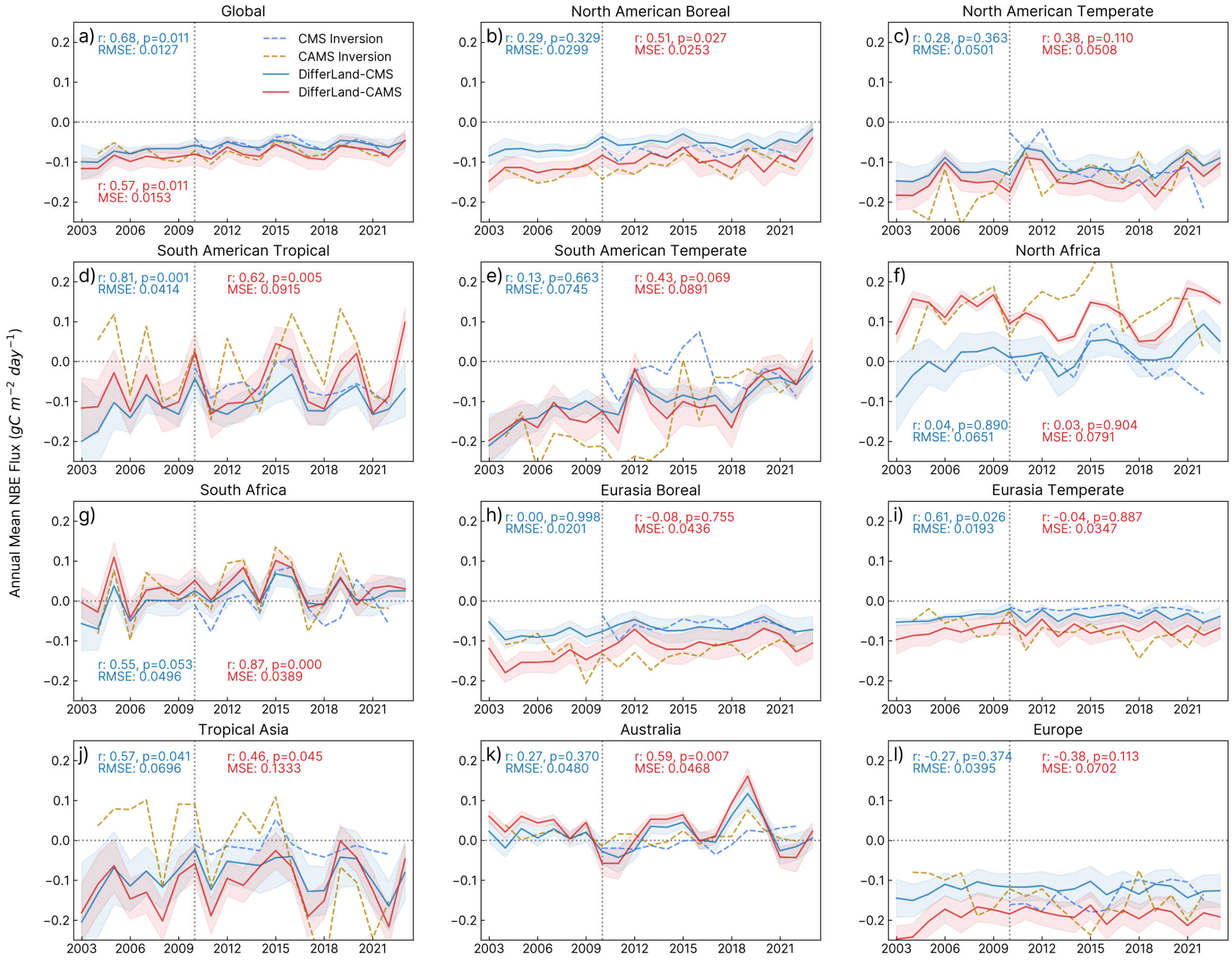

Fig. S30 | Similar to Fig. S30, but comparing only the annual mean NBE. The vertical dotted line indicates period before 2010 when only CAMS NBE inversion was available.

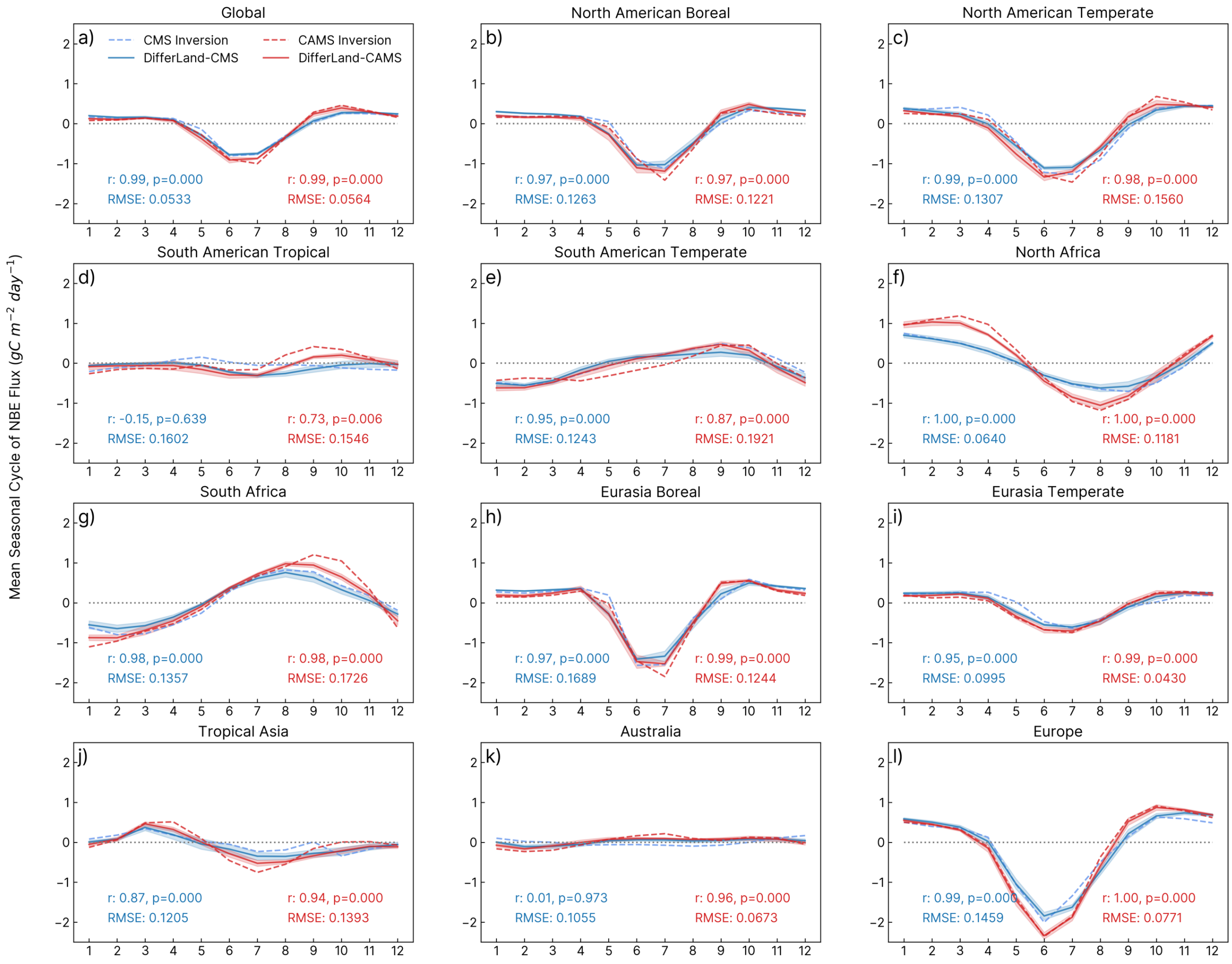

Fig. S32 | Similar to Fig. S30, but comparing the annual mean seasonal cycle.

We believe these sensitivity analyses highlight a key strength of integrating multiple streams of observations within a unified latent space to constrain underlying ecological processes. Such multimodal approaches are emerging as central methods in modern AI and computational sciences, including foundational models in Earth and geospatial research that learn low-dimensional embeddings (Bodnar et al., 2025; Brown et al., 2025). However, embeddings in purely AI-based models provide only abstract representations of geospatial information and cannot be directly linked to physical processes. In contrast, the latent environment–parameter relationships identified here are interpretable and physically consistent, offering the potential to robustly constrain the dynamics of multiple downstream ecological processes and variables.

4.Agriculture intensity: The claim that agricultural intensity is a major driver of spatially varying parameters requires caution. Agriculture intensity would indeed impact carbon fluxes, however, not through parameter spatialization, but through genetic cultivar differences and management practices, as

well as their relatively rapid temporal changes and technology adoptions (as opposed to natural systems). This comes to the question of how agriculture systems are treated in DALEC and DifferLand? As this is an intermediate complexity model, the agriculture system must be overly simplified. Therefore, it is puzzling that how agriculture intensity is impacting spatial variability of model parameters. Also, agriculture intensity can mean different things in different communities, what exactly the definition of agriculture intensity is in this study?

We agree with the reviewer that the phrasing “agricultural intensity is a major driver of spatially varying parameters” may cause confusion. What we intended to convey is that the third principal component of parameter variation is closely associated with cropland distribution, as shown in both the spatial pattern and the SHAP feature importance analysis for PC3 (Fig. 3). To avoid ambiguity, we revised our wording from “agricultural intensity” to the more precise term “cropland fraction.”

5.Parameter sensitivity and resolved/unresolved processes: Identifying which of the 40 parameters is most sensitive to spatialization is crucial. The most sensitive parameters indicate unresolved processes needing data-driven models, while the least sensitive highlight robust processes where spatial variation isn’t a priority.

We very much agree with the reviewer that identifying which of the 40 parameters is most sensitive to spatialization is crucial. To do so, we performed a parameter sensitivity analysis as detailed in the methods section. Subsequent analysis of trait coordination and trait-environment relationships are all based on parameters that meet the identifiability criteria.

Line 1195-1204: We conducted parameter identifiability analyses on the three setups using either the full set of predictors (PFT+CLIM+SOIL+AGE, Fig. S36), the environmental predictors only (CLIM+SOIL+AGE, Fig. S37), or the PFT predictors only (PFT, Fig. S38). For each model parameter and initial pool value, we computed the pixelwise coefficient of variance (CV) across ensemble members and used the spatial median CV as a diagnostic for predictor robustness. The rationale of this test is that if an ecological parameter is well constrained by the assimilated observations, it should converge to relatively stable values across independently initialized runs. Conversely, non-identifiable parameters with low sensitivity to the observational constraints can be expected to take substantially different values across independently initialized runs. We used a spatial median CV < 0.6 to select parameters for subsequent analyses to balance parameter robustness with process coverage under the available constraints.

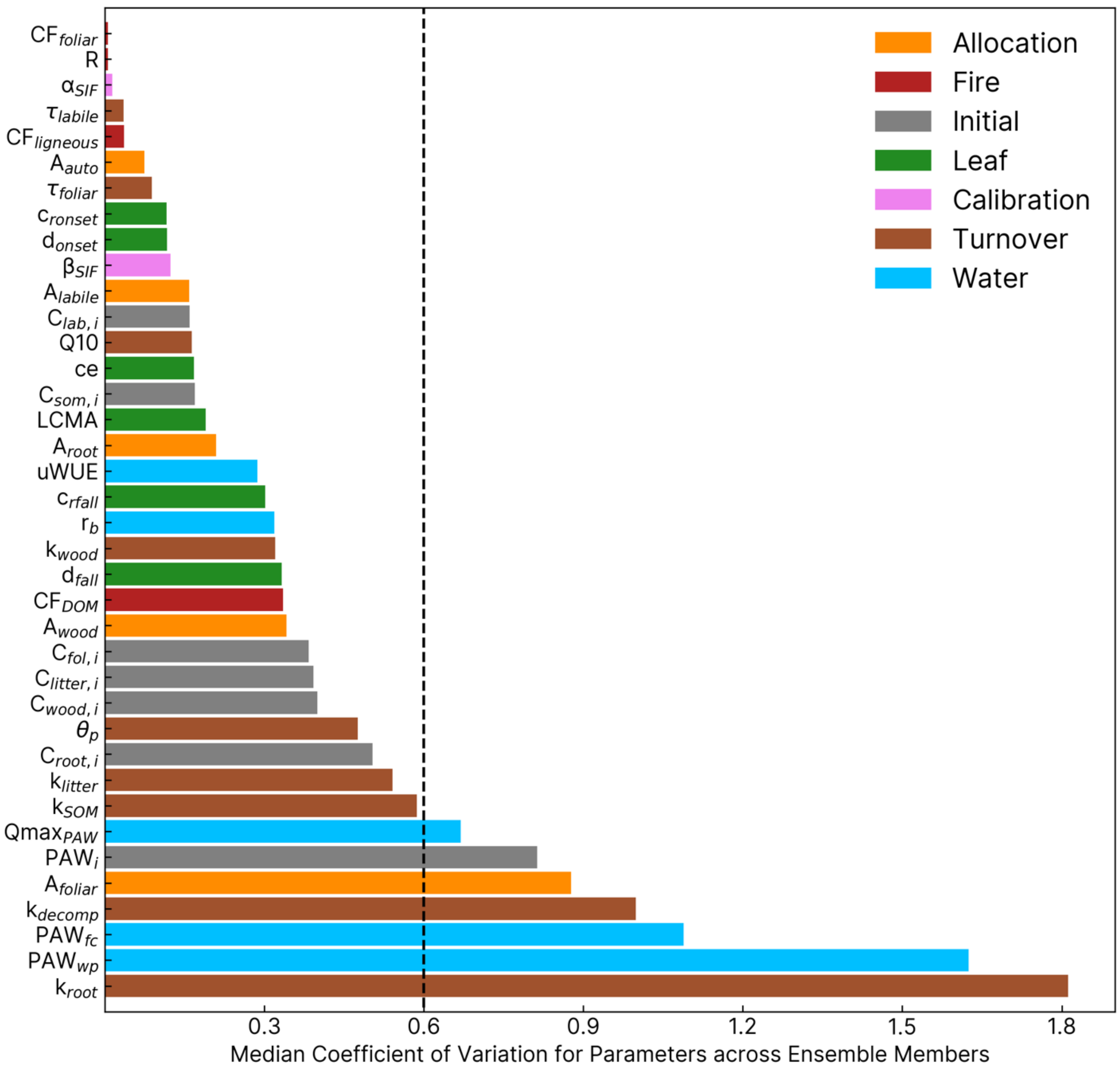

Fig. S36 | The spatial median coefficient of variation of parameters across 10 out of 20 ensembles of the PFT+CLIM+SOIL+AGE configuration that achieved the best training loss. The parameters are ranked with ascending median coefficient of variation. We used a median coefficient of variation of 0.6 as a threshold for identifiable latent model parameters. The parameters are color coded to represent their role in the DALEC model (see legend).

6.Performance of C storage: This study focuses on carbon fluxes only, of course due to more accessible and mature satellite products. But when quantifying carbon sequestration, the above- and below-ground biomass are more critical. Can the spatial-varying parameters in DifferLand improve carbon storage simulations?

We thank the reviewer for raising this important question. DifferLand does not focus only on carbon fluxes but also on carbon pools. Quantifying a model's ability to capture biomass storage is indeed a crucial step in evaluating its capacity to simulate long-term carbon sequestration by the terrestrial biosphere. As shown in Fig. R11, DifferLand's simulated total live biomass (above- and belowground) closely matches the assimilated Xu et al. (2021) dataset, reproducing both the mean spatial gradients and the temporal trends. In particular, the model captures biomass gains in East Asia and the eastern United States due to forest regrowth, as well as biomass losses in parts of the boreal and Southeast Asian tropical forests. Nevertheless, discrepancies remain in regions with pronounced deforestation, such as in the "arc of deforestation" of Amazon, where DifferLand underestimates biomass loss. This limitation primarily arises because the model does not yet incorporate explicit land-use and land-cover change forcing. Future work should integrate such drivers to better account for the anthropogenic impacts on biomass dynamics.

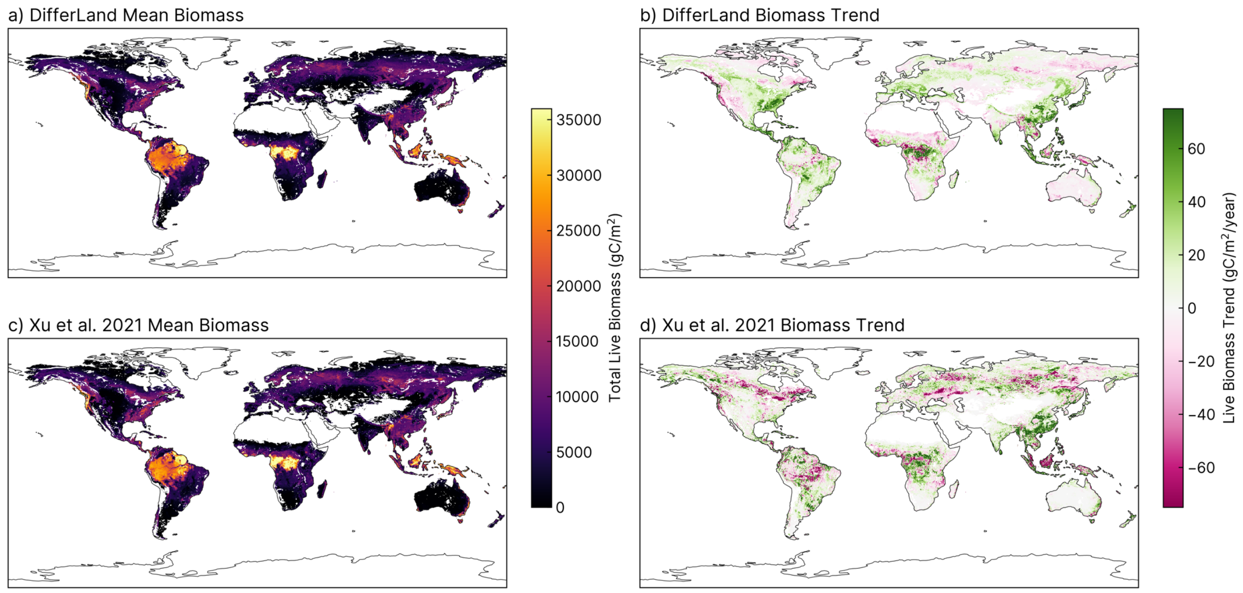

Fig. R11 (Fig. S22) | Evaluation of DifferLand simulated live biomass against the assimilated Xu et al. 2021 biomass. a) DifferLand mean biomass (2003-2020); b) DifferLand Biomass trend; c) Xu et al. 2021 mean biomass; d) Xu et al. 2021 biomass trend.

7.Model assessment: The current model assessment combines spatial and temporal variability, likely resulting in high R^2 values. Would these results hold if decomposed into seasonal, interannual, and trend analyses? The current high R^2 is primarily driven by seasonal and spatial variations.

To assess whether DifferLand can robustly capture carbon dynamics across different timescales—including decadal trajectories (Fig. S10), interannual anomalies—a major challenge for most DGVMs (Fig. S11), and seasonal cycles (Fig. S12)—we compared DifferLand-simulated net biome exchange

(NBE) with the TRENDYv12 S3 ensemble of state-of-the-art dynamic global vegetation models (DGVMs). We found that DifferLand consistently reduced RMSE (Fig. S8) and has competitive correlation with benchmark dataset compared with the majority of the DGVMs (Fig. S9), demonstrating improved skill in representing both short- and long-term carbon dynamics after data assimilation.

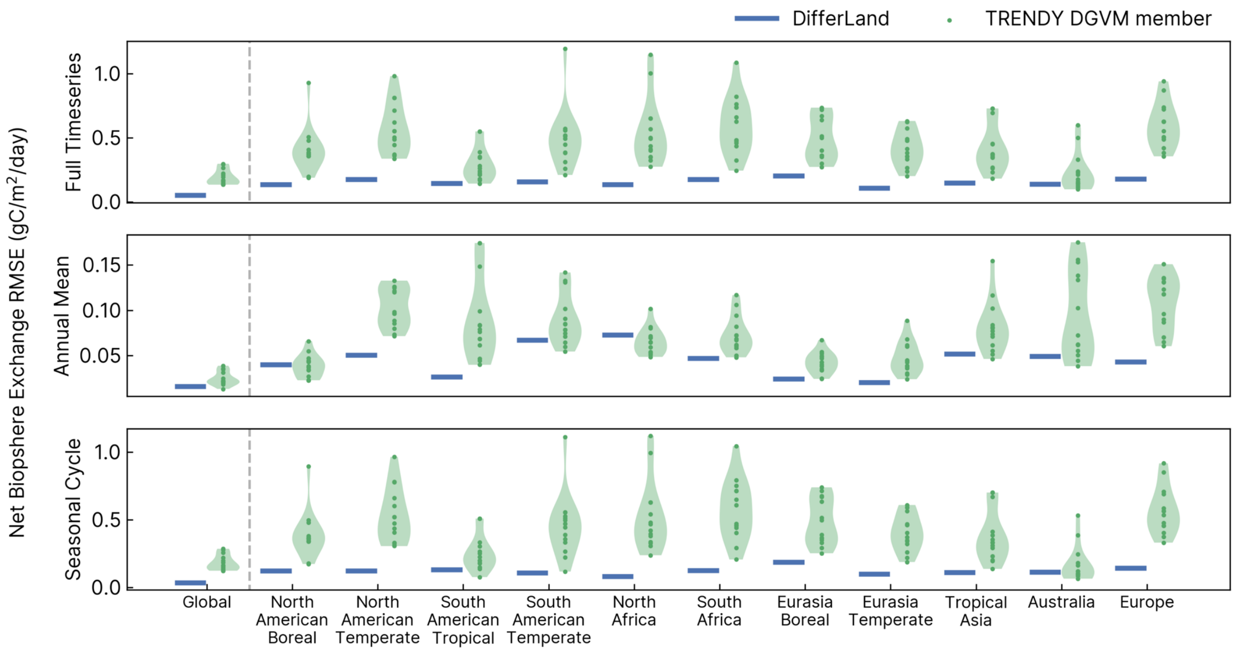

Fig. S8 | Comparison of net biosphere exchange (NBE) performance between DifferLand and TRENDY DGVMs across regions and temporal scales. Violin plots show the root mean square error (RMSE; gC m^{-2} day^{-1}) of NBE simulations compared to benchmark observations from the CMS-Flux atmospheric CO_2 inversion for the period 2010–2022. Errors are evaluated over: (top) the full time series, (middle) annual mean, and (bottom) seasonal cycle. Each green violin represents the distribution of RMSE from individual TRENDY dynamic global vegetation model (DGVM) members, while blue bars indicate the RMSE of DifferLand. Evaluations are conducted globally and across 11 TRANSCOM regions. Lower RMSE values indicate better agreement with the observational benchmark.

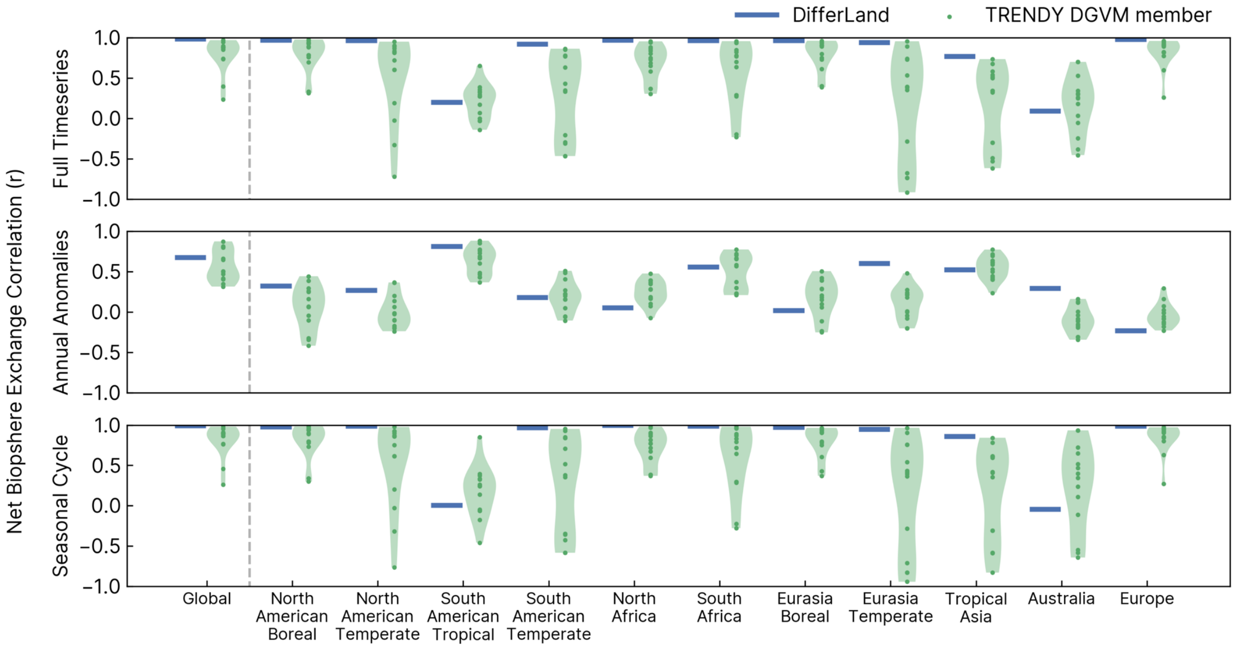

Fig. S9 | Comparison of net biosphere exchange (NBE) correlation between DifferLand and TRENDY DGVMs across regions and temporal scales. Violin plots show the Pearson correlation coefficient (r) between simulated and observed NBE from the CMS-Flux atmospheric CO_2 inversion over the period 2010–2022. Correlations are evaluated over: (top) the full time series, (middle) annual anomalies, and (bottom) seasonal cycles. Each green violin represents the distribution of correlations from individual TRENDY dynamic global vegetation model (DGVM) members, while blue bars indicate the correlation achieved by DifferLand. Evaluations are performed globally and across 11 TRANSCOM regions. Higher correlation values indicate better temporal agreement with the benchmark observations.

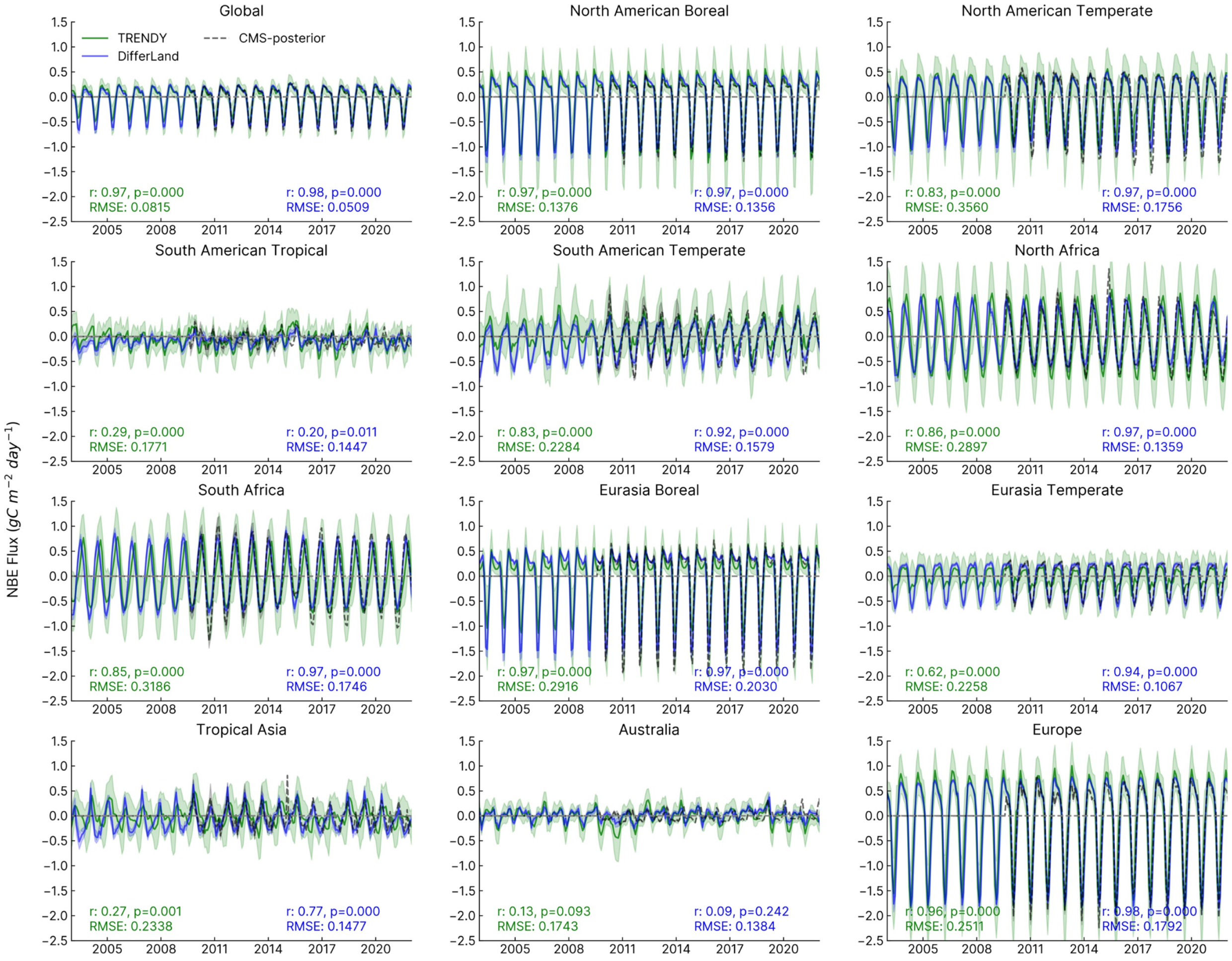

Fig. S10 | Time series of net biosphere exchange (NBE) from DifferLand and TRENDY models against CMS-Flux atmospheric CO_2 inversion. Time series of NBE (gC m^{-2} day^{-1}) from DifferLand (blue) and the multi-model mean of TRENDY DGVMs (green) from 2003 to 2022, as well as the benchmark CMS-Flux posterior from the available period of 2010-2022 (black). Panels show comparisons at the global scale and across 11 TRANSCOM regions. Pearson correlation coefficients (r), *p*-values, and RMSE values are reported for DifferLand and TRENDY relative to CMS-Flux.

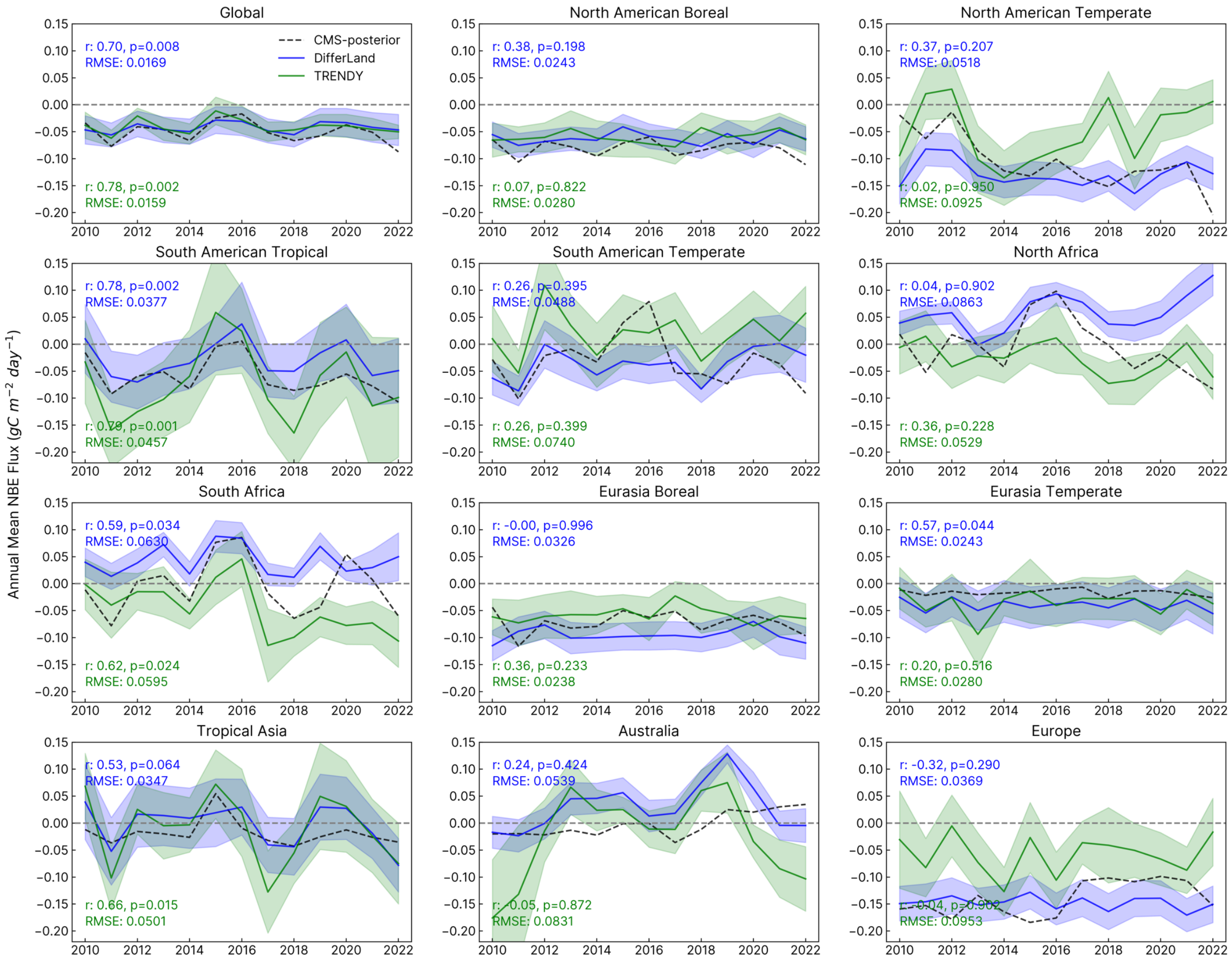

Fig. S11 | Annual mean net biosphere exchange (NBE) from DifferLand and TRENDY models against CMS-Flux atmospheric CO_2 inversion.

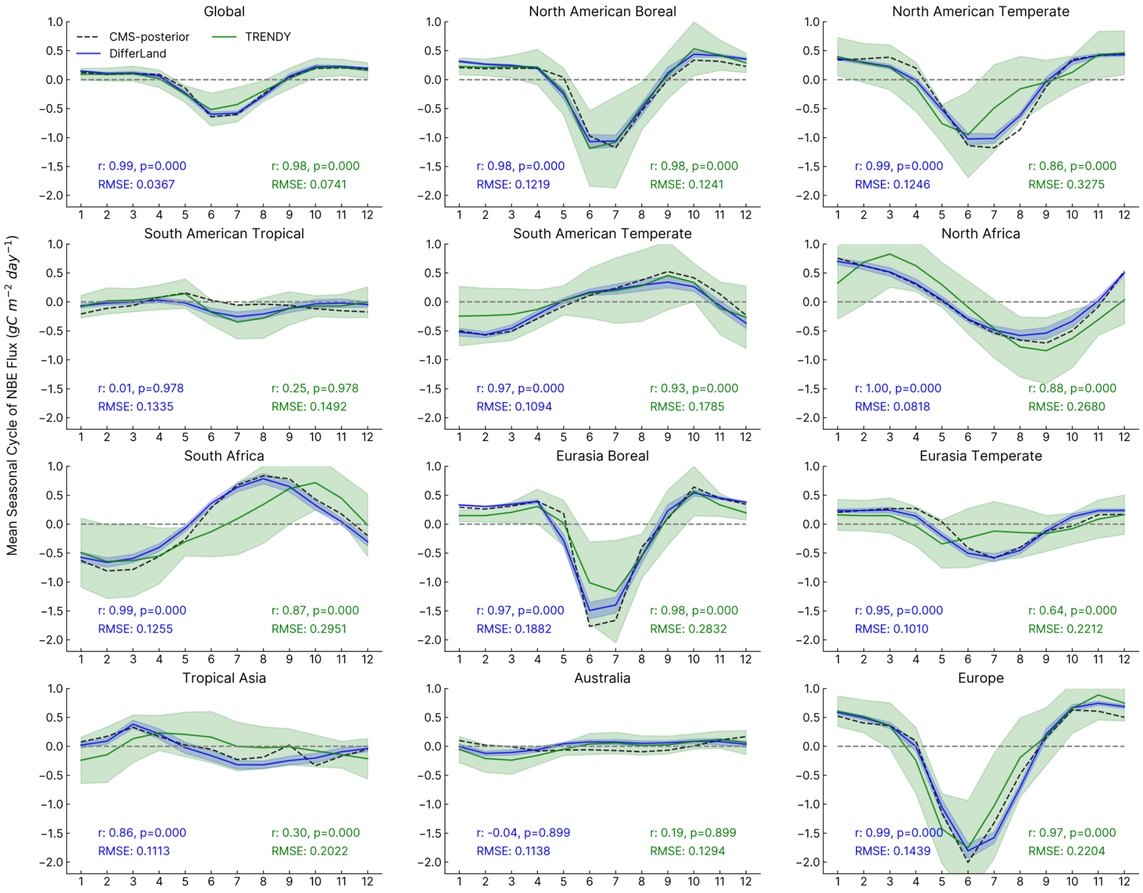

Fig. S12 | Mean seasonal cycle of net biosphere exchange (NBE) from DifferLand and TRENDY models against CMS-Flux atmospheric CO_2 inversion.

In addition, we evaluated pixelwise monthly temporal correlations between simulated and benchmark datasets for SIF, NBE, EWT, ET, and LAI, using (i) the full time series (2003–2023, Fig. S13) and (ii) annual anomalies (Fig. S14). The former removes the influence of spatial gradients, isolating regional temporal performance, while the latter removes the mean seasonal cycle, focusing on interannual variability. Across much of the globe, simulated and observed variables showed consistently high correlations. Correlations were somewhat lower in tropical and sparsely vegetated regions, reflecting greater uncertainties in satellite benchmarks due to cloud cover, signal saturation, or inherently low signal-to-noise ratios.

For annual anomalies, DifferLand captured interannual variability in the water cycle particularly well, achieving strong correlations for EWT and ET, which exhibit pronounced year-to-year fluctuations. The model also showed robust agreement with reconstructed SIF (LCSPP) and captured interannual variability of NBE in tropical and semi-arid ecosystems—regions known to drive global interannual carbon flux variability. In contrast, correlations for NBE were weaker in temperate and boreal regions such as Europe and Siberia, where vegetation productivity is largely light-limited and characterized by strong seasonal amplitudes but relatively small interannual signals.

Interannual correlations for LAI were generally low, except in regions experiencing substantial greening trends (e.g., East Asia). This limitation reflects the structural constraints of the DALEC phenology module, which prescribes fixed leaf onset and senescence dates each year and does not account for interannual variability in phenology. While dynamic phenology modules that respond to interannual climate variability (e.g., temperature-driven phenology) have been shown to improve such correlations, addressing this limitation is beyond the scope of the current study. Importantly, it does not affect our main conclusions, which focus on the spatial gradients of trait–environment relationships. We discussed this known structural limitation in Text S7 and highlighted the phenology submodule as a valuable avenue for future hybrid model development.

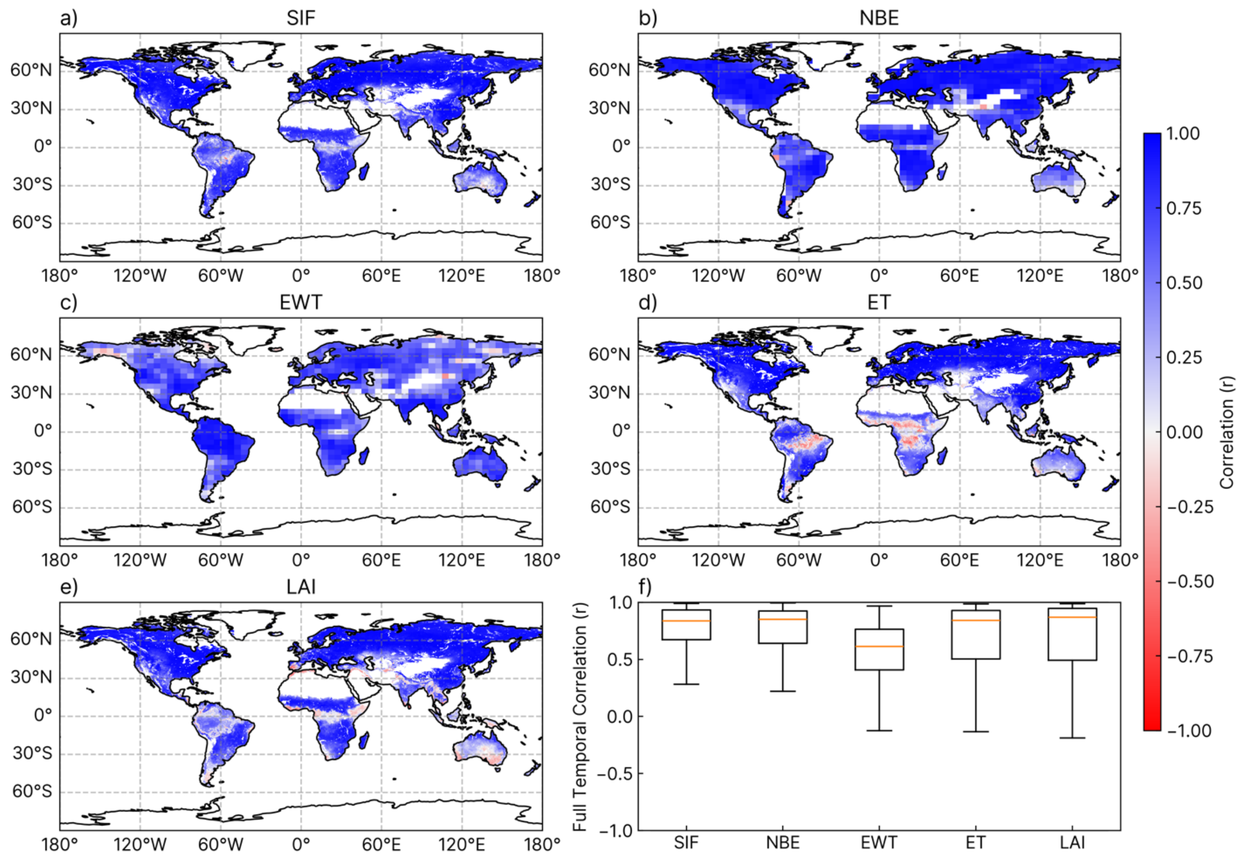

Fig. S13 | The pixelwise temporal correlation between simulated and observed variables. a) SIF, b) NBE, c) EWT, d) ET, e) LAI. The panels display the average correlation values across the 10 out of the 20 ensemble members using the full set of predictors with the lowest training loss. f) Shows the distribution of pixelwise correlations across pixels.

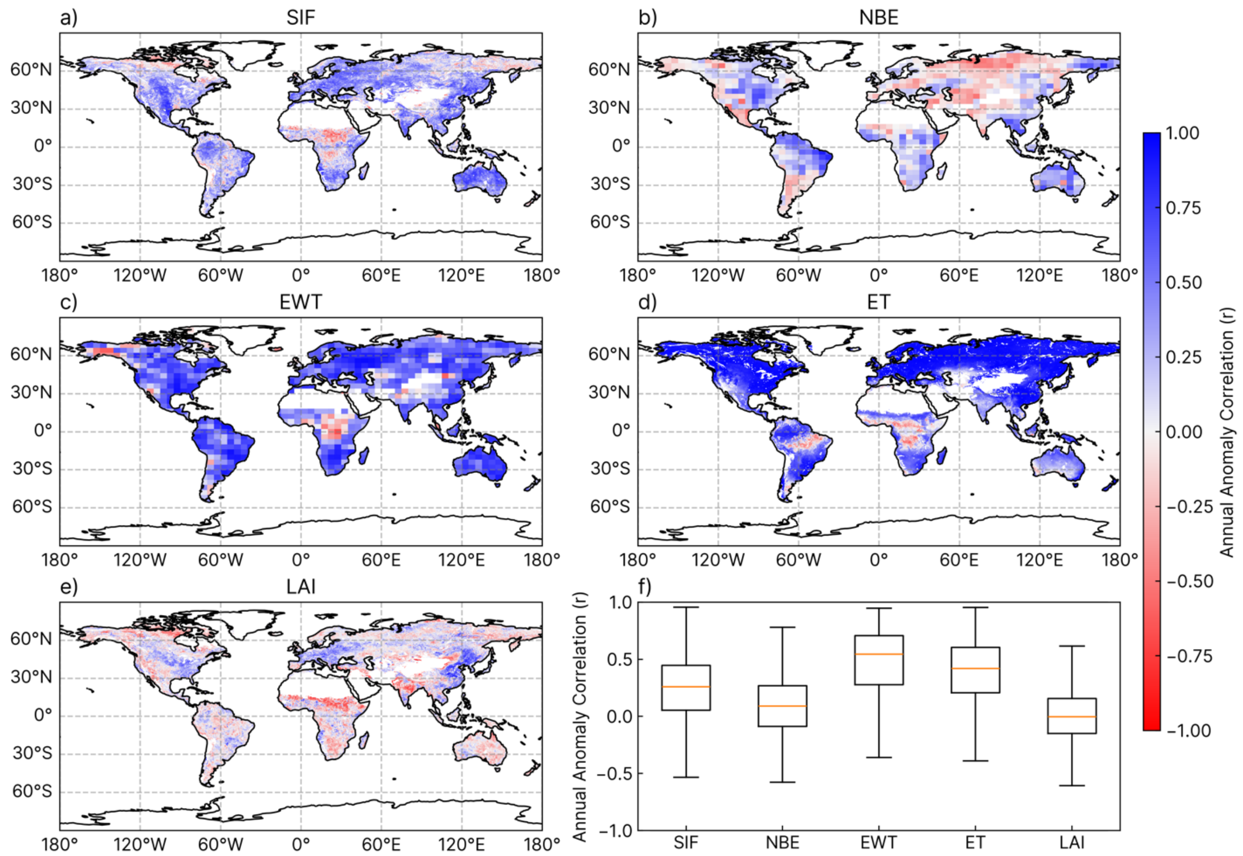

Fig. S14 | The pixelwise temporal correlations of the annual anomalies of simulated and observed variables. a) SIF, b) NBE, c) EWT, d) ET, e) LAI. The panels display the average correlation values across the 10 out of the 20 ensemble members using the full set of predictors with the lowest training loss. f) Shows the distribution of pixelwise correlations across pixels. Note: The low correlation in interannual LAI anomalies arises from DALEC2's fixed leaf onset and senescence (Bloom & Williams, 2015), which do not respond to interannual atmospheric variability. This limitation could be addressed by incorporating dynamic leaf carbon allocation within a more realistic phenology module (e.g., Norton et al., 2023).

8.Technical questions:

a.How top-down and bottom-up data products complement each other? What if only top-down? And What if only bottom-up?

Thank you for raising this important question. We believe that this is a fundamental advantage of DifferLand as they benefit each other. We conducted a detailed sensitivity analysis in which we either excluded bottom-up observations (i.e., eddy covariance flux data) or applied more stringent and relaxed thresholds for site representativeness within a grid cell. The results of these tests are reported in Text S5 of the SI. We did not attempt to train DifferLand using only site-level data, as such an approach would

lack sufficient spatial variability to capture the large-scale distributions of many ecological variables—particularly in the tropics and the Southern Hemisphere, where publicly available eddy covariance observations are sparse.

Text S5 Sensitivity analysis of different filtering criteria for spatial mismatches between assimilated eddy covariance datasets and model grid cells.

A source of uncertainty in this study arises from the scale mismatch between the eddy covariance (EC) tower footprint (Chu et al., 2021)—where turbulent fluxes of land–atmosphere exchange are measured, typically spanning ~100 m to several kilometers depending on wind direction and tower height—and the much larger 0.25° model grid cell (~25 km). To reduce this mismatch, we compute grid-cell mean fluxes by averaging across multiple EC sites within a cell whenever possible and exclude towers that represent atypical managed habitats (e.g., AU-Lox, an almond orchard in South Australia's Riverland). However, many grid cells contain only a single operating site, if any. To ensure minimal consistency between site vegetation and the containing grid cell, we require a threshold fraction of plant functional type (PFT) coverage for a site to be included. Higher thresholds improve representativeness but reduce site availability, whereas lower thresholds increase the risk of spatial mismatch. As a compromise, we tested four filtering schemes: a minimal coverage of 10% (ΔEC10, N = 213 sites), 25% (default, N = 180), and 50% (ΔEC50, N = 123), along with a control case excluding all EC data (ΔEC). The spatial distribution of the included sites and the number of sites per plant functional type under each threshold are shown in Fig. S33.

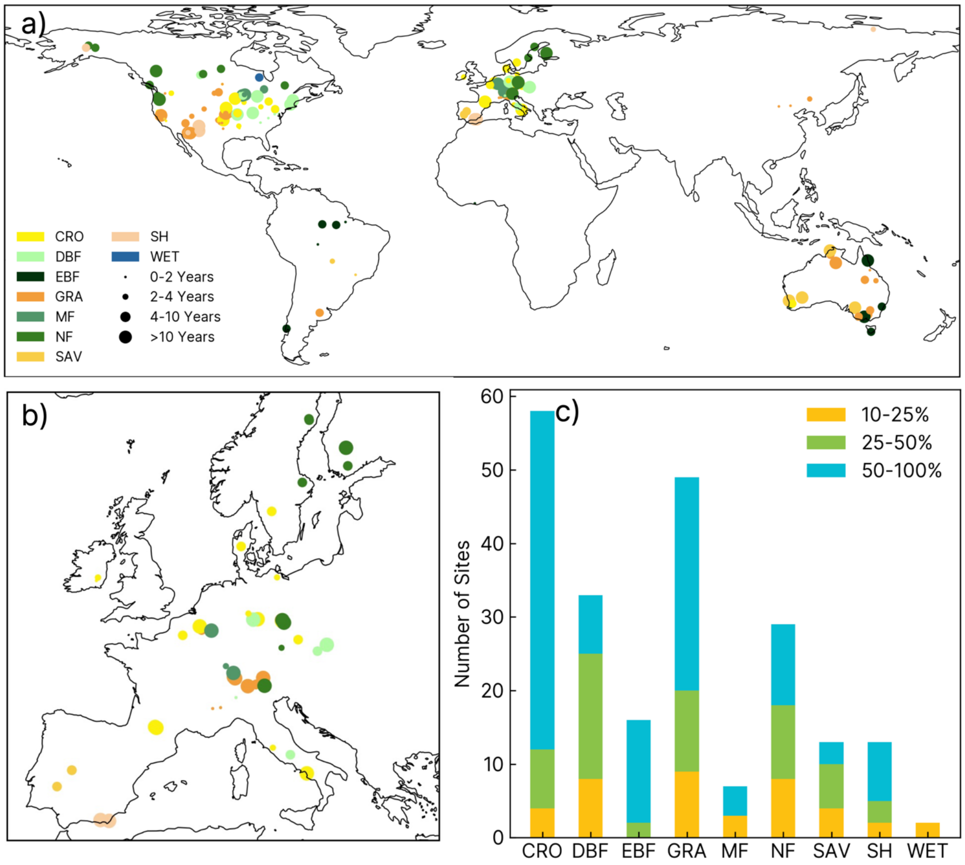

Fig. S33 | a) Spatial distribution of eddy covariance sites used in this study. Marker size indicates the length of available GPP observations at each site. b) Zoomed-in view of sites located in Europe. c) Distribution of sites by vegetation type. Colors indicate spatial homogeneity, defined as the fraction of the 0.25º grid cell that shares the same vegetation type as reported at the site level, categorized into three classes.

We found that applying different filtering thresholds or not assimilating eddy covariance data do not change the main conclusion of our study that incorporating spatial predictors improves the overall predictive performance of the model on various ecological variables (Fig. S34). These results suggest the general spatial gradient of the environmental variables is mostly constrained by remote sensing and atmospheric inversion variables, rather than the sparse eddy covariance data.

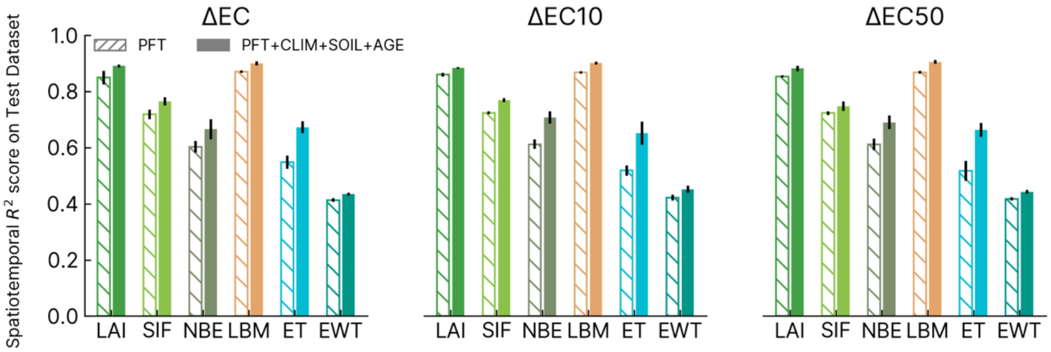

Fig. S34 | Sensitivity of the PFT and PFT+CLIM+SOIL+AGE configurations to different filtering thresholds for the spatial representativeness of eddy covariance data.

Eddy covariance data play a critical role in constraining the absolute magnitudes of ecosystem gross carbon uptake (GPP) and respiration (RECO) (Fig. S35). Without assimilation of eddy covariance observations (ΔEC), the DifferLand ensemble simulates a global GPP of 91.9 ± 27.8 PgC yr^{-1} and a global RECO of 88.2 ± 27.8 PgC yr^{-1} during 2003–2023. By contrast, under the default configuration with eddy covariance data assimilated, the model produces a global GPP of 136.7 ± 13.8 PgC yr^{-1} and RECO of 133.0 ± 13.6 PgC yr^{-1}. These values are consistent with independent benchmarks, including machine-learning–based upscaling of site-level GPP (e.g., FLUXCOM X-BASE, 125 ± 2.1 PgC yr^{-1}) and SIF-based global GPP estimates (135.5 ± 8.8 PgC yr^{-1})(Xing Li & Xiao, 2019).

The largest discrepancies between ΔEC and the default runs occur in equatorial regions: with assimilation, GPP and RECO reach ~7 gC m^{-2} day^{-1}, compared to only ~4 gC m^{-2} day^{-1} without assimilation. Site-level evaluations in tropical forests confirm that omitting eddy covariance constraints leads to systematic underestimation of both fluxes. This outcome is expected because tropical forests typically exhibit near-zero net carbon balance and weak seasonal cycle (e.g., Fig. S30d) despite high rates of photosynthesis and respiration; without point-based flux measurements, the model has little information to constrain the true magnitudes of these component processes. We note that differences among eddy covariance assimilation setups are relatively small for ET (Fig. S35), as its latitudinal gradient is already partially constrained by the assimilated GLEAM ET. Nonetheless, model runs assimilating site-level ET generally simulate lower values than those relying solely on gridded products.

Varying the filtering thresholds for site spatial representativeness had only a minor effect on simulated GPP and RECO, with global GPP estimates of 135.7 ± 8.2 PgC yr^{-1} under strict filtering (ΔEC50) and 139.6 ± 6.7 PgC yr^{-1} under more relaxed criteria (ΔEC10). This suggests that spatial mismatches between site footprints and model grid cells exert only a limited influence on global estimates. Nonetheless, future

work should pursue more effective multiscale data-assimilation strategies and evaluate whether the inferred environment–trait relationships are consistent across canopy, ecosystem, and biome scales.

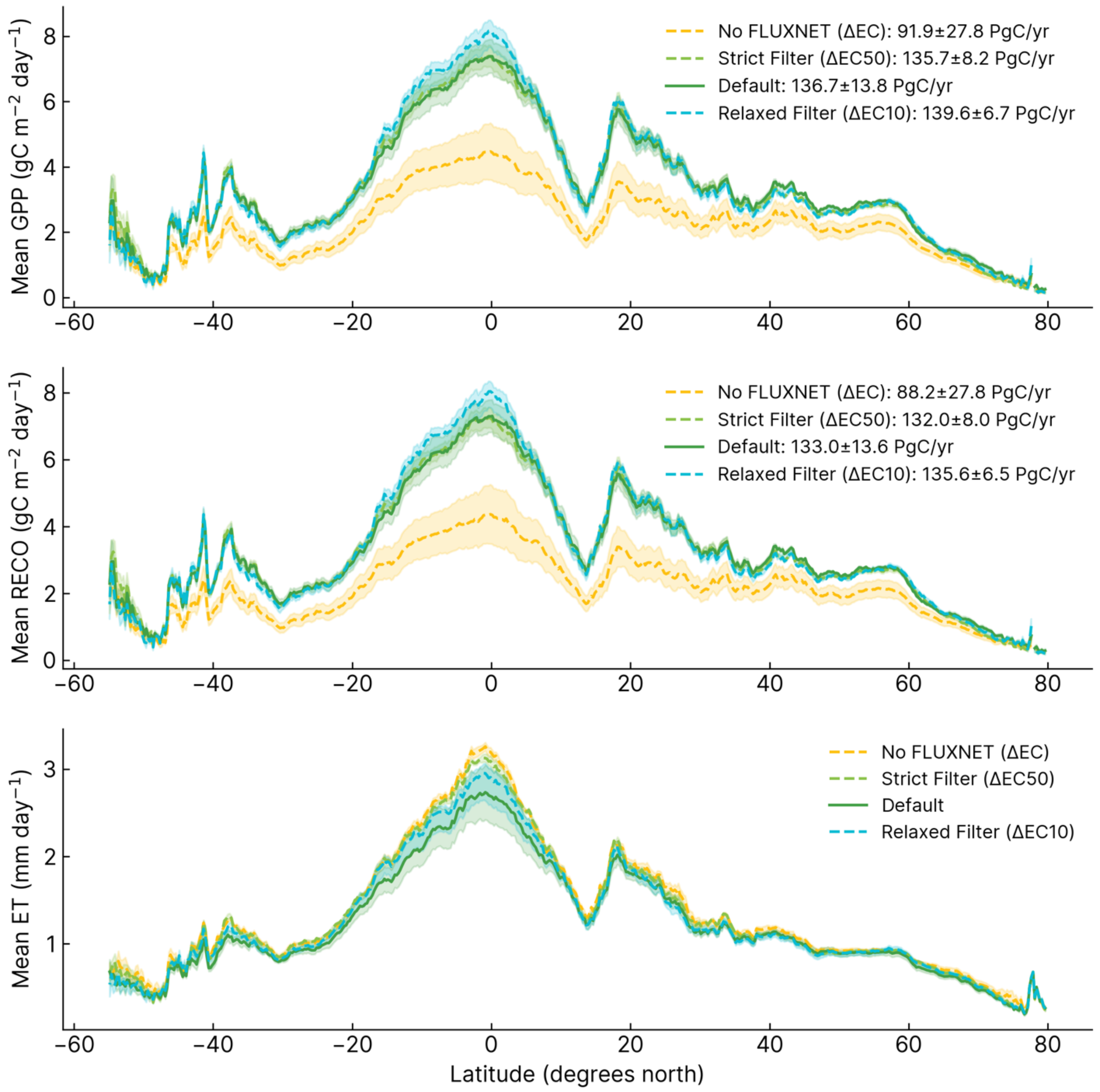

Fig. S43 | Latitudinal transects of mean GPP, RECO, and ET from model runs under four eddy covariance (EC) data assimilation configurations: no assimilation (ΔEC), a strict filtering criterion in which only sites whose reported plant type covers >50% of the overlapping grid cell are assimilated (ΔEC50, $N = 123$ sites), the default setup in which sites with >25% coverage are assimilated ($N = 180$ sites), and a relaxed criterion in which all sites with >10% coverage are assimilated (ΔEC10, $N = 218$ sites). Mean annual totals of GPP and RECO for each configuration are computed and labeled in the plots.

b.It must be noted SIF is NOT GPP; their decoupling (if using linear scaling) is most evident in the pan-tropics and high-latitude conifers. Details are lacking on how SIF was used to constrain parameters related to GPP.

Yes, we fully agree with the reviewer regarding the importance of not conflating SIF with GPP, and we acknowledge that there are clear instances of decoupling in the linear scaling between SIF and GPP, such as in the tropical forests and evergreen needleleaf forests (Pierrat et al., 2022). These varying relationships are most evident at the sub-diurnal timescale, due to strong variations in photosynthetically active radiation (PAR) and the light saturation of GPP (Pierrat et al., 2022; Zhang et al., 2016). However, the linearity between SIF and GPP generally increases with temporal and spatial coarse-graining (Zhang et al., 2016), as over longer aggregation periods the canopy structure signal in SIF and changes in light use efficiency (related to chlorophyll absorbed photosynthetically active radiation) dominates over the more transient and variable physiological component (Pierrat et al., 2022; P. Yang et al., 2023).

In our manuscript, we use a SIF-informed photosynthesis proxy (LCSPP) as an indirect constraint on GPP (Fang et al., 2025). Because LCSPP is assimilated at the monthly timescale, we expect the SIF–GPP relationship to be stronger. Previous tower-based evaluations at biweekly resolution show median R^2 values of ~0.4 in evergreen broadleaf forests and ~0.75 in needleleaf forests (Fig. R12). While an R^2 of 0.4 indicates a weaker linear relationship in tropical forests, we emphasize that this still provides a useful constraint on GPP, especially given the limited seasonality in many tropical ecosystems where traditional vegetation indices (e.g., NDVI, NIRv, kNDVI) often show little predictive power. Moreover, across biomes, LCSPP demonstrates stronger correlations with GPP than these commonly used indices. Accordingly, we employ a linear observation operator between LCSPP and GPP, while treating both the slope and intercept as model parameters to be calibrated during training, with priors informed by empirical SIF–GPP relationships at tower sites. This balances the parsimony needed for global assimilation with sufficient flexibility to capture ecosystem differences (e.g., between C_3 and C_4 vegetation).

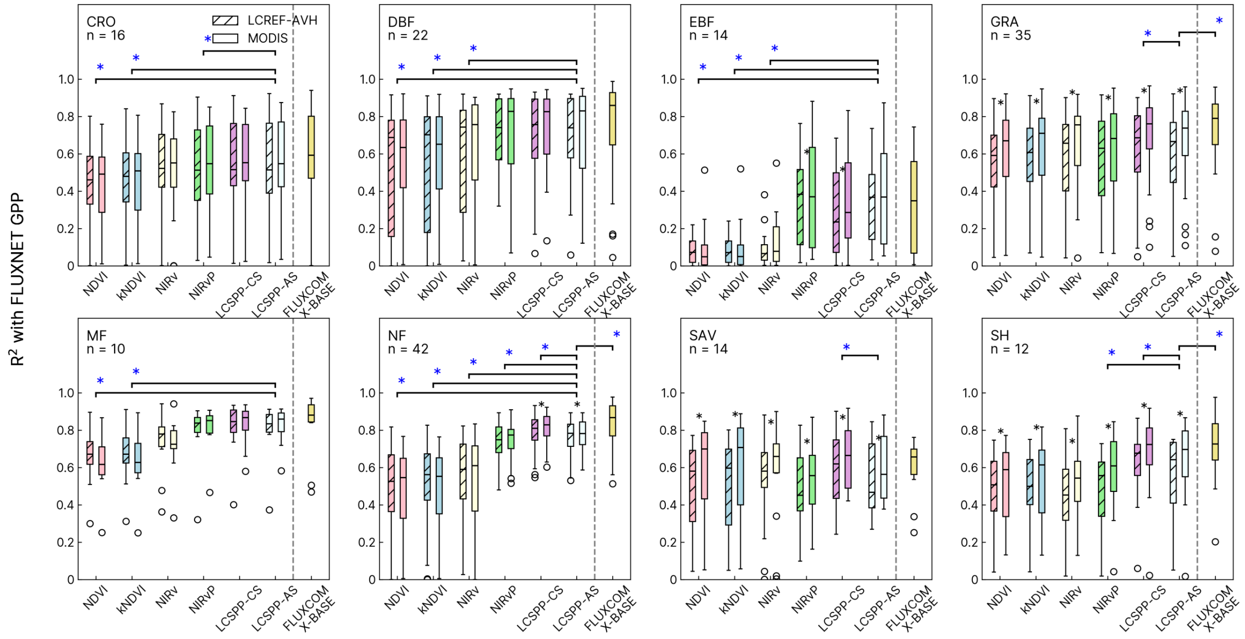

Fig. R12, adapted from Figure S12 from Fang et al. 2025: Comparing the strength of the linear relationships between LCSPP and site-level GPP across different plant types with other commonly used vegetation indices. Boxes and whiskers show the distribution across sites within the specified plant type. The MODIS based LCSPP-AS, with higher correlation with site-level GPP measurements at biweekly temporal resolution, is assimilated into DifferLand.

c.Details are lacking on how NEE was determined from CMS NBE products.

Thanks a lot for raising this clarifying question. We have included the details in the revised method section.

Line 1075-1086: Monthly NBE from the NASA Carbon Monitoring System Flux (CMS-Flux) GCP 2023 submission (Friedlingstein et al., 2023; J. Liu, 2024; J. Liu et al., 2021) was used to constrain DifferLand modeled NBE from 2010-2022. CMS-Flux is a top-down flux inversion system constrained by column CO_2 observations from the Greenhouse Gases Observing Satellite (GOSAT) and Observing Carbon Observatory-2 (OCO-2), which have broader and more even spatial coverage compared with ground-based CO_2 observations(J. Liu et al., 2014, 2017). Due to the computational cost of the atmospheric transport model, CMS-Flux has a native resolution of 4°×5°. We derived NBE in DifferLand as the sum of (negative) gross primary productivity (GPP), ecosystem respiration (RECO, which is itself the sum of autotrophic respiration, R_a, and heterotrophic respiration, R_h) and carbon emission from fire (F_{fire}). The first three terms constitute net ecosystem exchange (NEE), whereas fire carbon emission was additionally

constrained by the GFED5 dataset. A negative NBE represents a net flux of carbon from the atmosphere to the land (i.e., land carbon sink).

$$NBE = -GPP + R_a + R_h + F_{fire} = -GPP + RECO + F_{fire} = NEE + F_{fire}$$

d.Fig 3: I guess the impact of MAT has already been considered in current TBMs. For example, Vcmax25 is a parameter, but Vcmax depends on temperature variations. This means impact of local climate on parameters have already been incorporated in TBMs. So DifferLand identified additional MAT impact?

Thank you very much for raising this important point. We agree that most TBMs using the Farquhar photosynthesis formulation account for the short-term temperature dependence of Rubisco activity by standardizing V_{cmax} to 25 °C (V_{cmax25}) and then applying an Arrhenius-type function. This approach isolates an "intrinsic" trait that facilitates comparison across plant types. However, V_{cmax25} itself may acclimate to long-term growth temperature and other environmental gradients—a dimension that many TBMs neglect when treating V_{cmax25} as fixed within a PFT.

In our framework, the canopy photosynthesis module is a simplified aggregate model, where the retrieved "canopy efficiency" parameter plays a role analogous to V_{cmax25} but reflects nitrogen-limited photosynthetic capacity at the canopy scale. The temperature dependence we identify (now Fig. 4a) does not represent the short-term enzymatic limitation of Rubisco, but rather the long-term acclimation of photosynthetic efficiency to climatological mean annual temperature. By capturing this acclimation, DifferLand highlights an additional source of parameter variation along environmental gradients that is typically missing in TBMs assuming fixed V_{cmax25}.

e.Fig S21 and Fig. S22: It'd be great to enlarge the spatial maps of parameters. Just eyeballing of the small maps, there are not too much differences of these parameters from PFT-specific values.

Thank you very much for the suggestion. We have revised the manuscript to include enlarged maps on the retrieved residence times and allocation fractions (Fig. R13 and Fig. R14). Importantly, we emphasize that these maps of ecological parameters are not prescribed a priori, but rather emerge as latent variables inferred from the assimilation of vegetation states and fluxes. While it is expected that many parameters reflect plant functional type (PFT) distributions—for example, grasslands allocating less carbon to woody biomass than forests—the retrieved parameters also capture substantial within-PFT variation. This is evident in patterns such as the temperature dependence of autotrophic respiration and root allocation,

which do not collapse into uniform values even within regions dominated by a single vegetation type (Fig. 4).

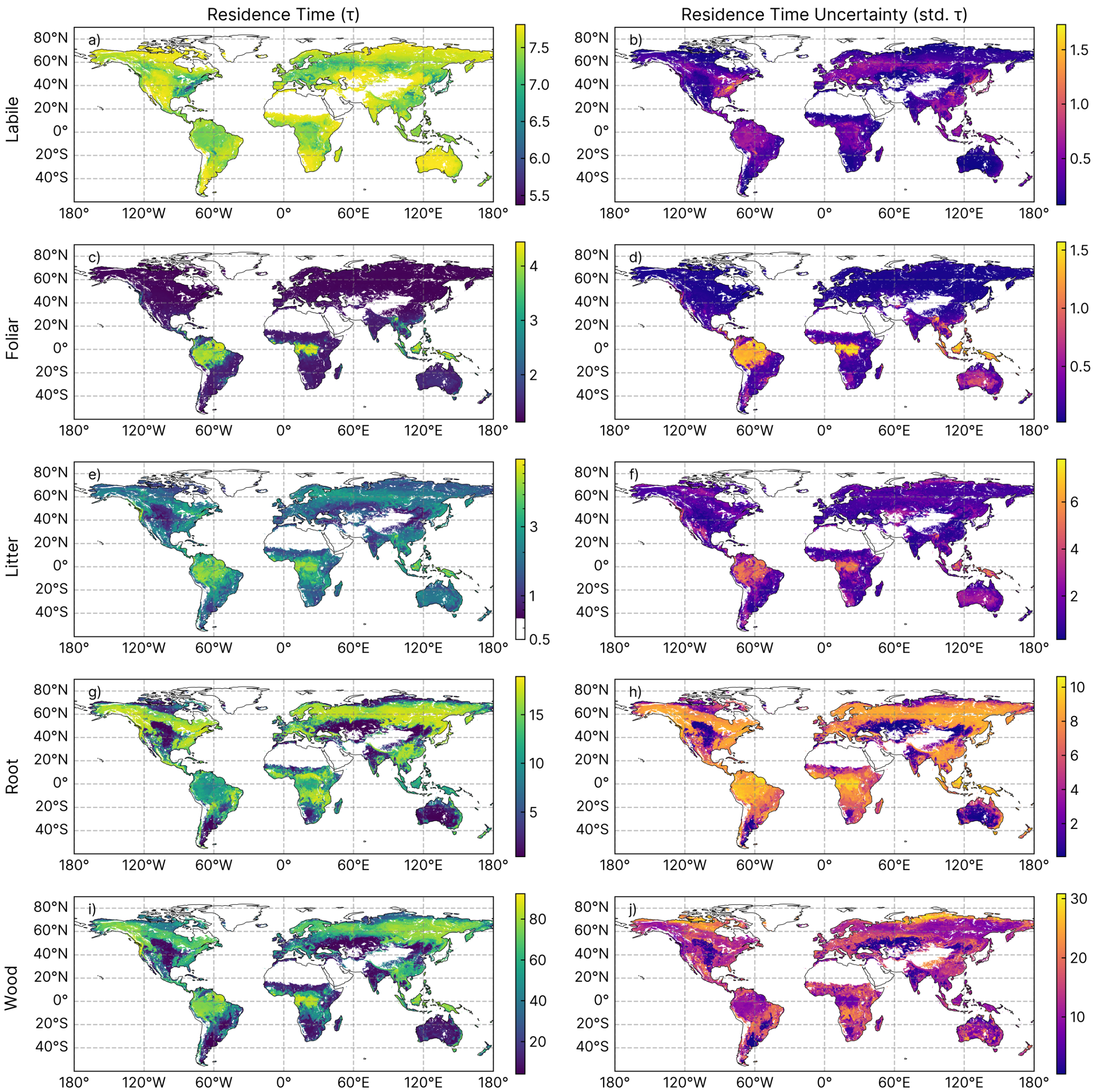

Fig. R13 (Fig. S40) | The mean residence time (τ) of different carbon pools across 10 out of the 20 ensembles of the PFT+CLIM+SOIL+AGE configuration that achieved the lowest training loss. The ensemble mean values are plotted in the first column, and the ensemble standard deviation are plotted in the second column.

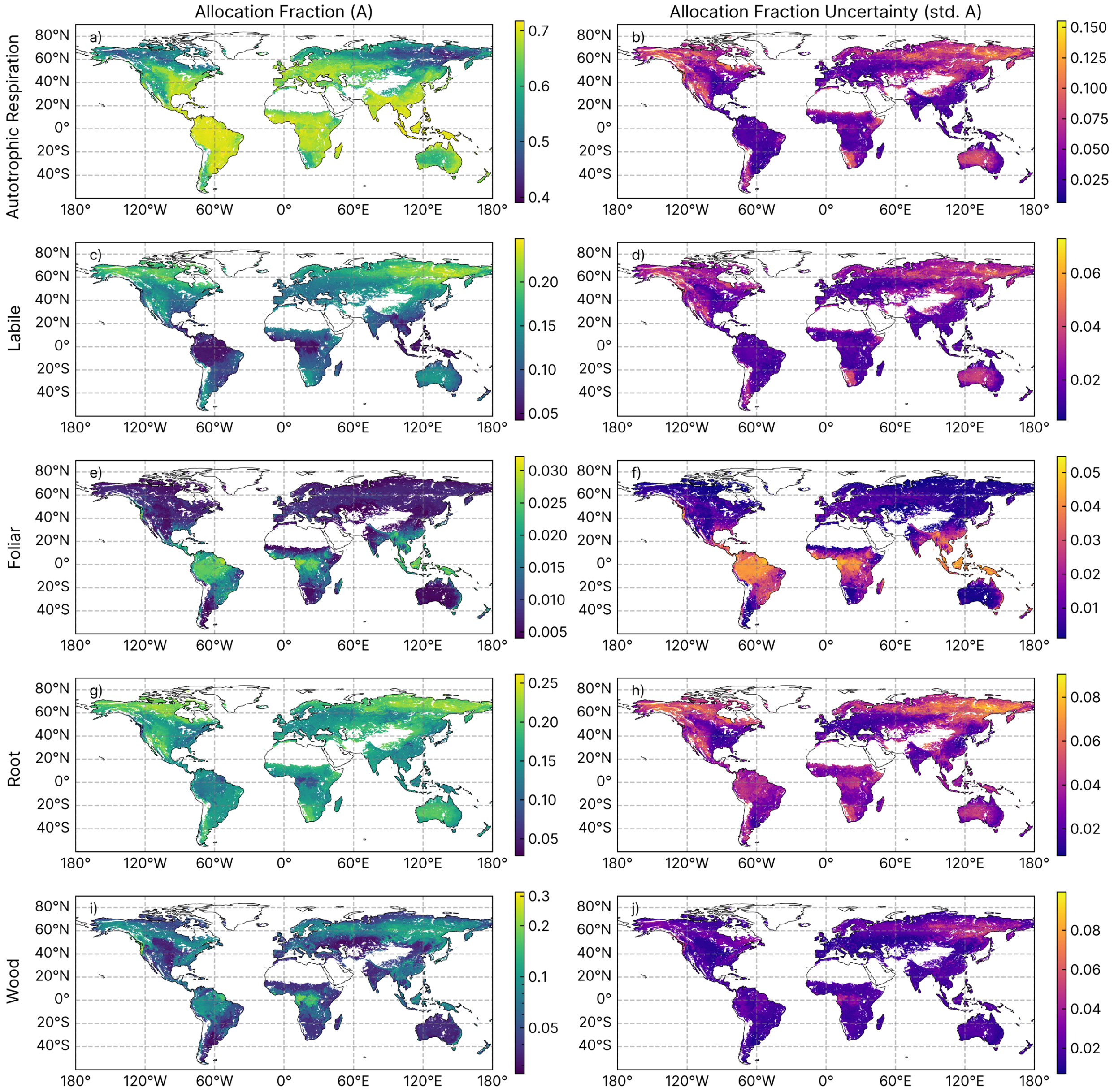

Fig. R14 (Fig. S41) | The allocation fraction (A) of different carbon pools across 10 out of the 20 ensembles of the PFT+CLIM+SOIL+AGE configuration that achieved the lowest training loss. The ensemble mean values are plotted in the first column, and the ensemble standard deviation are plotted in the second column.

Reviewer #3 (Remarks to the Author):

I co-reviewed this manuscript with one of the reviewers who provided the listed reports. This is part of the Nature Communications initiative to facilitate training in peer review and to provide appropriate recognition for Early Career Researchers who co-review manuscripts.
Thank you for your review and advice, which has greatly helped us improve our manuscript!

Reviewer #4 (Remarks to the Author):

Comments:
The manuscript "Differentiable Land Model Reveals Global Environmental Controls on Latent Ecological Functions" presents a hybrid modeling framework that combines process-based and machine-learning approaches to infer ecological parameters and their environmental controls. The study aims to improve upon traditional plant functional type (PFT)-based parameterizations by integrating diverse Earth observations and leveraging a differentiable land model. While the methodology is novel and technically sophisticated, the manuscript places a strong emphasis on modeling techniques rather than providing new ecological or climate science insights. Given this focus, the study may be better suited for a specialized modeling journal rather than Nature Communications, which prioritizes broader conceptual advancements with broad interdisciplinary appeal.

We appreciate the reviewer's thoughtful comments and recognition of the methodological novelty and technical sophistication of our study. While it is true that the manuscript introduces a new hybrid modeling framework, we respectfully disagree with the characterization that our work is primarily a technical contribution with limited ecological or climate science insight.

A major ecological discovery presented in our manuscript is the **characterization of the spatial coordination of plant traits as an emergent property of global biogeographical gradients.** Leveraging the coordinated adaptation and tradeoffs of plant traits to environmental conditions, our study demonstrates that the decadal responses of ecosystem fluxes and stocks to dynamic forcing variables can be effectively captured by a few leading spatial dimensions. Notably, our approach does not require the prior prescription of optimality principles between ecological properties and each environmental gradient. Instead, these coordination patterns **emerge as latent properties (emergent behavior)** of the ecosystem, revealed through the assimilation of global ecological observations within a flexible hybrid machine learning framework.

From a theoretical perspective, the trait–environment relationships embedded in DifferLand bridge the spatial legacy of plant adaptation to prevailing climatology and land use patterns with the more rapid, dynamic responses of ecosystems to changing forcings on sub-seasonal to interannual timescales. Thus, our model is not only a technical advance but also offers **a novel perspective by linking the slow and fast processes that govern the terrestrial biosphere**—revealing how plant trait-based ecological functions capture the enduring imprint of environmental history within a dynamic, ever-changing system.

On the practical front, our results resolve **a long-standing challenge that has impeded the widespread adoption of trait-based modeling in ecology**. While ecologists have long recognized the fundamental importance of capturing spatially heterogeneous plant adaptations, the inclusion of additional environmental gradients—such as topography, soil texture, forest age, disturbance regimes, and local climate—has traditionally led to a combinatorial explosion in the number of parameters required by land surface models. The sheer scale and complexity of this parameter space have made it virtually impossible to systematically measure and calibrate the necessary traits for every species across all environmental niches.

This predicament has left the field with a seemingly insurmountable dilemma: must we measure every plant trait for every species along every conceivable environmental gradient to accurately represent adaptation and forecast terrestrial biosphere responses? Our study offers a breakthrough by demonstrating that a small number of leading environmental dimensions can explain most of the spatial variability in ecological functions that govern land carbon fluxes and states over multi-decadal timescales. This finding reveals that **plant trait coordination in response to environmental conditions provides a powerful source of predictability**. As such, our work not only affirms the feasibility of trait-based modeling through environmental–trait relationships, but also clarifies why this approach is so effective for overcoming the parameterization challenge in terrestrial ecosystem modeling.

Using interpretable machine learning, we further demonstrated that **key ecological parameters—such as photosynthetic efficiency and carbon use efficiency—exhibit divergent sensitivities to temperature gradients across plant types, which differ from the apparent global average sensitivity**. These biome-specific patterns have important implications for ecosystem responses to anthropogenic climate change. On one hand, the existing distribution of plant types may buffer ecological shifts, as dispersal limitations can constrain rapid reorganization. On the other hand, stronger regional sensitivities of photosynthetic and carbon use efficiencies to temperature in certain biomes, such as Arctic ecosystems, may amplify ecological responses to warming. While we acknowledge that these analyses may involve uncertainties in changing ecological functions, we believe they provide a novel conceptual lens for understanding biome-

specific trait–environment relationships—as both an ecological legacy and a potential driver of change under anthropogenic climate forcing.

Beyond its ecological and climate science applications, our work represents a significant advancement in computational science by **demonstrating how artificial intelligence and process-based models can be combined to uncover new scientific principles**. This methodology is likely to inspire researchers working on physics-informed machine learning across various disciplines, as evidenced by the impact of recent frameworks such as NeuralGCM. To further support the community, we have revised our code to be modular and user-friendly and crafted detailed documentations and tutorials, facilitating its adoption as a framework for testing new scientific hypotheses. Our framework has potential to improve the representation of ecosystem processes in climate models, thus informing broader questions at the intersection of ecology, climate science, and Earth system modeling. We believe that this conceptual advancement and its relevance to global change research align well with the interdisciplinary scope and readership of *Nature Communications*.

We do agree with the reviewer that our initial submission might place too much emphasis on the technical details and might not fully explicate the scientific implications of our work. In our revised manuscript, we have carefully restructured the content to better highlight the scientific insights and relevance of our findings for a broad audience. We also improve the model schematics (Fig. 1) to provide a more conceptual overview of our framework. We hope the reviewer will reconsider the broader scientific significance of our work in light of these contributions and improvements. Below please find our detailed response to each comment raised by the reviewer.

The study effectively demonstrates that PFTs alone explain less than half of the spatial variability in ecological parameters controlling carbon fluxes and vegetation states. However, a more direct comparison with real-world observations is needed to support the validity of the model's inferred relationships. The brief discussion of R^2 values (Lines 138–146) lacks clarity on whether these statistics represent spatial or temporal variability, or whether they compare an ensemble mean across time with multiple field datasets. Including direct field comparisons, such as those presented in Figures S10 and S11, in the main text would enhance the credibility of the model's performance.

We thank the reviewer for this constructive comment. In the revised manuscript, we have substantially expanded the range of eddy covariance observations used for evaluation by incorporating a large number of additional sites from the ICOS, LBA-ECA, and OzFlux networks (Fig. S33). This expansion allows for a more rigorous assessment of the model against real-world observations (Fig. S19, Fig. S20, and Fig.

S21), particularly in tropical forests and Australia, where direct observational constraints were previously limited.

We have also clarified in the text that the reported R^2 values are based on combined spatial and temporal variability (see Fig. S15 and Fig. S16 for detailed performance metrics for different model configurations). Furthermore, we now provide more detailed evaluations of model performance at global, regional, and pixel levels, including assessments of both total temporal variability (Fig. S13) and interannual anomalies against benchmark datasets (Fig. S14). To further contextualize performance, we compared DifferLand's simulated NBE with that of several state-of-the-art DGVMs (Fig. S8 & Fig. S9), thereby demonstrating how the combination of multi-stream data assimilation and latent trait–environment relationships improve predictions of net biosphere exchange.

While many of these results are presented in the Supplementary Information, our primary goal in the main text is to highlight the broader scientific questions that the differentiable approach enables (as noted in response to the reviewer's first point), rather than to frame this work solely as a model description study. Nevertheless, we recognize the importance of rigorous evaluation, and have ensured that these analyses are thoroughly documented in the SI and explicitly referenced in the main text.

Furthermore, the manuscript would benefit from clearer distinctions between different sources of variability in the presented results. For example, the discussion in Line 228 mentions retrieved carbon use efficiency (CUE) values and independent site-level measurements, but it is unclear how these datasets were aggregated or how model performance is being evaluated. The interpretation of hybrid model outputs would be strengthened by explicitly defining whether specific results reflect training data variance, full predictor hybrid model performance, or validation against independent field observations.

We thank the reviewer for this helpful observation. In the revised manuscript, we have taken care to more clearly distinguish between different sources of variability and the basis for model evaluation. Specifically, we now explicitly define whether results reflect training variability across ensembles, hybrid model performance using the full predictor set, or independent validation against field observations. For the example of carbon use efficiency (CUE), we have expanded the figure caption of Fig. S39 to detail the data sources and aggregation procedure: *"For DifferLand, we first computed the ensemble mean NPP:GPP ratio across 10 out of the 20 ensembles of the PFT+CLIM+SOIL+AGE configuration that achieved the lowest training loss, and then derived the per-biome spatial distribution using WWF biome maps. For MODIS, we derived the mean NPP and GPP values from MOD17A3HGF.061 between 2010*

and 2023, and computed the per-biome distribution. For site observations, the results are obtained from Collati & Prentice (2019) by aggregating site-level studies based on the biome classification presented in the paper.” These clarifications ensure that the reader can distinguish the relative contributions of model ensemble variability, satellite product comparisons, and independent *in-situ* benchmarks to the interpretation of the retrieved CUE values.

The discussion of parameter importance is interesting but requires further elaboration. Figures S14 and S16 suggest that adding climate (CLIM) parameters improves model performance beyond PFT-based parameterizations, while additional variables (SOIL, AGE) provide diminishing returns. However, Figures S18 and 2f indicate that AGE and SOIL still contribute significantly in certain contexts. A more detailed analysis of these spatial variations—such as region-specific differences in parameter importance—would be valuable in clarifying these results. Additionally, the study notes that the hybrid model has lower predictive power for water cycle variables (ET, EWT), but the manuscript does not fully explore potential reasons for this limitation. Given the importance of water availability in future climate projections, discussing how the model handles moisture-related processes and whether additional constraints could improve performance would be worthwhile.

We thank the reviewer for this insightful suggestion. In the revised manuscript, we have expanded our analysis to better contextualize the roles of AGE and SOIL predictors. Specifically, in the updated Figure 2 we improved the symbology to highlight the dominant predictor group at each pixel and added an inset summarizing the fraction of global land area where each group is most important. This analysis shows that while CLIM predictors dominate in mid-latitude herbaceous biomes with strong climatological gradients (e.g., the Eurasian Steppe and North American Great Plains), AGE variables such as forest age and maximum canopy height provide critical explanatory power in perennial systems and mixed tree–grass biomes. SOIL variables, although less influential for carbon cycle processes, emerge as key predictors of water cycle dynamics (ET and EWT), particularly in sparsely vegetated regions where soil water storage plays a stronger role than canopy processes. These refinements clarify that AGE and SOIL predictors contribute important region-specific information that complements PFT- and CLIM-based parameterizations.

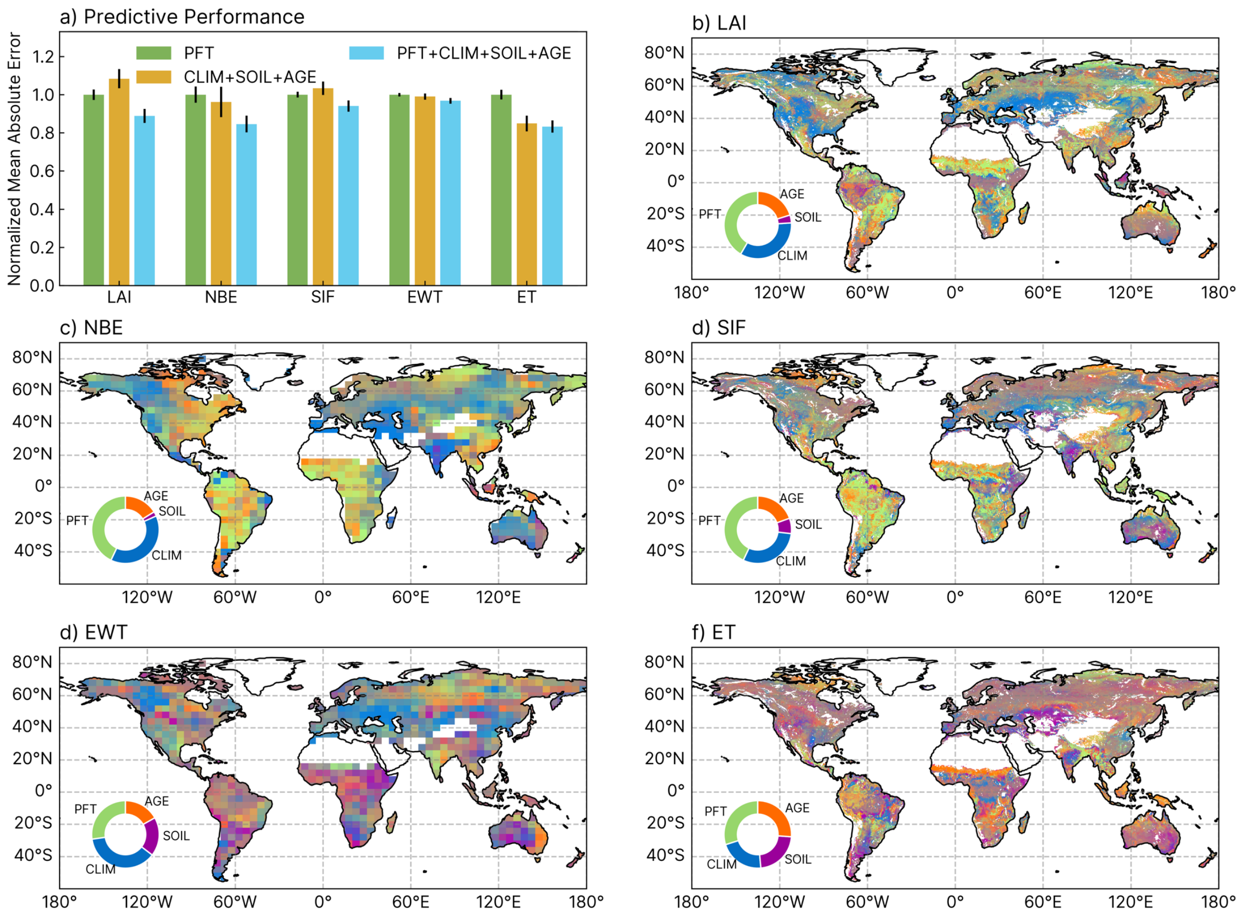

Fig. 2 | Hierarchical partitioning of explained variance by four predictors groups. Panel a) compares the mean absolute error on held-out pixels for LAI, NBE, SIF, EWT, and ET across three model configurations: PFT-only, environmental variables only (CLIM+SOIL+AGE), and combined PFT and environmental variables (PFT+CLIM+SOIL+AGE). Errors are normalized relative to the PFT-only configuration. Panels b-f) show the proportion of temporal variance explained in LAI, NBE, SIF, EWT, and ET within each grid cell, attributed to the model using ecological parameters predicted from spatial information in PFT, CLIM, AGE, and SOIL variables, respectively. The inset shows the proportion of pixels where each predictor group (PFT or environmental variables) has the dominant effect.

We also appreciate the reviewer's point about the lower predictive performance for water cycle variables. In the test set, the model achieves a total (spatiotemporal) R^2 of 0.68 for ET and 0.45 for EWT, somewhat lower than for carbon fluxes. We attribute this to three factors: (1) the relatively simple representation of evapotranspiration in the DALEC model, which assumes a fixed water-use efficiency formulation and

does not fully capture dynamic stomatal responses to meteorological variability; (2) scale mismatches between the modeled soil-bucket water storage and the GRACE-based equivalent water thickness (EWT), which integrates deeper groundwater, open water bodies and large-scale hydrological processes; and (3) the lack of explicit representation of vertical soil moisture transport and groundwater dynamics, as well as open water bodies in DALEC; (4) model uncertainties in the target datasets (GLEAM).

We agree with the reviewer that water availability is critical for future climate predictions. To this end, we note that future versions of hybrid modeling frameworks should incorporate: (1) more mechanistic stomatal conductance and transpiration formulations, potentially using hybrid-ML parameterizations to capture short-term responses to meteorological drivers; (2) multi-layer soil hydrology and plant-unavailable water pools; and (3) additional watershed-scale constraints, such as streamflow from gauge networks, to better anchor total water storage estimates. We have added discussion of these points in the revised manuscript (Text S7) to highlight potential pathways for improving the representation of moisture-related processes in hybrid land models.

Despite strong overall performance, the hybrid DALEC framework shows more reduced skill for water cycle variables (spatiotemporal R^2 = 0.68 for ET and 0.45 for EWT on test pixels). This likely reflects the simplified formulation of evapotranspiration based on fixed water-use efficiency, which cannot capture dynamic stomatal regulation under variable meteorological conditions. Additional mismatches arise because GRACE-derived equivalent water thickness integrates deep groundwater and large-scale hydrological processes that are absent from the DALEC soil-bucket scheme, and because vertical soil moisture transport and groundwater dynamics are not explicitly modeled. Potential improvements include more mechanistic stomatal and transpiration formulations (potentially supported by hybrid-ML approaches), multi-layer soil hydrology distinguishing plant-available versus unavailable water pools, and watershed-scale constraints such as streamflow to better anchor modeled water balance.

The study's methodological framework relies on four spatiotemporal parameter groups (PFT, CLIM, SOIL, AGE), which appear to have relatively low temporal variability. This could partially explain why the "Forcing and Structure" group (which includes daily temperature and precipitation) accounts for a large fraction of spatiotemporal variance (Line 190). Clarifying whether the hybrid model operates at a daily timestep or whether forcing variables are temporally aggregated to match the resolution of parameter groups would help resolve this issue.
We thank the reviewer for this important comment and acknowledge that our original manuscript did not fully clarify the respective roles of the spatial predictors and forcing variables. In our framework, the four

predictor groups (PFT, CLIM, SOIL, AGE) refer to *static spatial environmental inputs* to the spatialization network. These variables are assumed to capture long-term plant acclimation and adaptation to prevailing environmental conditions and are therefore treated as temporally invariant within the decadal timescales considered here. The spatialization network uses these predictors to estimate ecological parameters that vary across space but remain fixed in time for each grid cell.

By contrast, the forcing variables—including monthly averaged daily minimum and maximum temperature, total precipitation, vapor pressure deficit, shortwave radiation, and atmospheric CO_2 concentration—are *dynamic inputs* that evolve monthly. These forcing variables drive the process-based model forward in time as a dynamical system, integrating ecosystem states (carbon and water pools) at each grid cell (Fig. S4).

We also note that in the original manuscript we separated the contributions of "model structure and forcing" from those of the spatial predictor groups in the hierarchical partitioning, treating the former as the model performance when using a null set of non-informative predictors (white noise). Upon reevaluation, however, we realized that this artificial separation may neglect interactions between the spatial predictors and the model structure. In an end-to-end differentiable framework such as DifferLand, information from spatial predictors ultimately influences simulated ecological fluxes only through its interaction with the dynamic forcing. To resolve this, we redefined the baseline for hierarchical partitioning using a default pixelwise correlation of 0. The variance is now fully reallocated to the four predictor groups, which sum to 100% for each variable, thereby avoiding the confusion caused by the earlier formulation.

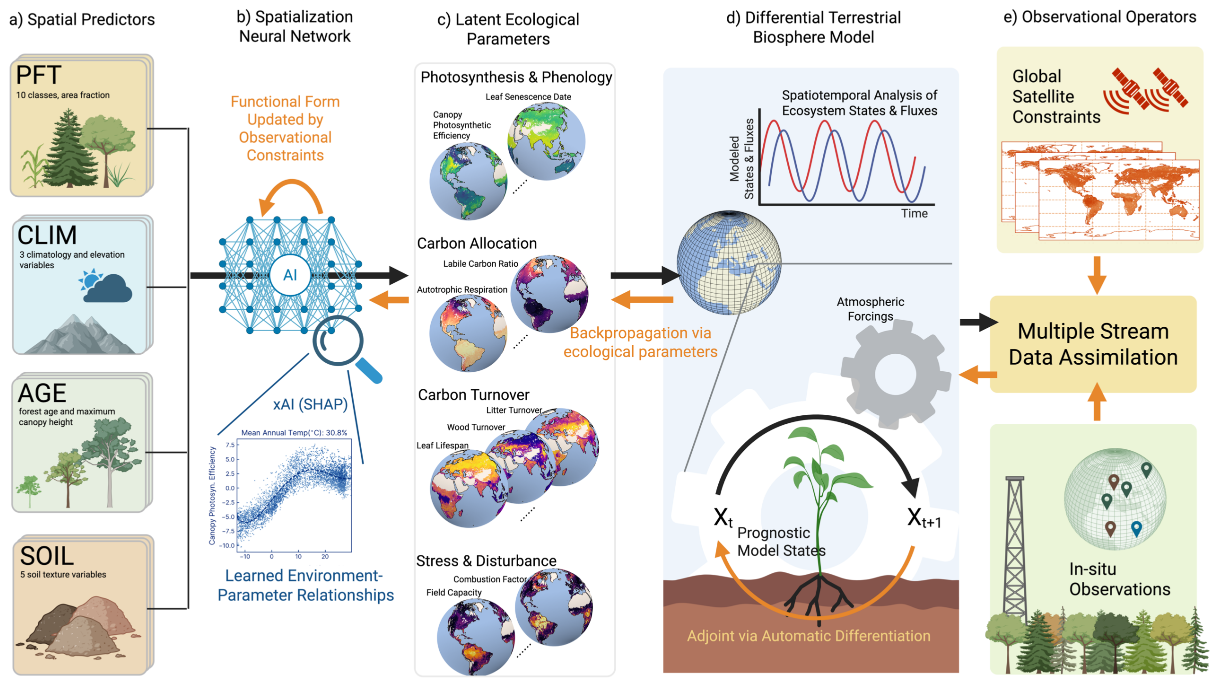

Fig. 1 | Schematics of the DifferLand framework. a) DifferLand integrates spatial information in four groups environmental predictors, including plant functional types (PFTs), climate variables (CLIM), forest age and maximum height (AGE), and soil texture (SOIL). b) During a forward pass (black arrows), a spatialization neural network models trait–environment relationships, mapping these predictors to c) global fields of 40 ecological parameters representing plant functional traits such as photosynthesis, carbon allocation, turnover rates, and sensitivity to drought and fire. d) A terrestrial biosphere model uses these parameters to simulate ecosystem states and fluxes under meteorological forcing, where X_t and X_{t+1} represents the evolution of model state from one time step to the next. e) DifferLand evaluates the simulated vegetation dynamics against multiple streams of satellite and *in-situ*-observations, and it computes the sensitivity of simulated vegetation dynamics to trait-based ecological parameters through its differentiable structure (orange arrows). The gradient information is used to update the parameters within the spatialization neural network and iteratively optimize the learned trait-environment relationships.

To clarify these design choices, we have revised Fig. 1 to provide a high-level overview of the DifferLand framework, showing the flow from static spatial predictors to parameter estimation, and then to the process-based model. We have also added a worked example at a representative pixel in Finland to illustrate the internal mechanism of the framework (Fig. S4). We hope these revisions more clearly explain the structural design and address the reviewer's concern.

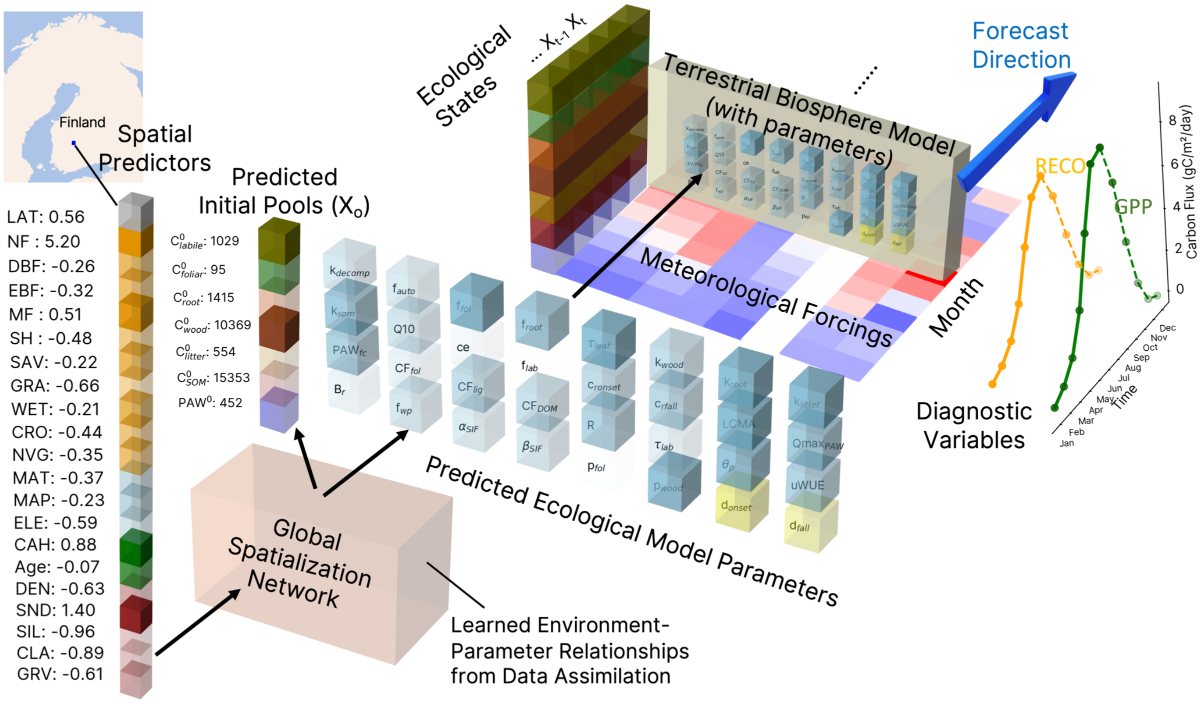

Fig. S4 | Example of the internal data flow through DifferLand for a grid cell in central Finland. A vector of standardized spatial predictors—including PFT fractions, climatological variables, forest age, maximum canopy height, and soil texture properties—is fed into the spatialization neural network to predict both the initial ecosystem states at the start of the simulation and the ecological parameters specific to the grid cell. These predicted ecological parameters are then used to parameterize the differentiable terrestrial biosphere model. The model's differential equations are integrated forward in time, driven by monthly meteorological forcings, to update ecosystem states and simulate monthly carbon and water exchanges within the grid cell. This process is repeated at each grid cell across the global land surface.

Additional technical refinements would also improve clarity. For example, Figure 2 should explicitly state whether it represents an aggregate over the entire time period (2010–2023), and the omission of ET from panel 2f should be confirmed as intentional or corrected. The manuscript also contains minor grammatical and typographical errors (e.g., Line 62: "requires" should be "require"; Line 241: "level-level" should likely be "leaf-level"), which should be addressed in revision.

We thank the reviewer for these constructive suggestions. In the revised manuscript, we have clarified the caption of Fig. 2a to specify that the mean absolute error (MAE) comparison between the PFT-only configuration and the CLIM+SOIL+AGE and PFT+CLIM+SOIL+AGE configurations is based on the total spatial and temporal model–data mismatch over 2003–2023 (with the simulation period extended for

a longer-term evaluation). We use normalized spatial–temporal MAE as it provides a single aggregate metric to evaluate overall model performance and allows direct comparisons across configurations and variables.

In contrast, Fig. 2b–f now focuses on hierarchical partitioning of predictor group contributions using the pixel-level temporal correlation coefficient (2003–2023). This pixelwise metric highlights the regional variation in the relative importance of different predictor groups.

The omission of ET in the original figure was inadvertent, and we have now included ET in the revised version. Following feedback from other reviewers, we also removed VOD from the default model run, as its inclusion provided only marginal benefits in constraining carbon pools and could introduce errors due to uncertainties in VOD–biomass relationships. Nonetheless, its effects are fully examined in a sensitivity analysis (SI Text S4).

Finally, we have carefully revised the manuscript to correct typographical and grammatical errors throughout.

Recommendation:
While the manuscript presents a technically rigorous modeling framework, its primary contributions lie in methodological advancements rather than broad conceptual or interdisciplinary insights. The study focuses heavily on the implementation and validation of a novel modeling approach rather than on generating new ecological or climate science findings with broad implications. Given this emphasis, I recommend rejection from Nature Communications but suggest resubmission to a more specialized journal focused on land surface modeling, machine learning applications in Earth system science, or geophysical research.
We thank the reviewer for their thoughtful feedback. While we appreciate the concern that the original submission may have emphasized technical implementation, we would like to stress that the revised manuscript goes significantly beyond methodological advancement. In the revision, we have carefully restructured the narrative to foreground the broad conceptual and interdisciplinary insights enabled by our framework. Specifically, the study reveals emergent axes of ecological organization that link plant functional traits, environmental gradients, and ecosystem fluxes; quantifies the relative contributions of global macroecological gradients versus plant-type specific adaptations in shaping ecosystem functioning; and provides a new pathway for integrating satellite and *in-situ* observations into land–climate models.

These findings extend well beyond technical novelty, offering new perspectives on fundamental ecological and climate science questions with direct implications for improving Earth system model predictions. We therefore respectfully believe that the manuscript does provide the type of conceptual and interdisciplinary contributions aligned with *Nature Communications*, and we hope the reviewer may be willing to reconsider their assessment in light of these substantial revisions.

Reviewer #5 (Remarks to the Author):

I co-reviewed this manuscript with one of the reviewers who provided the listed reports. This is part of the Nature Communications initiative to facilitate training in peer review and to provide appropriate recognition for Early Career Researchers who co-review manuscripts.

Thank you for your review and advice, which has greatly helped us improve our manuscript!

References

Bengio, Y., Courville, A., & Vincent, P. (2013). Representation Learning: A Review and New Perspectives. *IEEE Transactions on Pattern Analysis and Machine Intelligence*, *35*(8), 1798–1828. https://doi.org/10.1109/TPAMI.2013.50

Bloom, A. A., & Williams, M. (2015). Constraining ecosystem carbon dynamics in a data-limited world: Integrating ecological "common sense" in a model-data fusion framework. *Biogeosciences*, *12*(5), 1299–1315. https://doi.org/10.5194/bg-12-1299-2015

Bodnar, C., Bruinsma, W. P., Lucic, A., Stanley, M., Allen, A., Brandstetter, J., et al. (2025). A foundation model for the Earth system. *Nature*, *641*(8065), 1180–1187. https://doi.org/10.1038/s41586-025-09005-y

Bonan, G. B., & Doney, S. C. (2018). Climate, ecosystems, and planetary futures: The challenge to predict life in Earth system models. *Science*, *359*(6375), eaam8328. https://doi.org/10.1126/science.aam8328

Bowman, K. (2020). Carbon Monitoring System Carbon Flux for Fire L4 V2 [Data set]. Goddard Earth Sciences Data and Information Services Center (GES DISC). https://doi.org/10.5067/HO07ZJEQBMHE

Bowman, K., Cressie, N., Qu, X., & Hall, A. (2018). A Hierarchical Statistical Framework for Emergent Constraints: Application to Snow-Albedo Feedback. *Geophysical Research Letters*, *45*(23). https://doi.org/10.1029/2018GL080082

Brown, C. F., Kazmierski, M. R., Pasquarella, V. J., Rucklidge, W. J., Samsikova, M., Zhang, C., et al. (2025, July 29). AlphaEarth Foundations: An embedding field model for accurate and efficient global mapping from sparse label data. arXiv. https://doi.org/10.48550/arXiv.2507.22291

Chu, H., Luo, X., Ouyang, Z., Chan, W. S., Dengel, S., Biraud, S. C., et al. (2021). Representativeness of Eddy-Covariance flux footprints for areas surrounding AmeriFlux sites. *Agricultural and Forest Meteorology*, *301–302*, 108350. https://doi.org/10.1016/j.agrformet.2021.108350

Copernicus Atmosphere Monitoring Service. (2020). CAMS global inversion-optimised greenhouse gas fluxes and concentrations [Data set]. https://doi.org/10.24381/ed2851d2

Damgaard, C. (2019). A Critique of the Space-for-Time Substitution Practice in Community Ecology. *Trends in Ecology & Evolution*, *34*(5), 416–421. https://doi.org/10.1016/j.tree.2019.01.013

European Union's Copernicus Land Monitoring Service information. (2017a). Leaf Area Index 1999-2020 (raster 1 km), global, 10-daily – version 2 [Data set]. https://doi.org/10.2909/d5fdc595-2e03-4cbe-a39e-5f006f9cef07

European Union's Copernicus Land Monitoring Service information. (2017b). Leaf Area Index 2014-present (raster 300 m), global, 10-daily – version 1 [Data set]. https://doi.org/10.2909/219fdc9f-616b-444b-a495-198f527b4722

Fan, H., Bai, L., Fei, B., Xiao, Y., Chen, K., Liu, Y., et al. (2025, July 8). Physically Consistent Global Atmospheric Data Assimilation with Machine Learning in Latent Space. arXiv. https://doi.org/10.48550/arXiv.2502.02884

Fang, J., Lian, X., Ryu, Y., Jeong, S., Jiang, C., & Gentine, P. (2025). A long-term reconstruction of a global photosynthesis proxy over 1982–2023. *Scientific Data*, *12*(1), 372. https://doi.org/10.1038/s41597-025-04686-6

Fisher, R. A., & Koven, C. D. (2020). Perspectives on the Future of Land Surface Models and the Challenges of Representing Complex Terrestrial Systems. *Journal of Advances in Modeling Earth Systems*, *12*(4), e2018MS001453. https://doi.org/10.1029/2018MS001453

Franks, S. J., Weber, J. J., & Aitken, S. N. (2014). Evolutionary and plastic responses to climate change in terrestrial plant populations. *Evolutionary Applications*, *7*(1), 123–139. https://doi.org/10.1111/eva.12112

Friedlingstein, P., O'sullivan, M., Jones, M. W., Andrew, R. M., Bakker, D. C., Hauck, J., et al. (2023). Global carbon budget 2023. *Earth System Science Data*, *15*(12), 5301–5369.

Huang, Y., Song, X., Wang, Y.-P., Canadell, J. G., Luo, Y., Ciais, P., et al. (2024). Size, distribution, and vulnerability of the global soil inorganic carbon. *Science*, *384*(6692), 233–239. https://doi.org/10.1126/science.adi7918

Kelly, A. E., & Goulden, M. L. (2008). Rapid shifts in plant distribution with recent climate change. *Proceedings of the National Academy of Sciences*, *105*(33), 11823–11826. https://doi.org/10.1073/pnas.0802891105

Kochkov, D., Yuval, J., Langmore, I., Norgaard, P., Smith, J., Mooers, G., et al. (2024). Neural general circulation models for weather and climate. *Nature*. https://doi.org/10.1038/s41586-024-07744-y

Li, Xiaojun, Ciais, P., Frappart, F., Scipal, K., Fan, L., Yang, H., et al. (2025). IB-AGC: Annual 25 km global live biomass carbon product from SMOS L-band passive microwave vegetation optical depth. *Scientific Data*, *12*(1), 1156. https://doi.org/10.1038/s41597-025-05470-2

Li, Xing, & Xiao, J. (2019). Mapping Photosynthesis Solely from Solar-Induced Chlorophyll Fluorescence: A Global, Fine-Resolution Dataset of Gross Primary Production Derived from OCO-2. *Remote Sensing*, *11*(21), 2563. https://doi.org/10.3390/rs11212563

Liu, H., Mi, Z., Lin, L., Wang, Y., Zhang, Z., Zhang, F., et al. (2018). Shifting plant species composition in response to climate change stabilizes grassland primary production. *Proceedings of the National Academy of Sciences*, *115*(16), 4051–4056. https://doi.org/10.1073/pnas.1700299114

Liu, J. (2024). CMS-Flux GCP 2023 Submission [Data set]. Retrieved from https://cmsflux.jpl.nasa.gov/get-data/gcp-2023

Liu, J., Bowman, K. W., Lee, M., Henze, D. K., Bousserez, N., Brix, H., et al. (2014). Carbon monitoring system flux estimation and attribution: impact of ACOS-GOSAT XCO_2 sampling on the inference of terrestrial biospheric sources and sinks. *Tellus B: Chemical and Physical Meteorology*, *66*(1), 22486. https://doi.org/10.3402/tellusb.v66.22486

Liu, J., Bowman, K. W., Schimel, D. S., Parazoo, N. C., Jiang, Z., Lee, M., et al. (2017). Contrasting carbon cycle responses of the tropical continents to the 2015–2016 El Niño. *Science*, *358*(6360), eaam5690. https://doi.org/10.1126/science.aam5690

Liu, J., Baskaran, L., Bowman, K., Schimel, D., Bloom, A. A., Parazoo, N. C., et al. (2021). Carbon Monitoring System Flux Net Biosphere Exchange 2020 (CMS-Flux NBE 2020). *Earth System Science Data*, *13*(2), 299–330. https://doi.org/10.5194/essd-13-299-2021

Lundberg, S. M., & Lee, S.-I. (2017). A Unified Approach to Interpreting Model Predictions. In *Advances in Neural Information Processing Systems* (Vol. 30). Curran Associates, Inc. Retrieved from

https://proceedings.neurips.cc/paper/2017/hash/8a20a8621978632d76c43dfd28b67767-Abstract.html
Norton, A. J., Bloom, A. A., Parazoo, N. C., Levine, P. A., Ma, S., Braghiere, R. K., & Smallman, T. L. (2023). Improved process representation of leaf phenology significantly shifts climate sensitivity of ecosystem carbon balance. *Biogeosciences*, *20*(12), 2455–2484.
O'Neill, B. C., Tebaldi, C., Van Vuuren, D. P., Eyring, V., Friedlingstein, P., Hurtt, G., et al. (2016). The Scenario Model Intercomparison Project (ScenarioMIP) for CMIP6. *Geoscientific Model Development*, *9*(9), 3461–3482. https://doi.org/10.5194/gmd-9-3461-2016
Pierrat, Z., Magney, T., Parazoo, N. C., Grossmann, K., Bowling, D. R., Seibt, U., et al. (2022). Diurnal and Seasonal Dynamics of Solar-Induced Chlorophyll Fluorescence, Vegetation Indices, and Gross Primary Productivity in the Boreal Forest. *Journal of Geophysical Research: Biogeosciences*, *127*(2), e2021JG006588. https://doi.org/10.1029/2021JG006588
Quetin, G. R., Famiglietti, C. A., Dadap, N. C., Bloom, A. A., Bowman, K. W., Diffenbaugh, N. S., et al. (2023). Attributing past carbon fluxes to CO_2 and climate change: Respiration response to CO_2 fertilization shifts regional distribution of the carbon sink. *Global Biogeochemical Cycles*. https://doi.org/10.1029/2022GB007478
Raoult, N., Douglas, N., MacBean, N., Kolassa, J., Quaife, T., Roberts, A. G., et al. (2024, October 8). Parameter Estimation in Land Surface Models: Challenges and Opportunities with Data Assimilation and Machine Learning. https://doi.org/10.22541/essoar.172838640.01153603/v1
Robinson, N., Drever, R., Gibbs, D., Lister, K., Esquivel-Muelbert, A., Heinrich, V., et al. (2024). Protect young secondary forests for optimum carbon removal.
Schneider, T., Lan, S., Stuart, A., & Teixeira, J. (2017). Earth System Modeling 2.0: A Blueprint for Models That Learn From Observations and Targeted High-Resolution Simulations. *Geophysical Research Letters*, *44*(24), 12,396-12,417. https://doi.org/10.1002/2017GL076101
Shen, C., Appling, A. P., Gentine, P., Bandai, T., Gupta, H., Tartakovsky, A., et al. (2023). Differentiable modelling to unify machine learning and physical models for geosciences. *Nature Reviews Earth & Environment*, *4*(8), 552–567. https://doi.org/10.1038/s43017-023-00450-9
Skulovich, O., Famiglietti, C., Konings, A., & Gentine, P. (2024). *Constraining respiration flux and carbon pools in a simple ecosystem carbon model* (preprint). Preprints. https://doi.org/10.22541/au.170708964.48441645/v1
Skulovich, O., Gentine, P., Wigneron, J. P., & Xiaojun, L. (2024). GLAB-VOD: Global L-band AI-Based Vegetation Optical Depth Dataset Based on Machine Learning and Remote Sensing (Version 1.0) [Data set]. Zenodo. https://doi.org/10.5281/zenodo.10306094
Space-for-Time Substitution as an Alternative to Long-Term Studies. (1989). In S. T. A. Pickett, *Long-Term Studies in Ecology* (pp. 110–135). New York, NY: Springer New York. https://doi.org/10.1007/978-1-4615-7358-6_5
Tao, F., Huang, Y., Hungate, B. A., Manzoni, S., Frey, S. D., Schmidt, M. W. I., et al. (2023). Microbial carbon use efficiency promotes global soil carbon storage. *Nature*. https://doi.org/10.1038/s41586-023-06042-3
Terrer, C., Jackson, R. B., Prentice, I. C., Keenan, T. F., Kaiser, C., Vicca, S., et al. (2019). Nitrogen and phosphorus constrain the CO2 fertilization of global plant biomass. *Nature Climate Change*, *9*(9), 684–689. https://doi.org/10.1038/s41558-019-0545-2
Wigneron, J.-P., Ciais, P., Li, X., Brandt, M., Canadell, J. G., Tian, F., et al. (2024). Global carbon balance of the forest: satellite-based L-VOD results over the last decade. *Frontiers in Remote Sensing*, *5*, 1338618. https://doi.org/10.3389/frsen.2024.1338618
Xu, X., & Trugman, A. T. (2021). Trait-Based Modeling of Terrestrial Ecosystems: Advances and Challenges Under Global Change. *Current Climate Change Reports*, *7*(1), 1–13. https://doi.org/10.1007/s40641-020-00168-6
Yang, H., Ciais, P., Frappart, F., Li, X., Brandt, M., Fensholt, R., et al. (2023). Global increase in biomass carbon stock dominated by growth of northern young forests over past decade. *Nature Geoscience*, *16*(10), 886–892. https://doi.org/10.1038/s41561-023-01274-4

Yang, P., Liu, X., Liu, Z., Van Der Tol, C., & Liu, L. (2023). The roles of radiative, structural and physiological information of sun-induced chlorophyll fluorescence in predicting gross primary production of a corn crop at various temporal scales. *Agricultural and Forest Meteorology*, *342*, 109720. https://doi.org/10.1016/j.agrformet.2023.109720

Zhang, Y., Guanter, L., Berry, J. A., van der Tol, C., Yang, X., Tang, J., & Zhang, F. (2016). Model-based analysis of the relationship between sun-induced chlorophyll fluorescence and gross primary production for remote sensing applications. *Remote Sensing of Environment*, *187*, 145–155. https://doi.org/10.1016/j.rse.2016.10.016

Responses to the Reviewers

Reviewers' original comments are shown in black, our responses in blue, and the corresponding revisions to the manuscript in brown (with line number in the tracked change version of the manuscript).

Reviewer #1 (Remarks to the Author):

Dear authos,

I appreciate the substantially revised manuscript. By the current version, it has been reasonable to accept for publication.For the following reason:

The authors addressed the reviewers' comments by tightening the framing, expanding analyses, and clarifying methods. They reframed the narrative to emphasize ecological mechanisms and the added value of their approach beyond technical novelty, and they standardized evaluation by adopting a clear PFT-only baseline for comparison.

Methodologically, they distinguished static spatial predictors from time-varying forces, documented the full data flow with a schematic and a worked pixel example, and broadened observational evaluation by incorporating additional eddy-covariance networks and multiple alternative remote-sensing products in a structured sensitivity suite. Concerns about spatial scale mismatch were tackled with representativeness tests, while interpretability was strengthened through biome-level SHAP summaries and clearer variance partitioning of predictor contributions. They acknowledged and analyzed weaker skills in moisture-related processes, outlined concrete upgrades for stomatal conductance and multi-layer hydrology, and compared performance against established DGVM/TRENDY benchmarks across relevant timescales. Editorially, definitions (e.g., NBE) and terminology (e.g., agricultural intensity) were clarified, figures and metrics were cleaned up for consistency, and code, documentation, and tutorials were released to support transparency and reproducibility. Remaining scope limits—particularly true multi-decadal predictability—were explicitly discussed with reasoned justification and a roadmap for future work.

We are grateful to the reviewer for their positive evaluation and for the constructive comments that strengthened the manuscript.

Reviewer #1 (Remarks on code availability):

The code (based on core code DALEC993.py) demonstrates several notable strengths that indicate high-quality scientific software development.

The implementation provides a comprehensive carbon cycle model with seven carbon and water pools, multiple flux pathways, and sophisticated process representations, including phenology, fire disturbance, and water stress. Particularly impressive is the flexible framework offering six different water stress formulations, which allows for scientific experimentation and comparison of different modeling approaches. The code carefully implements minimum and maximum bounds on all pools with detailed mass balance corrections, ensuring physical consistency throughout the simulation.

Despite these strengths, to me only a technical issues that should be addressed to improve code robustness. The code uses several magic numbers such as WATER_POOL_SCALE = 1500 and GPP_SCALE = 8 without documentation of their scientific justification or rationale.

We thank the reviewer for the positive evaluation on our code structure. These constants serve as heuristic normalization factors for the embedded neural network within the DALEC model. We have added annotations in the code to explain their usage.

Reviewer #2 (Remarks to the Author):
I appreciate the substantial revisions and the authors' efforts to address my earlier concerns—for example, establishing an appropriate baseline configuration and analyzing parameter importance. That said, the extensive rewrite has made the manuscript more challenging to follow than the previous version. The study's objectives are not stated with sufficient clarity, partly due to ambiguous terminology and missing definitions of key concepts. I also noted apparent inconsistencies—for instance, in the discussion of acclimation effects and the suitability of the approach for extrapolation/long-term prediction. Clarifying these issues is essential for readers to understand the scientific contribution and how this work advances the broader goal of improving predictability of ecosystem dynamics under climate change.

Thank you for this careful and constructive assessment. We appreciate your recognition of the revisions and your detailed feedback on clarity, terminology, and consistency.

We agree that the objectives, definitions of key concepts, and the logical framing of the work must be transparent for readers to understand the contribution. In response, we have revised the manuscript to state the study objectives more explicitly in the Introduction, clarified and standardized terminology

throughout, added definitions where concepts were previously implicit, and resolved the inconsistencies you noted regarding acclimation and extrapolation. We have also revised the narrative to improve readability and to make the connection to predictability under climate change more rigorous.

We hope these targeted revisions address your concerns and make the manuscript clearer and more accessible to the reader.

1. Ambiguity/Inconsistency of terminology. There are many important concepts that were not clearly defined or misused. Here are some examples:

• Both "Plant-Type" and "PFT" were used throughout the manuscript and even in the same sentence. What are their differences?
Thanks for raising this point. We now use only the term "plant-functional type (PFT)" in the revised manuscript.

• What is the definition of "environment gradient" – one of the core concepts in this manuscript? What is its difference from environmental forcing? Gradient can mean many different things in different dimensions under different timescales.
Thanks for raising the clarifying point. In our context, "(spatial) environmental gradient" refers to the spatial variations in environmental predictors (e.g., climatology, elevation, soil properties, etc.) that are assumed to shape the distribution of ecological parameters in the DifferLand model at timescales longer than the simulation period. Thus, spatial gradients in environmental predictors, and the learned environment-parameter relationships, are assumed to be temporally stationary for the modeling purposes. We clarified this in the opening sentence of our introduction:

Line 51-53: Understanding how spatial gradients in abiotic and biotic drivers, such as climate, soils, and forest age, shape the distribution of plant functional traits is a long-standing question in ecological and climate research.

In contrast, "environmental forcing" refers to fast, time-varying drivers that act on ecosystems at a given location, including meteorological variability and disturbances such as temperature, precipitation, radiation, vapor pressure deficit, and fire. These forcings vary on sub-seasonal to interannual timescales and are prescribed as dynamic inputs to the process model at each pixel.

Line 135-138: By coupling spatial environmental gradients, which structure trait distributions on decadal timescales, with grid-cell-level process representation, DifferLand reveals functional relationships linking plant traits to climate, soils, and vegetation history while retaining sensitivity to transient local meteorological forcing at monthly timescales.

The distinction here is both functional and pragmatic. Environmental gradients describe slow, space-dominant controls on parameter distributions, whereas forcings describe fast, time-dominant drivers of ecosystem fluxes and states. This separation reflects a modeling design choice based on timescale separation. Latent ecological parameters encode the imprint of long-term environmental gradients, while the process model propagates transient forcings to short-term responses. We do not imply that these processes are truly stationary or separable in nature, but rather that this approximation is appropriate at the timescales and spatial resolution considered here.

We have revised the Introduction to explicitly define these terms and to clarify the rationale behind this separation of spatial gradients and temporal forcings in the model design.

• "Ecological parameters/traits": In Fig. 1 caption, "photosynthesis" is one of the 40 ecological parameters, however, photosynthesis is a process, a variable, a flux, not a parameter. Similarly, In the response letter: LAI and SIF are latent ecological traits, which are incorrect. Moreover, photosynthetic efficiency, and carbon use efficiency are all variables not parameters, so they are supposed to vary in time and space

Thank you for pointing out this important terminological and conceptual issue.

You are correct that photosynthesis, LAI, SIF, photosynthetic efficiency, and carbon use efficiency are processes or state and flux variables rather than parameters in the ecological sense.

In Figure 1, the label "photosynthesis & phenology" was intended as a process category rather than as a parameter itself. It refers to the subset of model parameters that control the representation of photosynthesis in the mechanistic model, such as nitrogen-limited photosynthetic capacity per unit leaf area or related phenological coefficients (see parameter annotations next to the spheres). These parameters are used by the model to simulate the photosynthesis process and its resulting fluxes. We have clarified this explicitly in the figure caption and text.

We also correct the earlier wording in the response letter. LAI and SIF are not latent ecological traits or parameters but observed state and flux variables that are assimilated to constrain the model. The manuscript now reflects this distinction consistently.

With respect to quantities such as photosynthetic efficiency and carbon use efficiency, we agree that they are variables that can vary in both space and time in real ecosystems. The distinction we make is therefore a modeling one rather than an ecological one. The model requires an explicit choice of which quantities are treated as prognostic state variables that vary in time, and which are treated as latent parameters whose values are inferred but assumed fixed over the simulation window. In our framework, parameters represent slowly varying or long-term effective properties that encode the imprint of environmental filtering and adaptation, while faster dynamics are represented explicitly through state variables and fluxes driven by time-varying forcings.

We have revised the caption to make this modeling distinction explicit and to avoid conflating processes and parameters in terminology.

Fig. 1 | Schematics of the DifferLand framework. a) DifferLand integrates spatial information from four groups of environmental predictors: plant functional types (PFTs), climatology and elevation (CLIM), forest age and maximum canopy height (AGE), and soil texture properties (SOIL). b) During a forward pass (black arrows), a spatialization neural network learns environment–parameter relationships, mapping these predictors to c) global fields of ecological *model parameters*, which act as model representations of plant functional traits and regulate vegetation processes such as photosynthesis, carbon allocation, carbon turnover in different carbon pools, and sensitivities to drought and fire. d) A terrestrial biosphere model uses these parameters together with meteorological forcing to simulate ecosystem *state variables* and *fluxes*, including leaf area index, biomass pools, photosynthesis, respiration, and carbon and water exchange. e) DifferLand then evaluates the simulated states and fluxes against multiple streams of satellite and in situ observations. Its differentiable structure (orange arrows) provides gradients of the model–data mismatch with respect to the ecological parameters. These gradients update the spatialization network through iterative optimization of the inferred environment–parameter relationships.

• “Ecological latent space”: How to validate the latent space? How do that compare in the context of theoretical/field ecology research?

Thank you for raising this insightful point. In our context, learning in a latent space refers to identifying low-dimensional structure that captures coordinated variation among high-dimensional ecological processes and parameters. This idea is well established across disciplines as a form of dimensionality reduction that reveals intrinsic organizing axes in complex systems (Bengio et al., 2013; Tenenbaum et al., 2000). In ecology, although the term “latent space” is more commonly used in machine learning and physical sciences (Fan et al., 2025; Floryan & Graham, 2022) the underlying concept has deep roots in theoretical and field ecology.

Canonical examples include the leaf economics spectrum and related trait spectra, which describe coordinated variation among multiple leaf traits along unobserved functional axes (Díaz et al., 2016; Wright et al., 2017), as well as ecosystem-level organizing axes such as productivity, water-use strategies, and carbon-use efficiency (Joswig et al., 2021; Migliavacca et al., 2021). These studies typically use principal component analysis or related techniques to extract latent axes that summarize ecological structure. In this sense, the latent space in DifferLand plays an analogous role, representing emergent axes of coordinated trait and process variation inferred from data.

Validation of such a latent space cannot rely on direct point-wise comparison, because the axes are not directly observable quantities. Instead, we evaluate its ecological relevance and realism in three complementary ways. First, we assess whether the dominant axes inferred by DifferLand align qualitatively and quantitatively with known ecological spectra and gradients reported in the literature, such as leaf economic strategies and short-tall plant stature axes (Fig. 3a). Second, we examine whether projections of independent ecological variables and fluxes onto these axes produce spatial patterns consistent with known environmental drivers (Fig. 3b). Third, we evaluate whether using these latent axes for parameter spatialization improves model performance and spatial generalization relative to baseline formulations based on plant functional types or globally uniform relationships (Fig. 2).

Together, these tests do not constitute a direct “validation” of the latent space in a strict sense, but they provide evidence that the inferred embedding captures meaningful ecological structure consistent with both theory and observations, while offering practical advantages for model representation and parameter spatialization.

Clear concept and consistent terminology is fundamental to ensure readers understands the manuscript.
Agreed. We have revised the manuscript to ensure clear definitions and consistent terminology use.

2. Long-term prediction. I appreciate some of the efforts that the authors made to address the suitability of

Differland for improving long-term prediction, i.e., over decadal to century timescales. Considering that "Improving the long-term predictability of terrestrial biosphere model is a key motivation for developing our hybrid differentiable framework" (quote in the response letter) and "embedding such relationships into terrestrial models establishes a pathway toward adaptive models that better predict ecosystem resilience under climate change", it is important to thoroughly address this issue. However, I found that the argument and evidence that authors provided are insufficient. The two pieces of "supporting evidences" provided are: 1) lower RMSE in simulated NBE relative to TRENDY simulations (Fig. S8), and 2) evaluation of DifferLand with CAMS NBE for a different period from the training period. These two tests do not necessarily mean "generalization into the future" in the time dimension, although it can imply that it can generalize in space during the contemporary period. It is also inconsistent that, on the one hand, the authors said "full decadal-to-centennial testing is beyond the scope of this study", but on the other hand, the authors made a dedicated section "Implications under changing climate" using extrapolation approach based on the assumption of space for time substitution. The key problem is that all observational products, used for training DifferLand, represent the environmental regime and variations only for a limited period, while future prediction is for a totally new/novel regime that was never seen by contemporary observational product. Yes, the training period of 2010-2022 differs from the validation period 2003-2009, but the difference between these two period is NOT at the same level of magnitude for their difference with the period of 2080-2099 (as is shown in Fig. S2, the second test). Even though DifferLand is doing a superb job in optimizing parameter, and especially the combination of parameters in the latent space, it does not necessarily mean these parameters/traits and their relationship with ambient environmental conditions will hold into the future. But in line 774-775, the authors "assumed that the relationships between parameters and the environment remained stable throughout the study period", and apply such relationship for extrapolation. It is risky to assume the generalizability of DifferLand to the future, and the extrapolation results can be misleading.

We thank the reviewer for this thoughtful feedback on our treatment of long-term prediction and extrapolation. We agree that the original framing overstated what can be demonstrated with contemporary observational constraints, and we appreciate the opportunity to clarify the scope, limitations, and appropriate interpretation of our results.

We agree with the reviewer that the two tests we presented, namely improved agreement with TRENDY during the contemporary period and evaluation on an earlier historical period, primarily demonstrate improved spatial and near-term temporal generalization within the present climate regime. They do not constitute evidence of reliable generalization into fundamentally novel future climate conditions. We also agree that the difference between the training and validation periods is modest compared to the magnitude

of projected late-century climate change and therefore cannot be used to validate true long-term extrapolation.

Our original motivation in highlighting long-term implications was based on the view that parameter uncertainty is a dominant contributor to uncertainty in terrestrial biosphere projections, and that constraining parameters using multiple contemporary data streams is a necessary step toward improving model credibility. However, we agree with the reviewer that this alone does not justify strong claims about predictive skill under future, potentially non-stationary ecological regimes.

We also acknowledge the reviewer's point regarding internal consistency. It is indeed inconsistent to state that decadal-to-centennial testing is beyond the scope of the study while simultaneously presenting a dedicated section on implications under changing climate using space-for-time substitution. We recognize that space-for-time substitution is a heuristic, not a validation strategy for future prediction, and that it rests on assumptions about stationarity of trait–environment relationships that may not hold under strong climate forcing.

While we hypothesized that learning relationships in a latent parameter space might be more robust than extrapolating observable states or fluxes directly, we agree that this remains a hypothesis rather than a demonstrated property, and that evolutionary, acclimatory, and demographic processes can alter trait distributions and trait–environment relationships over time. We therefore agree that assuming their stability for future projection is risky and potentially misleading.

In response to these concerns, we have made the following substantive revisions:

1. We have toned down claims regarding long-term predictability and removed language suggesting that DifferLand is validated for decadal-to-centennial forecasting.
2. We have removed the dedicated section on future climate extrapolation and space-for-time substitution and instead incorporated a more cautious discussion of extrapolation uncertainties into the Discussion section.
3. We now frame our contribution explicitly as improving spatial generalization and the retrieval of present-day environment–trait relationships, rather than as providing reliable future projections.
4. We have incorporated a clearer discussion of the key limitations, including non-stationarity of traits, lack of long-term observational benchmarks, and the need for independent constraints on ec o-evolutionary and acclimation processes.

5. We outline concrete directions for future work, including the use of emerging long-term observational records, integration of demographic and eco-evolutionary constraints, and explicit treatment of non-stationarity, as necessary steps toward credible long-term prediction.

Line 631-643: We note that the conclusions drawn from this research are dependent on the timeframe considered for the observations. In this 23-year study, spatial predictors were treated as temporally invariant, under the assumption that their temporal variations are negligible compared to their mean spatial gradients. Furthermore, we assumed that the relationships between parameters and the environment remained stable throughout the study period, implying that adaptation to environmental changes occur on much longer timescales. However, these assumptions may not always hold for long-term predictions, in which case environmental conditions, land use patterns, population distributions (Kelly & Goulden, 2008), community compositions (H. Liu et al., 2018), and plant functional responses (Franks et al., 2014) could all undergo significant shifts. At multidecadal to centennial timescale, slow processes such CO_2 fertilization (Quetin et al., 2023), nutrient limitation (Terrer et al., 2019), evolving forest demography (Robinson et al., 2024; Yang et al., 2023), and belowground carbon dynamics (Huang et al., 2024; Tao et al., 2023) can dominate the trajectories of land-atmosphere carbon exchange, yet most of these slow processes can be constrained only with long-term observations or by making equilibrium assumptions. Future work should further investigate shifts in ecological parameters to changing environment using long-term observational records.

We believe these revisions better align the manuscript with what is empirically supported by the current analysis, while still situating the work within the broader goal of improving predictive ecological modeling. We thank the reviewer for prompting this clarification and for helping us sharpen both the scope and the rigor of the paper.

In addition, one of the fundamental assumptions here: Space-for-time substitution, can cause misleading results: Evans et al., 2025: https://www.nature.com/articles/s41558-025-02392-0 . So the authors have to be really cautious in extrapolating their findings into the long-term future predictability – the ultimate goal.

We thank the reviewer for pointing us to this important and timely reference. We fully agree with Evans et al. (2025) that space-for-time substitution can be misleading and should be treated with caution, particularly when used to infer long-term temporal change from spatial gradients.

As discussed by Evans et al., space-for-time substitution can fail for at least two fundamental reasons. First, spatial correlations between ecological variables and climate do not necessarily reflect causal relationships, as they may arise from confounding factors such as species composition, edaphic conditions, disturbance history, or biogeographic constraints. Second, temporal ecological responses to climate change may be strongly transient and lagged, rather than in equilibrium with contemporary climate, such that spatial gradients cannot be interpreted as temporal sensitivities.

Our original intent in presenting extrapolations based on global versus PFT-specific environment–trait relationships was not to claim predictive validity for future ecosystem change, but to illustrate how different assumptions embedded in space-for-time approaches can lead to qualitatively divergent outcomes. For example, global apparent relationships can conflate differences among plant functional types with climate sensitivities, thereby introducing confounding consistent with the first failure mode described by Evans et al. Conversely, PFT-specific relationships assume relatively stable functional identities and sensitivities, which may themselves become invalid under rapid climate change due to migration lags, demographic inertia, or evolutionary change, consistent with the second failure mode related to transient dynamics.

We agree, however, that even with careful framing, this analysis risks being interpreted as a substantive statement about future ecosystem trajectories, which was not our intention and is not the main finding of the manuscript. We therefore agree with the reviewer that its inclusion may be misleading in the context of the present study.

In response, we have removed the "Implications under changing climate" section from the manuscript and now reserve this type of analysis for future work that explicitly addresses when, where, and under what conditions space-for-time assumptions may hold or break down, using appropriate long-term benchmarks and mechanistic constraints. In the revised manuscript, we focus the scope of our contribution on the discovery and characterization of environment–trait relationships in the contemporary climate, and we discuss the limitations of extrapolation and non-stationarity more explicitly in the Discussion (see above).

We thank the reviewer for highlighting this issue and helping us improve the conceptual clarity and rigor of the manuscript.

3.Data uncertainty. I appreciate the authors' massive efforts to evaluate the uncertainty of satellite product on the final results. But I'm a little confused about Text S4 and Fig. S30-31. Do these figures show the

absolute magnitude of NBE or deltaNBE? The figures say the former while the text says the latter. It is also confusing that what does R2 means for deltaNBE, DeltaLAI, etc, in Fig. S29. With lack of clarity of these figures, it's hard to evaluate the robustness of the results to the data choice.

Thank you for this clarification request. We agree that the notation and presentation were potentially ambiguous and have revised the text and figure captions accordingly.

In the Supplement, the prefix "Δ" does **not** denote a difference field. Instead, it labels a *sensitivity experiment* in which the default assimilated dataset is replaced by an alternative product. Specifically, ΔNBE denotes the experiment in which CMS-Flux NBE is replaced by CAMS-NBE, and ΔLAI denotes the experiment in which MODIS LAI is replaced by the Copernicus LAI product. The precise definition of each sensitivity experiment and the corresponding dataset substitutions are now explicitly listed in Table S1 and referenced consistently in the captions.

Figures S31–32 (originally S30-31) therefore show the **actual regional mean values of NBE** obtained under the sensitivity experiment, not the difference between experiments. We have clarified this in the captions and the accompanying text to avoid confusion with a true anomaly or perturbation notation.

The R^2 values shown in Fig. S30 are the aggregated spatiotemporal coefficients of determination computed on held-out test pixels for each experiment configuration. They quantify model predictive performance under each data-assimilation setup and are not defined on differences between experiments. We have now stated this explicitly in the figure caption and text.

The purpose of Figs. S30–33 is to demonstrate that the overall model skill is robust to the choice of assimilated dataset. In particular, the PFT+CLIM+SOIL+AGE configuration consistently outperforms the PFT-only baseline across all sensitivity experiments, indicating that the main conclusions are not driven by a specific data product choice.

In Text S4: We found that this experiment assimilating the alternative CAMS NBE inversion (corresponding to the "ΔNBE" experiment in Table S1) shows consistent spatial and temporal patterns as the default runs (Fig. S31), although the overall net carbon sink is stronger at -2.79±0.94 PgC/year compared with the -2.09±0.62 PgC/year in the default runs, consistent with the more sinky pattern in CAMS (-2.77 PgC/year) versus CMS (-2.08±0.09 PgC/year).

Moreover, when looking at Fig. S31d, there sign of NBE in south American Tropics (the Amazon region) is different between CAMS inversion and CMS inversion: the former shows an overall weak carbon

source while the latter shows strong carbon sink. Because DifferLand was trained on CMS product (over 2010-2023), its predicted NBE during 2003 to 2009 is also a carbon sink, which is in great contrast to the strong source from CAMS inversion.

Thank you for this careful observation. As shown in Fig. S31d (now Fig. S32d), CAMS and CMS inversions indeed diverge substantially over the Amazon, with CAMS indicating a weak net carbon source while CMS indicates a pronounced carbon sink. This discrepancy highlights the considerable uncertainty in inversion-based NBE products over the Amazon and how such uncertainties propagate into data-assimilated model simulations.

We note that uncertainties in the CAMS inversion are particularly large in this region. Prior to 2014, CAMS relies exclusively on surface CO_2 concentration measurements for flux inversion, yet long-term in situ monitoring sites in the Amazon are sparse (Liu et al., 2021). As a result, tropical and Southern Hemisphere flux estimates from CAMS during the early 2000s are weakly constrained and sensitive to prior assumptions and transport errors. These limitations motivate our choice of CMS-Flux as the default NBE product for assimilation, given its stronger constraints during the satellite era, particularly in the Southern Hemisphere. The comparison shown in Fig. S31d explicitly demonstrates that uncertainties in assimilated datasets can directly influence inferred regional carbon balance in DifferLand.

We acknowledge that independent regional inversion studies using airborne vertical CO_2 profiles have provided evidence for a weakening Amazon carbon sink, with parts of the southern Amazon acting as a net carbon source during the 2010–2018 period due to deforestation and increasing heat and drought stress (Gatti et al., 2021). Incorporating such airborne and ground-based observations through regionally targeted data assimilation frameworks and reconciling differences with other observational constraints are promising directions for future work to better constrain carbon cycle dynamics in this ecologically critical region.

4. Agriculture intensity. Even though the authors replaced “agricultural intensity” with “cropland fraction”, it does not address my previous concern on why cropland is a major driver of spatially varying ecological parameters? This is not intuitive. Neither cropland distribution nor agriculture activity can impact the ecological traits for natural vegetation. They do not have any causal connections. Cropland fraction appears as the 3rd axes is primarily an artifact that satellite products of carbon fluxes, used for DifferLand training, is greatly impacted by agriculture activities. But “agriculture activity” or “cropland fraction” itself is not the cause for the ecological parameters or the coordination across them.

Thank you for this clarification. We fully agree that cropland fraction does not causally control the ecological traits of natural vegetation, and we do not interpret it as such. In the revised manuscript we have clarified that only the first two axes of the PCA correspond to coordinated variation in latent ecological traits associated with natural vegetation, namely the leaf economics spectrum and the tall–short plant stature gradient. The third axis is orthogonal to these trait-coordination axes and reflects a distinct signal associated with human land use, primarily cropland coverage.

We therefore do not interpret the third axis as a driver of ecological traits, but as a diagnostic dimension capturing systematic differences in carbon and water flux signatures between managed and natural ecosystems that are present in the assimilated observations. This signal arises because croplands exhibit characteristic phenology, particular peak growing season photosynthesis, and harvest-induced carbon removals that are distinct from those of natural vegetation, and these differences are embedded in the atmospheric and satellite constraints used for training DifferLand. In this sense, the third axis reflects the imprint of agricultural land use on the terrestrial carbon cycle, not a causal control on plant trait coordination.

This interpretation is supported by independent evidence. For example, Sun et al. (2021) showed that only terrestrial biosphere models that represent strong, seasonally peaked carbon uptake in U.S. Midwest croplands are consistent with observed atmospheric CO_2 variability, whereas models with weak cropland seasonality produce CO_2 signals inconsistent with measurements. Thus, although DifferLand does not explicitly simulate agricultural management, the distinct flux and phenological signatures of croplands can be inferred through data assimilation and appear as a separate dimension in parameter space.

We have revised the text and figure caption accordingly to avoid implying any causal role of cropland fraction in shaping ecological traits of natural vegetation, and to emphasize that the third axis represents an emergent land-use imprint in the observation-constrained system rather than a mechanistic ecological gradient.

Line 336-347: The analysis also revealed a third component (PC3), orthogonal to the two natural trait coordination axes, which explains 10.5% of the variance (Fig. 3b) and is associated with regions of high photosynthetic efficiency and intensive cropland use, such as the U.S. Midwest, Southern Europe, and Eastern China. This pattern is consistent with the widespread cultivation of high-yield crop varieties and the prevalence of photosynthetically efficient C4 crops, in these regions.

Line 668-672: The two dominant axes of latent parameter variation correspond to natural leaf economics and plant stature, while a third diagnostic axis captures residual effects of cropland coverage. Together, these axes highlight that environment–parameter relationships governing carbon and water fluxes emerge from the interaction between macroecological gradients, plant-type–specific effects, and human land management.

BTW, this phrase pf "agricultural intensity" still exists in the revised manuscript – line 820.
Thanks. We have adjusted this phrase in the revised text.

5. Acclimation: Their authors' notion and discussion on "Acclimation" is self-contradictory. Specifically, in Line 771-786 "We note that the conclusions drawn from this research are dependent on the timeframe considered for the observations. In this 23-year study, spatial predictors were treated as temporally invariant, under the assumption that their temporal variations are negligible compared to their mean spatial gradients. Furthermore, we assumed that the relationships between parameters and the environment remained stable throughout the study period, implying that plant acclimation and adaptation to environmental changes occur on much longer timescales".

When addressing my questions on Vcmax, the authors responded that "The temperature dependence we identify (now Fig. 4a) does not represent the short-term enzymatic limitation of Rubisco, but rather the long-term acclimation of photosynthetic efficiency to climatological mean annual temperature. By capturing this acclimation, DifferLand highlights an additional source of parameter variation along environmental gradients that is typically missing in TBMs assuming fixed Vcmax25."
So it's very confusing whether DifferLand included acclimation or not, from authors' texts.

Thank you for pointing out this inconsistency. We agree that our previous wording created confusion, and we have revised the text to clarify both the terminology and the modeling assumptions.

The source of the confusion is that we previously used the term "acclimation" in two different senses. In a physiological sense, acclimation refers to within-generation, phenotypic adjustment of plant function to environmental conditions. In a modeling sense, acclimation refers to explicitly representing non-stationary, time-evolving changes in model parameters in response to changing environmental forcing.

DifferLand does not simulate acclimation in the modeling sense. All ecological parameters, including nitrogen-limited photosynthetic efficiency (which is analogous but slightly different to V_{cmax25} in Farquhar-like models as it represents a canopy-aggregated value) are assumed temporally invariant within a simulation, and the inferred relationships between parameters and environmental variables are assumed stationary over the study period. Thus, DifferLand does not represent time-dependent adjustment of parameters to climate change.

The temperature dependence of canopy efficiency identified in Fig. 4a should therefore be interpreted as a spatial pattern across climatological gradients, reflecting systematic differences in ecosystem function associated with long-term mean climate. This pattern may integrate effects of evolutionary adaptation and long-term phenotypic adjustment embedded in the spatial data, but it does not represent dynamic acclimation over time within the model.

To remove this ambiguity, we have revised the manuscript to use “acclimation” only in the modeling sense, following the reviewer’s framing, and we now refer to the physiological concept as “within-generation response.” We have also clarified that the parameter–environment relationships inferred by DifferLand are spatial and stationary, and that the framework does not represent temporal acclimation processes.

6. Abstract. I recommend a thorough revision of the abstract to enhance clarity. Suggestions below.
Thanks a lot for these detailed and insightful suggestions. We have thoroughly revised the abstract to enhance clarity. The revised abstract now reads as:

Line 9-22: The spatial distribution of plant functional traits observed today are living imprints of current environmental gradients and past selection, offering insight into how plants have adapted to their environments. What remains insufficiently understood is how traits combine and coordinate across environments, and whether such coordination reflects organizing principles in ecology that can improve modeling of ecosystem functional diversity and decadal-scale carbon exchange. Here we present DifferLand, a differentiable hybrid model that learns high-dimensional, coordinated environment–trait relationships directly from multi-modal satellite and in situ observations. DifferLand reveals a small number of latent axes that represent how suites of plant traits jointly shape vegetation dynamics and carbon–water fluxes, enabling the model to capture both long-term adaptation patterns and short-term responses to meteorological variability, and to outperform models that rely solely on plant functional

types in spatial generalization. The spatialization network learns nonlinear interactions between plant functional attributes and environmental gradients, organizing latent ecological parameters that represent functional traits at the global scale. This environment trait–trait structure reveals large-scale patterns of ecosystem functional diversity and improves the spatial generalization of terrestrial biosphere models.

- Line 9: Do ecosystems primarily reflect evolutionary history or current environment? – which aspects of ecosystems – morphology, composition, functional traits, fluxes, storage? As the first sentence setting the tone of the manuscript, it is important to have precise and clear description.

Thanks a lot for the suggestion. We have clarified that we are mainly focusing on functional traits. The opening sentence now reads as:

The spatial distribution of plant functional traits observed today are living imprints of current environmental gradients and past selection, offering insight into how plants have adapted to their environments.

- Line 10: “unresolved questions” – The answer to the question is known, because its from both evolutionary history and current environment. What is unknown is how do traits combine and coordinate, and whether represent such coordination can make a difference in improving predictability of TBMs for future environments.

Thanks a lot for this suggestion which helps us clarify the logical connections. We have rephrased the phrasing.

Rephrased sentence: What remains insufficiently understood is how traits combine and coordinate across environments, and whether such coordination reflects organizing principles in ecology that can improve modeling of ecosystem functional diversity and decadal-scale carbon exchange.

Line 10-13: there is no logical connection between the two clauses.

We have removed these clauses to avoid ambiguities.

Line 13: “govern” means “determine”; traits itself do not determine the carbon fluxes or vegetation dynamics, it only “impact”.

We have clarified this logical connection in the revised text.

Line 14: “yet such multivariate relationships cannot be directly observed” – this is already being observed by many studies, which also cited by the authors.

We have revised the sentence to be more precise.

Line 17: “DifferLand” is an approach, it cannot “explain” the variance; only certain processes/mechanisms can “explain” variances of observed variables.

Revised sentence: DifferLand reveals a small number of latent axes that represent how suites of plant traits jointly shape vegetation dynamics and carbon–water fluxes, enabling the model to capture both long-term adaptation patterns and short-term responses to meteorological variability, and to outperform models that rely solely on plant functional types in spatial generalization.

Line 19-20: The “nonlinear interactions” is already known, not uniquely revealed by “interpretable machine learning”; but ML can capture such nonlinear interactions.

Revise sentence: The spatialization network learns nonlinear interactions between plant functional attributes and environmental gradients, organizing latent ecological parameters that represent functional traits at the global scale. This environment trait–trait structure reveals large-scale patterns of ecosystem functional diversity and improves the spatial generalization of terrestrial biosphere models.

Line 21: “such relationships” – be explicit.

We have removed the vagueness in the expression.

7. Fig. 4. Fig. 4 and Fig. S1 seem to contradict to each other. The similar shapes of traits-temperature relationship (e.g., slopes) across PFTs in Fig. 4 indicate that the S3 scenario is sufficient – “Consistent scaling across PFTs – mean trait values differ among PFTs, but trait-environment relationship are similar across PFTs”.

Thank you for raising this point. Figure S1 and Figure 4 serve different purposes and therefore are not intended to be directly comparable or mutually exclusive.

Figure S1 is a purely conceptual illustration constructed from synthetic examples to delineate a set of idealized scenarios of how trait–environment relationships could differ across plant functional types. It is intended as a reference framework to guide interpretation, not as a result derived from data.

Figure 4, by contrast, shows empirical relationships inferred from the data using conditional SHAP analysis applied to DifferLand outputs. It is true the results indicate that, for several traits, the trait–

temperature relationships have broadly similar slopes across PFTs but different mean levels, corresponding most closely to the “consistent scaling across PFTs” scenario illustrated conceptually in Fig. S1 (scenario S3). However, we note there are also other examples that align more with scenario S1 and S4.

We have clarified this correspondence in the revised text to avoid the impression of a contradiction, and to state explicitly that Fig. S1 provides a conceptual taxonomy of possible patterns, while Fig. 4 shows which of those patterns is most consistent with the inferred relationships in the data.

8. SIF and GPP relationship. Although the authors justify the linear relationship between SIF and GPP at the monthly scale and beyond, their slope/intercept values in Table S2 are wild. Theoretically, there should be no intercept between them. SIF is directly related to the actual electron transport rate, which is balanced with carboxylation in the dark reaction. Their mechanistic equations are all multipliable, no additive.

Thank you for this careful and technically grounded comment. We agree with the reviewer that, from a mechanistic perspective, the relationship between SIF and GPP should pass through the origin, since SIF is proportional to the actual photosynthetic electron transport rate, which in turn is coupled multiplicatively to carboxylation in the dark reactions. There is therefore no physical basis for a nonzero intercept in the ideal, noise-free case.

In practice, however, remotely sensed SIF retrievals contain systematic and random uncertainties, including instrument noise, atmospheric correction errors, cloud and aerosol contamination, bidirectional reflectance effects, and residual background signals. These effects can introduce small offsets in empirical SIF–GPP regressions, even when the underlying physiological relationship is strictly proportional. For this reason, the empirical regressions we use do not enforce a zero intercept.

We also note that the originally reported intercept values in Table S2 were misleading because of an incorrect unit conversion, which has now been corrected in the revised manuscript. After correction, the intercepts are small and close to zero, consistent with the theoretical expectation and with the magnitude expected from retrieval noise rather than from a true physiological offset.

Finally, we clarify that the slope and intercept values in Table S2 are not derived from the mechanistic equations of our model, but from independent empirical relationships between satellite SIF and eddy-covariance GPP used for observational calibration (Fang et al., 2025). These regressions are intended as

statistical mappings to link observational products, not as statements of mechanistic causality. We have clarified this distinction and the associated limitations in the revised text.

Parameter	Description	Unit	Min	Max
α_{SIF}	Slope factor GPP-SIF relationship	(mW m^{-2} nm^{-1} sr^{-1}) / (gC m^{-2})	1/35	1/3
β_{SIF}	Intercept of GPP-SIF relationship	mW m^{-2} nm^{-1} sr^{-1}	-0.5	1/6

Other comments:

1. Validation and comparison with existing datasets/studies: As this work aims not only to demonstrate a technical framework (Figs. 1-2) but also to test ecological hypotheses and facilitate knowledge discovery (e.g., Figs. 3-4), assessing the accuracy of the inferred parameters is equally important.

We appreciate this comment from the reviewer. In the revised manuscript, we have incorporated additional comparisons that evaluated the retrieved parameters and parameter-relationship with observational benchmarks or published gridded datasets derived from upscaled site-level observations. Please see the detailed results below.

(a) While I appreciate the authors' efforts in comparing two inferred parameters (CUE and LCMA, Figs. S39 and S42) with independent datasets and site-level measurements, the validation appears limited and largely qualitative. More quantitative analyses would add credibility.

In addition to CUE and LCMA, we now present spatially explicit evaluations for two additional inferred parameters: the temperature sensitivity of heterotrophic respiration (Q10) and the root to total biomass allocation fraction. These assessments rely on independent model-data fusion products and upscaled global datasets whenever such references are available. The corresponding analyses and figures appear in Supplementary Information Text S6 and the associated figures.

Direct quantitative validation of inferred parameters remains inherently challenging because of fundamental differences between the physical meaning of model parameters and the quantities represented in available observations. For example, DifferLand estimates the fine root to total biomass allocation fraction, whereas the only globally available benchmark describes total root to total biomass, including both fine and coarse roots. Within DifferLand, coarse roots are represented as part of woody biomass, so the two metrics describe different ecological quantities and cannot be directly equated. Comparable conceptual mismatches affect several other parameters.

Further inconsistencies arise from differences in spatial and temporal scale. Many observational benchmarks derive from plot-level or species-level measurements, whereas DifferLand infers effective parameters at a 0.25° grid-cell scale. Likewise, short-term field measurements often reflect local and transient conditions, while the model parameters represent long-term climatological means. These mismatches limit the extent to which point measurements can serve as strict quantitative validation targets.

During the reevaluation of LCMA, we identified substantial discrepancies in tropical regions and a large spread among existing benchmark products (Bloom et al., 2016; Lusk et al., 2025; Moreno-Martínez et al., 2018). Importantly, these differences persist even among upscaled datasets built from overlapping trait databases. The results indicate considerable observational uncertainty in LCMA for tropical evergreen broadleaf forests, where extreme species diversity and structural heterogeneity complicate both measurement and upscaling. They also highlight the difficulty of comparing leaf-level trait measurements with grid-cell effective parameters inferred by the model. Given these ambiguities, we removed LCMA from the quantitative comparisons in Fig. 4 and instead provide a focused discussion of its limitations and potential sources of error in Text S6.

Despite these constraints, the inferred parameters show consistent large-scale spatial agreement with independent benchmarks across all evaluated traits, even when absolute magnitudes and fine-scale patterns diverge. This convergence supports the physical realism of the retrieved parameters at the spatial scales for which the model is designed. DifferLand also offers a flexible framework that can readily incorporate additional observational constraints or observation-informed priors as they become available. This capability positions the model to progressively strengthen the robustness of retrieved ecological parameters and to improve process representation as new data sources emerge.

In Text S6: We further evaluated additional parameters introduced in the revised manuscript. The retrieved temperature sensitivity of heterotrophic respiration (Q10) was compared with Q10 values inferred from soil organic carbon stocks using the Carnegie–Ames–Stanford Approach under equilibrium assumptions (Fig. S). DifferLand retrieves biome-scale Q10 values that are consistently lower than those inferred by Zhou et al. (2009), but show a similar ordering across biomes, with higher Q10 in colder ecosystems and lower Q10 in warmer ones. We note that substantial differences in model structure, assimilated datasets, and modeling assumptions may introduce biases that limit comparability.

For belowground allocation, we compared the retrieved fine-root-to-total biomass fraction with the one of the few globally available benchmarks, which reports total root (fine plus coarse) to total biomass(Huang

et al., 2021). Despite the structural mismatch between these quantities, both the retrieved and benchmark datasets show a systematic decline in root allocation with increasing precipitation. When stratified by precipitation quantiles in log space, both datasets exhibit decreasing median root allocation from arid to humid regimes, although the retrieved fine-root fraction is consistently lower in magnitude, as expected given that coarse roots are treated as part of woody biomass in DifferLand (Fig. S). This agreement in gradient, despite differences in definition and scale, indicates that DifferLand captures the large-scale climatic control on belowground investment.

Taken together, these comparisons demonstrate that while direct pointwise or absolute validation is constrained by differences in variable definitions, spatial and temporal scales, and observational availability, the inferred parameters are quantitatively plausible and consistently reproduce large-scale gradients across independent datasets. Although the comparison underscores the limitations of data in certain biomes like tropical forests, we acknowledge that DifferLand has the capacity to readily incorporate additional observations or incorporate observation-informed priors of ecological parameters. This potential allows DifferLand to further enhance the robustness of the retrieved ecological parameters and process dynamics as new observations become available.

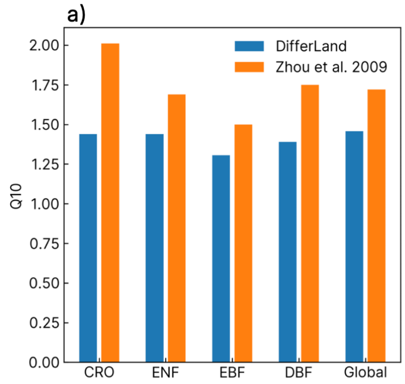

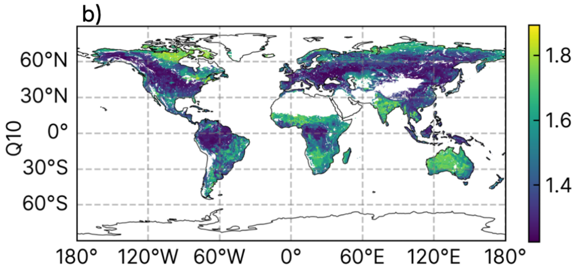

Fig. S47 | a) Comparison of DifferLand-retrieved Q_{10} with Q_{10} inverted from soil organic carbon data using Carnegie-Ames-Stanford Approach under equilibrium conditions (CASA)(Zhou et al., 2009); b) Q10 map retrieved from DifferLand.

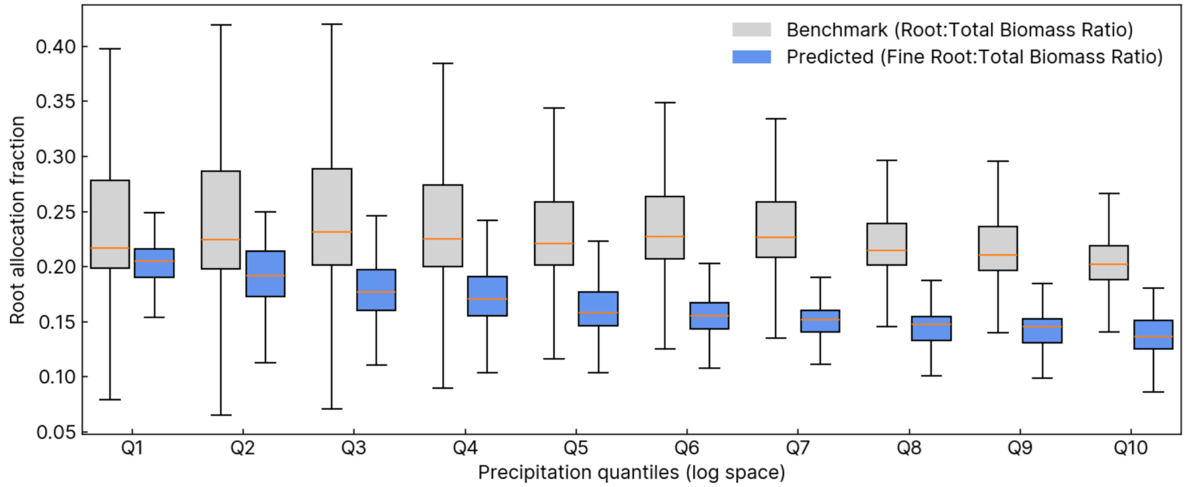

Fig. S48 | Comparison of DifferLand-simulated fine-root allocation fraction with independent observational benchmarks of root (fine + coarse) to total biomass fraction (Huang et al., 2021).

(b) The manuscript would also benefit from supporting the findings in Fig. 4 with observational evidence or laboratory experiments.

Thank you for this suggestion. We have now explicitly evaluated whether the trait–environment relationships diagnosed by the spatialization network are supported by independent observational constraints.

Specifically, we compared the per-PFT relationships retrieved by DifferLand with globally upscaled observational products for (i) V_{cmax25} (Luo et al., 2021), (ii) carbon use efficiency (Luo et al., 2025), and (iii) belowground-to-total live biomass fraction (Huang et al., 2021). For each product and each PFT, we fitted a fourth-degree multivariate polynomial relating the target variable to mean annual temperature, log-transformed mean annual precipitation, and maximum canopy height, using only grid cells in which a single PFT occupies at least 90 percent of the area. These fitted relationships are shown in Fig. S49 and are directly comparable to the conditional SHAP relationships in Fig. 4.

Across variables and PFTs, the large-scale patterns inferred by DifferLand are generally consistent with those emerging from the independent datasets. For example, both DifferLand and the observational products show a systematic decline in photosynthetic efficiency proxies with increasing precipitation, with grasslands and shrublands exhibiting higher values than forests. Carbon use efficiency decreases with increasing temperature and precipitation in both the retrieved relationships and the upscaled dataset, and tropical forests consistently exhibit lower carbon use efficiency than cooler or drier biomes. For belowground allocation, both DifferLand and the observational constraints show reduced root allocation in wetter ecosystems, consistent with a shift toward aboveground investment under less water limitation.

We also observe systematic differences. The dependence of V_{cmax25} on temperature is weaker in the upscaled dataset than in DifferLand, likely because DifferLand's photosynthetic efficiency parameter represents an effective canopy-scale quantity within the aggregated canopy model, whereas V_{cmax25} is derived from site-level leaf measurements and then spatially extrapolated. Similarly, the observational products show weaker dependence on canopy height, plausibly because canopy height is not explicitly used as a predictor in those upscaling efforts and because forests of different heights remain under-sampled.

Overall, these comparisons show that the main trait–environment gradients diagnosed by DifferLand are supported by independent observational constraints at large spatial scales, while the remaining differences are consistent with known structural and semantic mismatches between model parameters and observational proxies. We have added these analyses and discussion to SI Text S6 and Fig. S49.

Line 461-468: These retrieved relationships are broadly consistent with independent upscaled datasets, despite differences due to scale mismatch, observation uncertainties, and different physical meanings between individual plant trait measurements and model parameters representing effective traits at ecosystem level (Fig. S49 & Text S6).

In Text S6: We also compared the trait–environment relationships retrieved by DifferLand (Fig. 4) with relationships inferred from independent globally upscaled datasets (Fig. S49), including V_{cmax25} from Luo et al. (2021), carbon use efficiency from Luo et al. (2025), and the belowground-to-total live biomass fraction from Huang et al. (2021). Across plant functional types and variables, the dominant spatial gradients are broadly similar between the retrieved relationships and those inferred from these independent products. Photosynthetic capacity proxies and carbon use efficiency both tend to decline with increasing mean annual precipitation, with grasslands and shrublands exhibiting higher values than forests and tropical forests exhibiting lower carbon use efficiency than cooler or drier biomes. Carbon use efficiency also shows a clear decline with increasing mean annual temperature, consistent with the patterned captured by the spatialization network. The belowground-to-total biomass fraction likewise decreases in wetter environments, indicating a shift toward aboveground investment under reduced water limitation. These shared patterns indicate that the spatialization network in DifferLand retrieves large-scale gradients that are consistent with those emerging from independent observational syntheses.

At the same time, the strength and shape of the relationships differ in some cases. In particular, the observational products exhibit weaker sensitivities to temperature and canopy height than the retrieved DifferLand parameters, especially for V_{cmax25}. This likely reflects differences in how the quantities are defined and estimated, as the DifferLand parameters represent effective canopy-scale quantities within an aggregated model framework, whereas the observational datasets are constructed by extrapolating site-level measurements using a limited set of predictors that often do not include canopy height. In addition, the observational root allocation product integrates over both fine and coarse roots, whereas DifferLand explicitly represents fine-root allocation. These differences suggest that the comparisons are most informative at the level of large-scale gradients rather than absolute magnitudes or local sensitivities.

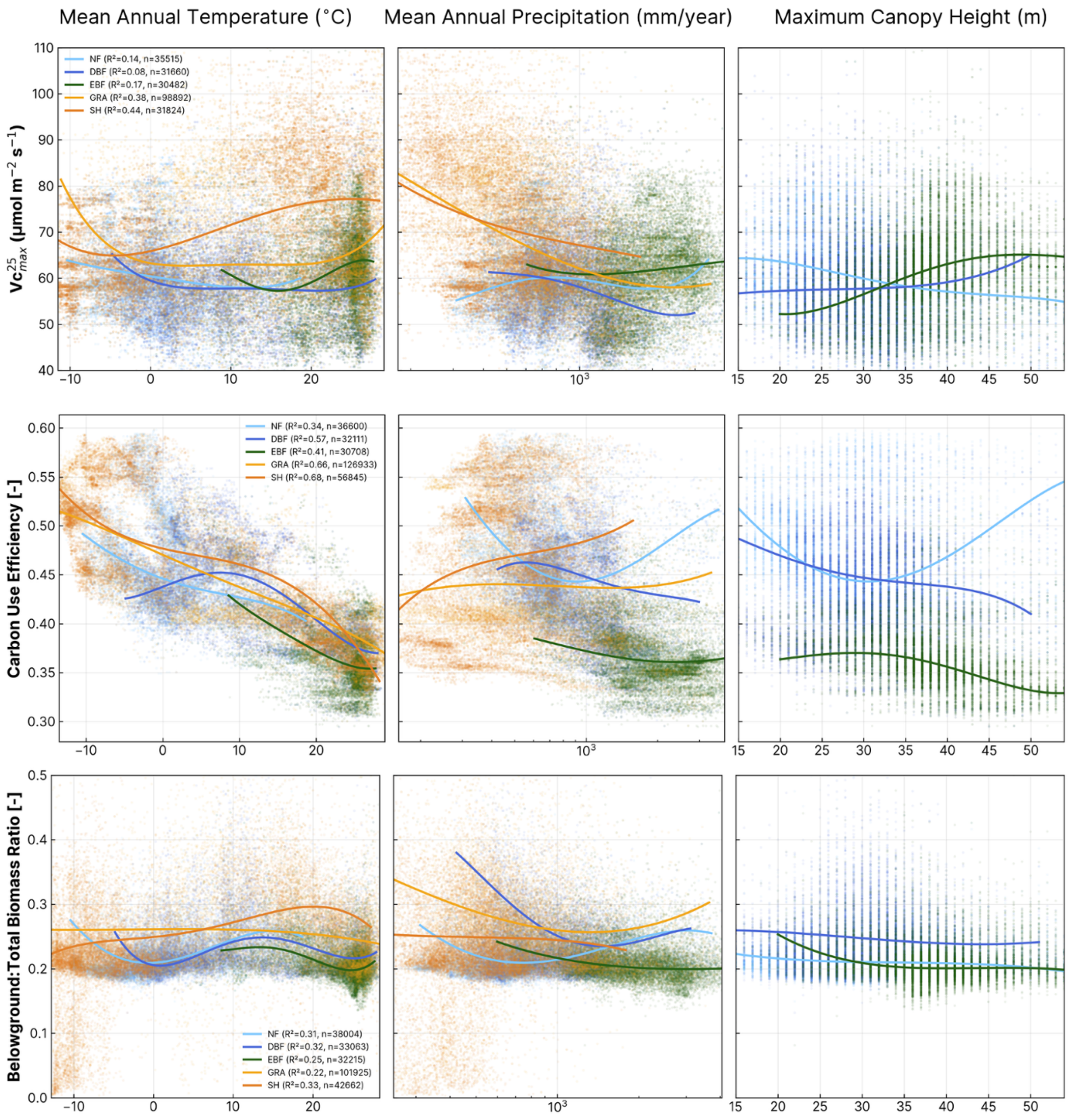

Fig. S49 | Spatial relationships between independently upscaled V_{cmax25} (Luo et al., 2021), carbon use efficiency (Luo et al., 2025), and belowground-to-total live biomass fraction (Huang et al., 2021) and mean annual temperature (MAT), mean annual precipitation (MAP), and maximum canopy height (MCH), shown by plant functional type. Rows correspond to different traits, and columns correspond to the same predictors used in DifferLand. For each trait and PFT, we fitted a fourth-degree multivariate polynomial using MAT, log-transformed MAP, and MCH as predictors, restricting the analysis to 0.25° grid cells in which the PFT occupies at least 90 percent of the area. The number of grid cells and the coefficient of determination are shown in the legend. These fitted relationships are compared with the conditional SHAP relationships in Fig. 4.

(c) Fig. S36 shows low variability across ensembles initialized with different starting values, suggesting robustness of the inferred parameters. However, these values may still be affected by structural errors in the physics-based model, not only in the hydrology components (as discussed in Text S7) but also potentially in any components, which should be discussed. The inferred values and their linkage with environmental variables may also be sensitive to the selection of data constraints. While the authors tested how the selection of data constraints affects relative performance of hybrid models (Fig. S29), its impact on knowledge discovery (Figs. 3-4) remains to be evaluated.

Thank you for this important and constructive comment. We agree that while Fig. S36 (now Fig. S40) demonstrates numerical robustness of the inferred parameters with respect to initialization, this does not preclude systematic biases arising from structural errors in the underlying physics-based model or from the choice of observational constraints.

We have therefore taken two steps in the revision.

First, we have expanded the discussion of potential structural model errors beyond hydrology in Text S7 to explicitly acknowledge that misspecification in other components of the physics-based model, including photosynthesis, respiration, carbon turnover, and belowground carbon, can also bias the inferred parameter values and their apparent environmental dependencies. We now discuss how such structural errors can be partially absorbed by the inferred parameters.

In Text S7: In addition to these discussed errors, misspecification and process simplification in other components of the mechanistic model, including photosynthesis, respiration, carbon turnover, and belowground carbon, can also bias the inferred parameter values and their apparent environmental dependencies. These structural errors can be partially absorbed by the inferred parameters and the result in biased environment-parameter relationships. Together, these limitations underscore priority areas for future hybrid land model development, highlighting the need for more mechanistic yet flexible formulations to improve predictions of ecosystem responses under a changing climate.

Second, we have added an analysis to evaluate how the choice of observational constraints affects the spatial structure of the latent ecological parameters. We summarize the results by comparing the principal components of the retrieved plant trait fields across these alternative constraint configurations The dominant modes of trait variability and their large-scale spatial patterns are generally consistent across datasets despite their differences in local spatial patterns, indicating that the main spatial ecological gradients identified in Figs. 3-4 are robust to these data substitutions.

We have added this discussion and the new sensitivity analysis to the revised manuscript, and we believe it addresses the concern regarding the influence of structural model error and data-constraint selection on the robustness and interpretation of the inferred ecological relationships.

In Text S4: Using the latent parameters inferred from the sensitivity experiments that assimilated alternative observational constraints, including the ΔNBE configuration using CAMS-NBE instead of CMS-Flux (Fig. S1), the ΔLAI configuration using Copernicus LAI instead of MODIS LAI (Fig. S2), and the ΔEC (Fig. S3) configuration excluding eddy-covariance flux data, we evaluated how these choices affect the emergent structure of the parameter space. For each sensitivity configuration, we applied the same principal component analysis as in Fig. 3 to the selected latent parameters, using identical preprocessing, standardization, and spatial masking. While the relative ordination of individual parameters in the principal component space exhibits minor variations across configurations, the large-scale spatial patterns associated with each principal component and the fraction of variance they explain are highly consistent with the baseline case. This indicates that the dominant modes of covariation among latent parameters are robust to the choice of assimilated data streams and are not driven by any single observational constraint.

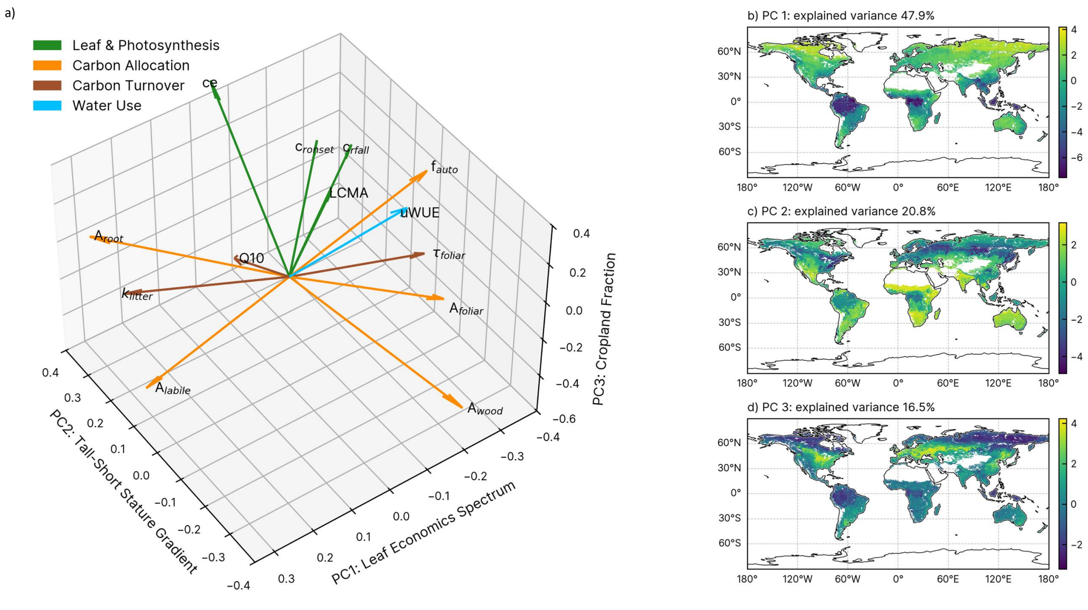

Fig. S1 | Principal Component Analysis of the latent parameters under the ΔNBE experiment, with the same notations as Fig. 3 in the main text. a) shows the loadings that project each parameter onto the first three PCs are plotted in the 3D plot. b-d) shows the spatial distribution of PC1–PC3 scores projected from

the spatialization neural network onto the PCA axes, with the proportion of variance explained by each component labeled.

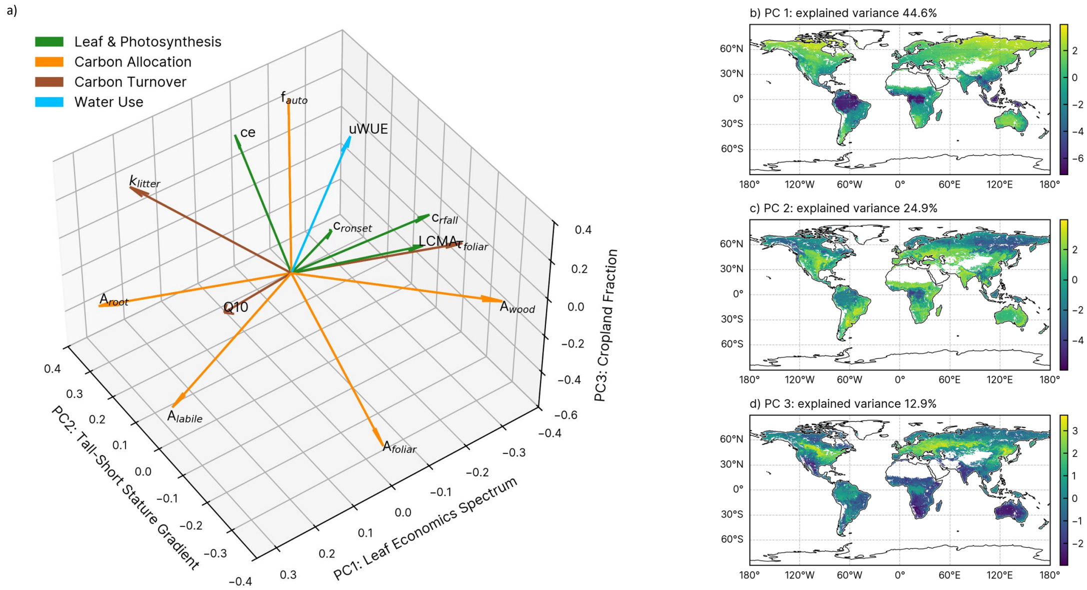

Fig. S2 | Same as Fig. S1 but for the ΔLAI sensitivity experiment.

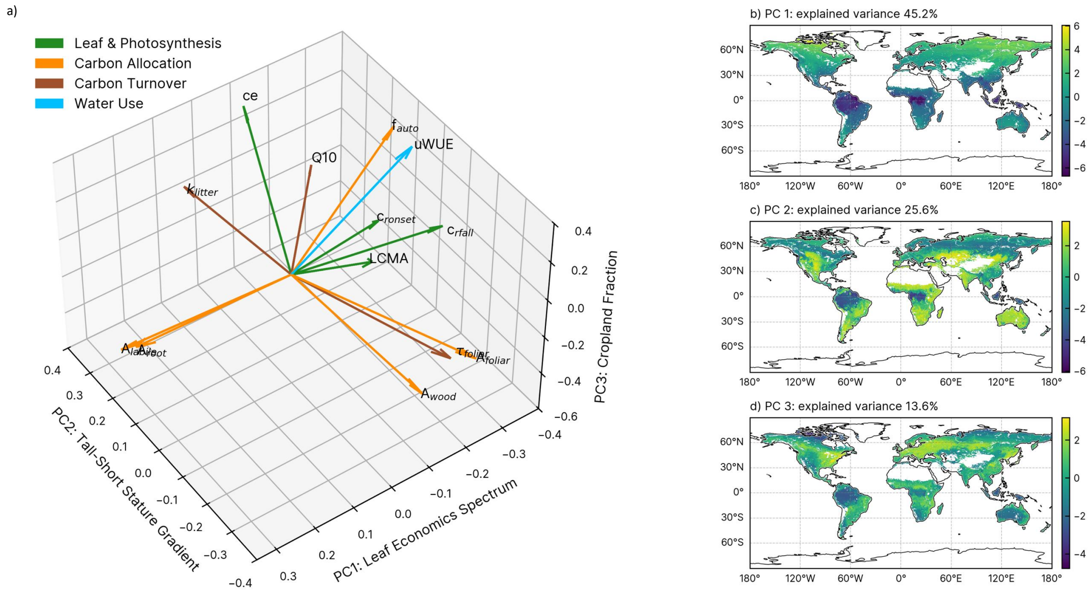

Fig. S3 | Same as Fig. S1 but for the ΔEC sensitivity experiment.

2. Improved model performance vs increased number of predictors and degrees of freedom: Although the full set (PFT+CLIM+SOIL+AGE) performs best compared to the PFT-only setting, in many cases the improvement is incremental (Figs. S15-18). Have the authors accounted for the increased degrees of freedom associated with using substantially more predictors? How might this affect the comparison and interpretation of the relative importance of PFT versus other spatialization variables?

Thanks a lot for raising this point. To ensure that the improvement in model performance is not solely a result of the increased degrees of freedom in the model, we performed an experiment where we kept all the PFT predictors (as our baseline) by randomly permutating the CLIM, AGE, and SOIL predictors spatially prior to training the model (PFT + perm[CLIM, SOIL, AGE]). By applying the permutation on these additional predictors, we disrupt the spatial structure of these variables while keeping the total degrees of freedom of the model (i.e., the number of parameters in the neural network) the same as the PFT+CLIM+SOIL+AGE configuration. We found that the model with the permuted CLIM, SOIL, AGE parameters offer no performance gain on the test dataset compared with the PFT-only baseline, suggesting that expanded degrees of freedom alone is unlikely to explain the performance gain.

Line 242-244: We also confirmed that the improved performance when including additional environmental variables cannot be explained solely by the increased degrees of freedom in the spatialization network (Fig. S21).

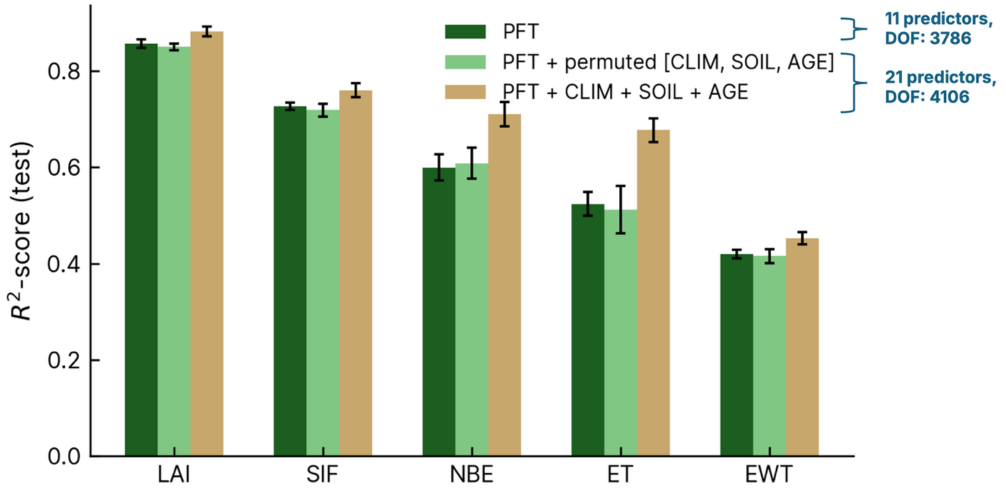

Fig. S4 | Evaluating against the null hypothesis that increasing degrees of freedom in the spatialization neural network *per se* explains the model performance gain compared with the PFT-online baseline. We randomly permuted the CLIM, SOIL, and AGE predictors to destroy the spatial pattern but kept the same input dimensionality and parameter count in the neural network as the PFT+CLIM+SOIL+AGE configuration. We found the PFT + permuted[CLIM, SOIL, AGE] setup offered no performance gain compared to the PFT-only baseline, rejecting the null hypothesis.

More broadly, the rationale for choosing these spatialization variables should be clarified. Is there any variable selection procedure? Additionally, could model performance be artificially improved simply by including more predictors that introduce spatial variability, even if some of them are only weakly or incidentally related to the underlying processes?

Thanks a lot for this important question. We adopted an iterative approach to feature selection by beginning with a basic set of predictors (i.e. the PFT-baseline) and add in additional groups of predictors only when there is demonstrated performance gain. This procedure is detailed in the hierarchical partition section with results reported in S15-17. For each group of CLIM, SOIL, and AGE predictors, we use only variables that have demonstrated clear relationship with plant traits in the literature (Bao et al., 2023; Famiglietti et al., 2023; Migliavacca et al., 2021). Our approach prioritizes parsimony and interpretability. For instance, while some studies have found the use of multiple bioclimatic variables may improve prediction skills (Bao et al., 2023), we opt to use only the climatological mean annual temperature and mean annual prediction to reduce collinearity.

To test whether model performance may be artificially improved by including variables that merely introduce spatial variability, including those that are incidentally related to the underlying dynamics, we conducted an experiment where we used longitude and latitude in addition to the PFT (PFT+LONLAT) as predictors. We then trained both the PFT+LONLAT configuration and the PFT+CLIM+SOIL+AGE configurations on pixels in Australia, Eurasia, Africa, and South America, and evaluated the model on North America as an out-of-sample distribution. A model trained on PFT+LONLAT would be able to capture the within PFT variations by "memorizing" the spatial variability in the training dataset but not the true physical environment-trait relationships as the coordinates. However, we found that while both configurations effectively fitted the data on the training pixels, only the PFT+CLIM+SOIL+AGE configuration generalized reasonably well on the held-out continent, while the PFT+LONLAT configuration performed poorly in North America as it is unable to extrapolate outside of the training domain. We do note that the out-of-sample performance is slightly lower for GRACE equivalent water thickness and aboveground biomass, which is expected as the model has no prior knowledge of disturbance history and human water extraction in an unseen continent that affect these variables. Overall, the robust out-of-sample generalization of the PFT+CLIM+SOIL+AGE configuration suggests the retrieved spatial relationship is largely reflecting the underlying dynamics rather than merely spatial autocorrelation. We included the out-of-sample analysis in the SI and referenced it in the methods section.

Line 844-850: Overall, our predictor selection process prioritized parsimony and interpretability by iteratively adding spatial predictors that enhance model performance across multiple observational

constraints (Fig. S14-17). While we recognize that incorporating additional predictors, such as bioclimatic variables, might further enhance prediction, we opted to use a limited set of climatological predictors to minimize confounding effects and maintain interpretability. We excluded variables that are only incidentally correlated with spatial variation but do not represent causal controls on the parameters (for example, latitude and longitude), because such predictors encourage the model to learn spurious spatial structure and thereby reduce its ability to generalize in out of sample spatial extrapolation (Fig. S22).

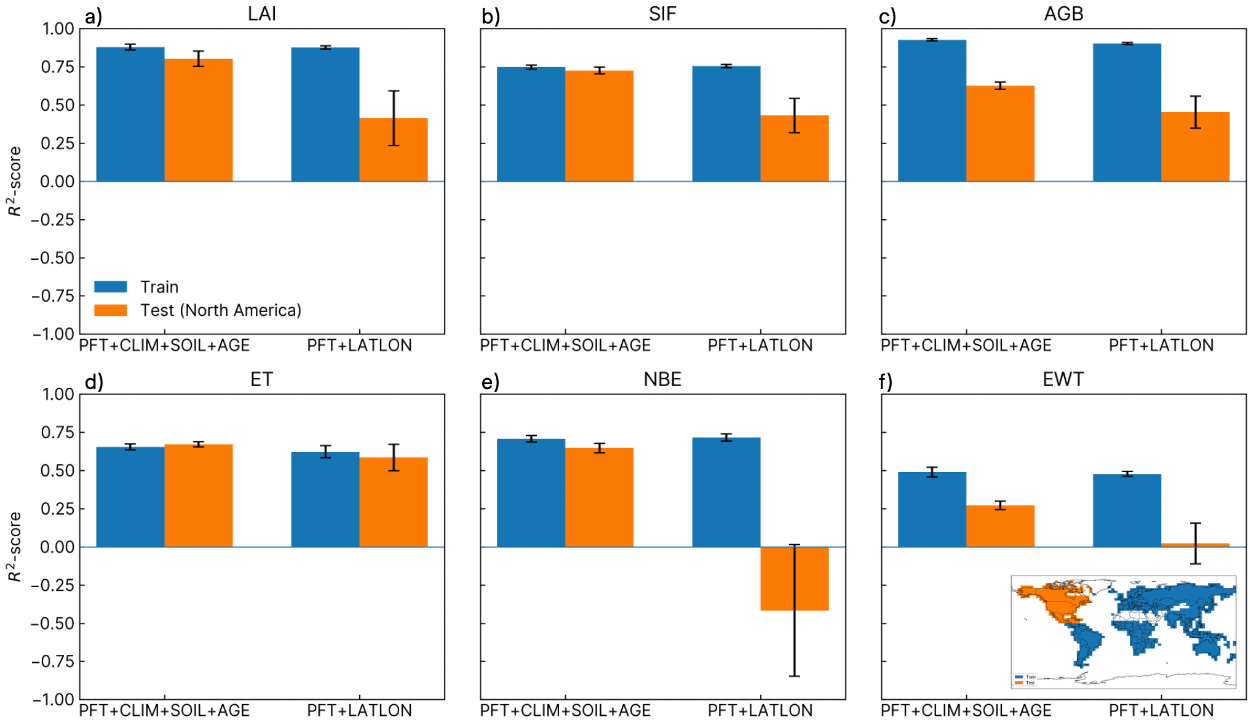

Fig. S5 | Evaluating DifferLand against the null hypothesis of artificial performance gain due to spatial autocorrelation and confounding variables. The PFT+CLIM+SOIL+AGE set up was compared with the the PFT+LATLON setup, which uses the latitude and longitude coordinates in addition to the PFT variables. We trained both models with only pixels in Eurasia, Oceania, Africa, and South America. We then evaluated model performance on North America as an out-of-sample generalization test. We found that although the PFT+CLIM+SOIL+AGE and PFT+LATLON configurations achieved near equal performance on the training set, the PFT+LATLON configurations that simply memorized the within-PFT spatial variations in the training set generalized poorly on the out-of-sample test. In contrast, the PFT+CLIM+SOIL+AGE configuration generalized well in the unseen continent.

I appreciate that the authors tested different datasets for calibration. However, the spatialization predictors may also have uncertainties, especially forest age. It may lead to incorrect interpretation for predictor attribution. In addition to the MPI-BGC data, the authors may consider testing with the global forest age dataset (GFAD) (https://doi.pangaea.de/10.1594/PANGAEA.897392).

We thank the reviewer for this helpful suggestion. Following this comment, we repeated the spatialization analysis using the Global Forest Age Dataset in place of the MPI-BGC forest age product. We found the model performance results are generally consistent with those obtained using the MPI-BGC dataset. While we acknowledge that uncertainties in the forest age datasets may affect interpretation of parameter attribution, in absence of independent datasets it is difficult to conclude which dataset is categorically better, and thus we decide to use the MPI-BGC data as default in the current study.

The comparison between simulated NBE from DifferLand and TRENDY DGVMs is interesting (Figs. S8-S12). However, since DifferLand is calibrated using CMS NBE, using CMS NBE as the reference may not represent an entirely independent comparison. The description in Lines 236-239 may therefore be inaccurate.

We agree with the reviewer that the comparison against CMS-NBE is not an entirely independent assessment, since DifferLand is calibrated against CMS-NBE. Our goal in making this comparison with TRENDY DGVM it not to claim whether DifferLand is superior to the more structurally sophisticated TRENDY DGVMs, but rather to indicate that parameter uncertainties dominate decadal land-atmosphere fluxes, and hybrid model-data fusion can be used to effectively reduce parameter uncertainties and bring the model closer in alignment with observations—despite that DifferLand is structurally simplified compared with the state-of-the art models. In the revised manuscript, we used more precise language to highlight the fact that DifferLand has been calibrated with CMS-Flux and further clarify the purpose of our comparison.

Line 287-294: Despite its comparatively simplified process representation, DifferLand achieved significantly lower root mean squared errors and comparable correlation with the atmospheric inversion dataset than the much more structurally sophisticated dynamic global vegetation models (DGVMs) in the TRENDYv12 S3 ensemble (Friedlingstein et al., 2023) (Table S5), both globally and regionally, after assimilating the CMS-Flux (Fig. S7-11). These results suggest that uncertainties in model parameters are a major source of error in simulating land–atmosphere carbon fluxes at decadal timescales, and that learning trait-environment relationships with differentiable model-data fusion can effectively reduce parameter uncertainties and improve alignment with observational constraints.

L986-987: MCD12C1 is provided at 0.05 degree, not 500m.
Thank you for pointing this out. We apologize for the typo. MCD12C1 is indeed provided at 0.05°, with the fractional area of each land cover class derived from aggregating the underlying 500 m MODIS land

cover product within each 0.05° grid cell. We have corrected this in the manuscript. We further resampled the 0.05° product to 0.25° to match the modeling grid.

Line 809-811: PFT fractions are derived from MCD12C1.v061 MODIS/Terra + Aqua Yearly Land Cover Type (Friedl & Sulla-Menashe, 2022), with the percentage of underlying 500m classes aggregated to 0.05° grid cells. We further averaged the product to 0.25° to match the modeling grid.

Reviewer #3 (Remarks to the Author):

I co-reviewed this manuscript with one of the reviewers who provided the listed reports. This is part of the Nature Communications initiative to facilitate training in peer review and to provide appropriate recognition for Early Career Researchers who co-review manuscripts.

Thank you for reviewing our manuscript. Your comments have helped us substantially improve our work.

Reviewer #4 (Remarks to the Author):

The authors have addressed all of my concerns adequately. I look forward to seeing how the scientific community reacts to this work.

We thank the reviewer for the positive evaluation of our manuscript and the insightful suggestions that helped us substantially improve our work.

Reviewer #5 (Remarks to the Author):

I co-reviewed this manuscript with one of the reviewers who provided the listed reports. This is part of the Nature Communications initiative to facilitate training in peer review and to provide appropriate recognition for Early Career Researchers who co-review manuscripts.

Thank you for reviewing our manuscript. Your comments have helped us substantially improve our work.

References

Bao, S., Alonso, L., Wang, S., Gensheimer, J., De, R., & Carvalhais, N. (2023). Toward Robust Parameterizations in Ecosystem-Level Photosynthesis Models. *Journal of Advances in Modeling Earth Systems*, *15*(8), e2022MS003464. https://doi.org/10.1029/2022MS003464

Bengio, Y., Courville, A., & Vincent, P. (2013). Representation Learning: A Review and New Perspectives. *IEEE Transactions on Pattern Analysis and Machine Intelligence*, *35*(8), 1798–1828. https://doi.org/10.1109/TPAMI.2013.50

Bloom, A. A., Exbrayat, J.-F., Van Der Velde, I. R., Feng, L., & Williams, M. (2016). The decadal state of the terrestrial carbon cycle: Global retrievals of terrestrial carbon allocation, pools, and residence times. *Proceedings of the National Academy of Sciences*, *113*(5), 1285–1290.

Díaz, S., Kattge, J., Cornelissen, J. H. C., Wright, I. J., Lavorel, S., Dray, S., et al. (2016). The global spectrum of plant form and function. *Nature*, *529*(7585), 167–171. https://doi.org/10.1038/nature16489

Famiglietti, C. A., Worden, M., Quetin, G. R., Smallman, T. L., Dayal, U., Bloom, A. A., et al. (2023). Global net biome CO2 exchange predicted comparably well using parameter–environment relationships and plant functional types. *Global Change Biology*, *29*(8), 2256–2273. https://doi.org/10.1111/gcb.16574

Fan, H., Bai, L., Fei, B., Xiao, Y., Chen, K., Liu, Y., et al. (2025, July 8). Physically Consistent Global Atmospheric Data Assimilation with Machine Learning in Latent Space. arXiv. https://doi.org/10.48550/arXiv.2502.02884

Fang, J., Lian, X., Ryu, Y., Jeong, S., Jiang, C., & Gentine, P. (2025). A long-term reconstruction of a global photosynthesis proxy over 1982–2023. *Scientific Data*, *12*(1), 372. https://doi.org/10.1038/s41597-025-04686-6

Floryan, D., & Graham, M. D. (2022). Data-driven discovery of intrinsic dynamics. *Nature Machine Intelligence*, *4*(12), 1113–1120. https://doi.org/10.1038/s42256-022-00575-4

Franks, S. J., Weber, J. J., & Aitken, S. N. (2014). Evolutionary and plastic responses to climate change in terrestrial plant populations. *Evolutionary Applications*, *7*(1), 123–139. https://doi.org/10.1111/eva.12112

Friedl, M., & Sulla-Menashe, D. (2022). MODIS/Terra+Aqua Land Cover Type Yearly L3 Global 0.05Deg CMG V061 [Data set]. https://doi.org/10.5067/MODIS/MCD12C1.061

Friedlingstein, P., O'sullivan, M., Jones, M. W., Andrew, R. M., Bakker, D. C., Hauck, J., et al. (2023). Global carbon budget 2023. *Earth System Science Data*, *15*(12), 5301–5369.

Huang, Y., Ciais, P., Santoro, M., Makowski, D., Chave, J., Schepaschenko, D., et al. (2021). A global map of root biomass across the world's forests. *Earth System Science Data*, *13*(9), 4263–4274. https://doi.org/10.5194/essd-13-4263-2021

Huang, Y., Song, X., Wang, Y.-P., Canadell, J. G., Luo, Y., Ciais, P., et al. (2024). Size, distribution, and vulnerability of the global soil inorganic carbon. *Science*, *384*(6692), 233–239. https://doi.org/10.1126/science.adi7918

Joswig, J. S., Wirth, C., Schuman, M. C., Kattge, J., Reu, B., Wright, I. J., et al. (2021). Climatic and soil factors explain the two-dimensional spectrum of global plant trait variation. *Nature Ecology & Evolution*, *6*(1), 36–50. https://doi.org/10.1038/s41559-021-01616-8

Kelly, A. E., & Goulden, M. L. (2008). Rapid shifts in plant distribution with recent climate change. *Proceedings of the National Academy of Sciences*, *105*(33), 11823–11826. https://doi.org/10.1073/pnas.0802891105

Liu, H., Mi, Z., Lin, L., Wang, Y., Zhang, Z., Zhang, F., et al. (2018). Shifting plant species composition in response to climate change stabilizes grassland primary production. *Proceedings of the National Academy of Sciences*, *115*(16), 4051–4056. https://doi.org/10.1073/pnas.1700299114

Liu, J., Baskaran, L., Bowman, K., Schimel, D., Bloom, A. A., Parazoo, N. C., et al. (2021). Carbon Monitoring System Flux Net Biosphere Exchange 2020 (CMS-Flux NBE 2020). *Earth System Science Data*, *13*(2), 299–330. https://doi.org/10.5194/essd-13-299-2021

Luo, X., Keenan, T. F., Chen, J. M., Croft, H., Colin Prentice, I., Smith, N. G., et al. (2021). Global variation in the fraction of leaf nitrogen allocated to photosynthesis. *Nature Communications*, *12*(1), 4866. https://doi.org/10.1038/s41467-021-25163-9

Luo, X., Zhao, R., Chu, H., Collalti, A., Fatichi, S., Keenan, T. F., et al. (2025). Global variation in vegetation carbon use efficiency inferred from eddy covariance observations. *Nature Ecology & Evolution*. https://doi.org/10.1038/s41559-025-02753-0

Lusk, D., Wolf, S., Svidzinska, D., Dormann, C. F., Kattge, J., Bruelheide, H., et al. (2025, March 13). From smartphones to satellites: Uniting crowdsourced biodiversity monitoring and Earth observation to fill the gaps in global plant trait mapping. Ecology. https://doi.org/10.1101/2025.03.10.641660

Migliavacca, M., Musavi, T., Mahecha, M. D., Nelson, J. A., Knauer, J., Baldocchi, D. D., et al. (2021). The three major axes of terrestrial ecosystem function. *Nature*. https://doi.org/10.1038/s41586-021-03939-9

Moreno-Martínez, Á., Camps-Valls, G., Kattge, J., Robinson, N., Reichstein, M., Van Bodegom, P., et al. (2018). A methodology to derive global maps of leaf traits using remote sensing and climate data. *Remote Sensing of Environment*, *218*, 69–88. https://doi.org/10.1016/j.rse.2018.09.006

Quetin, G. R., Famiglietti, C. A., Dadap, N. C., Bloom, A. A., Bowman, K. W., Diffenbaugh, N. S., et al. (2023). Attributing past carbon fluxes to CO_2 and climate change: Respiration response to CO_2 fertilization shifts regional distribution of the carbon sink. *Global Biogeochemical Cycles*. https://doi.org/10.1029/2022GB007478

Robinson, N., Drever, R., Gibbs, D., Lister, K., Esquivel-Muelbert, A., Heinrich, V., et al. (2024). Protect young secondary forests for optimum carbon removal.

Tao, F., Huang, Y., Hungate, B. A., Manzoni, S., Frey, S. D., Schmidt, M. W. I., et al. (2023). Microbial carbon use efficiency promotes global soil carbon storage. *Nature*. https://doi.org/10.1038/s41586-023-06042-3

Tenenbaum, J. B., Silva, V. D., & Langford, J. C. (2000). A Global Geometric Framework for Nonlinear Dimensionality Reduction. *Science*, *290*(5500), 2319–2323. https://doi.org/10.1126/science.290.5500.2319

Terrer, C., Jackson, R. B., Prentice, I. C., Keenan, T. F., Kaiser, C., Vicca, S., et al. (2019). Nitrogen and phosphorus constrain the CO2 fertilization of global plant biomass. *Nature Climate Change*, *9*(9), 684–689. https://doi.org/10.1038/s41558-019-0545-2

Wright, I. J., Dong, N., Maire, V., Prentice, I. C., Westoby, M., Díaz, S., et al. (2017). Global climatic drivers of leaf size. *Science*, *357*(6354), 917–921. https://doi.org/10.1126/science.aal4760

Yang, H., Ciais, P., Frappart, F., Li, X., Brandt, M., Fensholt, R., et al. (2023). Global increase in biomass carbon stock dominated by growth of northern young forests over past decade. *Nature Geoscience*, *16*(10), 886–892. https://doi.org/10.1038/s41561-023-01274-4

Zhou, T., Shi, P., Hui, D., & Luo, Y. (2009). Global pattern of temperature sensitivity of soil heterotrophic respiration (Q10) and its implications for carbon-climate feedback. *Journal of Geophysical Research: Biogeosciences*, *114*(G2). https://doi.org/10.1029/2008JG000850
